# Anion-Based Self-assembly of Resorcin[4]arenes and Pyrogallol[4]arenes

**DOI:** 10.1021/jacs.1c11793

**Published:** 2022-03-11

**Authors:** Monika Chwastek, Piotr Cmoch, Agnieszka Szumna

**Affiliations:** Institute of Organic Chemistry, Polish Academy of Sciences, Kasprzaka 44/52, 01-224 Warsaw, Poland

## Abstract

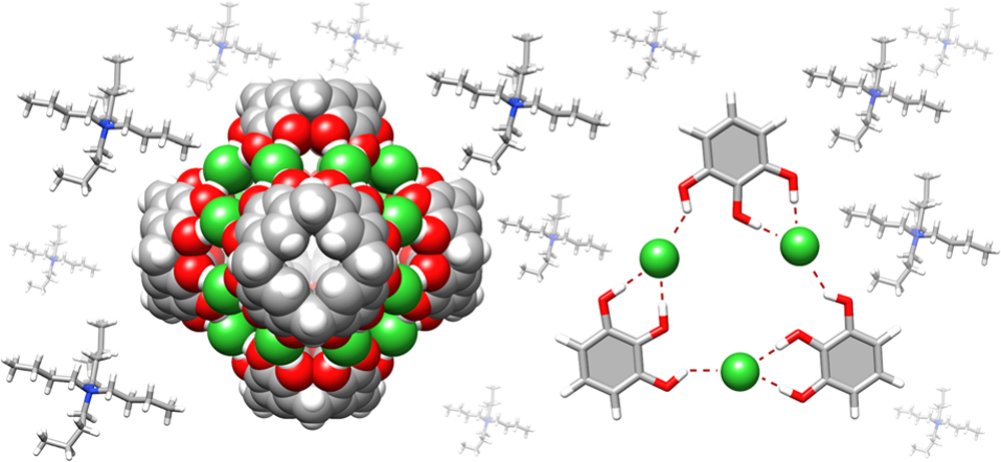

Spatial sequestration of molecules is a prerequisite for the complexity of biological systems, enabling the occurrence of numerous, often non-compatible chemical reactions and processes in one cell at the same time. Inspired by this compartmentalization concept, chemists design and synthesize artificial nanocontainers (capsules and cages) and use them to mimic the biological complexity and for new applications in recognition, separation, and catalysis. Here, we report the formation of large closed-shell species by interactions of well-known polyphenolic macrocycles with anions. It has been known since many years that C-alkyl resorcin[4]arenes (**R4C**) and C-alkyl pyrogallol[4]arenes (**P4C**) narcissistically self-assemble in nonpolar solvents to form hydrogen-bonded capsules. Here, we show a new interaction model that additionally involves anions as interacting partners and leads to even larger capsular species. Diffusion-ordered spectroscopy and titration experiments indicate that the anion-sealed species have a diameter of >26 Å and suggest stoichiometry (**M**)_6_(X^–^)_24_ and tight ion pairing with cations. This self-assembly is effective in a nonpolar environment (THF and benzene but not in chloroform), however, requires initiation by mechanochemistry (dry milling) in the case of non-compatible solubility. Notably, it is common among various polyphenolic macrocycles (**M**) having diverse geometries and various conformational lability.

## Introduction

**Figure 1:**
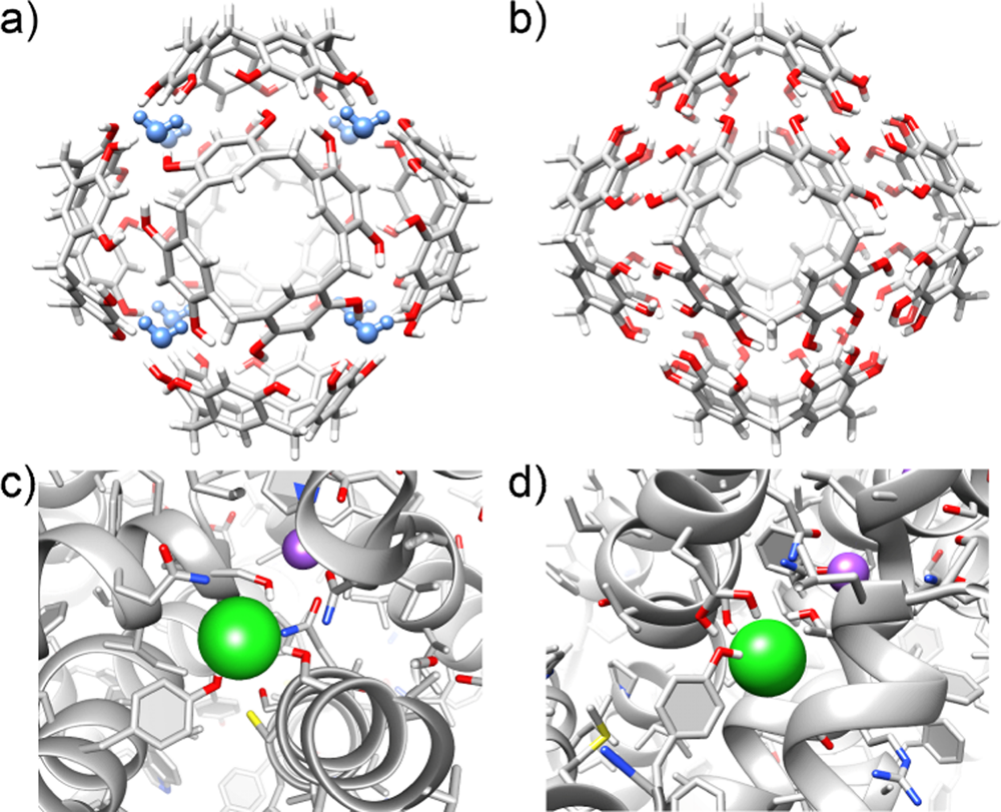
State of the art: previously known hexameric capsules (a) (**R4C**)_6_(H_2_O)_8_ (ref (1)), (b) (**P**_**4**_**C**)_6_ (ref (2)), and (c, d) chloride binding sites found in proteins (refs (15) and (16)).

Complexity requires spatial organization. To perform various chemical reactions or physical processes (recognition, separation, and catalysis), nature has evolved compartmentalization strategies that utilize tailored protein cavities or various cellular containers. Chemists, inspired by nature, utilize synthetic building blocks to construct synthetic organizational systems like capsules and cages. Hexameric capsules (**R4C**)_6_(H_2_O)_8_ and (**P**_**4**_**C**)_6_ (Figure 1a,b) are one of the largest and, at the same time, the easiest to obtain artificial capsules based on hydrogen bonds.^1,2^ They spontaneously form by interactions between polyphenolic macrocycles [C-alkyl resorcin[4]arenes (**R**_**4**_**C**) or C-alkyl progallol[4]arenes (**P**_**4**_**C**) Figure 1a,b], enclosing >1000 Å^3^of the internal space. These hexameric capsules (found in the solid state^1,2^ and low-polarity solvents^3^) have unique encapsulation properties,^4^ exhibit high-fidelity self-sorting,^5^ and amazing enzyme-like catalytic activity.^6−8^ They are nowadays considered the classics of supramolecular chemistry.^6d,9−12^ After many years of extensive studies, it seems that these macrocycles carry no mysteries. However, our recent studies performed for related compounds ([5]arenes^13^) demonstrated a new interaction model that has not been known before for polyphenolic macrocycles. It has been found that [5]arenes are capable of forming capsules via hydrogen bonds between hydroxyl groups (OH) and anions.^14^ These findings inspired us to re-visit interactions between a series of well-known [4]arenes and anions to explore the universal character of such interactions and test the possibility of the formation of new capsular structures using old building blocks.

**Figure 2:**
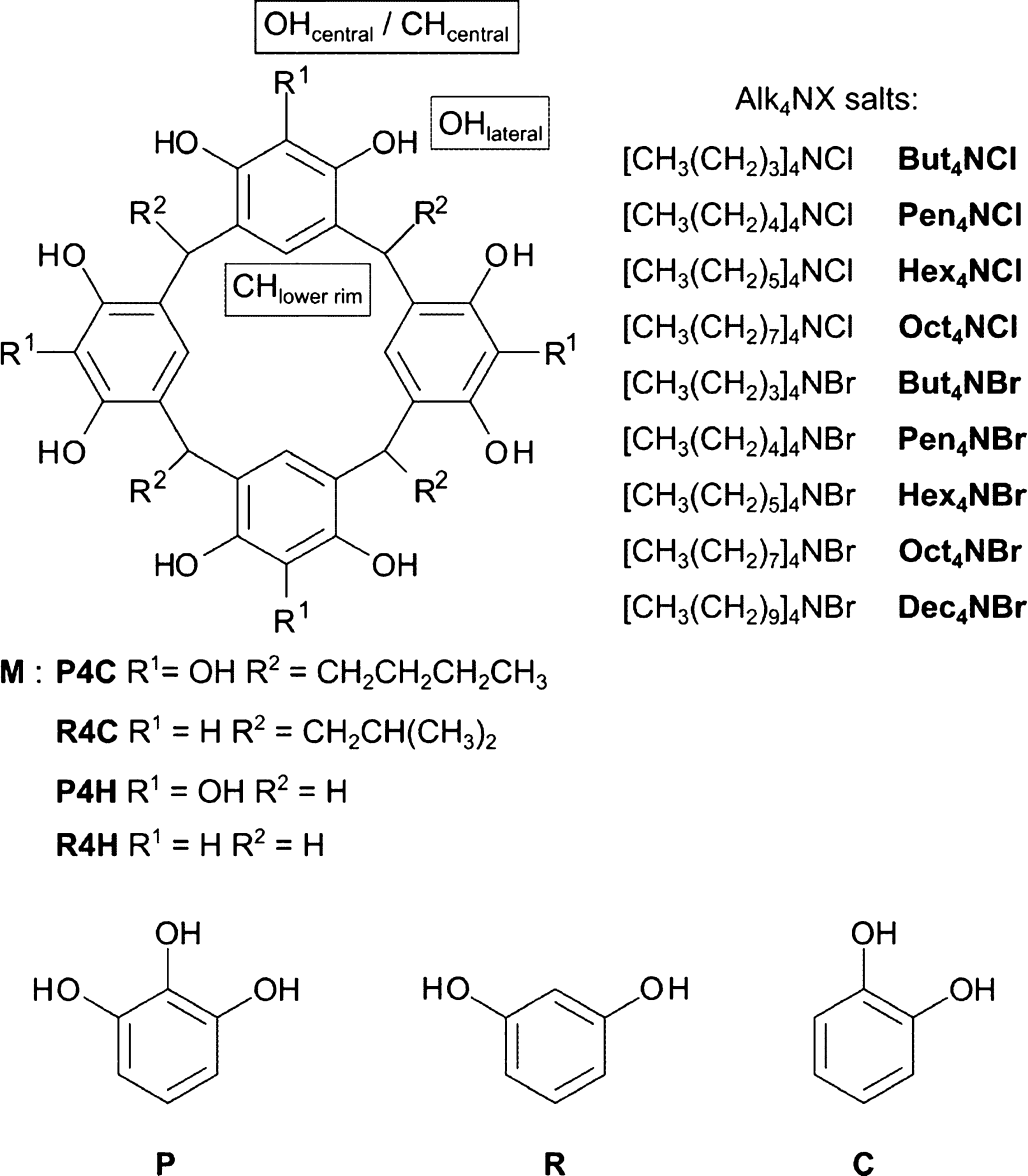
Chemical structures of the compounds used in this work and notation of NMR signals.

The current studies take additional inspiration from the analysis of anion binding sites in proteins (e.g., in chloride-dependent neurotransmiter sodium symporters^15,16^) in which tyrosine or serine (the OH-containing amino acids) are frequently found and OH-anion interactions are common (Figure 1c,d). There is also a growing appreciation of the strength of OH-anion interactions in the field of artificial anion receptors.^17^ The non-innocent role of anions during encapsulation of small ammonium cations in dimeric resorcin[4]arene capsules and during interactions between halogenated resorcin[4]arenes and tetraalkylammonium cations has also been noticed by the groups of Rissanen, Bayeh, and Schalley.^18−21^ Despite these strong indications, the use of anion-based interactions to assemble polyphenolic macrocycles has been abandoned. In this study, we demonstrate that anion-based self-assembly leads to the formation of large capsular species that possess well-defined structures that are ion-paired with cations. We also show that it is a common phenomenon among many polyphenolic macrocycles (**M**, Figure 2), involving those containing different substitution patterns and having various conformational lability. Anion-based self-assembly is effective in various media, although, in the case of non-compatible solubility, its initiation requires a non-standard approach (we report here the effectiveness of mechanochemistry).

## Results and Discussion

### Interactions with Anions in THF

**Figure 3:**
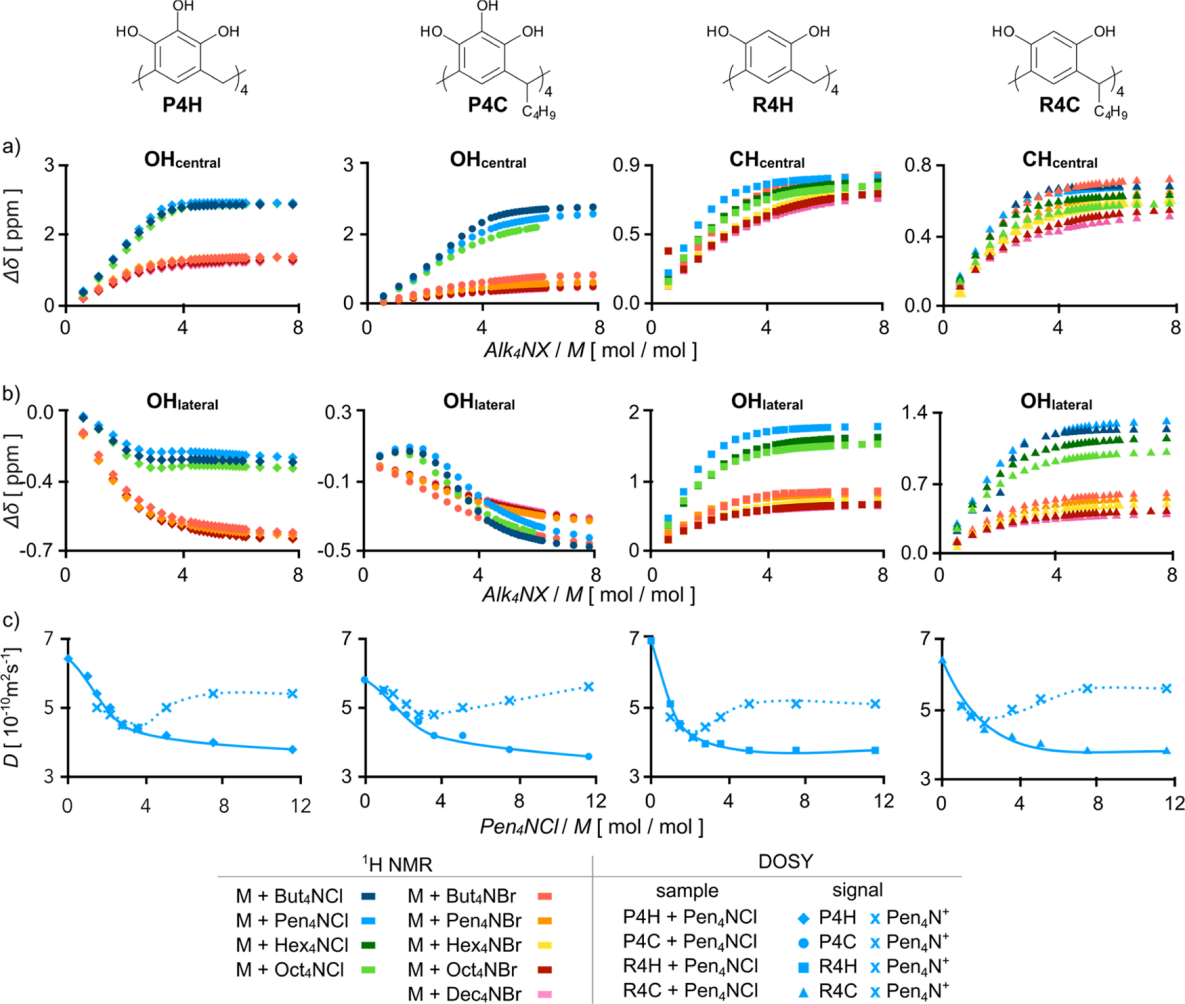
^1^H NMR and DOSY titrations of macrocycles (**M**) **P4H**, **P4C**, **R4H**, and **R4C** with Alk_4_NX salts in [D_8_]THF: (a) changes in chemical shifts (Δδ) for OH/CH central, (b) changes in chemical shifts (Δδ) for OH_lateral_, and (c) changes of diffusion coefficients (*D*) for signals of **M** and Alk4N^+^. All titrations were performed using solutions of analytes, *C*(**M**) = 2.5 mM, and titrant, *C*(**M**) = 2.5 mM + *C*(Alk_4_NX) = 65 mM, at 298 K, 600 MHz.

The interactions between macrocycles (**M**) and tetraalkylammonium salts which serve as sources of anions (Alk_4_NX, Figure 2) were first studied in THF-*d*_8_. In THF-*d*_8_, contrary to CDCl_3_ and benzene, all macrocycles are soluble and exist in monomeric forms, as evidenced from their diffusion coefficients (*D*). Upon addition of Alk_4_NX, the ^1^H NMR signals of upper-rim protons (OH_lateral_ and OH_central_ for **P4C** and **P4H** and OH_lateral_ and CH_central_ for **R4C** and **R4H**) experience substantial downfield shifts (Δδ_max_ ≈ +2.5 ppm, Figure 3a). Although the magnitudes of Δδ values and the trajectories are different for different macrocycles and salts, in all cases, the magnitudes of Δδ depend on the type of anion but remain insensitive to the type of cation, indicating that the interactions are dominated by anions. DOSY spectra^22^ recorded during titrations show a monotonic increase in the average size of species formed by the macrocycles with borderline values that are similar for all macrocycles (Figure 3c). The size of the species, calculated using the Einstein−Stokes equation from *D* values reached after addition of 8 equiv of the salt (see Supporting Information), corresponds to hydrodynamic diameters *d*_H_ = 23 Å for **P4H** and **R4H** and *d*_H_ = 25 Å for **P4C** and **R4C**, where *d*_H_ = **2rH**. In all titration experiments, the plateau is reached at amounts of Alk_4_NX close to 4 equiv per macrocycle.

**Figure 4:**
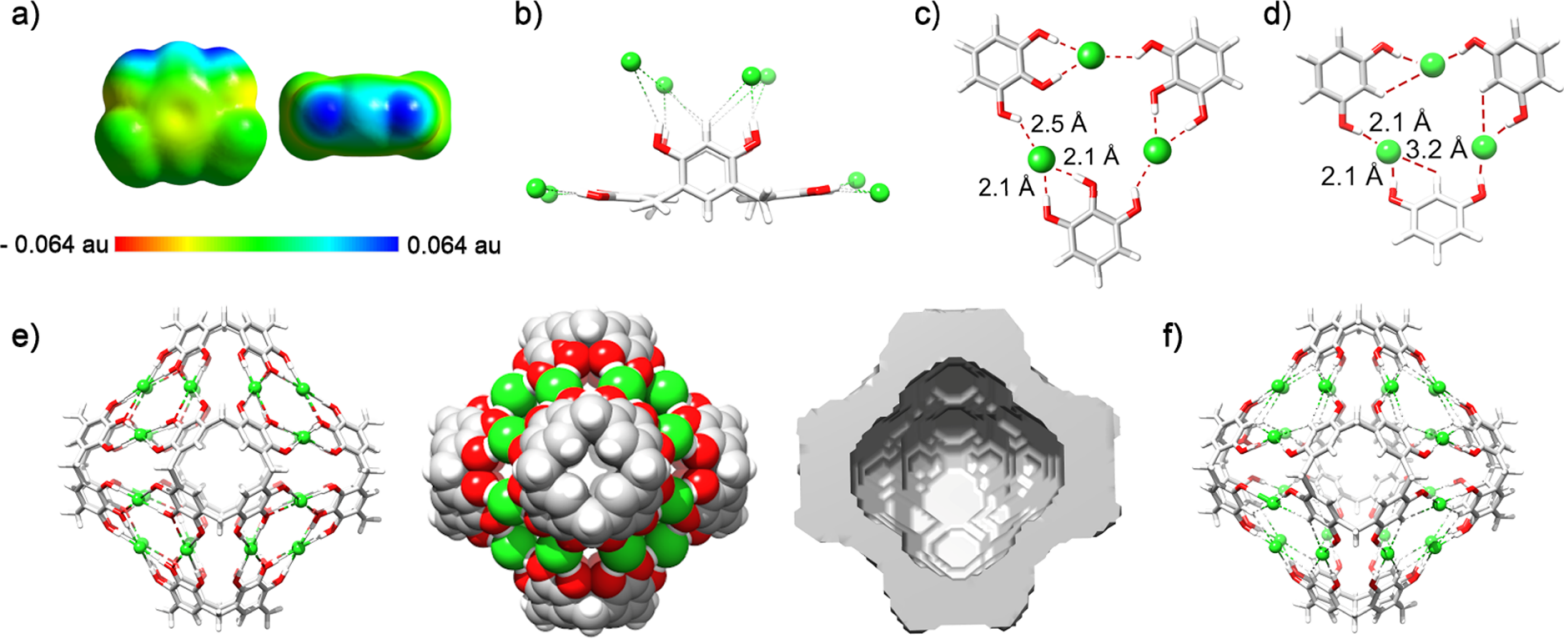
Rationale and the suggested models of anion-based closed-shell capsules: (a) ESP at the van der Waals surface of resorcinol (DFT B3LYP/6-31g), (b) X-ray structure of an anion-surrounded resorcinarene molecule (CCDC 195432), geometry-optimized binding motifs (DFT B3LYP/def2ZVPP, in THF, PCM solvent model) for (c) pyrogallol and (d) resorcinol, (e) suggested structure of hexamer (**P4H**)_6_(Cl^–^)_24_ with the binding motif, external shape, and internal cavity, and (f) suggested structure of hexamer (**R4H**)_6_(Cl^–^)_24_ with the binding motif.

To suggest a plausible model of interactions between the macrocycles and anions, an analysis of the crystallographic database (CCDC) and a series of DFT calculations were performed (Figure 4a–d).^31^ A plot of electrostatic potential (ESP) of the DFT-optimized pyrogallol and resorcinol structures indicates the presence of large clusters of positive ESP along OH-decorated rims, which are responsible for interactions with negatively charged species. Notably, the positive ESP is also present for CH_central_ in resorcinol (Figure 4a). In line with these findings, crystal structures retrieved from CCDC demonstrate that resorcin[4]arenes or pyrogallol[4]arenes co-crystallized with Alk_4_NX are surrounded by anions positioned close to their upper rims (various modes, see Figure 4b). However, these CCDC crystal structures were obtained by crystallization from competitive solvents (alcohols) and represent non-discrete structures. Using this structural information, experimental *D* values, and estimated 1:4 stoichiometry, two hypothetical anion-based discrete structures were constructed: tetramer, (M)_4_(X^–^)_16_ (Figure S115) and hexamer (M)_6_(X^–^)_24_ (Figure 4e,f). The tetramer was excluded due to its small size, exposed charges, and electrostatic repulsions. The hexamer with theoretical *d*_H_ = 23 ÷ 25 Å (the model neglects counterions and a solvation sphere) corresponds quite well to experimental *d*_H_ = 23 Å. The internal volume of the hexamer is 1830 Å^3^, which is 40% larger than the internal volume of hydrogen-bonded (**P**_**4**_**C**)_6_ (1310 Å^3^).The hexamer is based on a C_4_-crown conformation of **P4H**, and the binding motif involves the formation of trimeric clusters with anions separated/bridged by OH groups. This binding motif was inspired by the geometry of coordination hexamers,^23^ the geometry of anion–water clusters retrieved from CCDC, and it is analogous to the one that has been suggested for [5]arenes.^14^ The geometry of the binding motif was optimized using DFT in a vacuum and in THF (continuous solvation model, Figure 4c,d). In a vacuum, geometry optimization of the motif leads to its disintegration due to repulsion between chlorides. On the contrary, in THF, the optimized structure remains hydrogen-bonded with each chloride held by three hydrogen bonds with typical distances, Cl···O(H), in the range of 3.1 ÷ 3.3 Å. The Cl···Cl distances, which are expected to be repulsive, are 6.2 Å, which are longer than the shortest distances observed for such interactions in the solid state (e.g., in dinuclear oligourea/pyrrole foldamers, Cl···Cl = 3.6 ÷ 4.6 Å)^24^ and typical for H-separated interchloride distances.

Although the final structure of anion-based species remains uncertain, we think that the model of the hexamer corresponds reasonably well to the experimental data; however, non-symmetrical structures being in dynamic equilibrium are also possible.

### Interactions with Anions in Benzene

**Figure 5:**
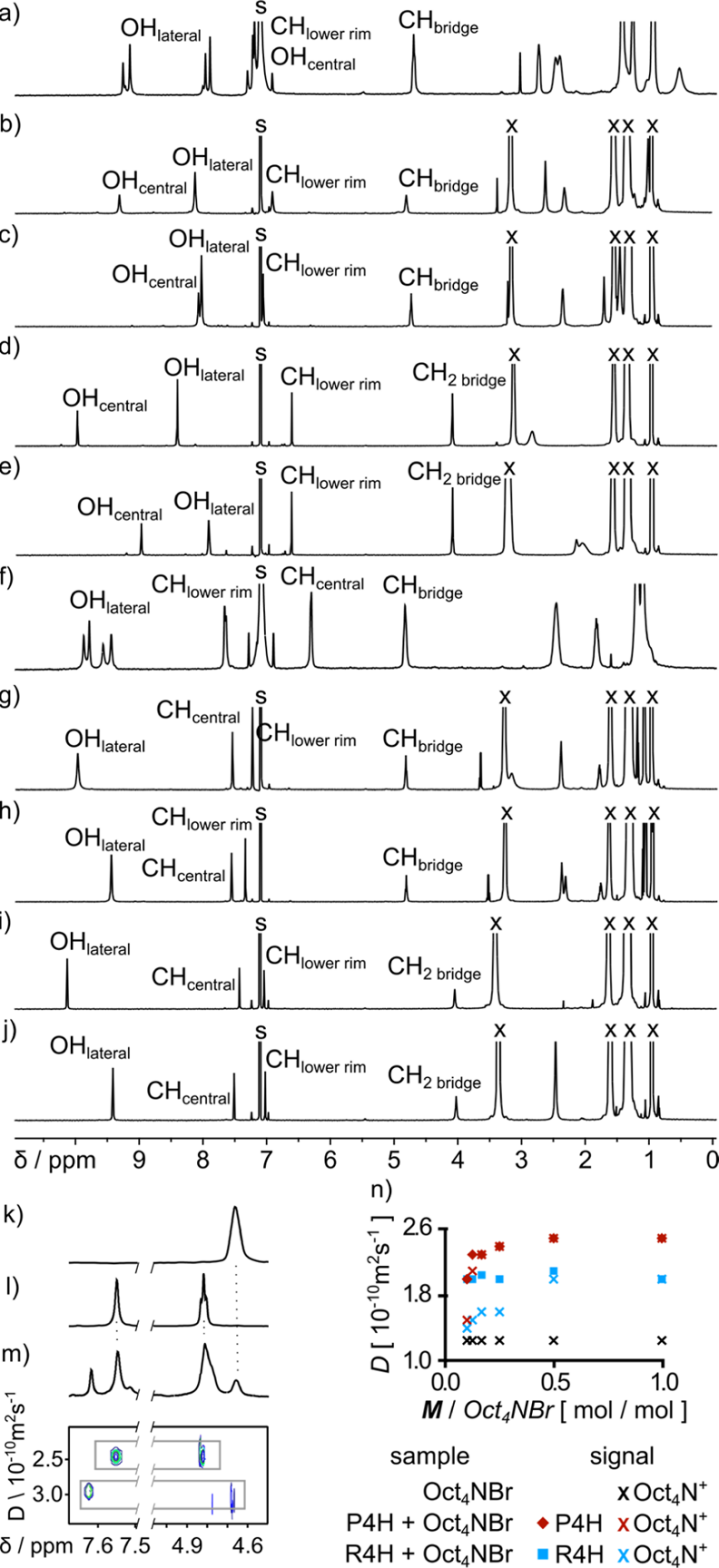
Interactions of macrocycles **M** with Alk_4_NX in benzene. ^1^H NMR spectra for (a) (**P4C**)_6_, (b) **P4C** + Oct_4_NCl, (c) **P4C** + Oct_4_NBr, (d) **P4H** + Oct_4_NCl, (e) **P4H** + Oct_4_NBr, (f) (**R4C**)_6_(H_2_O)_8_, (g) **R4C** + Oct_4_NCl, (h) **R4C** + Oct_4_NBr, (i) **R4H** + Oct_4_NCl, and (j) **R4H** + Oct_4_NBr (x—Oct_4_N^+^ signals and s—solvent). Partial ^1^H NMR spectra for (k) (**P4C**)_6_ and (l) (**P4C**)_6_Br_24_^–^, (m) partial ^1^H NMR and DOSY spectrum of (**P4C**)_6_ + (**P4C**)_6_Br_24_^–^, and (n) changes of diffusion coefficients (*D*) upon variation of the concentration of the macrocycle (*C*(Alk_4_NX) 40 mM, *C*(**M**) 4–40 mM). All samples were prepared mechanochemically and dissolved in C_6_D_6_ (see the experimental part for the procedures, 600 MHz, 298 K).

Expecting that in less polar environments, the anion-sealed capsules may be more stable (thermodynamically and kinetically), we undertook attempts to obtain anion-sealed capsules in benzene. Without Alk_4_NX being added, only **P4C** and **R4C** have detectable solubility in benzene, exhibiting patterns characteristic for (**P4C**)_6_ or (**R4C**)_6_(H_2_O)_8_ (Figure 5a,f). With positive experience in the application of mechanochemical ball milling as a method to initialize interaction between the components,^25−28^ we used this method to pre-treat the samples.

Macrocycles and the respective salts (1–8 equiv) were dry-milled in a planetary ball mill, and the solids were treated with benzene-*d*_6_. Resorcinarenes (**R4C** or **R4H**) with 1 or 2 equiv of Oct_4_NX remained insoluble. However, the addition of 4–8 equiv of Oct_4_NX leads to a substantial increase in solubility of **R4C** and **R4H**. Solubility in benzene, especially of the previously insoluble macrocycles, is a strong indication of the formation of closed-shell structures, which engages polar hydroxyl groups and saturates “solvation spheres” of anions. The signals in the ^1^H NMR spectra are sharp, and the chemical shifts are anion-dependent but remain insensitive to the cations’ size and the concentration (Figures 5 and S85–S98). The fact that at least 4 equiv of the salt are needed for good solubility supports (M)_6_(X^–^)_24_ stoichiometry. ^1^H NMR signals of OH_lateral_ for resorcinarene-based capsules appear at δ ≈ 10 ppm for chlorides and at δ ≈ 9.5 ppm for bromides, reflecting a typical trend and hydrogen bond accepting ability of anions. Particularly notable are the positions of signals of CH_central_ because they move from their typical position at ca δ = 6.4 ppm (observed, e.g., for (**R**_**4**_**C**)_6_(H_2_O)_8_, Figure 5f) to δ = 7.3–7.6 and exhibit higher values of δ for bromides than for chlorides (Figure 5g,h, a similar trend is also observed during titrations in THF, Figure 3). These downfield shifts indicate that the interactions with anions for resorcinarenes involve not only hydroxyl groups but also CH···anion interactions, and these contributions seem to be particularly relevant for interactions with large bromide anions, which is in line with the suggested binding motif.

Pyrogallolarenes (**P4H** and **P4C**) also form anion-sealed capsules in benzene-*d*_6_. **P4H**, initially insoluble in benzene-*d*_6_, becomes soluble (partially or fully) upon the addition of Alk_4_NX and mechanochemical pre-treatment. The ratio of **P4H**:Alk_4_NX in the dissolved part of the sample is at least 1:4, and the chemical shifts remain independent of the various concentrations of the Alk_4_NX. The downfield shifts of hydroxyl group signals are consistent with the involvement of all OH groups in hydrogen bonding interactions with anions, with chlorides inducing higher downfield shifts than bromides. **P4C** behaves differently because the **P4C**:Alk_4_NX ratio in the solution roughly follows the ratio in the solid samples (starts from 1:0.8, not like in previous cases from 1:4). The signals in the ^1^H NMR spectrum show concentration-dependent chemical shifts, and the final values of Δδ are much lower than for complexes of other macrocycles. This indicates that interactions of **P4C** with anions are weaker than those of other macrocycles despite its higher solubility in benzene. Thanks to a stepwise transformation from (**P4C**)_6_ to (**P4C**)_6_(Br^–^)_24_, we were able to detect the simultaneous presence of two capsular forms in one sample, and DOSY measurements confirm that the anion-sealed species are larger than neutral hydrogen-bonded capsules (Figure 5k–m).

The crucial role of anions in the self-assembly process was further supported by experiments with salts containing other, non-interacting anions. After mechanochemical pre-treatment of the macrocycles mixed with But_4_NF, But_4_NPF_6_, But_4_NH_2_PO_4_, But_4_NHSO_3_, But_4_NNO_3_, or Pen_4_NI (4 equiv), we found no traces of dissolution of the resulting samples in benzene.

### Role of Cations

Although self-assembly is predominantly anion-dependent, cationic species are inherently present as counterions. Alk_4_N^+^ can reside either inside or outside the cavity, and due to the dynamic nature of the capsules, they can be in a fast exchange between these positions and various forms of uncompleted species (free or non-specifically aggregated).

**Figure 6:**
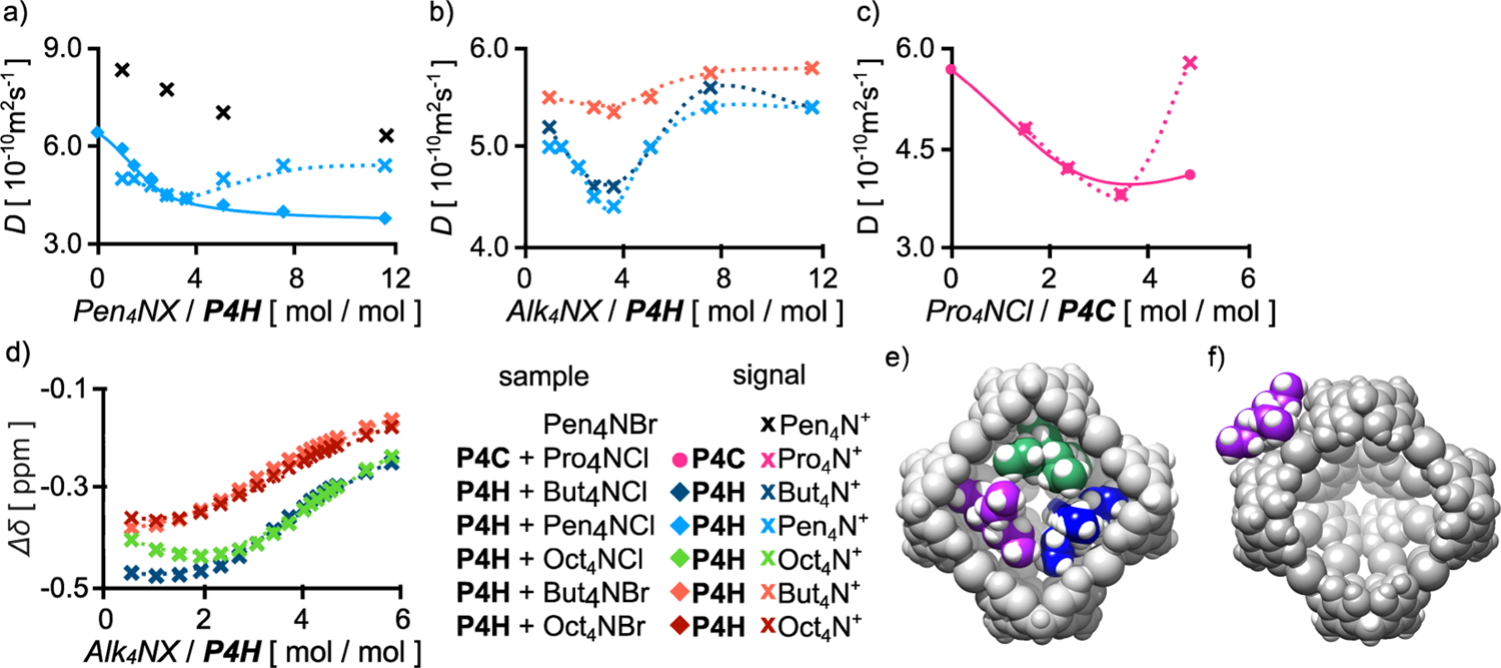
Interactions of capsules with cations in [D_8_]THF: (a,b) changes in diffusion coefficients (*D*) during the titration of **P4H** with Alk_4_NX and a comparison with concentration-dependent changes of Pen_4_NBr alone (the same concentration of Alk_4_NX, 2.5 ÷ 30 mM), (c) diffusion coefficients (*D*) for the samples of **P4H** (mmol) + Pro_4_NCl (1 ÷ 5 equiv), ball-milled and dissolved in [D_8_]THF; the ratio between components was calculated by integration of the spectral (d) changes in ^1^H chemical shifts (Δδ) of −CH_2_N^+^ signals during titration of **P4H** with Alk_4_NX [all experiments at 298 K, 600 MHz, analyte: *C*(**P4H**) = 2.5 mM and titrant: *C*(**P4H**) = 2.5 mM + *C*(Alk_4_NX) = 65 mM]. A model of the anion-sealed capsule with (e) three Pro_4_N^+^ in the cavity, and (f) Bu_4_N^+^ interacting externally (the atoms in the front were partially removed to visualize the interior of the cavity).

^1^H NMR signals of Alk_4_N^+^ undergo upfield shifts upon addition of macrocycles (both in THF-*d*_8_ and in benzene-*d*_6_). For all Alk_4_N^+^, the largest Δδ is observed for the methylene protons next to the nitrogen (−CH_2_–N^+^, Δδ_max_ ≈ −0.48 ppm, Figure 6d), while other signals experience considerably smaller shifts (Δδ_max_ < −0.17 ppm, Figure S118a). Analysis of Δδ for various Alk_4_NX indicates that the effect of cation size (But_4_NCl vs Oct_4_NCl) is smaller than the effect of the anion type (Alk_4_NCl vs Alk_4_NBr) and chlorides impose larger Δδ values than bromides (Figure 6d). These properties are interpreted in terms of ion pairing of Alk_4_N^+^ with anion-based capsules (either inside or outside the cavity, Figure 6e,f), which is stronger for capsules containing chlorides than bromides. An upfield shift of −CH_2_–N^+^ signals can be explained by electrostatic attractions within an ion pair that change the conformation of Alk_4_N^+^ so that the cationic core is exposed and placed in the proximity of the aromatic walls of the capsules (Figures 6f and S118c).

Ion pairing was also confirmed by DOSY both in THF-*d*_8_ (Figure 6a,b) and benzene-*d*_6_ (Figure 5n). In THF-*d*_8_, in the absence of the macrocycles, *D*(Alk_4_N^+^) values decrease monotonically with increasing concentration of Alk_4_NX, in line with concentration-dependent non-specific aggregation (Figures 6a and S101d). On the contrary, in the presence of the macrocycles, the profiles of changes are substantially different. Upon addition of Alk_4_NX to **M** (constant concentration) in THF-*d*_8_, the titration curve with respect to *D*(Alk_4_N^+^) is non-monotonic (Figure 6a,b). It is interpreted assuming that Alk_4_N^+^ forms an ion pair(s) with a large and highly negatively charged capsule, which leads to a low *D*(Alk_4_N^+^) value when the relative concentration of the capsules is high (initial points) and to an increase in the *D*(Alk_4_N^+^) value as the amount of Alk_4_NX increases. In line with this interpretation, *D*(**M**) values remain at the same level in this experiment and weaker ion pairing with capsules containing Br^–^ leads to less pronounced effects (Figure 6b). In benzene-*d*_6_, non-specific Alk_4_NX aggregation is very strong (Figure S116) and the macrocycles have limited solubility. Therefore, the reversed titrations were performed at constant Alk_4_NX concentration (Figure 5n). In such a case, *D*(Alk_4_N^+^) values are expected to stay constant in absence of specific interactions. However, upon the addition of the macrocycles, *D*(Alk_4_N^+^) values systematically increase—up to the values of the postulated anion-sealed capsules. These observations are in line with the hypothesis that cations form ion pairs with large anion-sealed capsules.

To check the possibility of encapsulation of smaller cations, Met_4_NX, Et_4_NX, and Pro_4_NX were also tested. All these salts are soluble neither in THF nor in benzene. Therefore, the samples containing macrocycles and the respective salts (at a ratio of 1:1 up to 1:8) were ball-milled and subsequently treated with benzene or THF. In benzene, all samples remained insoluble. Quite surprisingly, most of them were also insoluble in THF, even though the macrocycles in their “free” forms were readily soluble in THF. Only **P4C**/Pro_4_NBr and **P4C**/Pro_4_NCl were partially dissolved in THF-*d*_8_ but revealed distinct behaviors. For **P4C**/Pro_4_NBr, the ratio between dissolved components remains constant, 1:1, irrespective of the initial ratio in the solid sample (Figure S110). Importantly, *D*(Pro_4_N^+^) values indicate the formation of small species, most likely by the complexation of cations in the monomeric cavitand. On the contrary, for **P4C**/Pro_4_NCl, the ratio between dissolved components is variable—from 1:1 to the maximum limiting value of 1:4 (Figure S109). In **P4C**/Pro_4_NCl, the *D* values for the macrocycle and the cation are ratio-dependent and much lower than in **P4C**/Pro_4_NBr. Particularly striking is the abrupt differentiation of *D* values between **P4C** signals and Pro_4_N^+^ signals at the 1:4 ratio (Figure 6c). These data confirm that (1) four Cl^–^ per macrocycle are needed for capsule formation and (2) tight ion pairing involves less than four Pro_4_N^+^ per macrocycle; therefore, at the **P4C**/Pro_4_NCl 1:4 ratio, there is an exchange between “bound” and “free” cations, leading to a higher *D*(Pro_4_N^+^) value. Although it is uncertain if the cations reside inside or outside the cavity (or both), our models indicate that three Pro_4_N^+^ cations can fit in the cavity (occupancy 32%), but placing four cations leads to steric hindrance (although possible from the point of view of occupancy). For comparison, the hexameric capsule (**R4C**)_6_(H_2_O)_8_ can accommodate two Eth_4_N^+^ cations as reported by Cohen.^4f^

Low-temperature experiments for **P4C**/Pro_4_NCl and **P4C**/Pen_4_NCl show that within the temperature range 298 ÷ 233 K, the complexes remain dynamic (Figures S119–S122).

### Interactions with Anions in Chloroform

C-alkyl-substituted macrocycles **P4C** and **R4C** are known to spontaneously and quantitatively form self-assembled capsules (**R4C**)_6_(H_2_O)_8_ and (**P4C**)_6_ in chloroform and encapsulate suitably sized Alk_4_N^+^ cations (up to Oct_4_N^+^).^4a^ It has been reported that Alk_4_NX salts have to be added in small amounts for encapsulation because, otherwise, they induce disassembly.^4a^ Here, we evaluate the ability of anions to become components of self-assembled species, and therefore, we used higher amounts of Alk_4_NX and systematically screened various [4]arenes by DOSY.

**Figure 7:**
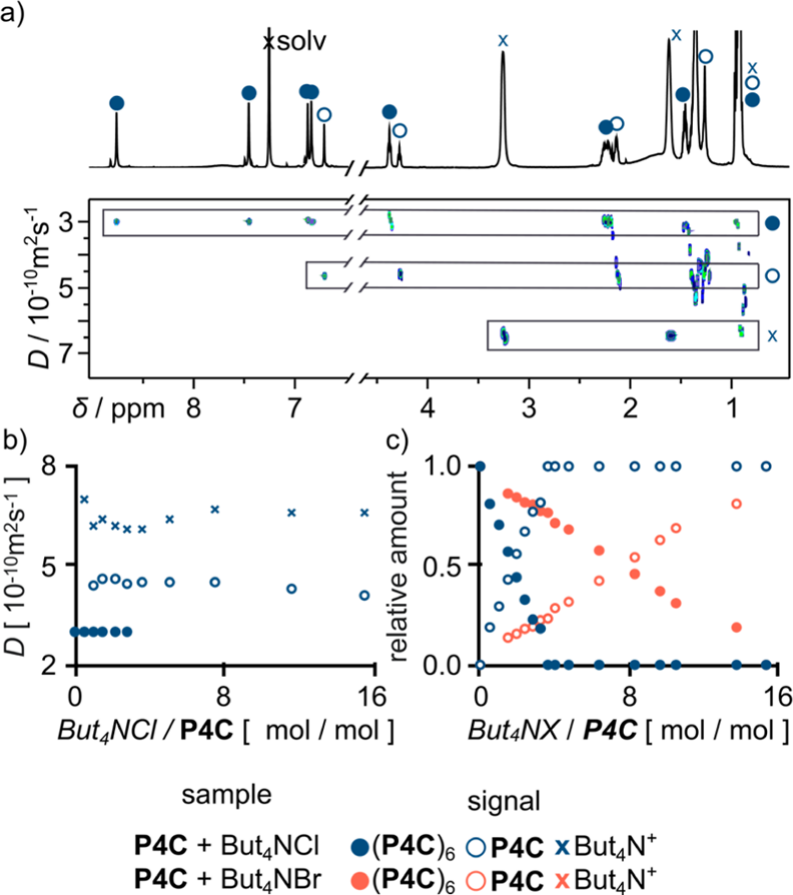
Interactions of macrocycles with Alk_4_NX in chloroform: (a) ^1^H NMR and DOSY spectra of mixture **P4C** (2.5 mM) + **Oct**_**4**_**NCl** (65 mM), (b) diffusion coefficients (*D*) for all species during titration of **P4C** with **Oct**_**4**_**NCl**, and (c) profiles of the anion-induced disassembly (all in CDCl_3_, 600 MHz, 298 K).

The spectra of **P4C** in chloroform indicate the formation of hexamers (**P4C**)_6_, in line with the previous findings. The addition of But_4_NCl leads to the gradual disintegration of the hexamers to monomers, and ^1^H NMR spectra show two separate sets of signals having different diffusion coefficients, *D* (Figure 7a). *D* values and chemical shifts for both species remain invariable during the titration (Figure 7b). At 4 equiv of But_4_NCl being added, initially formed capsules (**P4C**)_6_ are completely disintegrated. Disassembly requires higher amounts of But_4_NBr than But_4_NCl (Figure 7c), indicating that disassembly is anion-mediated and reflecting a higher hydrogen bonding affinity of chlorides than bromides.

These data demonstrate that in chloroform, interactions between macrocycles and anions are distinctly different than in other solvents: addition of Alk_4_NX leads to the disintegration of neutral hydrogen-bonded hexamers and the anion-based capsules are not re-assembled.

### Stability of Complexes

Due to the complex character of equilibria, various solubility values, and possible cooperative effects, we were not able to determine absolute association constants. However, the relative order of stability can be estimated based on a general rule that, for the same stoichiometry and identical initial concentrations, a stronger interaction gives a stronger curvature of a titration curve.^29^ Thus, the data were normalized to the 0–1 range (Δδ or *D*), and data were analyzed to determine the relative strength of interactions (Figure S103). The plots suggest that for a given macrocycle, the complexes with chlorides are always stronger than those with bromides, which reflects the order of the hydrogen bonding ability of anions. However, the preference toward chlorides is less pronounced for resorcinarenes than for pyrogallolarenes. This may reflect the particular role of CH···anion interactions for bromides and a good fit between this large anion and a small central atom (here hydrogen, see binding motif). Among the chloride complexes with various macrocycles, the order of stability is as follows: **P4H** > **R4H** ≈ **R4C** > **P4C**. Thus, lower-rim crowded macrocycles are less prone to form complexes, which suggests that some conformational flexibility is required for optimal interactions.

The stability of **P4H**/Pen_4_NCl toward the addition of polar solvents was tested by adding water or methanol (0 ÷ 5% vol/vol) to its solution in THF-*d*_8_. The addition of polar solvents leads to an increase in *D* values of the components, indicating gradual disassembly (Figure S117). It should be noted that water exerts weaker effects than methanol, when added in the same amounts. The relatively low sensitivity to traces of water in THF (<2%) is also supported by the high reproducibility of the results from experiments performed using different batches of solvent (see Figure S123).

### Role of the Solvent

**Figure 8:**
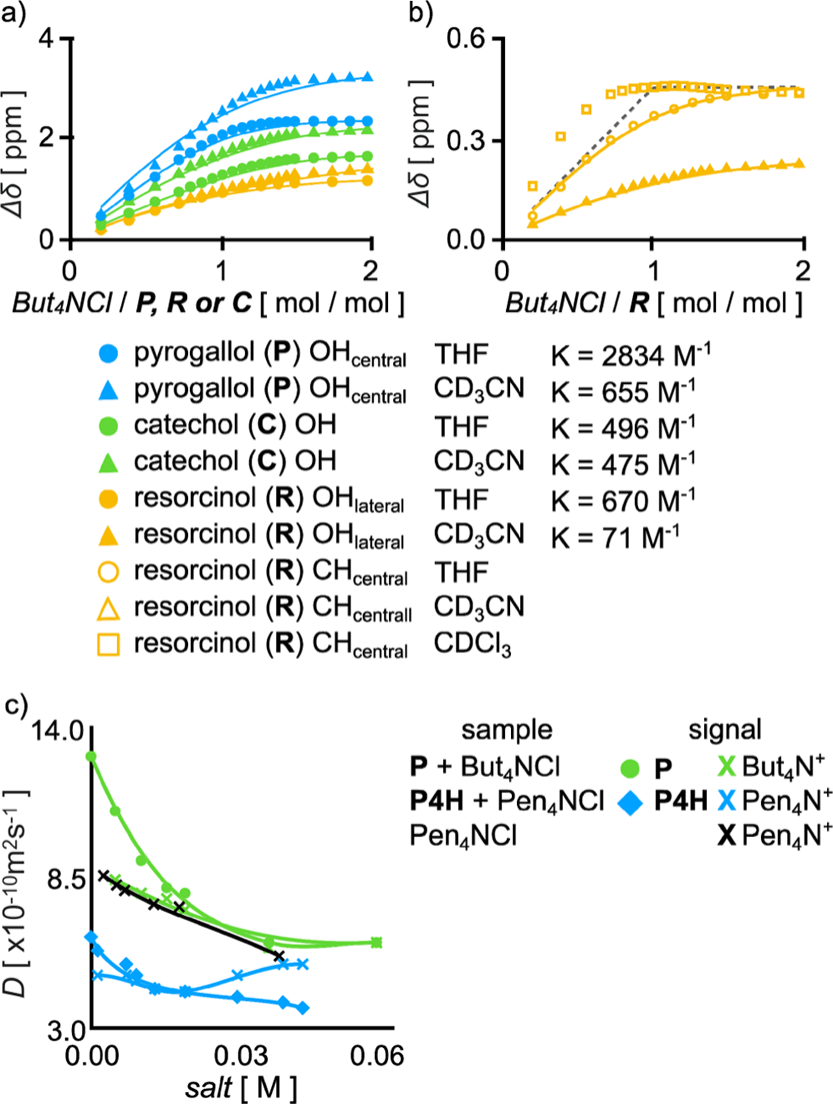
^1^H NMR titrations of pyrogallol (**P**), catechol (**C**), and resorcinol (**R**) with But_4_NCl in CDCl_3_, THF-*d*_8_, and MeCN-*d*_3_: (a) signals of OH for **P** and **C** and (b) signals of CH_central_ for **R**. Solid lines represent fitted curves and dashed lines represent theoretical curves for the 1:1 model (all experiments at 298 K and 400 MHz). (c) Diffusion coefficients (*D*) for all species during titrations of **P** (10 mM) and **P4H** (2.5 mM) with Alk_4_NCl.

The question about the role of the solvent, especially the non-intuitive formation of the complexes in THF but not in less polar chloroform, was posed and theoretically discussed in the previous paper.^14^ Here, we evaluated the influence of a solvent on anion binding by using reference non-macrocyclic polyphenols—resorcinol (**R**), pyrogallol (**P**), and catechol (**C**). These polyphenols were titrated with But_4_NX in CDCl_3_, THF-*d*_8_, and CD_3_CN (Figure 8).

In THF and CD_3_CN (used for comparison with the literature),^17^ the titration results were fitted using OH signals with 1:1 models (Figure 8a). The association constants (*K*) are considerably higher in THF (reflecting its lower ε) than in CD_3_CN, and the order of stability is **P** > **R** > **C**. The formation of complexes in THF was also detected by DOSY (Figures 8c and S125). For example, upon the addition of Alk_4_NCl salt, the values of *D*(**P**) decrease to values similar to those of *D*(Alk_4_NCl), reflecting the formation of complexes that have a size similar to the size of the salt itself but smaller than in the case of macrocyclic ligands (Figure 8c). These control experiments explain why macrocyclic compounds, composed of similar phenolic building blocks, also interact with anions in THF.

Determination of stability of anion complexes of **P**, **R,** and **C** in chloroform is difficult due to disappearance of OH signals. Therefore, the comparison between solvents was made only for **R** using its CH_central_ signal (Figure 8b). The shape of the titration curve in CDCl_3_ indicates that interactions between Cl^–^ and **R** are stronger and more complex in CDCl_3_ than in THF-*d*_8_ and CD_3_CN and reflects the coexistence of 2:1, 1:1, and 1:2 complexes (the first maximum is reached before 1 equiv and distinct changes are visible after reaching 2 equiv of the salt). Thus, lack of formation of anion-sealed capsules by macrocyclic compounds in chloroform cannot be attributed solely to weaker interactions between phenolic building blocks and anions in this solvent. Other factors (solvation of the macrocycles, entropic factors, and ion pair formation) have to be further analyzed.

## Conclusions

The current findings shed new light on the possible modes of interactions between pyrogallol[4]arenes and resorcin[4]arenes with tetralakylammonium salts, pointing out the crucial role of hydrogen bonds with anions. In weakly anion-solvating environments (THF and benzene), anion···OH/CH interactions lead to the formation of large self-assembled capsular species. In sharp contrast, in chloroform, the analogous interactions lead to the destruction of initially formed hexamers and anions do not participate in self-assembly. The newly formed anion-sealed capsules bear a high-density negative charge and form tight but still dynamic ion pairs with cations. These properties are expected to generate unique recognition properties and possibly also catalytic properties. We also think that the ability of resorcinarenes to interact with anionic species may help to understand widely discussed Brønsted acidity of resorcinarene capsules,^6a^ unusual selectivity in C–X bond activation,^7a^ anion-dependent encapsulations in (**R4C**)_6_(H_2_O)_8_ reported by Rebek^4a^ or Horiuchi,^30^ and the anion-dependent extrusion of water molecules from (**R4C**)_6_(H_2_O)_8_ mentioned by Cohen.^4d,4f^

Importantly, the current results indicate that the anion-based self-assembly mode is general—it was detected for polyphenolic macrocycles of various ring sizes ([4]arenes and [5]arenes) having different substitution patterns (pyrogallol and resorcinol derivatives) and various levels of conformational rigidity (lower-rim-substituted and unsubstituted derivatives). Thus, we envision that in the future, it can also be found for other polyphenolic macrocycles leading to the discovery of new anion-based closed-shell structures.

## Experimental Section

### General Procedure for ^1^H NMR Titrations

To the solution of a macrocycle (*C* = 0.005 M, 0.0025 mmol) in THF-*d*_8_ (0.5 mL), a solution containing Alk_4_NX (C = 0.075 M, 0.075 mmol) and the macrocycle (C = 0.005 M, 0.005 mmol) in THF-*d*_8_ (1 mL) was added in portions. ^1^H NMR spectra were recorded at 303 K using Bruker 400 MHz.

### General Procedure for DOSY Titrations

To the solution of a macrocycle (*C* = 0.0025 M, 0.00125 mmol) in THF-*d*_8_ (0.5 mL), a solution containing Alk_4_NX (*C* = 0.0656 M, 0.0656 mmol) and the macrocycle (*C* = 0.0025 M, 0.0025 mmol) in THF-*d*_8_ (1 mL) was added in portions. ^1^H NMR and DOSY spectra were recorded at 303 K using Varian 600 MHz.

### Preparation of the Samples by Mechanochemistry

Solid sample of a macrocycle (0.01 mmol) and Alk_4_NX (1 ÷ 8 equiv) were ball-milled for 1 h in a planetary ball mill. Then, the powder was dissolved in benzene-*d*_6_ (0.7 mL). The sample was filtered, and the solution was analyzed by NMR.

## Supporting Information Available

Experimental details, NMR and DOSY titration curves, and numerical values (PDF)
Atomic coordinates of a hexamer cage (**P4H**)_6_Cl_24_ (PDB)
Atomic coordinates of a hexamer cage (**R4H**)_6_Cl_24_ (PDB)

## Supplementary Material

ja1c11793_si_001.pdf

ja1c11793_si_002.pdb

ja1c11793_si_003.pdb
