# Supplementary material for: Anion-Based Self-assembly of Resorcin[4]arenes and Pyrogallol[4]arenes
Source: J Am Chem Soc. 2022 Mar 11;144(12):5350–8. doi: 10.1021/jacs.1c11793 (PMC8972256; doi:10.1021/jacs.1c11793)
Supplement: Supplementary file 1 — ja1c11793_si_001.pdf [file ja1c11793_si_001.pdf]

Anion-based self-assembly of resorcin[4]arenes and pyrogallol[4]arenes

Monika Chwastek,[a] Piotr Cmoch[a] and Agnieszka Szumna*[a]

[a] MSc. M. Chwastek, Dr. P. Cmoch, Prof. A. Szumna

Institute of Organic Chemistry

Polish Academy of Sciences

Kasprzaka 44/52, 01-224 Warsaw, Poland

E-mail: agnieszka.szumna@icho.edu.pl

Table of Contents

1. GENERAL INFORMATION 3
2. GENERAL PROCEDURE FOR ^{1}H NMR TITRATIONS 3
3. GENERAL PROCEDURE FOR DOSY TITRATIONS 3
4. PROCEDURE FOR MECHANOCHEMICAL TREATMENT OF THE SAMPLES 3
5. TITRATIONS OF PYROGALLOL[4]ARENE (P4C) IN THF 4
6. TITRATIONS OF PYROGALLOL[4]ARENE (P4H) IN THF 17
7. TITRATIONS OF RESORCIN[4]ARENE (R4C) IN THF 30
8. TITRATIONS OF RESORCIN[4]ARENE (R4H) IN THF 41
9. ^{1}H NMR SPECTRA FOR MECHANOCHEMICALLY TREATED SAMPLES 51
10. TITRATIONS IN CHLOROFORM (P4C) 71
11. DATA COMPARISON 72
12. TITRATIONS OF PYROGALLOL 77
13. TITRATIONS OF RESORCINOL 79
14. TITRATIONS OF PYROCATECHOL 81
15. INTERACTIONS WITH SMALLER SALTS (P4C) 83
16. CONTROL EXPERIMENTS 85
17. MODEL OF TETRAMER $(M)_4(X^-)_{16}$ 87
18. NON-SPECIFIC ALK_4NX AGGREGATION IN BENZENE AND THF 87
19. INFLUENCE OF METHANOL AND WATER ON THE STABILITY OF THE CAPSULE. 88
20. COMPARISON OF CHANGES OF CHEMICAL SHIFT SOF ALK_4N+ SIGNALS 89
21. VARIABLE TEMPERATURE ^{1}H NMR SPECTRA 90
22. REPEATABILITY 92
23. DOSY TITRATIONS OF RESORCINOL IN THF 93
24. DOSY TITRATIONS OF PYROGALLOL IN THF 94
25. DOSY TITRATIONS OF CATECHOL IN THF 95
26. COMPARISON OF THE SIZE OF P4H AND P5H CAPSULES 96
27. DOSY MEASUREMENT AND CALCULATION OF THE SIZES 97
28. AB INITIO CALCULATIONS 97

1. General information

All solvents and chemicals used were purchased from Sigma Aldrich, TCI Europe N. V., Roth and Euriso-top, were of reagent grade and were used without further purification.

^{1}H NMR spectra were recorded at 303 K on Bruker 400 MHz and Varian 600 MHz instruments with residual solvent signal as internal standard.

Macrocycles were synthesized using literature procedures R4H[i], R4H[ii], P4C[iii], P4H[iv].

2. General procedure for ^{1}H NMR titrations

To the solution of a macrocycle (C = 0.005 M, 0.0025 mmol) in THF-d_8 (0.5 ml) the solution containing Alk_4NX (C = 0.075 M, 0.075 mmol) and the macrocycle (C = 0.005 M, 0.005 mmol) in THF-d_8 (1 ml) was added. ^{1}H NMR spectra were recorded at 303 K using Bruker 400 MHz.

3. General procedure for DOSY titrations

To the solution of a macrocycle (C = 0.0025 M, 0.00125 mmol) in THF-d_8 (0.5 ml) the solution containing Alk_4NX (C = 0.0656 M, 0.0656 mmol) and the macrocycle (C = 0.0025 M, 0.0025 mmol) in THF-d_8 (1 ml) was added. ^{1}H NMR spectra and DOSY measurement were recorded at 303 K using Bruker 600 MHz.

4. Procedure for mechanochemical treatment of the samples

Solid sample of macrocycle (0.01 mmol) and Alk_4NX (various rations from 1 eq to 8 eq) were ball-milled (1h dry-milling in a planetary ball-mill) and the powders were dissolved in benzene-d_6 (0.7 ml). The sample was filtered, and the solution was checked by ^{1}H NMR.

5. Titrations of Pyrogallol[4]arene (P4C) in THF

5.1 Titration of pyrogallol[4]arene (P4C) with tetrabutylammonium chloride (But_4NCl)

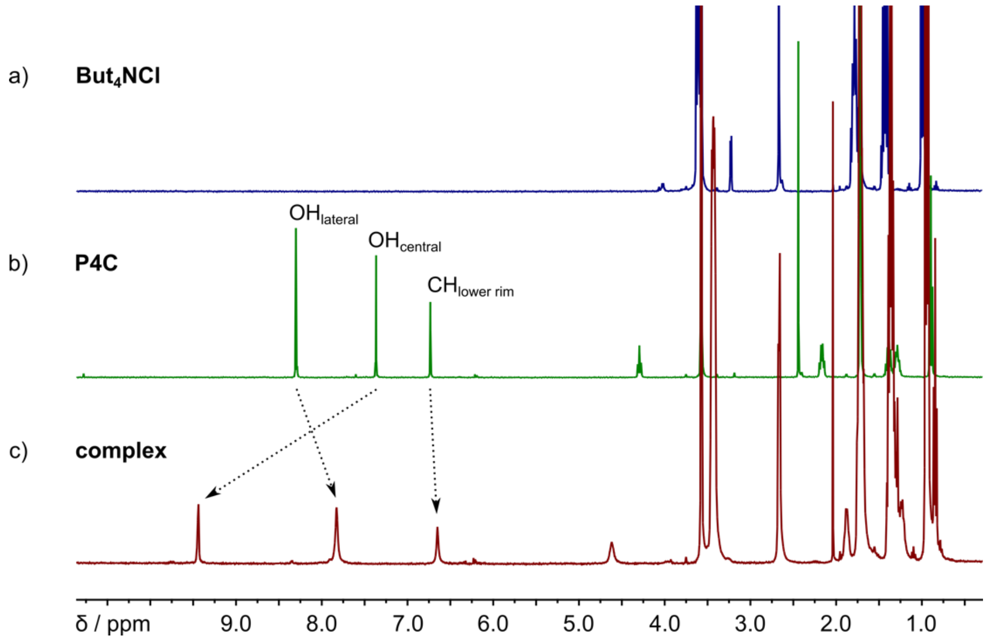

Figure S1. ^{1}H NMR spectra of (a) **But_4NCl**; (b) **P4C**; (c) complex of **P4C** and **But_4NCl** (400 MHz, 303 K, THF-d_8).

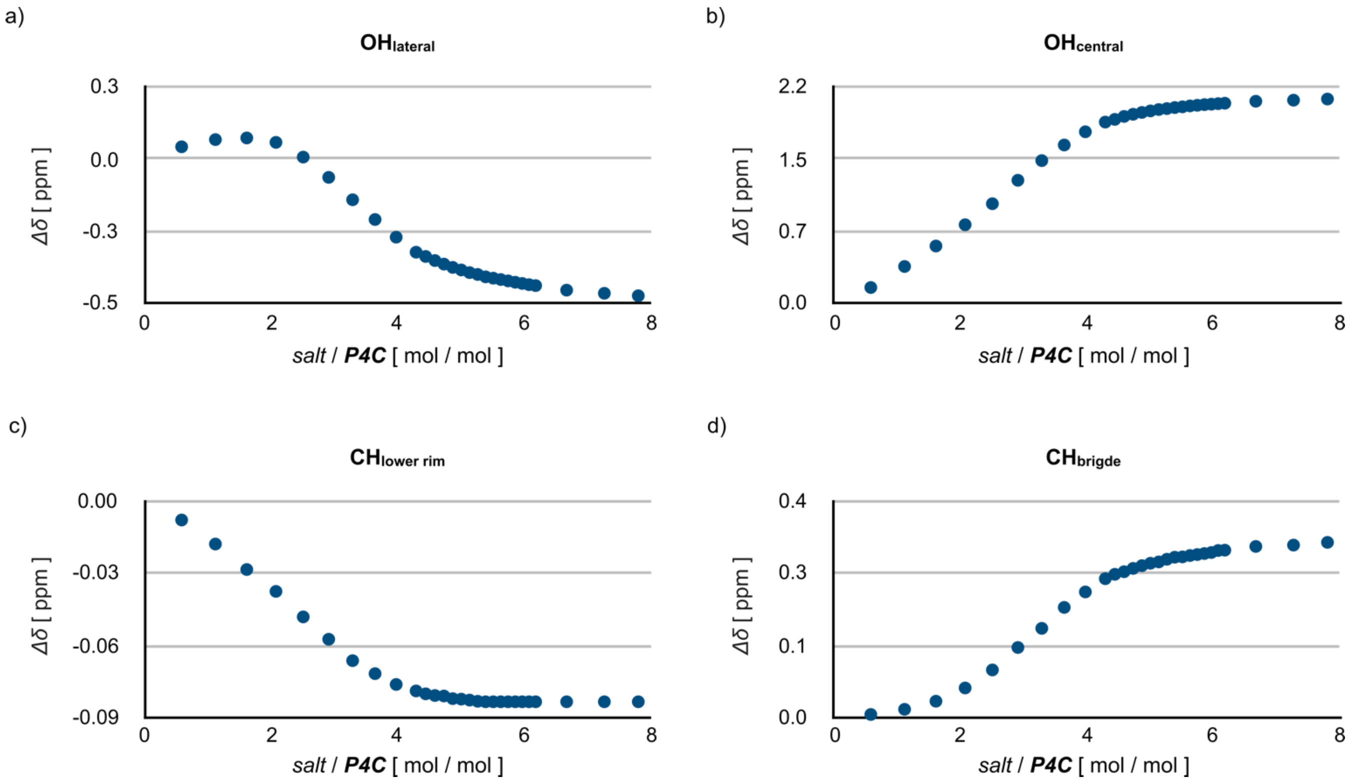

Figure S2. ^{1}H NMR titration curves for titration of **P4C** (C = 5.0 mM) with titrant **P4C** (C = 5.0 mM) + **But_4NCl** (C = 75 mM). ^{1}H NMR chemical shifts change for: (a) $OH_{lateral}$; (b) $OH_{central}$; (c) $CH_{lowr\ rim}$; (d) CH_{bridge} (400 MHz, 303 K, THF-d_8).

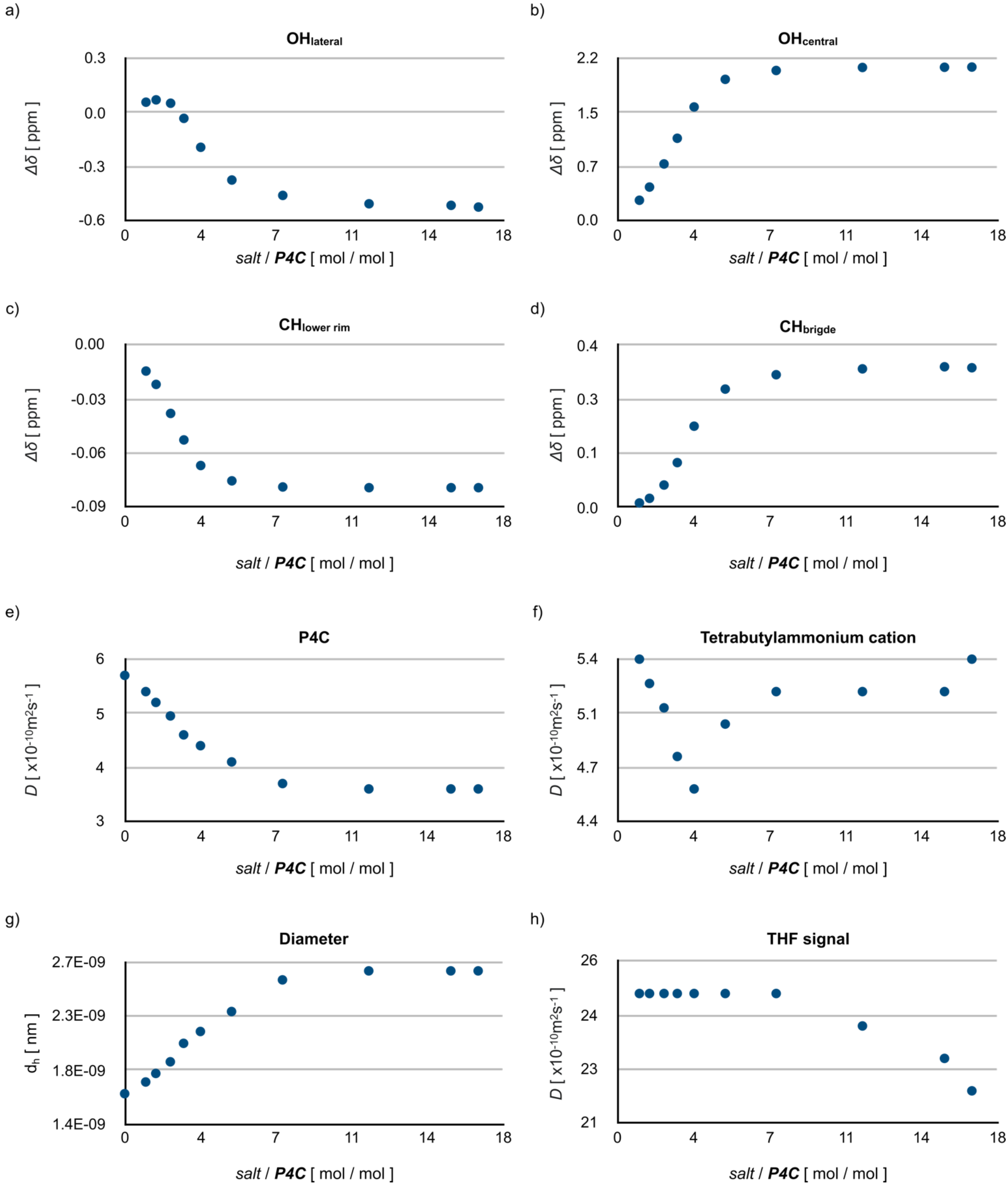

Figure S3. DOSY titration curves for titration of **P4C** (C = 2.5 mM) with titrant **P4C** (C = 2.5 mM) + **But$_4$NCl** (C = 65.6 mM). ^{1}H NMR chemical shift changes for: (a) OH$_{lateral}$; (b) OH$_{central}$; (c) CH$_{lower\ rim}$; (d) CH $_{bridge}$. Diffusion coefficient changes: for (e) **P4C;** (f) **But$_4$NCl**; (g) diameter of complex; (h) THF (600 MHz, 303 K, THF-d$_8$).

P4C	But_4N^+	Salt / M [mol / mol]
5.7		0
5.4	5.4	1
5.2	5.25	1.48
4.95	5.1	2.17
4.6	4.8	2.8
4.4	4.6	3.6
4.1	5	5.08
3.7	5.2	7.5
3.6	5.2	11.6
3.6	5.2	15.5
3.6	5.4	16.8

Table S1. Data for DOSY titration (**P4C** (C = 2.5 mM) with titrant **P4C** (C = 2.5 mM) + **But_4NCl** (C = 65.6 mM).)

5.2 Titration of pyrogallol[4]arene (P4C) with tetrapentylammonium chloride (Pen_4NCl)

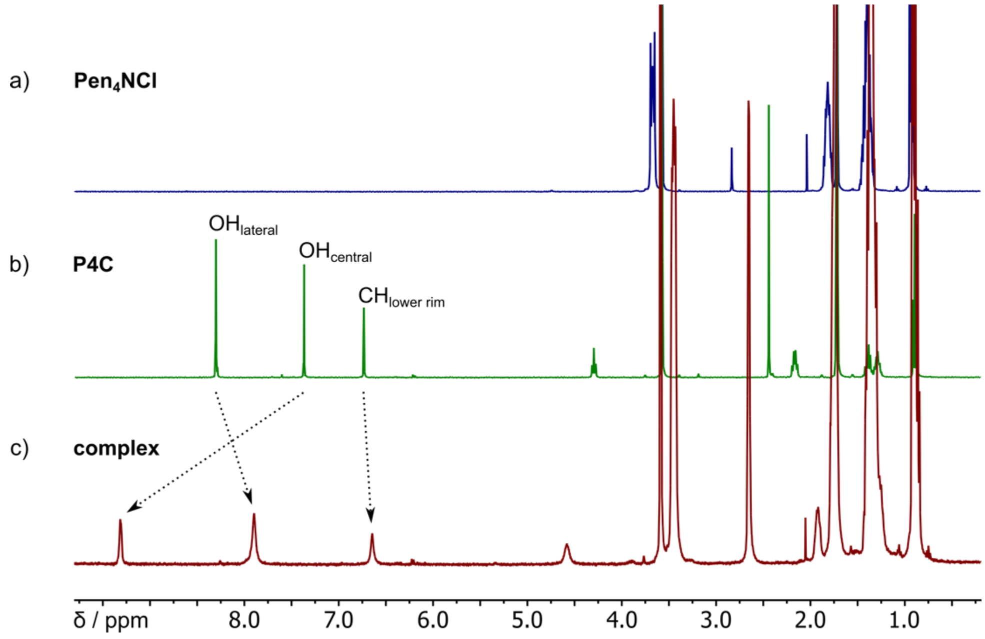

Figure S4. ^{1}H NMR spectra of (a) **TPenACl**; (b) **P4C**; (c) complex of **P4C** and **Pen_4NCl** (400 MHz, 303 K, THF-d_8).

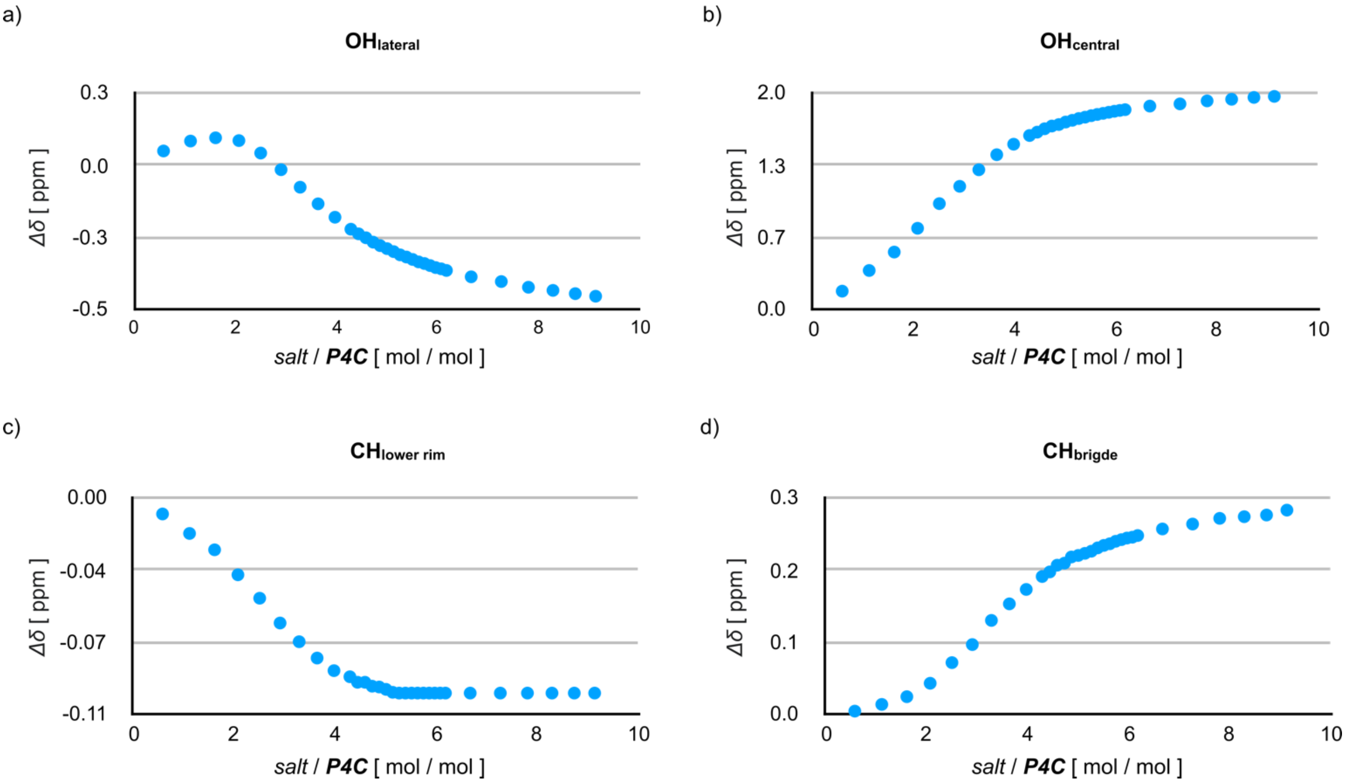

Figure S5. ^{1}H NMR titration curves for titration of **P4C** (C = 5.0 mM) with titrant **P4C** (C = 5.0 mM) + **Pen_4NCl** (C = 75 mM). ^{1}H NMR chemical shifts change for: (a) $OH_{lateral}$; (b) $OH_{central}$; (c) $CH_{lowr\ rim}$; (d) CH_{bridge} (400 MHz, 303 K, THF-d_8).

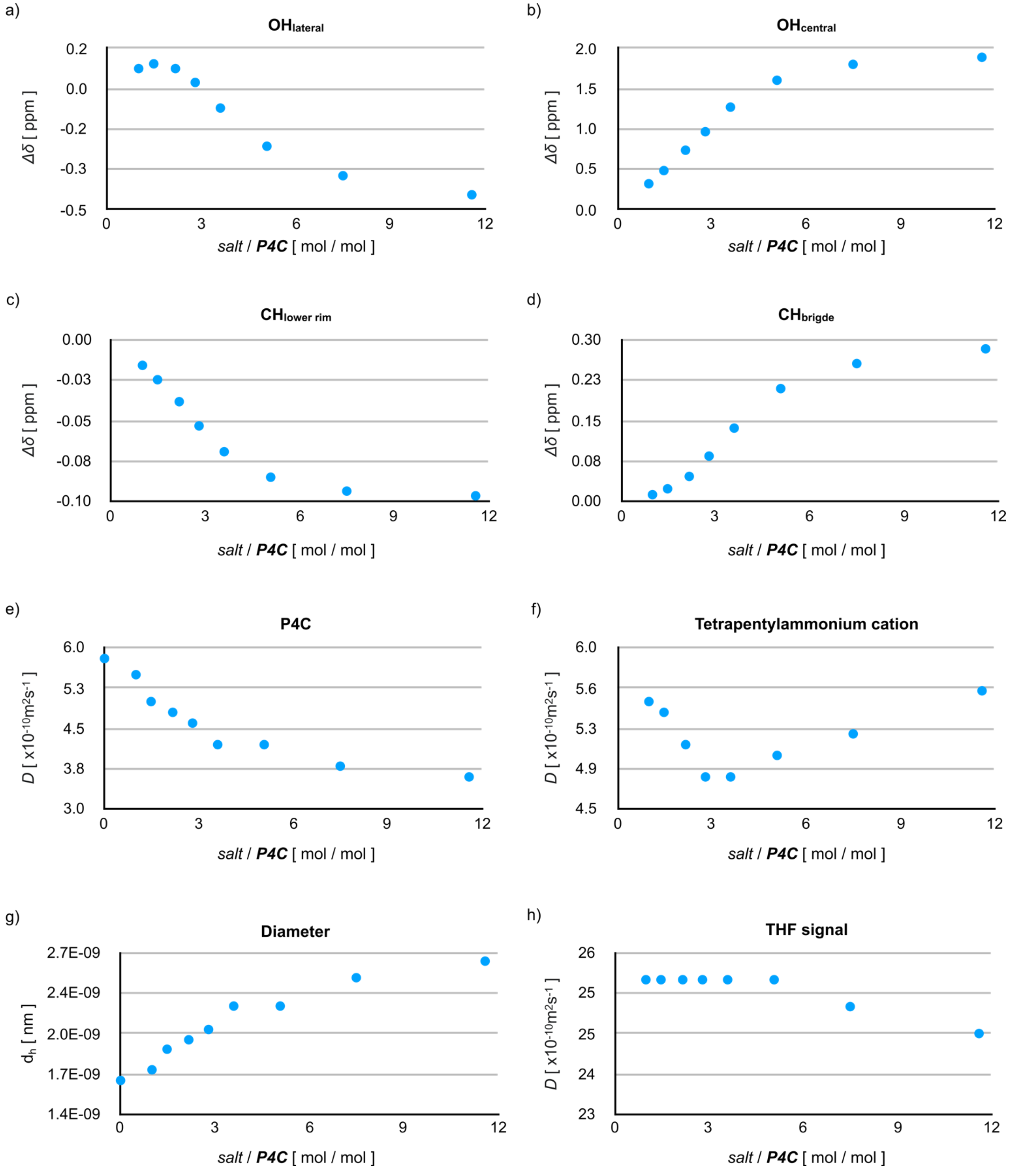

Figure S6. DOSY titration curves for titration of **P4C** (C = 2.5 mM) with titrant **P4C** (C = 2.5 mM) + **Pen$_4$NCl** (C = 65.6 mM). ^{1}H NMR chemical shift changes for: (a) $OH_{lateral}$; (b) $OH_{central}$; (c) $CH_{lower\ rim}$; (d) CH_{bridge}. Diffusion coefficient changes: for (e) P4C; (f) Pen$_4$NCl; (g) diameter of complex; (h) THF (600 MHz, 303 K, THF-d_8).

P4C	Pen_4N^+	Salt / M [mol / mol]
5.8		0
5.5	5.5	1
5	5.4	1.48
4.8	5.1	2.17
4.6	4.8	2.8
4.2	4.8	3.6
4.2	5	5.08
3.8	5.2	7.5
3.6	5.6	11.6

Table S2. Data for DOSY titration **P4C** (C = 2.5 mM) with titrant **P4C** (C = 2.5 mM) + **Pen_4NCl** (C = 65.6 mM)).

5.3 Titration of pyrogallol[4]arene (P4C) with tetraoctylammonium chloride (Oct_4NCl)

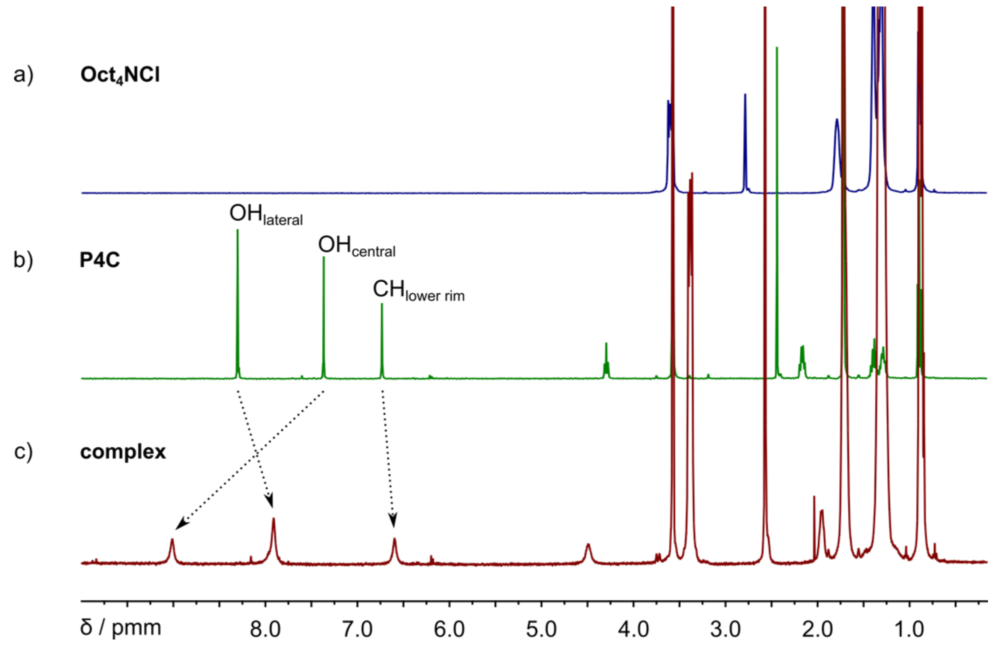

Figure S7. ^{1}H NMR spectra of (a) **TOctACl**; (b) **P4C**; (c) complex of **P4C** and **Oct_4NCl** (400 MHz, 303 K, THF-d_8).

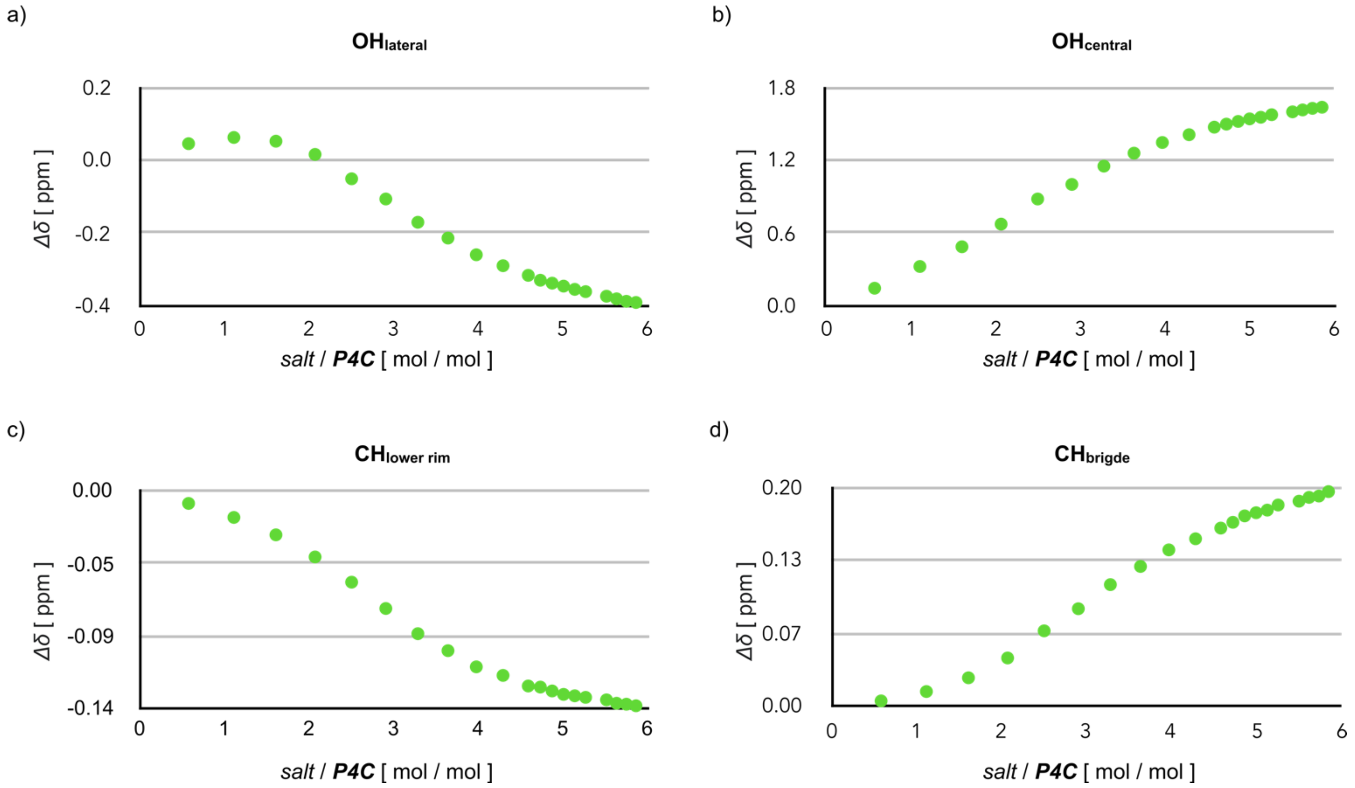

Figure S8. ^{1}H NMR titration curves for titration of **P4C** (C = 5.0 mM) with titrant **P4C** (C = 5.0 mM) + **Oct_4NCl** (C = 75 mM). ^{1}H NMR chemical shifts change for: (a) $OH_{lateral}$; (b) $OH_{central}$; (c) $CH_{lowr\ rim}$; (d) CH_{bridge} (400 MHz, 303 K, THF-d_8).

5.4 Titration of pyrogallol[4]arene (P4C) with tetrabutylammonium bromide (But_4NBr)

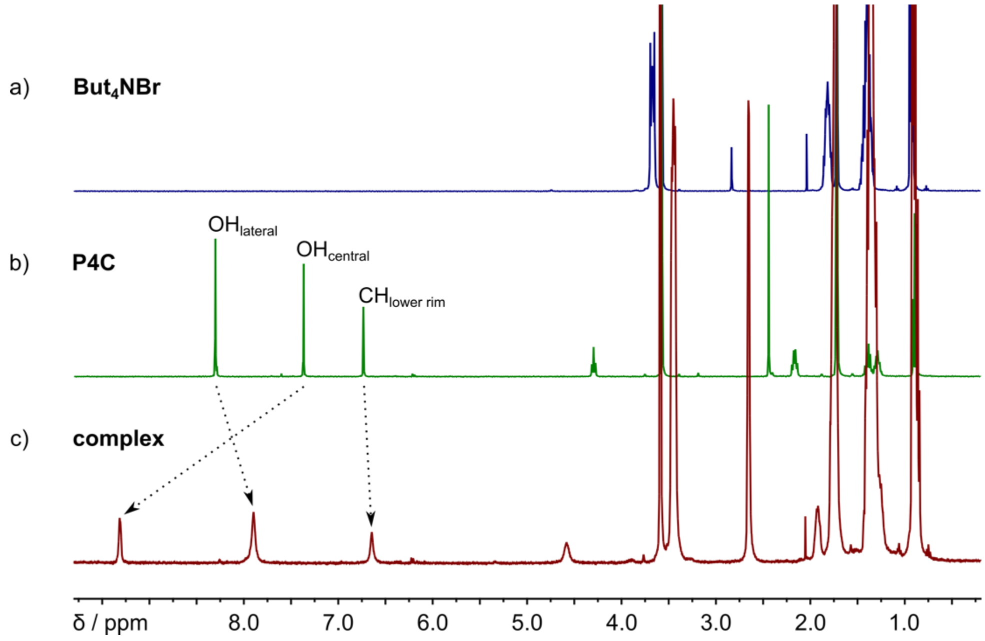

Figure S9. ^{1}H NMR spectra of (a) **TButABr**; (b) **P4C**; (c) complex of **P4C** and **But_4NBr** (400 MHz, 303 K, THF-d_8).

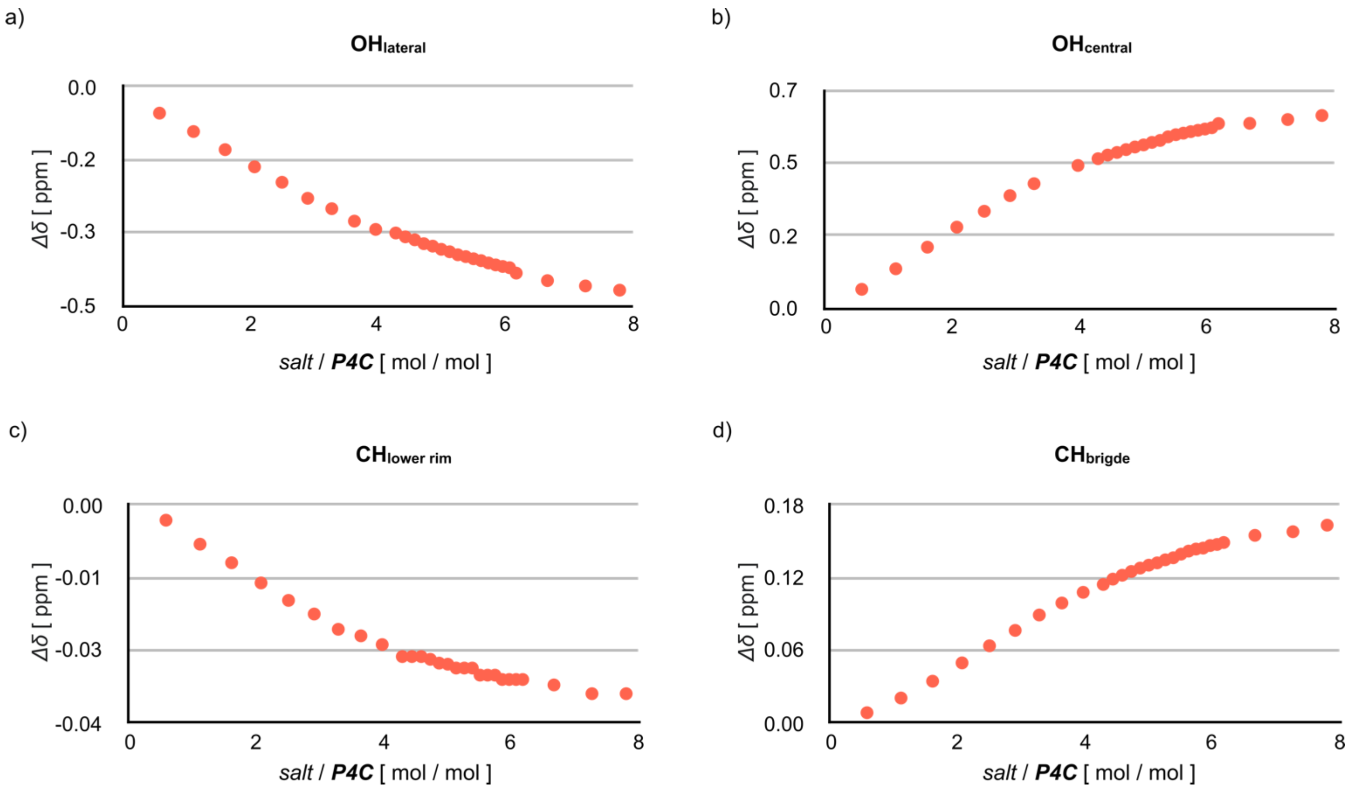

Figure S10. ^{1}H NMR titration curves for titration of **P4C** (C = 5.0 mM) with titrant **P4C** (C = 5.0 mM) + **But_4NBr** (C = 75 mM). ^{1}H NMR chemical shifts change for: (a) $OH_{lateral}$; (b) $OH_{central}$; (c) $CH_{lowr\ rim}$; (d) CH_{bridge} (400 MHz, 303 K, THF-d_8).

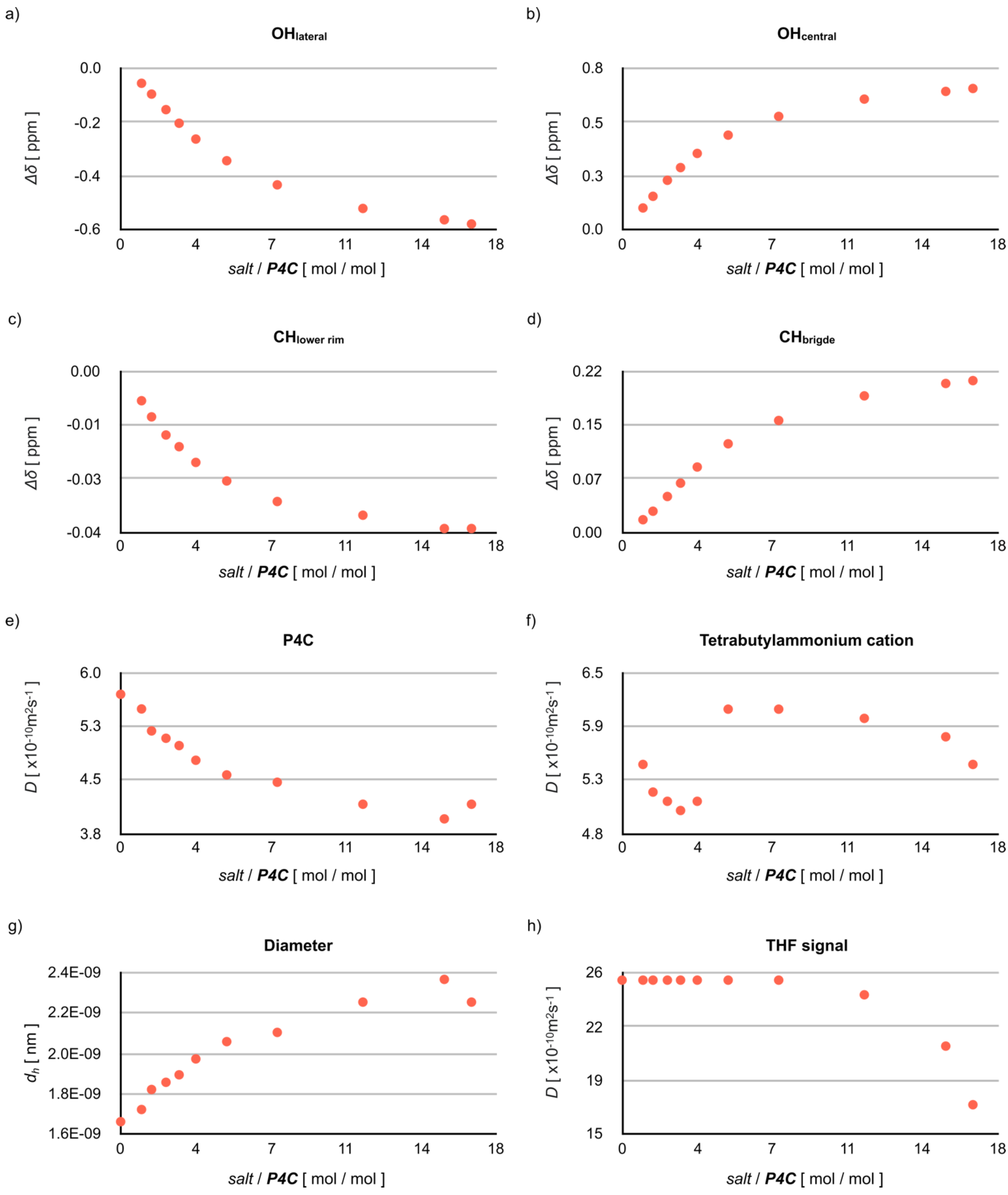

Figure S11. DOSY titration curves for titration of **P4C** (C = 2.5 mM) with titrant **P4C** (C = 2.5 mM) + **But$_4$NBr** (C = 65.6 mM). ^{1}H NMR chemical shift changes for: (a) $OH_{lateral}$; (b) $OH_{central}$; (c) $CH_{lower\ rim}$; (d) CH_{bridge}. Diffusion coefficient changes: for (e) **P4C;** (f) **But$_4$NBr**; (g) diameter of complex; (h) THF (600 MHz, 303 K, THF-d$_8$).

P4C	But_4N^+	Salt / M [mol / mol]
5.7	5.7	0
5.5	5.5	1
5.2	5.2	1.48
5.1	5.1	2.17
5.0	5.0	2.8
4.8	5.1	3.6
4.6	6.1	5.08
4.5	6.1	7.5
4.2	6.0	11.6
4.0	5.8	15.5
4.2	5.5	16.8

Table S3. Data for DOSY titration (**P4C** (C = 2.5 mM) with titrant **P4C** (C = 2.5 mM) + **But_4NBr** (C = 65.6 mM).).

5.5 Titration of pyrogallol[4]arene (P4C) with tetrapentylammonium bromide (Pen_4NBr)

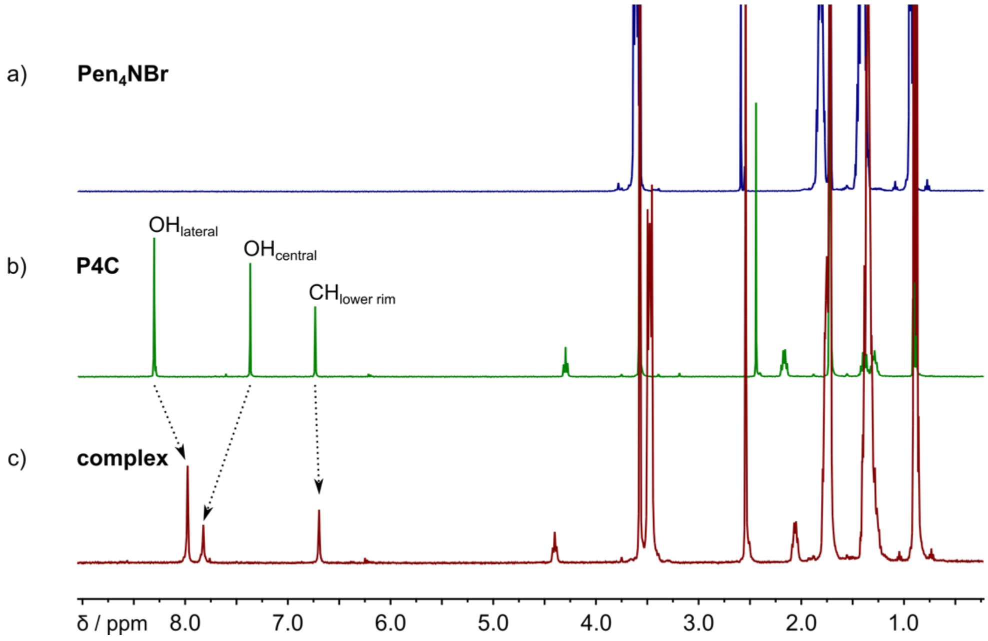

Figure S12. ^{1}H NMR spectra of (a) **Pen_4NBr**; (b) **P4C**; (c) complex of **P4C** and **Pen_4NBr** (400 MHz, 303 K, THF-d_8).

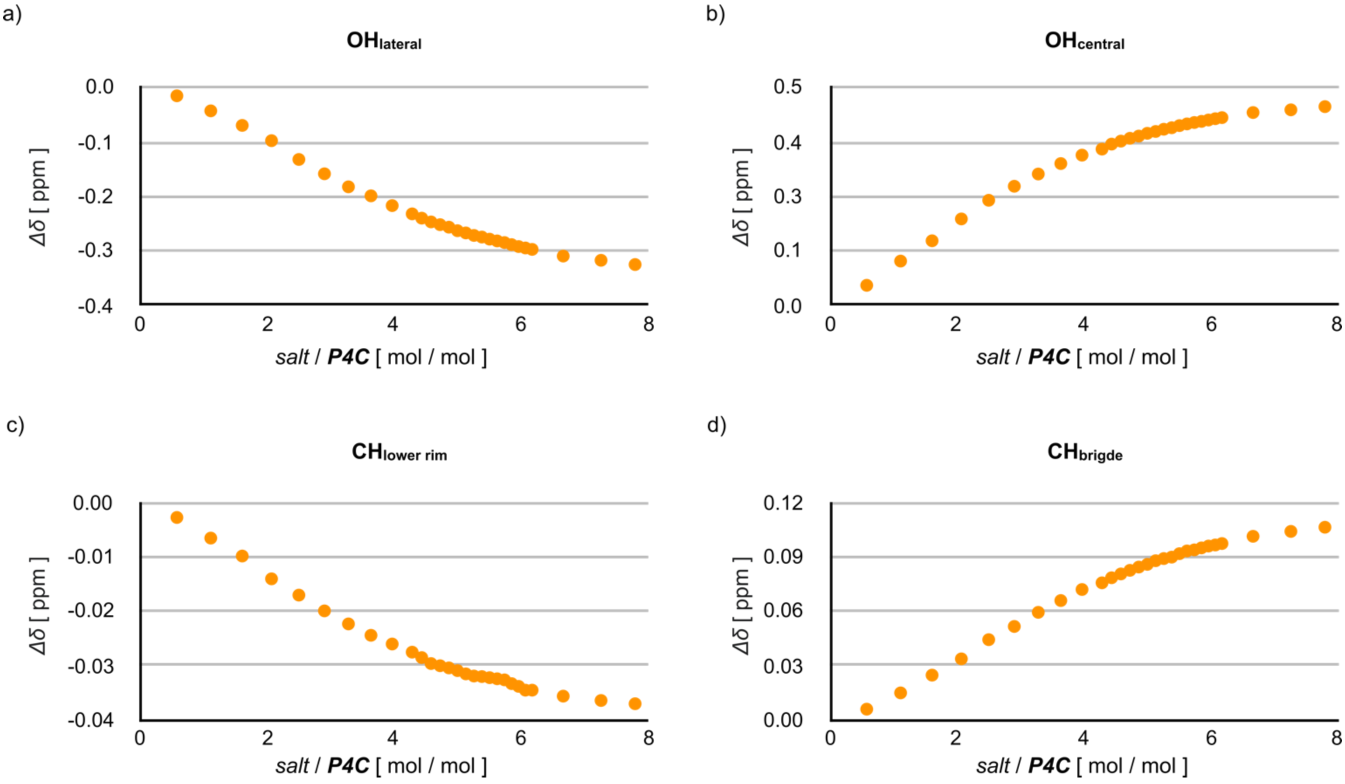

Figure S13. ^{1}H NMR titration curves for titration of **P4C** (C = 5.0 mM) with titrant **P4C** (C = 5.0 mM) + **Pen_4NBr** (C = 75 mM). ^{1}H NMR chemical shifts change for: (a) $OH_{lateral}$; (b) $OH_{central}$; (c) $CH_{lowr\ rim}$; (d) CH_{bridge} (400 MHz, 303 K, THF-d_8).

5.6 Titration of pyrogallol[4]arene (P4C) with tetraoctylammonium bromide (Oct_4NBr)

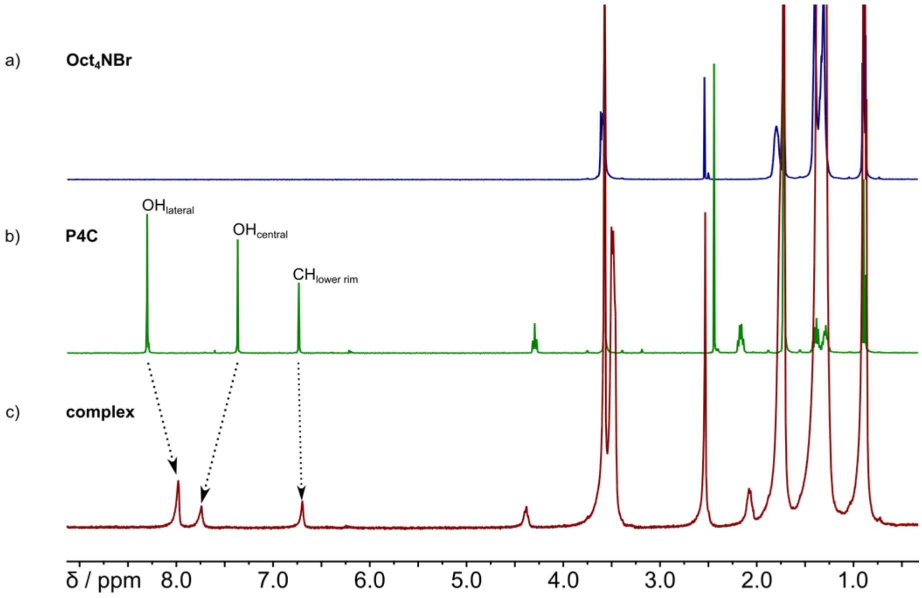

Figure S14. ^{1}H NMR spectra of (a) **Oct_4NBr**; (b) **P4C**; (c) complex of **P4C** and **Oct_4NBr** (400 MHz, 303 K, THF-d_8).

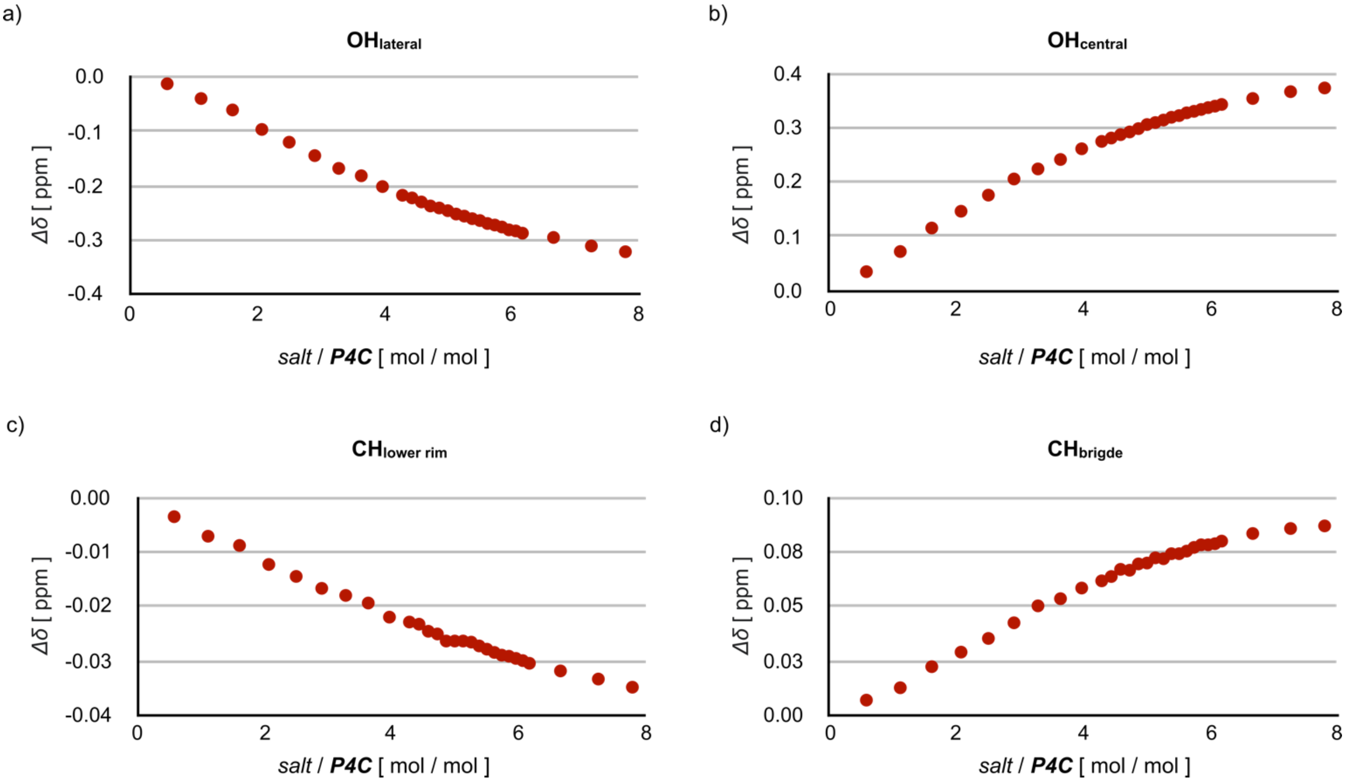

Figure S15. ^{1}H NMR titration curves for titration of **P4C** (C = 5.0 mM) with titrant **P4C** (C = 5.0 mM) + **Oct_4NBr** (C = 75 mM). ^{1}H NMR chemical shifts change for: (a) $OH_{lateral}$; (b) $OH_{central}$; (c) $CH_{lowr\ rim}$; (d) CH_{bridge} (400 MHz, 303 K, THF-d_8).

5.7 Titration of pyrogallol[4]arene (P4C) with tetradecylammonium bromide (Dec_4NBr)

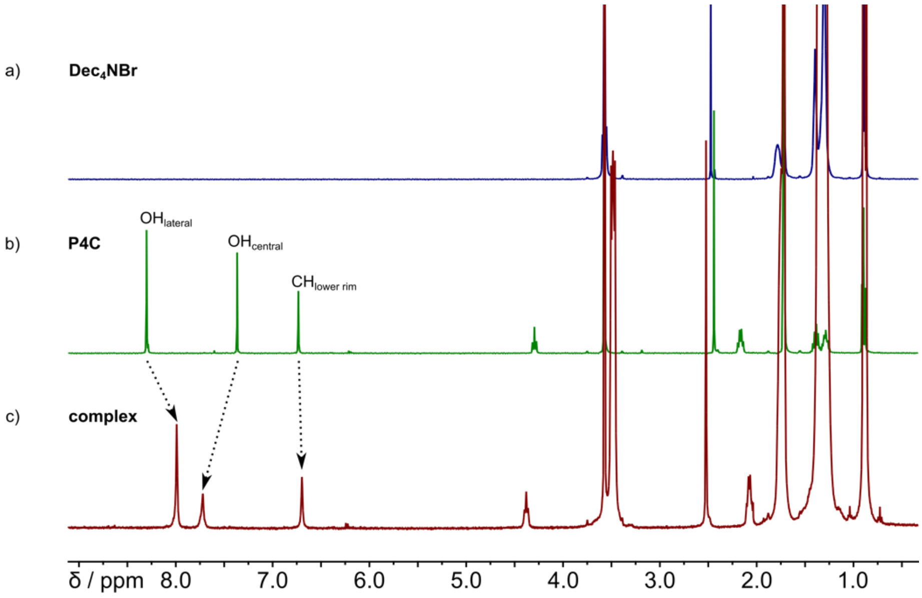

Figure S16. ^{1}H NMR spectra of (a) **Dec_4NBr**; (b) **P4C**; (c) complex of **P4C** and **Dec_4NBr** (400 MHz, 303 K, THF-d_8).

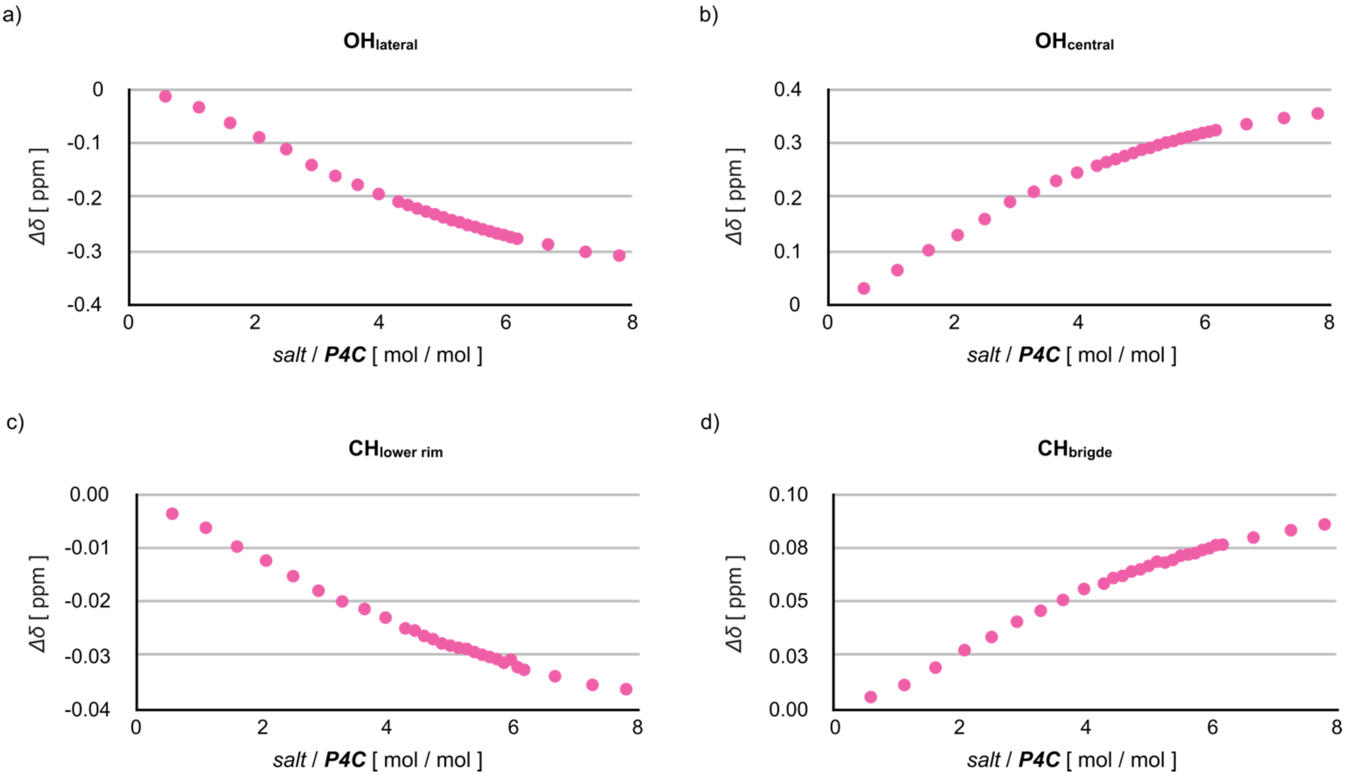

Figure S17. ^{1}H NMR titration curves for titration of **P4C** (C = 5.0 mM) with titrant **P4C** (C = 5.0 mM) + **Dec_4NBr** (C = 75 mM). ^{1}H NMR chemical shifts change for: (a) $OH_{lateral}$; (b) $OH_{central}$; (c) $CH_{lowr\ rim}$; (d) CH_{bridge} (400 MHz, 303 K, THF-d_8).

6. Titrations of Pyrogallol[4]arene (P4H) in THF

6.1 Titration of pyrogallol[4]arene (P4H) with tetrabutylammonium chloride (But_4NCl)

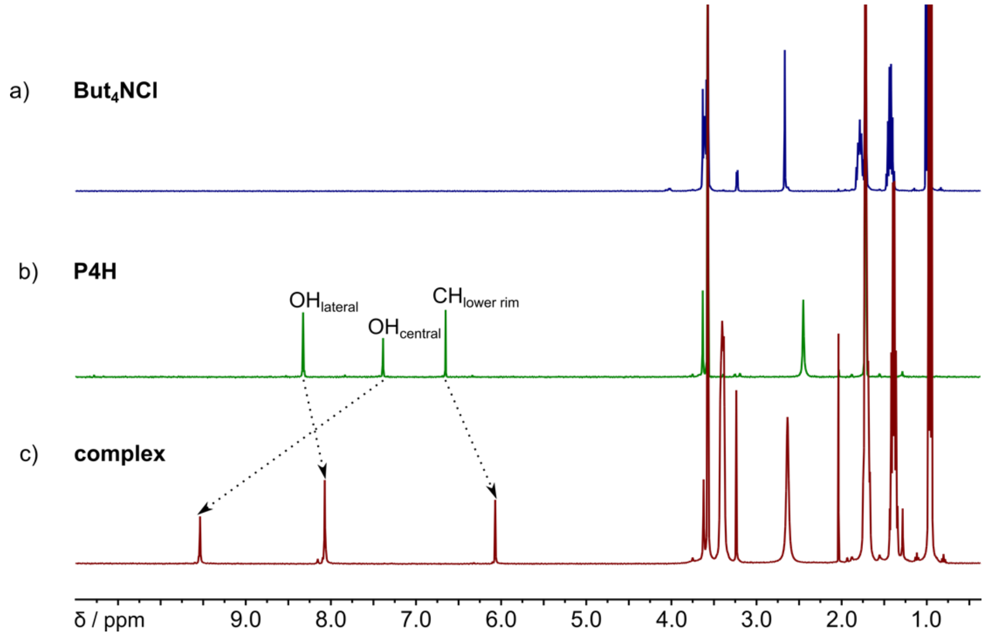

Figure S18. ^{1}H NMR spectra of (a) **But_4NCl**; (b) **P4H**; (c) complex of **P4H** and **But_4NCl** (400 MHz, 303 K, THF-d_8).

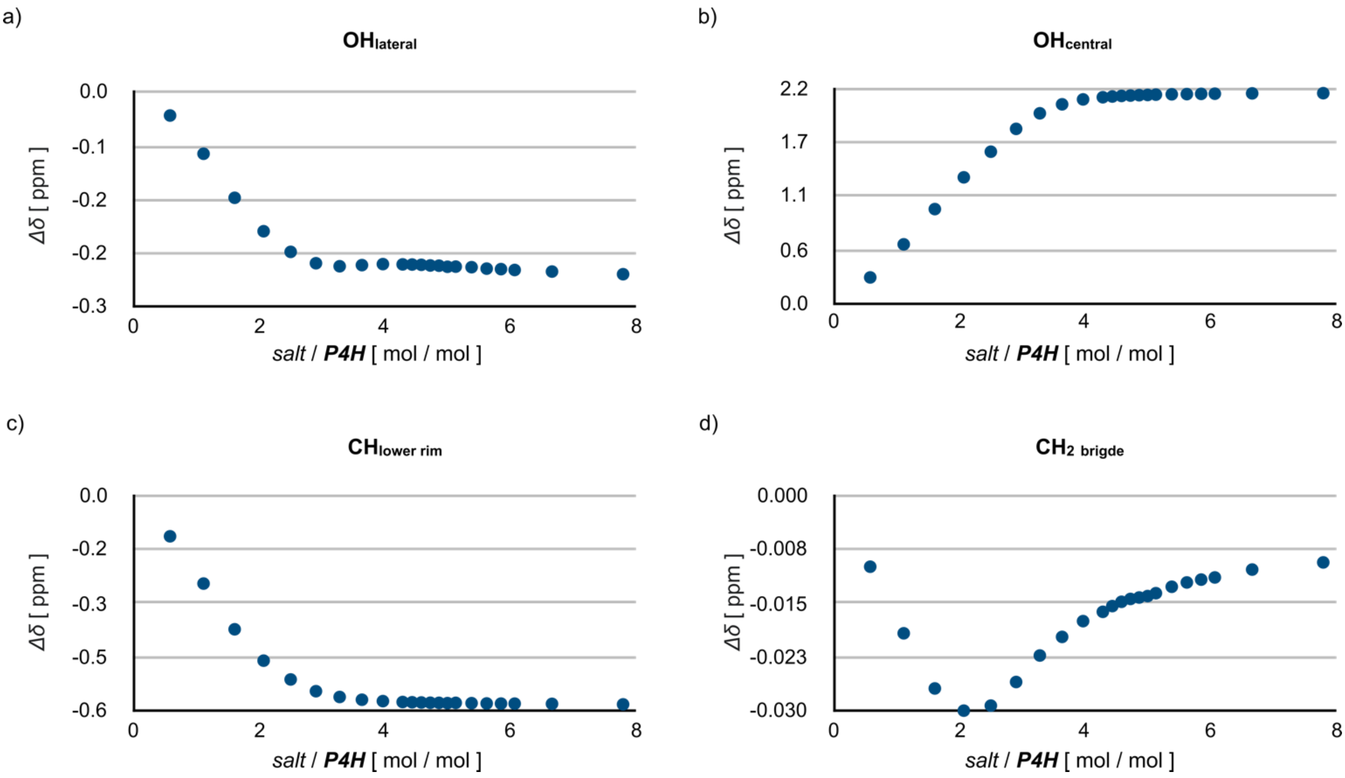

Figure S19. ^{1}H NMR titration curves for titration of **P4H** (C = 5.0 mM) with titrant **P4H** (C = 5.0 mM) + **But_4NCl** (C = 75 mM). ^{1}H NMR chemical shifts change for: (a) $OH_{lateral}$; (b) $OH_{central}$; (c) $CH_{lowr\ rim}$; (d) $CH_{2\ bridge}$ (400 MHz, 303 K, THF-d_8).

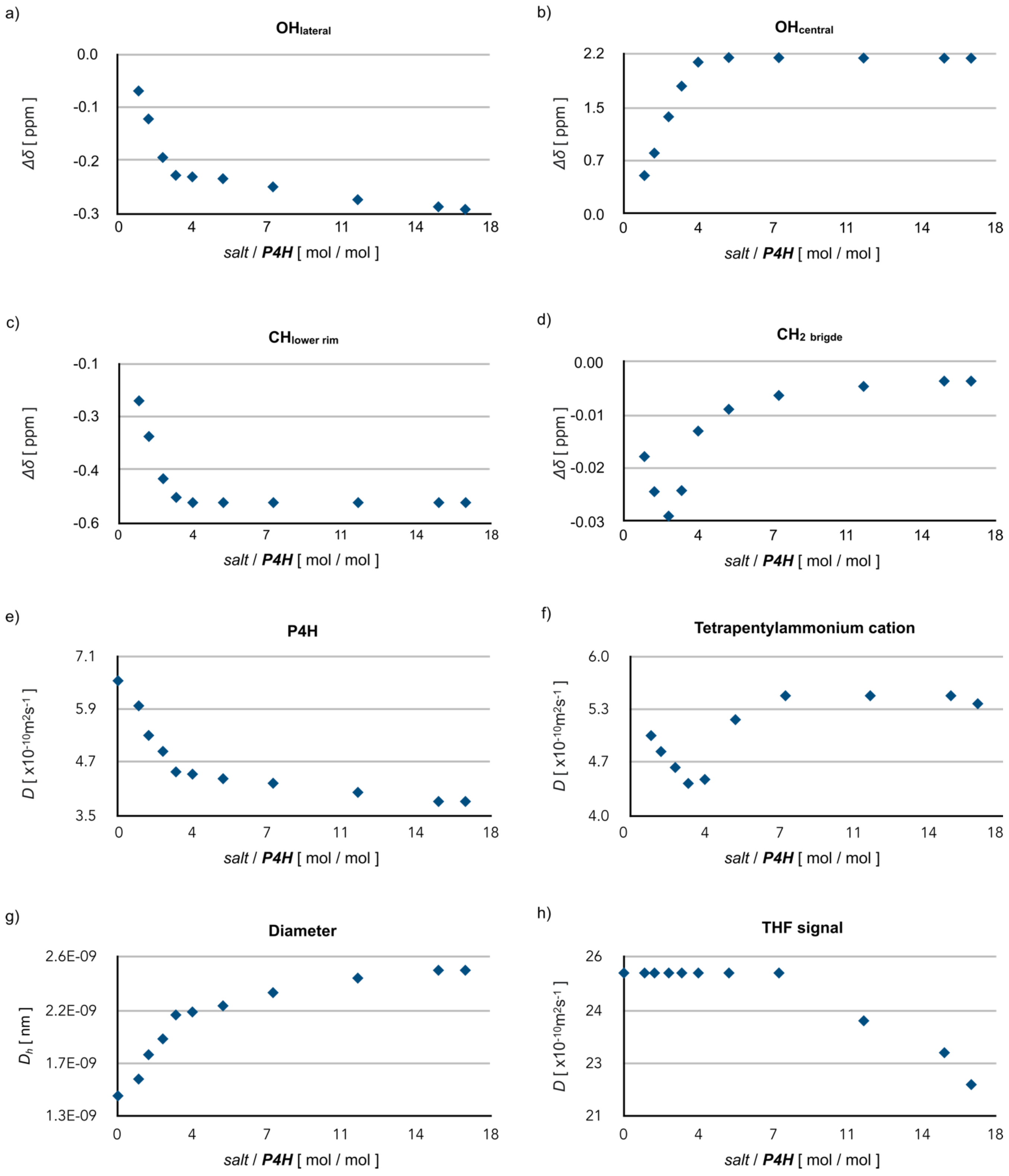

Figure S20. DOSY titration curves for titration of **P4H** (C = 2.5 mM) with titrant **P4H** (C = 2.5 mM) + **But$_4$NCl** (C = 65.6 mM). ^{1}H NMR chemical shift changes for: (a) $OH_{lateral}$; (b) $OH_{central}$; (c) $CH_{lower\ rim}$; (d) $CH_{2\ bridge}$. Diffusion coefficient changes: for (e) **P4H;** (f) **But$_4$NCl**; (g) diameter of complex; (h) THF (600 MHz, 303 K, THF-d$_8$).

P4H	Pen_4N^+	Salt / M [mol / mol]
6.5		0
5.9	5	1
5.3	4.8	1.48
4.9	4.6	2.17
4.5	4.4	2.8
4.4	4.5	3.6
4.3	5.2	5.08
4.2	5.5	7.5
4.0	5.5	11.6
3.8	5.5	15.5
3.8	5.5	16.8

Table S4. Data for DOSY titration (**P4H** (C = 2.5 mM) with titrant **P4H** (C = 2.5 mM) + **But_4NCl** (C = 65.6 mM).).

6.2 Titration of pyrogallol[4]arene (P4H) with tetrapentylammonium chloride (Pen_4NCl)

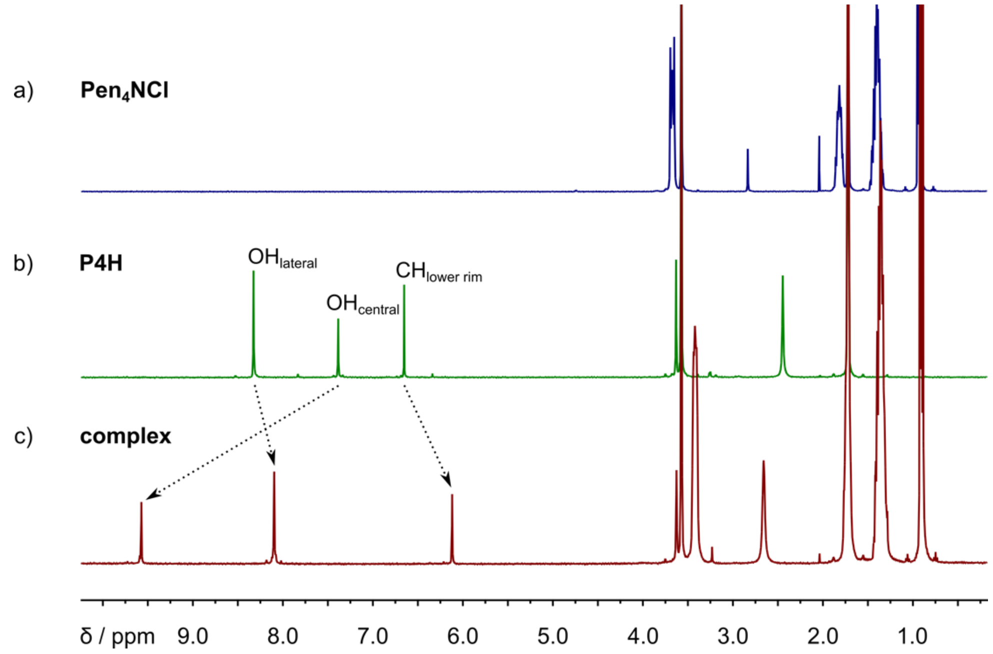

Figure S21. ^{1}H NMR spectra of (a) **Pen_4NCl**; (b) **P4H**; (c) complex of **P4H** and **Pen_4NCl** (400 MHz, 303 K, THF-d_8).

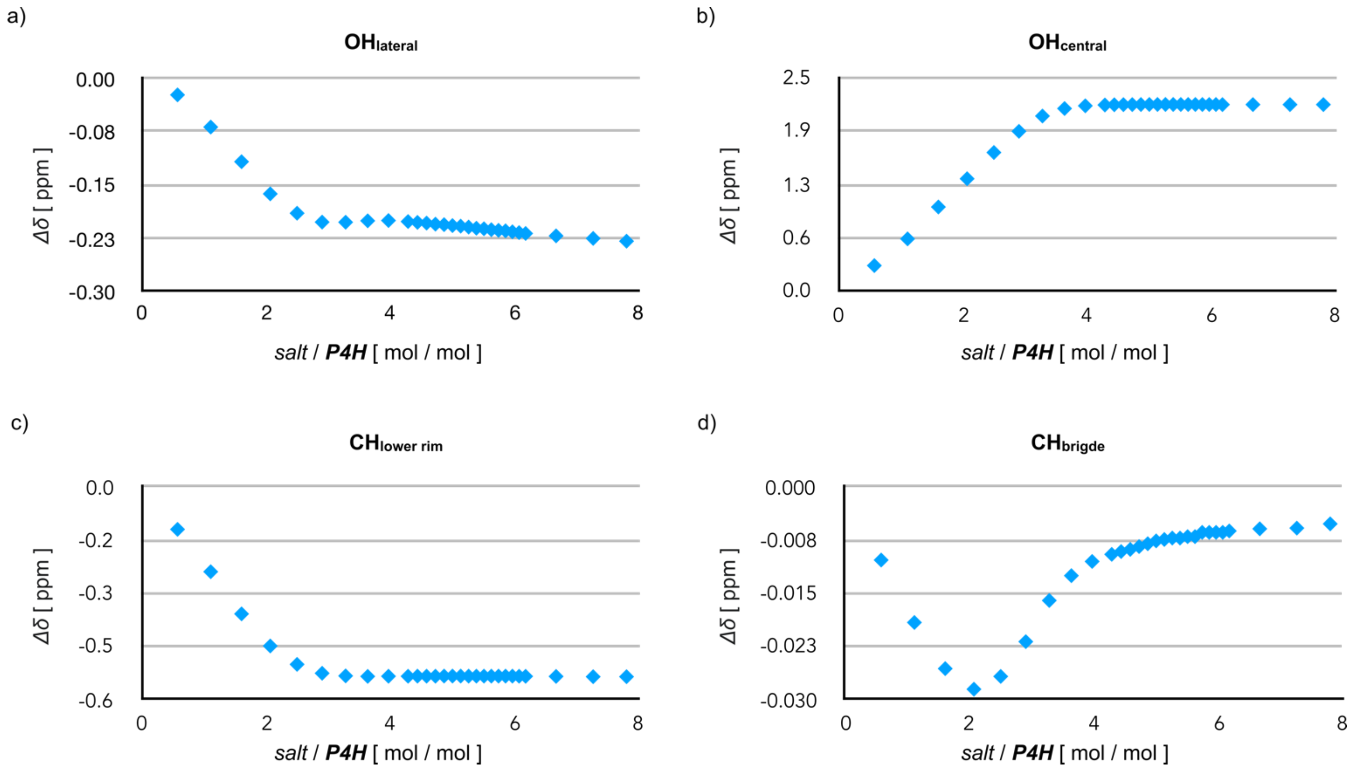

Figure S22. ^{1}H NMR titration curves for titration of **P4H** (C = 5.0 mM) with titrant **P4H** (C = 5.0 mM) + **Pen_4NCl** (C = 75 mM). ^{1}H NMR chemical shifts change for: (a) $OH_{lateral}$; (b) $OH_{central}$; (c) $CH_{lowr\ rim}$; (d) $CH_{2\ bridge}$ (400 MHz, 303 K, THF-d_8).

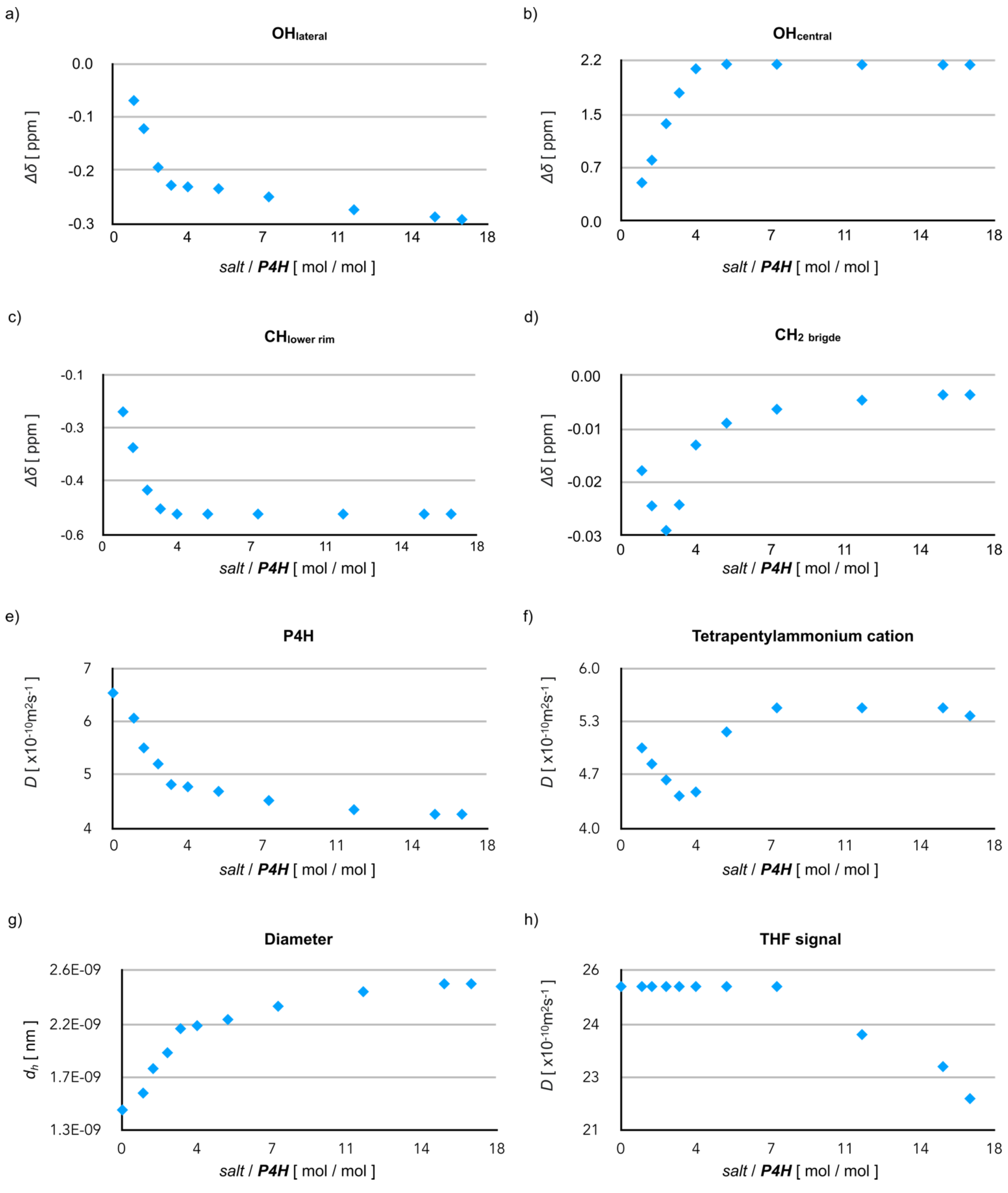

Figure S23. DOSY titration curves for titration of **P4H** (C = 2.5 mM) with titrant **P4H** (C = 2.5 mM) + **Pen_4NCl** (C = 65.6 mM). ^1H NMR chemical shift changes for: (a) $OH_{lateral}$; (b) $OH_{central}$; (c) $CH_{lower\ rim}$; (d) $CH_{2\ bridge}$. Diffusion coefficient changes: for (e) **P4H;** (f) **Pen_4NCl**; (g) diameter of complex; (h) THF (600 MHz, 303 K, THF-d_8).

P4H	Pen_4N^+	salt / M [mol / mol]
6.5		0
5.9	5.0	1
5.3	4.8	1.48
4.9	4.6	2.17
4.5	4.4	2.8
4.4	4.5	3.6
4.3	5.2	5.08
4.2	5.5	7.5
4.0	5.5	11.6
3.9	5.5	15.5
3.8	5.5	16.8

Table S5. Data for DOSY titration (**P4H** (C = 2.5 mM) with titrant **P4H** (C = 2.5 mM) + **Pen_4NCl** (C = 65.6 mM))

6.3 Titration of pyrogallol[4]arene (P4H) with tetraoctylammonium chloride (Oct_4NCl)

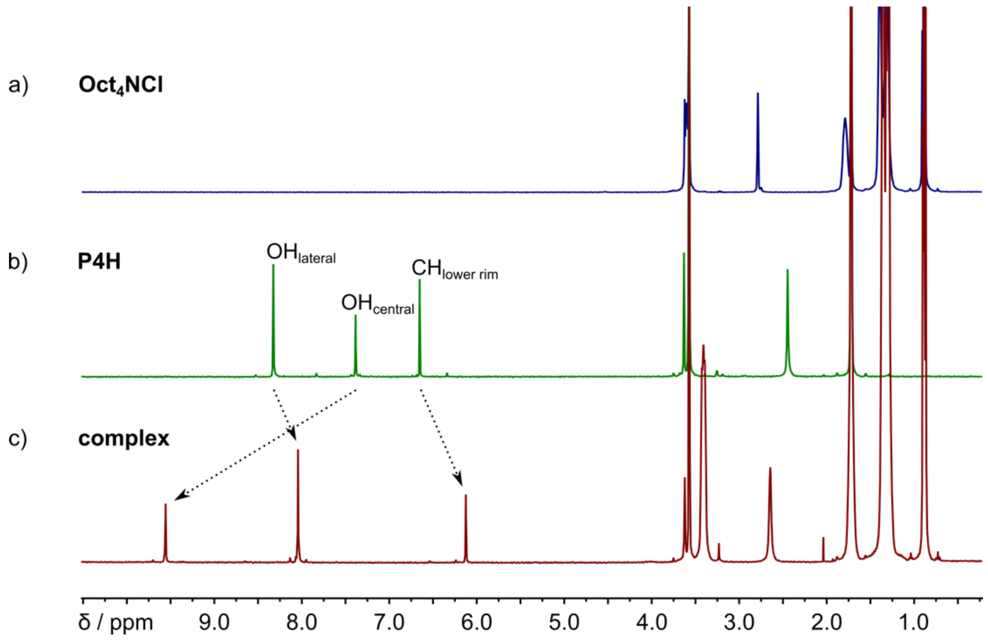

Figure S24. ^{1}H NMR spectra of (a) **Oct_4NCl**; (b) **P4H**; (c) complex of **P4H** and **Oct_4NCl** (400 MHz, 303 K, THF-d_8).

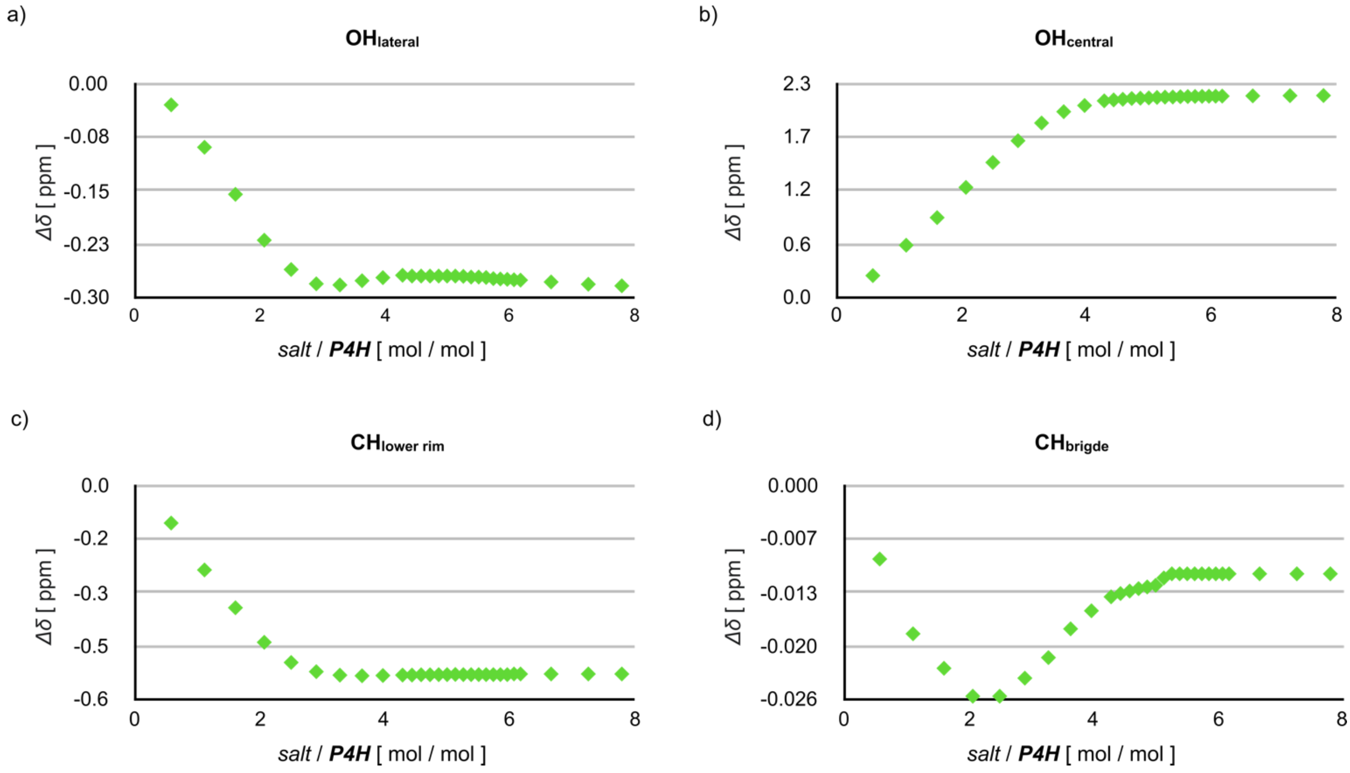

Figure S25. ^{1}H NMR titration curves for titration of **P4H** (C = 5.0 mM) with titrant **P4H** (C = 5.0 mM) + **Oct_4NCl** (C = 75 mM). ^{1}H NMR chemical shifts change for: (a) $OH_{lateral}$; (b) $OH_{central}$; (c) $CH_{lowr\ rim}$; (d) $CH_{2\ bridge}$ (400 MHz, 303 K, THF-d_8).

6.4 Titration of pyrogallol[4]arene (P4H) with tetrabutylammonium bromide (But_4NBr)

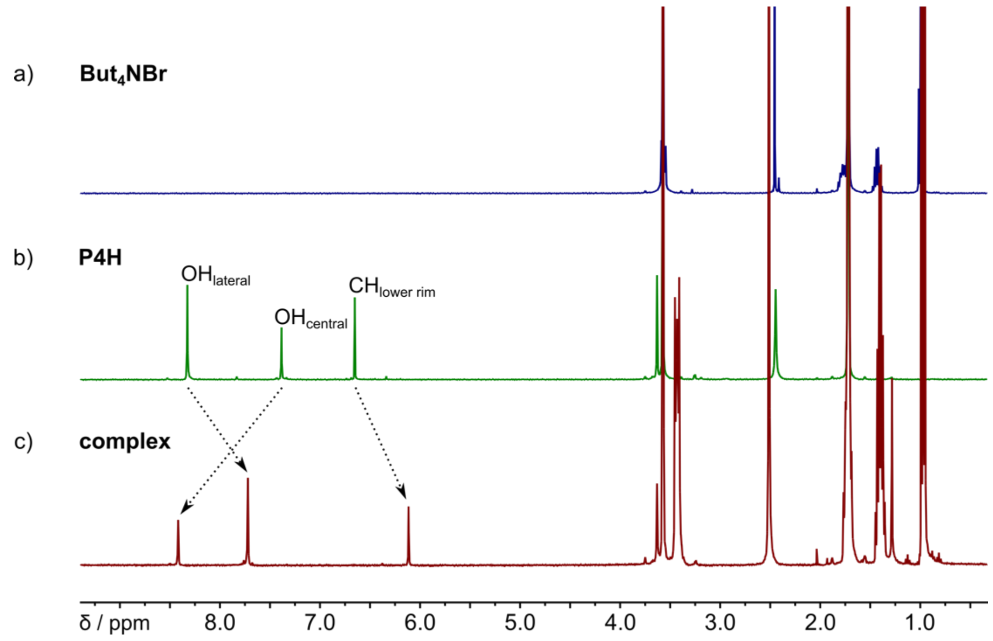

Figure S26. ^{1}H NMR spectra of (a) **But₄NBr**; (b) **P4H**; (c) complex of **P4H** and **But₄NBr** (400 MHz, 303 K, THF-d₈).

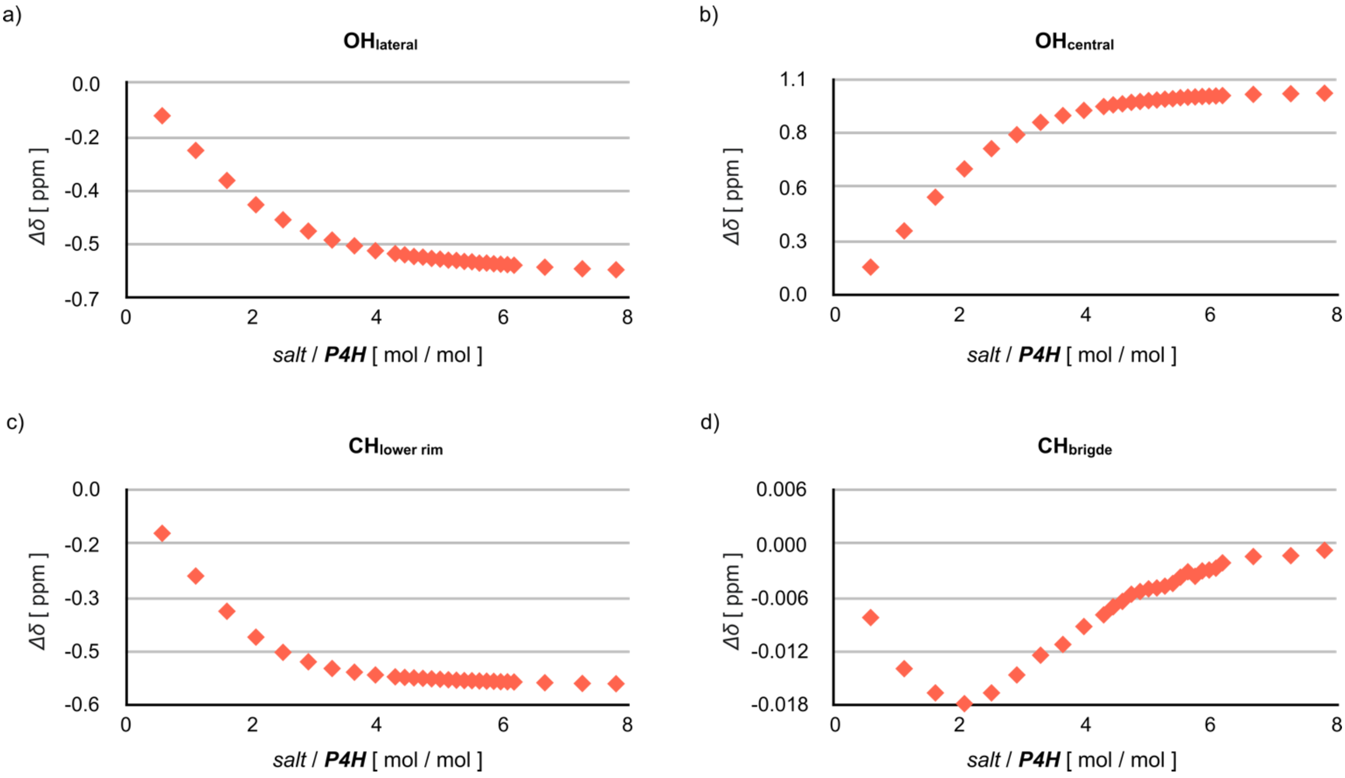

Figure S27. ^{1}H NMR titration curves for titration of **P4H** (C = 5.0 mM) with titrant **P4H** (C = 5.0 mM) + **But₄NBr** (C = 75 mM). ^{1}H NMR chemical shifts change for: (a) $OH_{lateral}$; (b) $OH_{central}$; (c) $CH_{lowr\ rim}$; (d) $CH_{2\ bridge}$ (400 MHz, 303 K, THF-d₈).

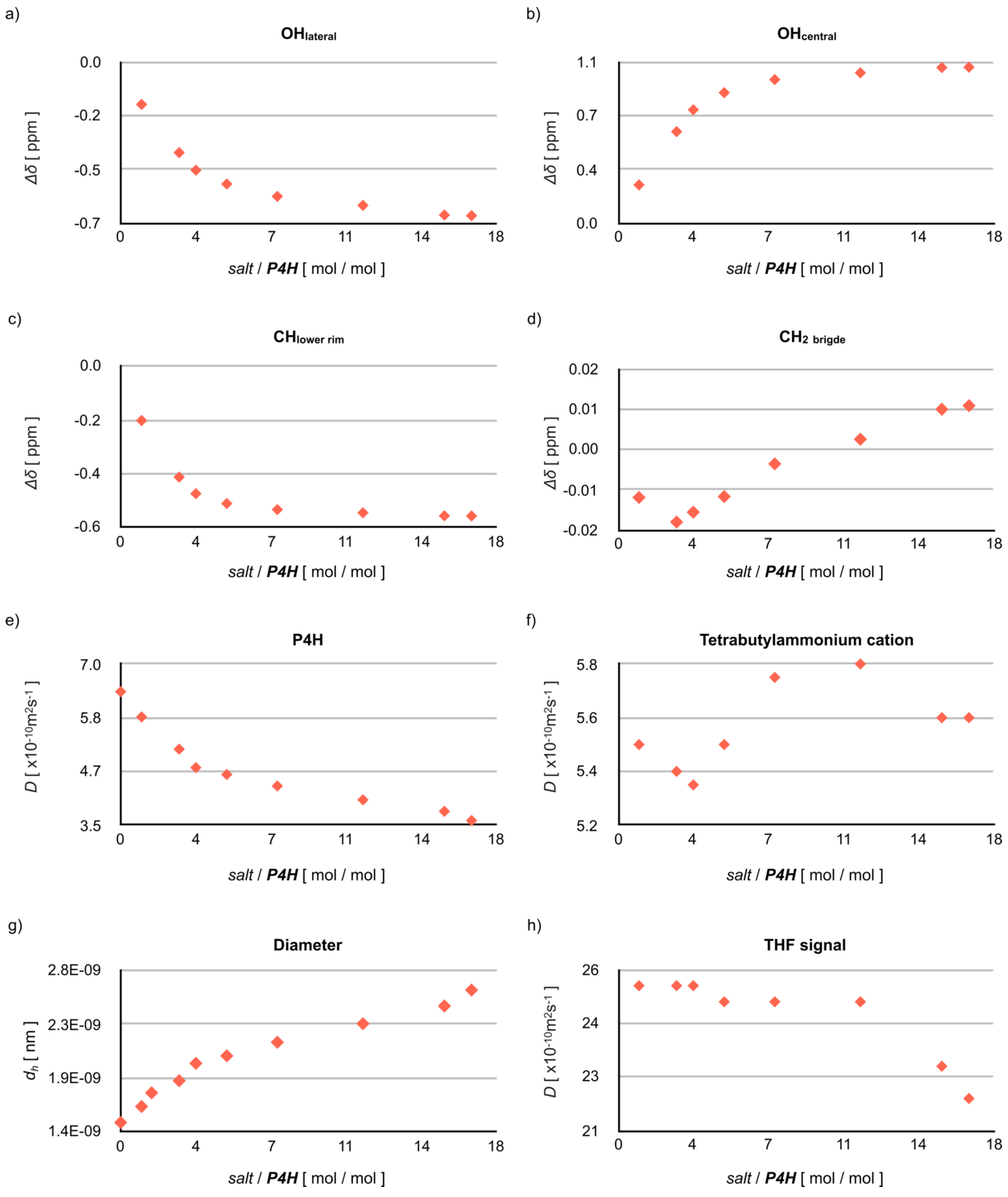

Figure S28. DOSY titration curves for titration of **P4H** (C = 2.5 mM) with titrant **P4H** (C = 2.5 mM) + **But$_4$NBr** (C = 65.6 mM). ^{1}H NMR chemical shift changes for: (a) $OH_{lateral}$; (b) $OH_{central}$; (c) $CH_{lower\ rim}$; (d) $CH_{2\ bridge}$. Diffusion coefficient changes: for (e) **P4H;** (f) **But$_4$NBr**; (g) diameter of complex; (h) THF (600 MHz, 303 K, THF-d$_8$).

P4H	But_4N^+	Salt / M [mol / mol]
6.4		0
5.85	5.5	1
5.15	5.4	2.8
4.75	5.35	3.6
4.6	5.5	5.08
4.35	5.75	7.5
4.05	5.8	11.6
3.8	5.6	15.5
3.6	5.6	16.8

Table S6. Data for DOSY titration (**P4H** (C = 2.5 mM) with titrant **P4H** (C = 2.5 mM) + **But_4NBr** (C = 65.6 mM)).

6.5 Titration of pyrogallol[4]arene (P4H) with tetrapentylammonium bromide (Pen_4NBr)

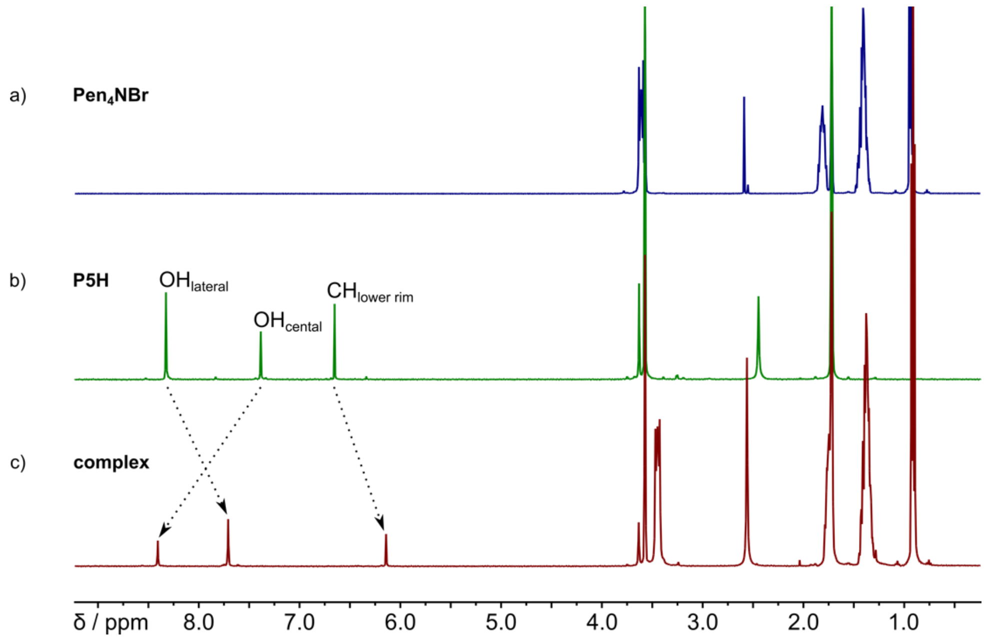

Figure S29. ^{1}H NMR spectra of (a) **Pen_4NBr**; (b) **P4H**; (c) complex of **P4H** and **Pen_4NBr** (400 MHz, 303 K, THF-d_8).

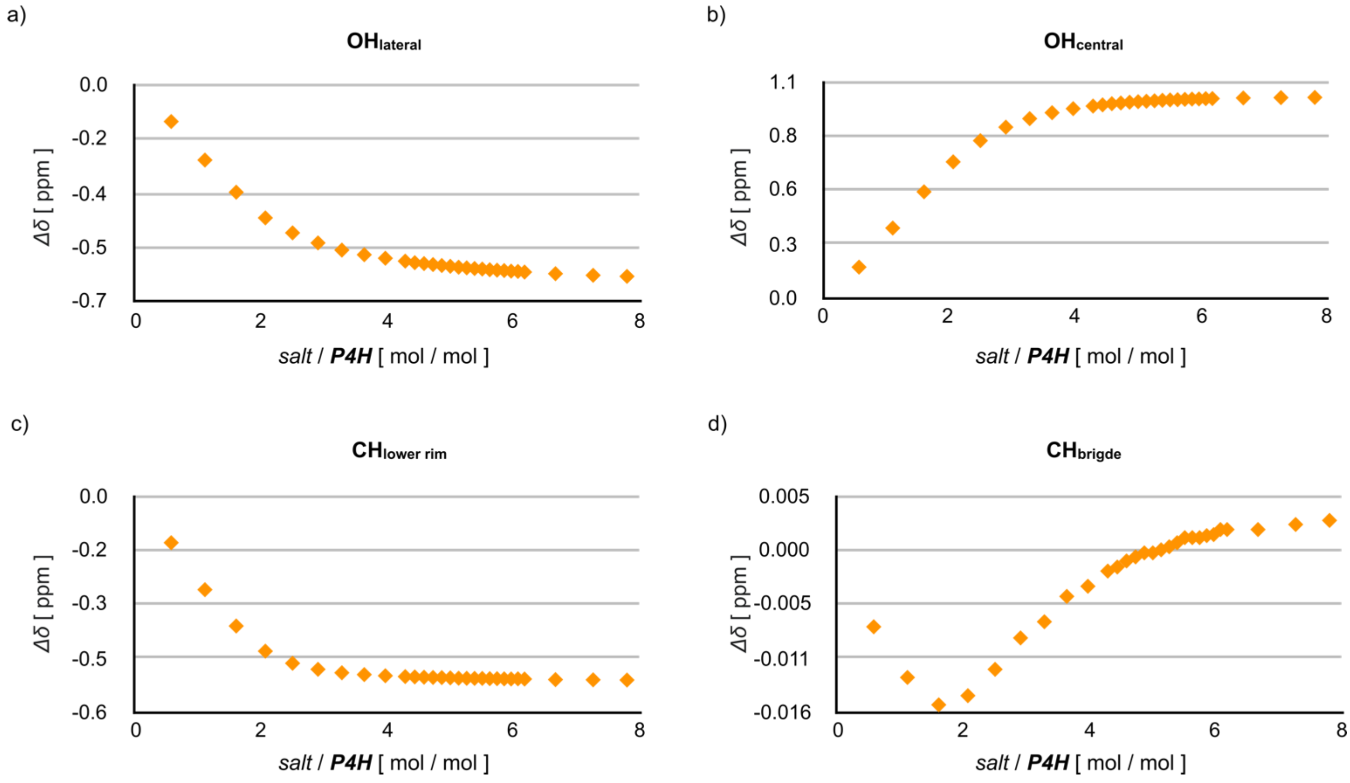

Figure S30. ^{1}H NMR titration curves for titration of **P4H** (C = 5.0 mM) with titrant **P4H** (C = 5.0 mM) + **Pen_4NBr** (C = 75 mM). ^{1}H NMR chemical shifts change for: (a) $OH_{lateral}$; (b) $OH_{central}$; (c) $CH_{lowr\ rim}$; (d) $CH_{2\ bridge}$ (400 MHz, 303 K, THF-d_8).

6.6 Titration of pyrogallol[4]arene (P4H) with tetraoctylammonium bromide (Oct_4NBr)

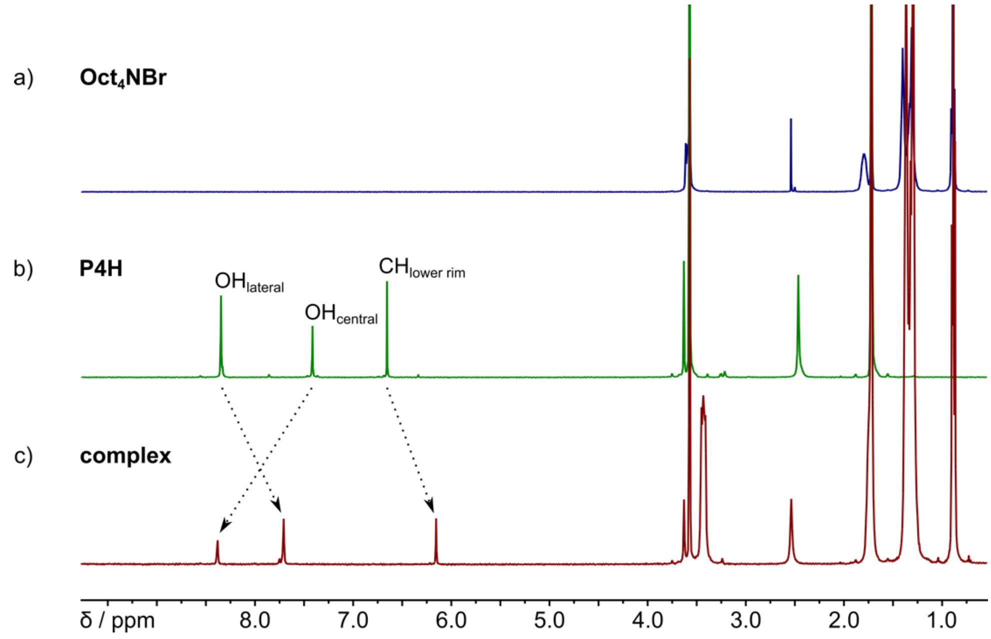

Figure S31. ^{1}H NMR spectra of (a) **Oct_4NBr**; (b) **P4H**; (c) complex of **P4H** and **Oct_4NBr** (400 MHz, 303 K, THF-d_8).

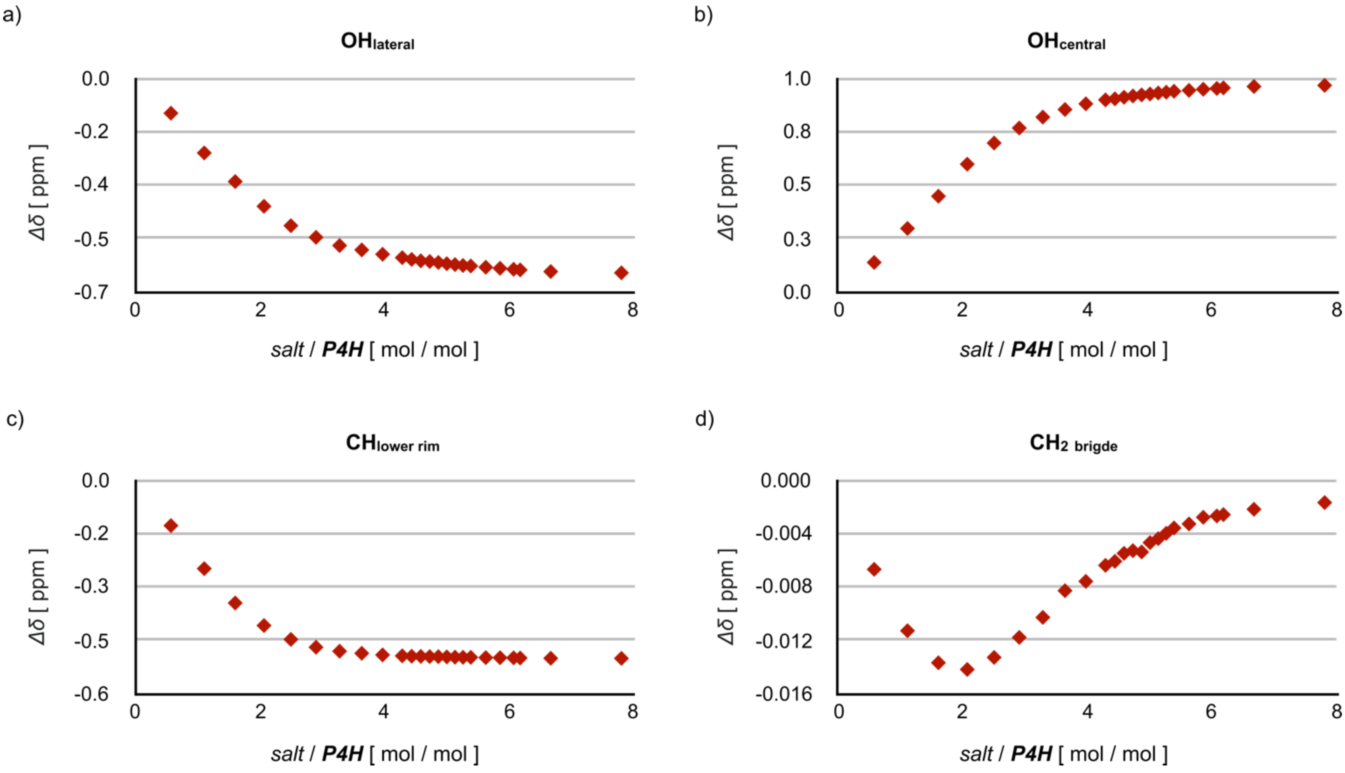

Figure S32. ^{1}H NMR titration curves for titration of **P4H** (C = 5.0 mM) with titrant **P4H** (C = 5.0 mM) + **Oct_4NBr** (C = 75 mM). ^{1}H NMR chemical shifts change for: (a) $OH_{lateral}$; (b) $OH_{central}$; (c) $CH_{lowr\ rim}$; (d) $CH_{2\ bridge}$ (400 MHz, 303 K, THF-d_8).

6.7 Titration of pyrogallol[4]arene (P4H) with tetradecylammonium bromide (Dec_4NBr)

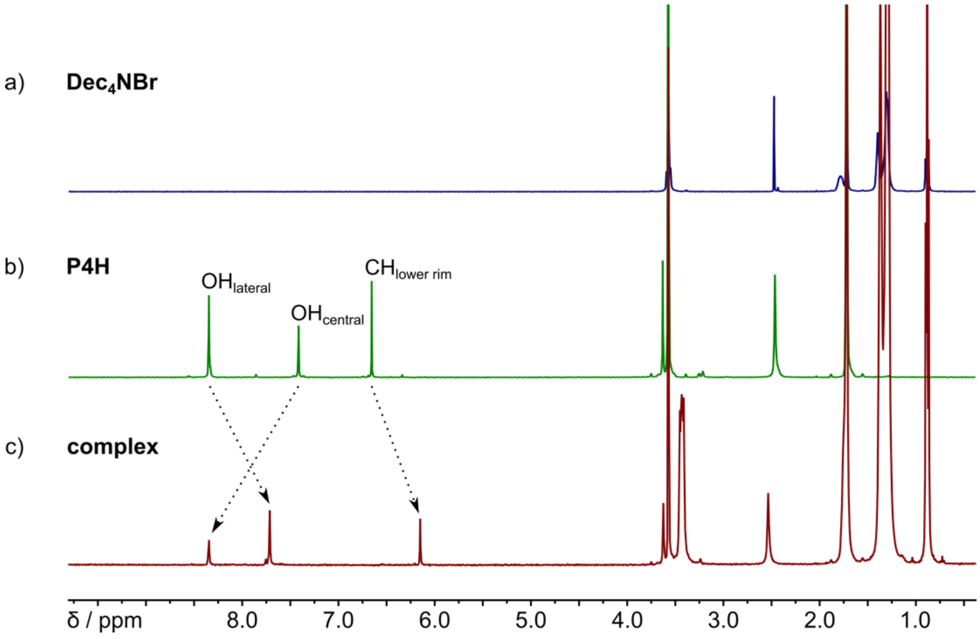

Figure S33. ^{1}H NMR spectra of (a) **Dec_4NBr**; (b) **P4H**; (c) complex of **P4H** and **Dec_4NBr** (400 MHz, 303 K, THF-d_8).

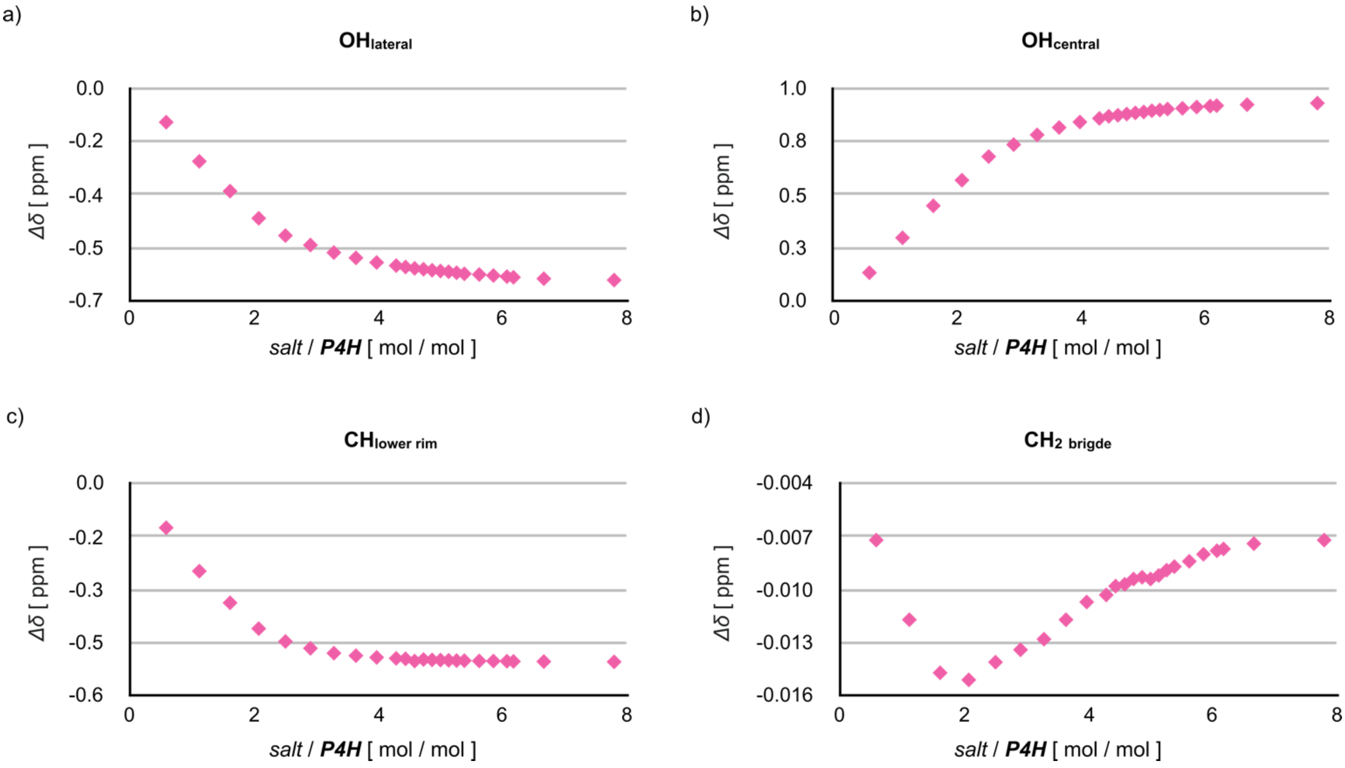

Figure S34. ^{1}H NMR titration curves for titration of **P4H** (C = 5.0 mM) with titrant **P4H** (C = 5.0 mM) + **Dec_4NBr** (C = 75 mM). ^{1}H NMR chemical shifts change for: (a) $OH_{lateral}$; (b) $OH_{central}$; (c) $CH_{lowr\ rim}$; (d) $CH_{2\ bridge}$ (400 MHz, 303 K, THF-d_8).

7. Titrations of Resorcin[4]arene (R4C) in THF

7.1 Titration of resorcin[4]arene (R4C) with tetrabutylammonium chloride (But_4NCl)

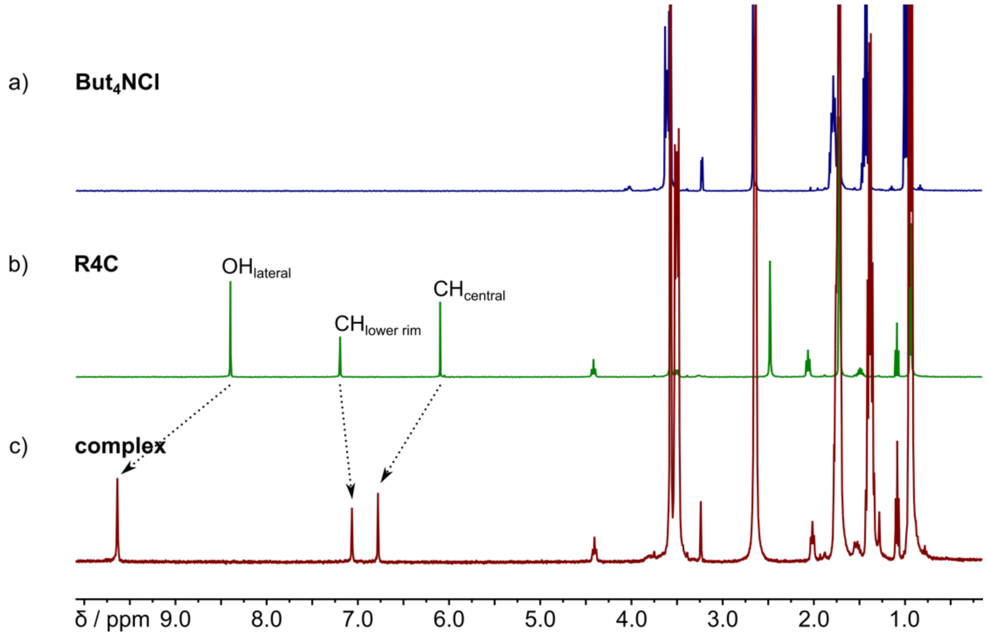

Figure S35. ^{1}H NMR spectra of (a) **But_4NCl**; (b) **R4C**; (c) complex of **R4C** and **But_4NCl** (400 MHz, 303 K, THF-d_8).

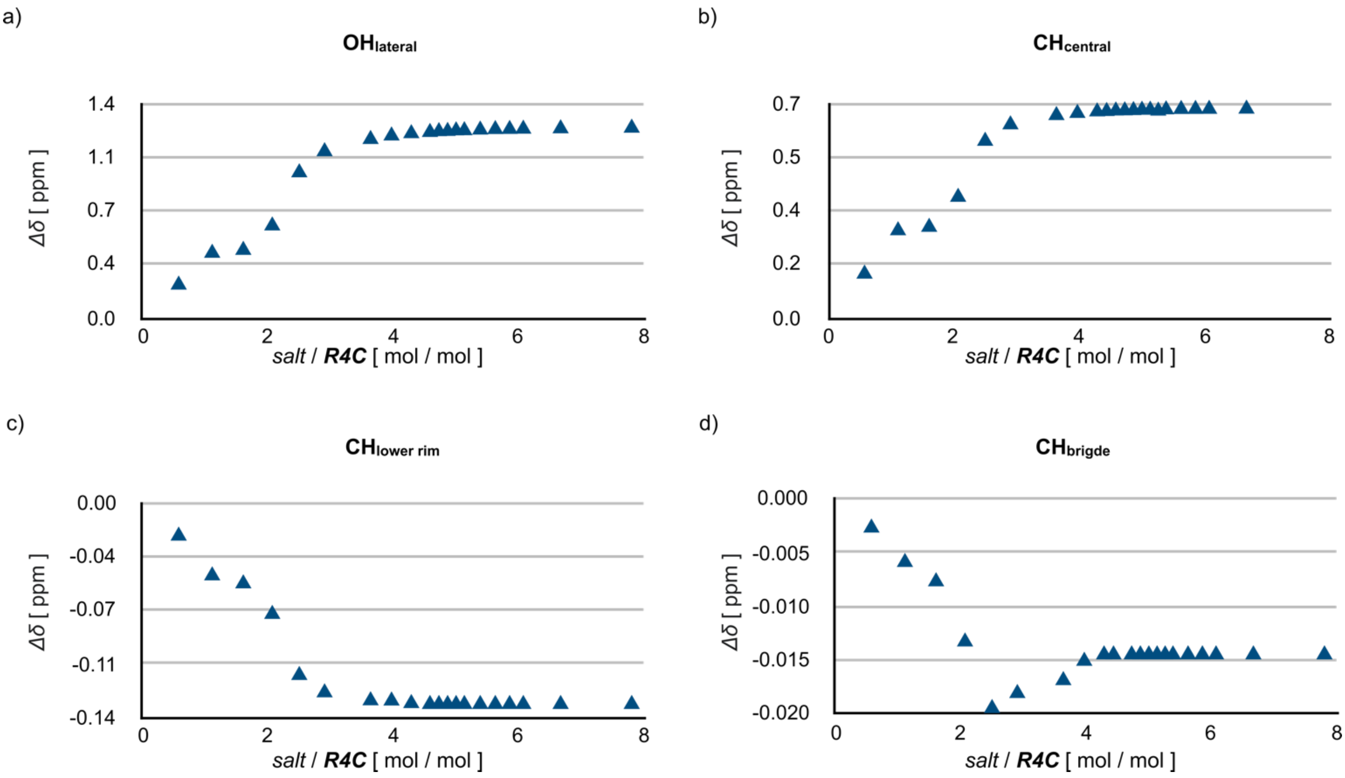

Figure S36. ^{1}H NMR titration curves for titration of **R4C** (C = 5.0 mM) with titrant **R4C** (C = 5.0 mM) + **But_4NCl** (C = 75 mM). ^{1}H NMR chemical shifts change for: (a) $OH_{lateral}$; (b) $OH_{central}$; (c) $CH_{lowr\ rim}$; (d) CH_{bridge} (400 MHz, 303 K, THF-d_8).

7.2 Titration of resorcin[4]arene (R4C) with tetrapentylammonium chloride (Pen_4NCl)

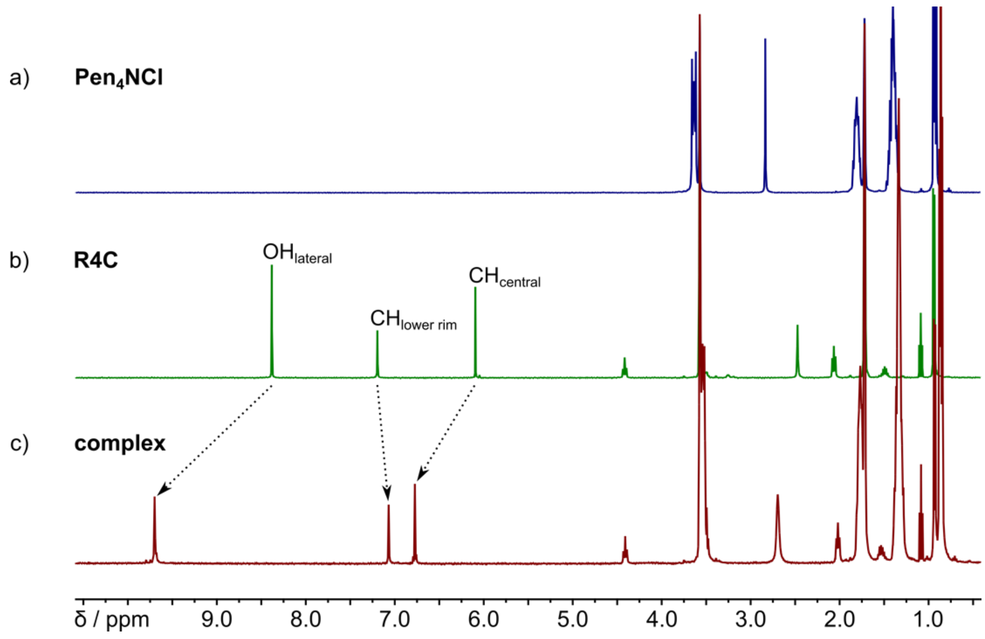

Figure S37. ^{1}H NMR spectra of (a) **Pen_4NCl**; (b) **R4C**; (c) complex of **R4C** and **Pen_4NCl** (400 MHz, 303 K, THF-d_8).

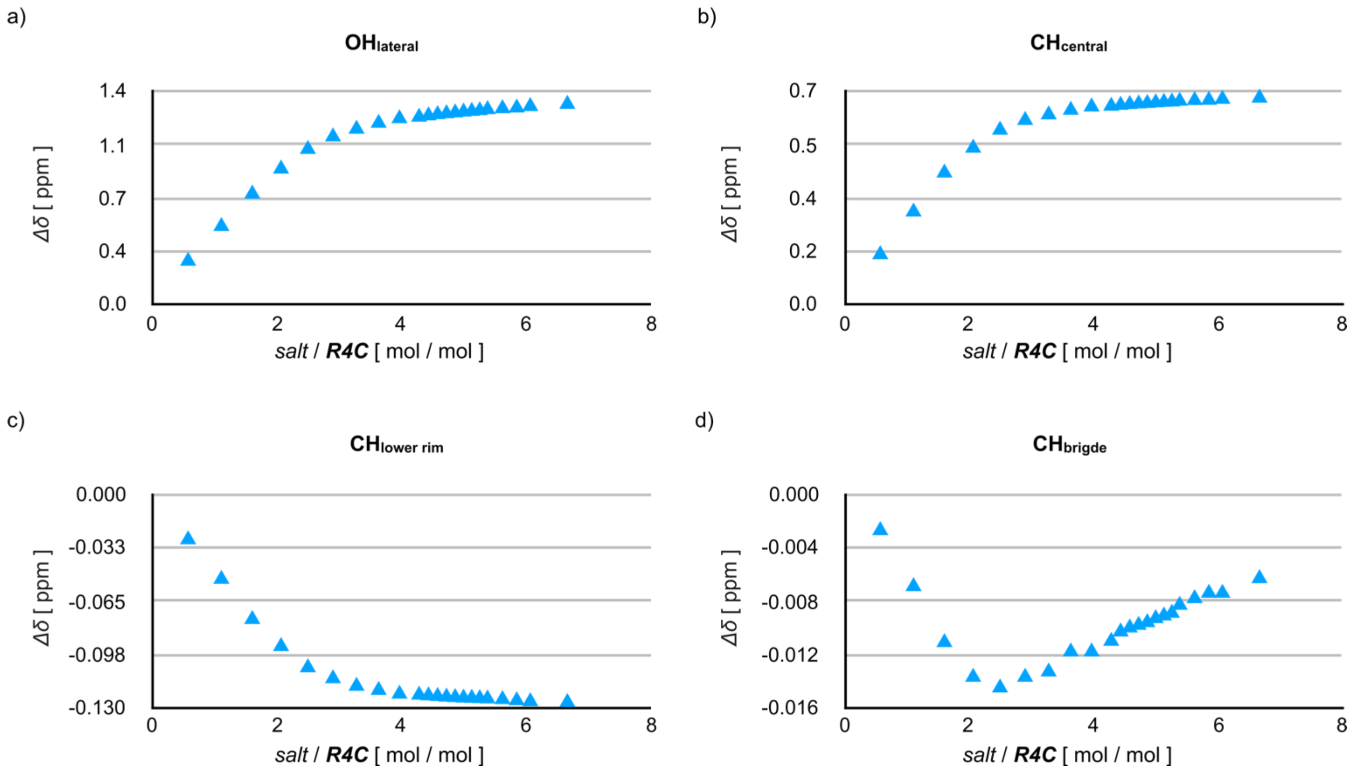

Figure S38. ^{1}H NMR titration curves for titration of **R4C** (C = 5.0 mM) with titrant **R4C** (C = 5.0 mM) + **Pen_4NCl** (C = 75 mM). ^{1}H NMR chemical shifts change for: (a) $OH_{lateral}$; (b) $OH_{central}$; (c) $CH_{lowr\ rim}$; (d) CH_{bridge} (400 MHz, 303 K, THF-d_8).

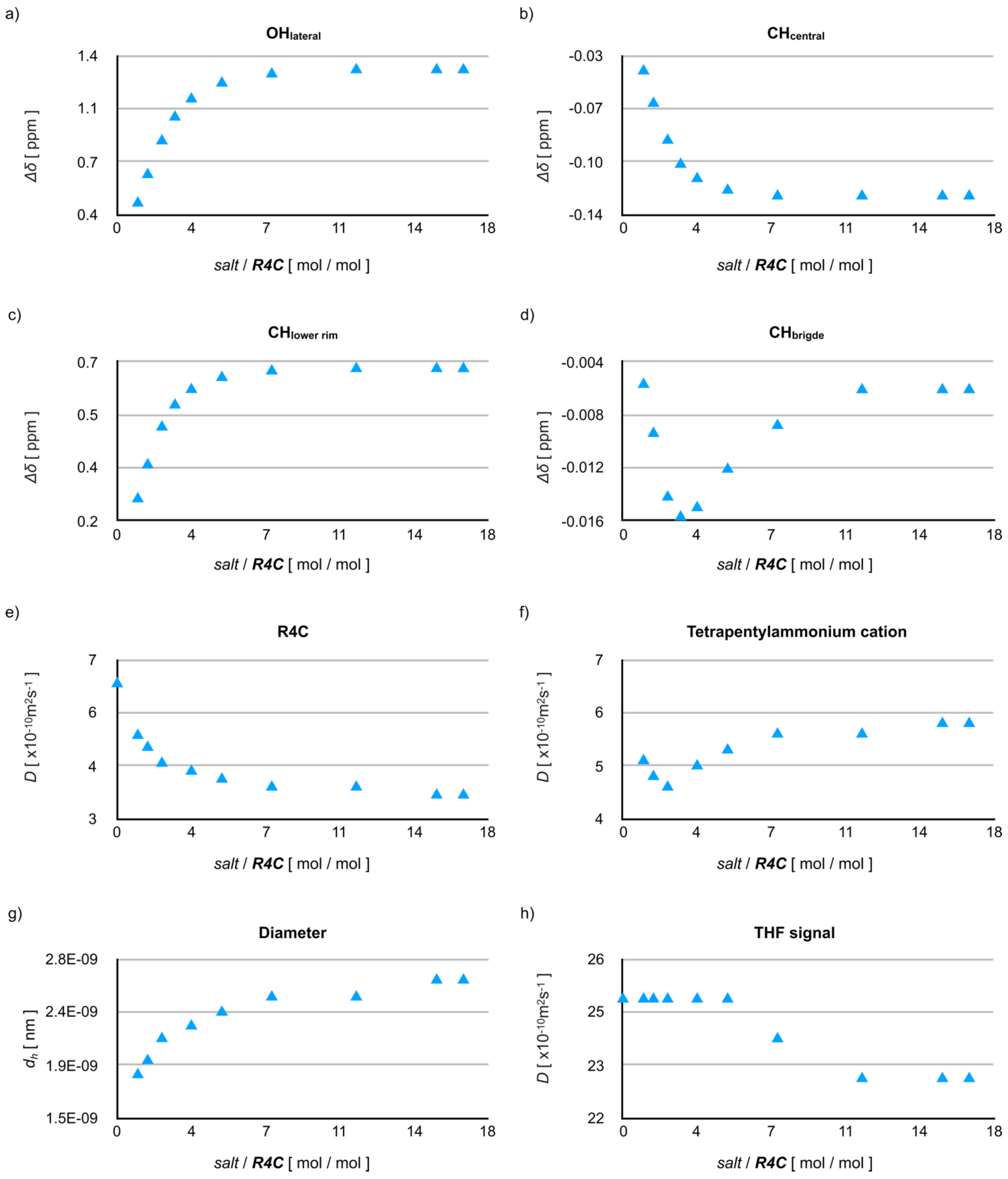

Figure S39. DOSY titration curves for titration of **R4C** (C = 2.5 mM) with titrant **R4C** (C = 2.5 mM) + **Pen_4NCl** (C = 65.6 mM). ^{1}H NMR chemical shift changes for: (a) $OH_{lateral}$; (b) $OH_{central}$; (c) $CH_{lower\ rim}$; (d) CH_{bridge}. Diffusion coefficient changes: for (e) **R4C;** (f) **Pen_4NCl**; (g) diameter of complex; (h) THF (600 MHz, 303 K, THF-d_8).

R4C	Pen_4N^+	Salt / M [mol / mol]
6.4		0
5.1	5.1	1
4.8	4.8	1.48
4.4	4.6	2.17
4.2	5	3.6
4	5.3	5.08
3.8	5.6	7.5
3.8	5.6	11.6
3.6	5.8	15.5
3.6	5.8	16.8

Table S7. Data for DOSY titration (**R4C** (C = 2.5 mM) with titrant **R4C** (C = 2.5 mM) + **Pen_4NCl** (C = 65.6 mM)).

7.3 Titration of resorcin[4]arene (R4C) with tetrahexylammonium chloride (Hex_4NCl)

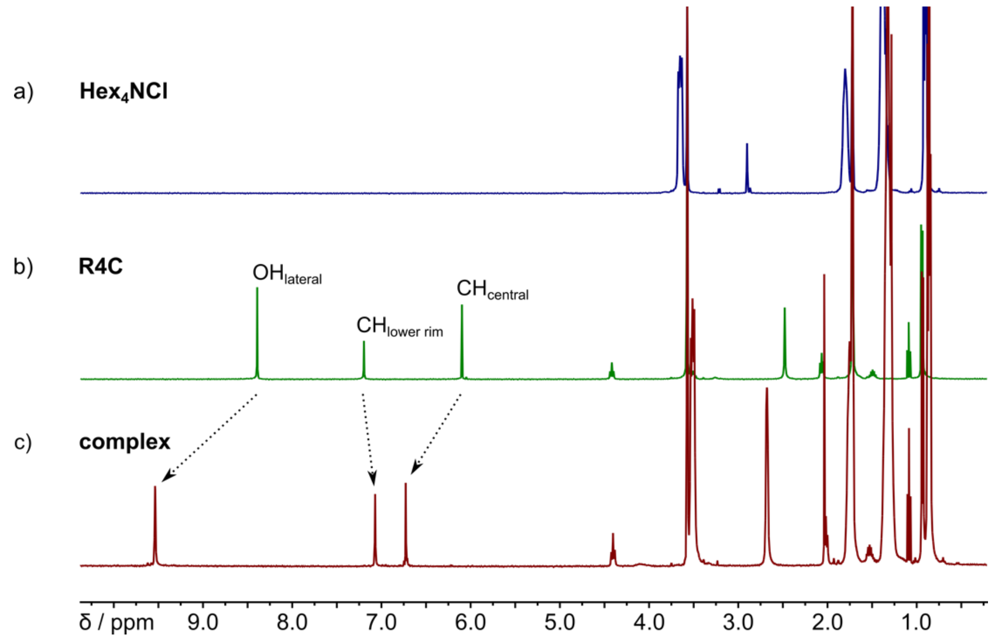

Figure S40. ^{1}H NMR spectra of (a) **Hex_4NCl**; (b) **R4C**; (c) complex of **R4C** and **Hex_4NCl** (400 MHz, 303 K, THF-d_8).

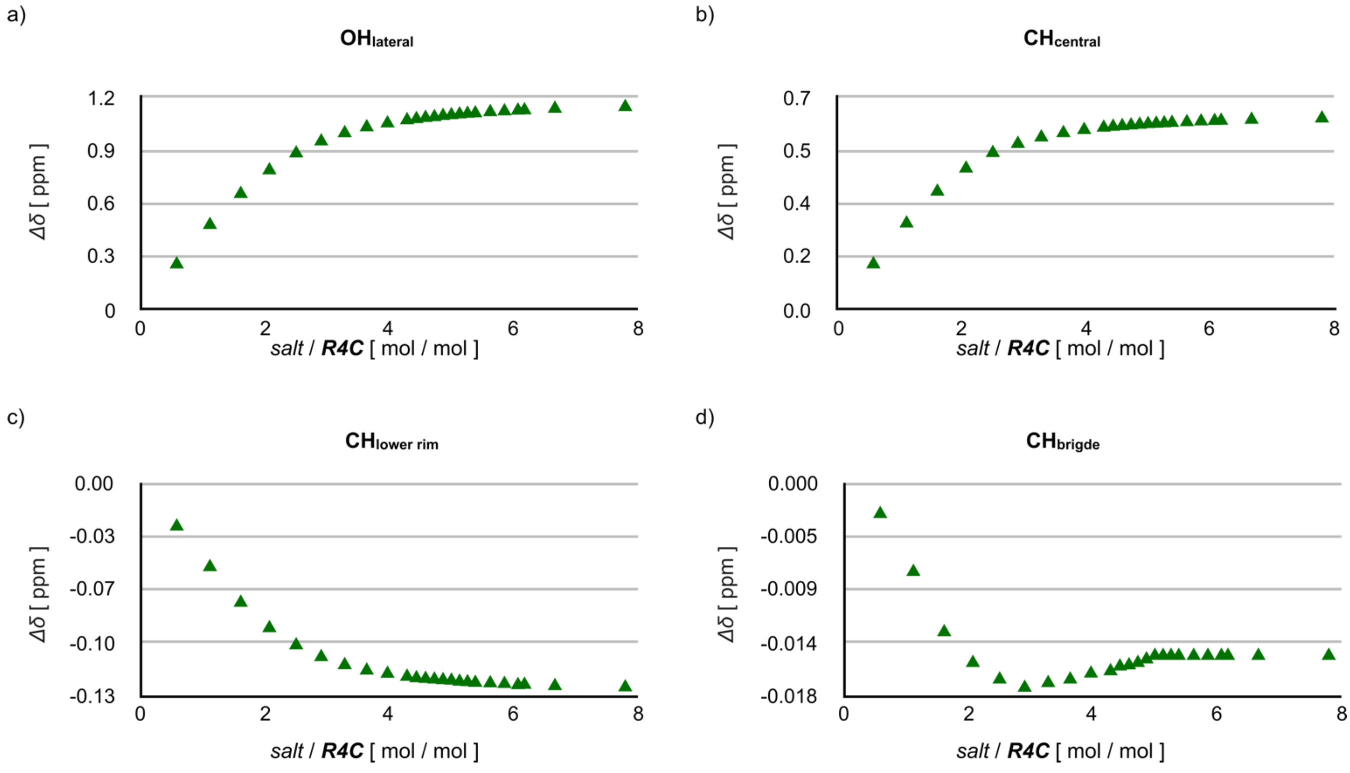

Figure S41. ^{1}H NMR titration curves for titration of **R4C** (C = 5.0 mM) with titrant **R4C** (C = 5.0 mM) + **Hex_4NCl** (C = 75 mM).^{1}H NMR chemical shifts change for: (a) $OH_{lateral}$; (b) $OH_{central}$; (c) $CH_{lowr\ rim}$; (d) CH_{bridge} (400 MHz, 303 K, THF-d_8).

7.4 Titration of resorcin[4]arene (R4C) with tetraoctylammonium chloride (Oct_4NCl)

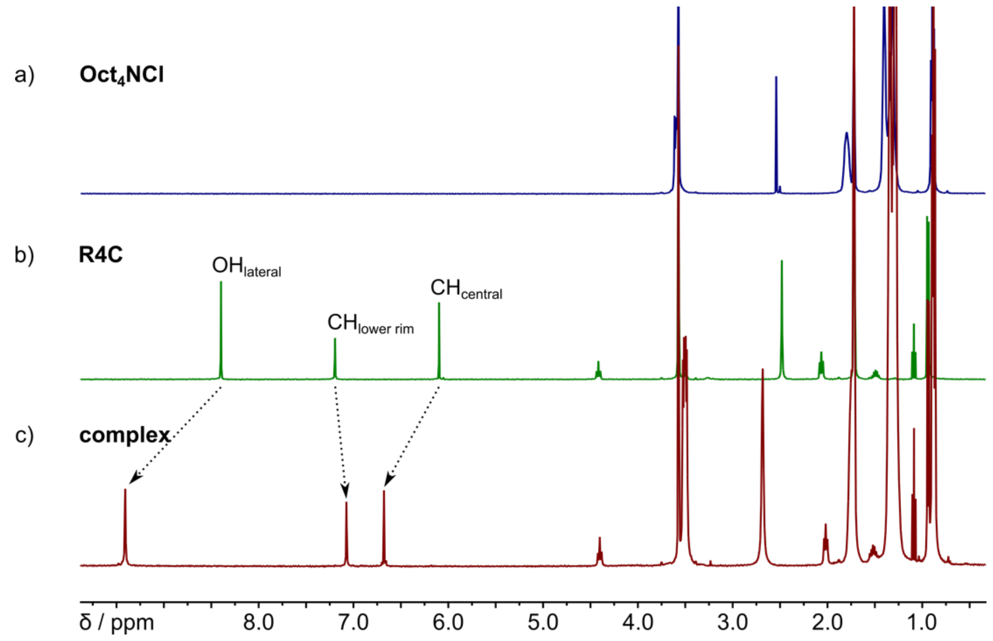

Figure S42. ^{1}H NMR spectra of (a) **Oct_4NCl**; (b) **R4C**; (c) complex of **R4C** and **Oct_4NCl** (400 MHz, 303 K, THF-d_8).

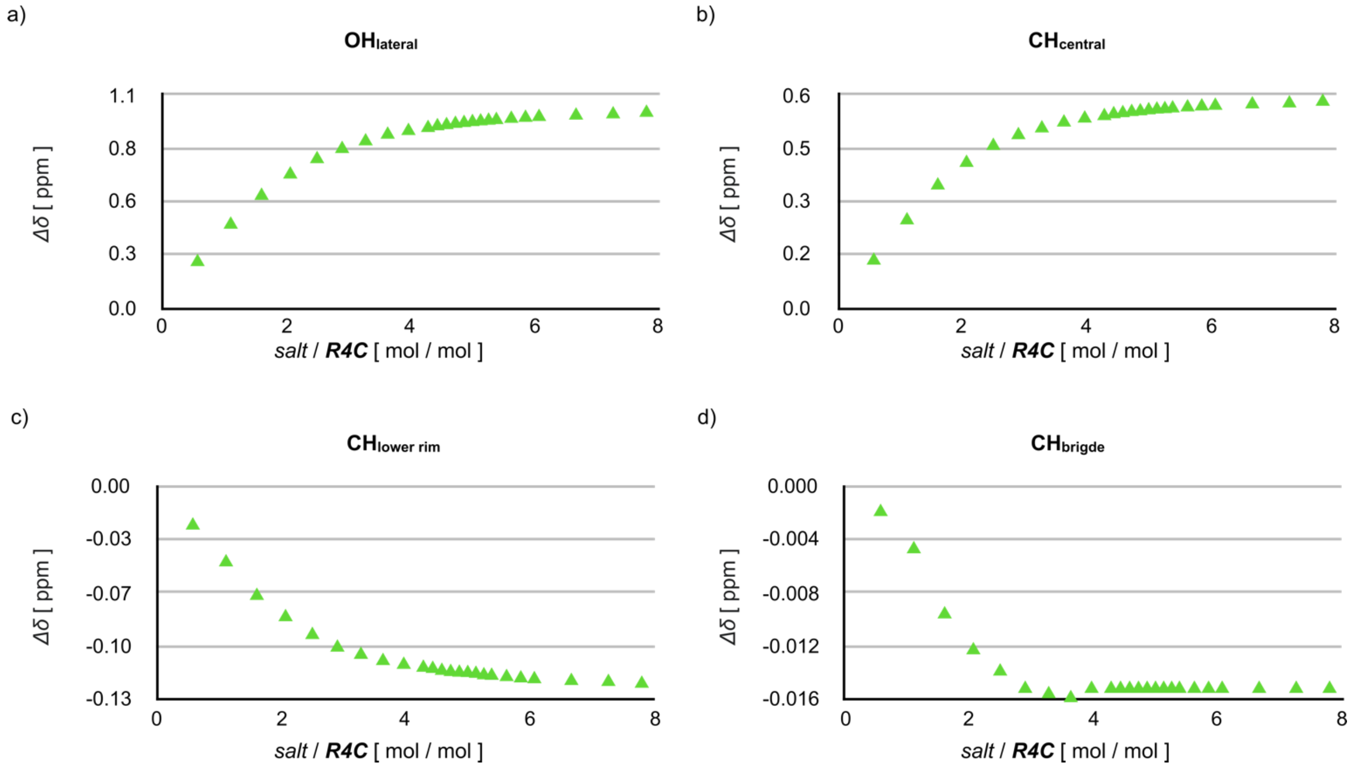

Figure S43. ^{1}H NMR titration curves for titration of **R4C** (C = 5.0 mM) with titrant **R4C** (C = 5.0 mM) + **Oct_4NCl** (C = 75 mM).^{1}H NMR chemical shifts change for: (a) $OH_{lateral}$; (b) $OH_{central}$; (c) $CH_{lowr\ rim}$; (d) CH_{bridge} (400 MHz, 303 K, THF-d_8).

7.5 Titration of resorcin[4]arene (R4C) with tetrabutylammonium bromide (But_4NBr)

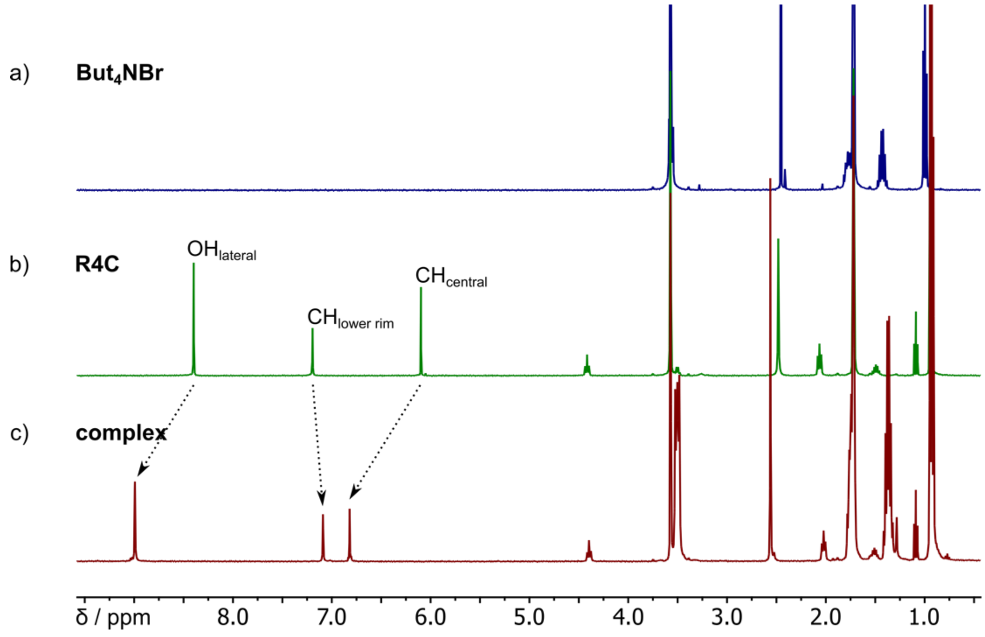

Figure S44. ^{1}H NMR spectra of (a) **But_4NBr**; (b) **R4C**; (c) complex of **R4C** and **But_4NBr** (400 MHz, 303 K, THF-d_8).

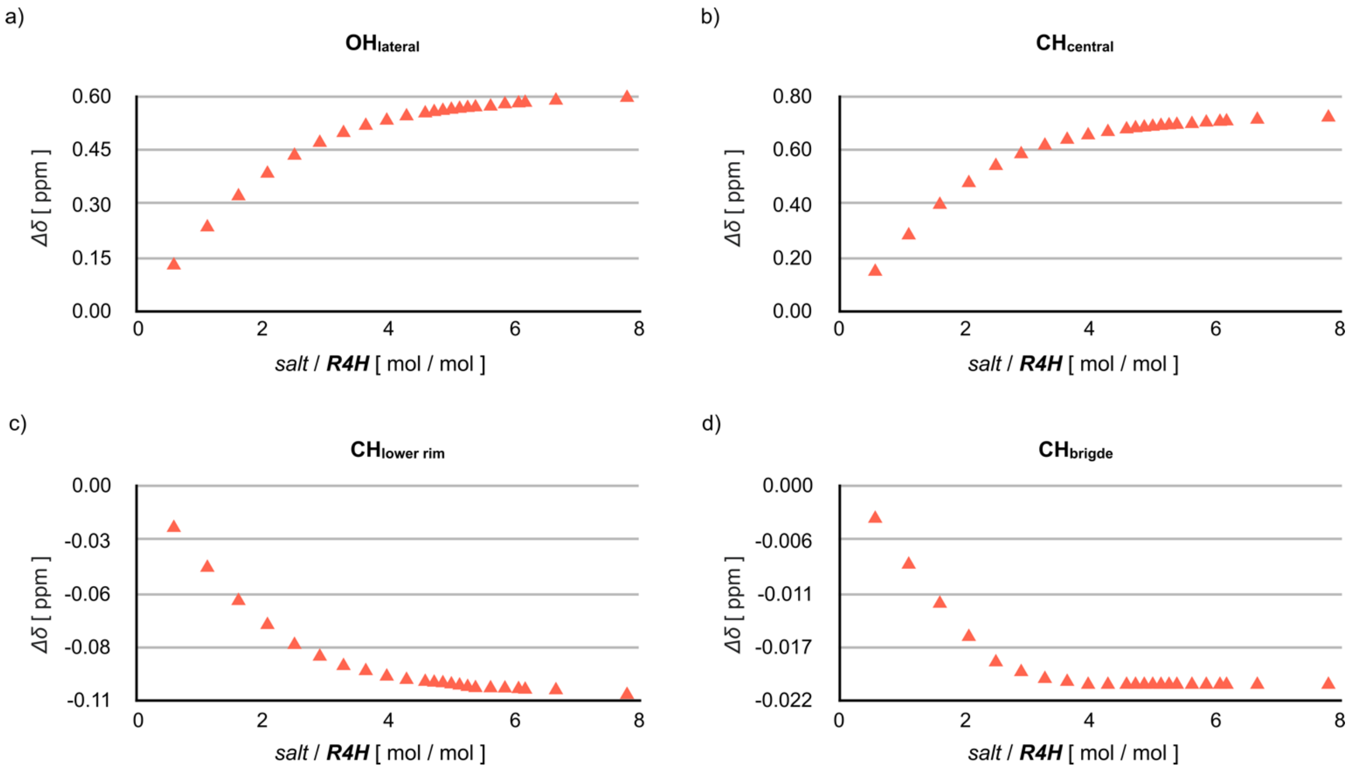

Figure S45. ^{1}H NMR titration curves for titration of **R4C** (C = 5.0 mM) with titrant **R4C** (C = 5.0 mM) + **But_4NBr** (C = 75 mM).^{1}H NMR chemical shifts change for: (a) $OH_{lateral}$; (b) $OH_{central}$; (c) $CH_{lowr\ rim}$; (d) CH_{bridge} (400 MHz, 303 K, THF-d_8).

7.6 Titration of resorcin[4]arene (R4C) with tetrapentylammonium bromide (Pen_4NBr)

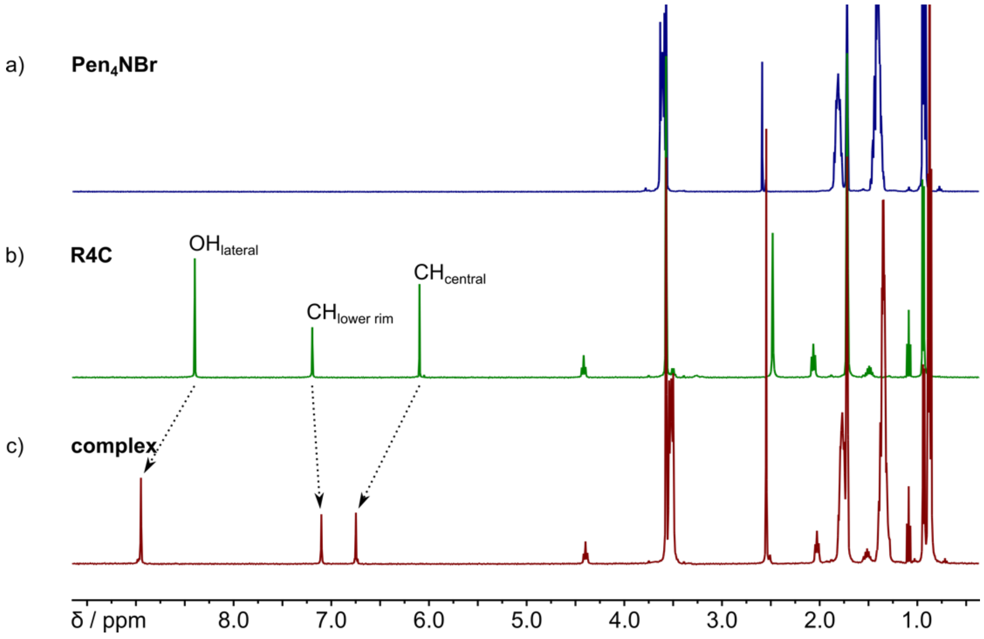

Figure S46. ^{1}H NMR spectra of (a) **Pen_4NBr**; (b) **R4C**; (c) complex of **R4C** and **Pen_4NBr** (400 MHz, 303 K, THF-d_8).

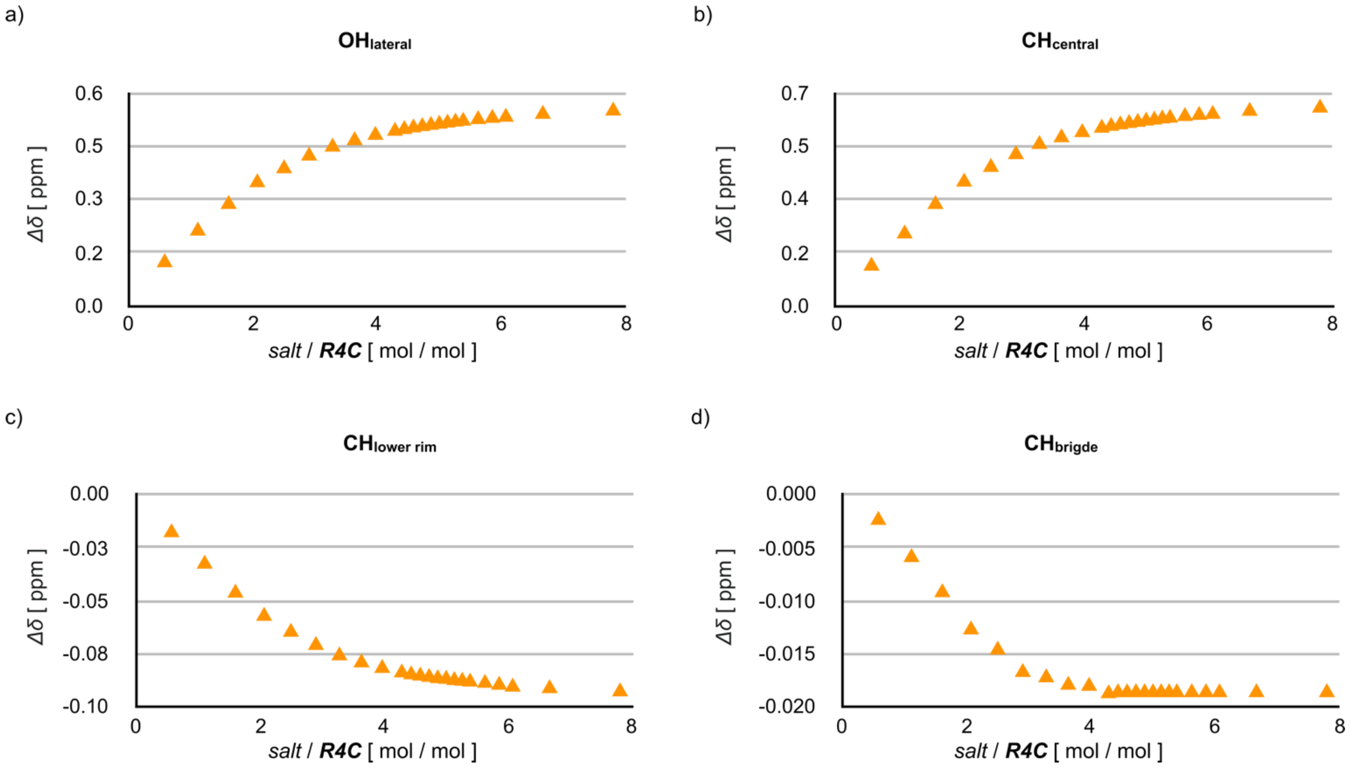

Figure S47. ^{1}H NMR titration curves for titration of **R4C** (C = 5.0 mM) with titrant **R4C** (C = 5.0 mM) + **Pen_4NBr** (C = 75 mM).^{1}H NMR chemical shifts change for: (a) $OH_{lateral}$; (b) $OH_{central}$; (c) $CH_{lowr\ rim}$; (d) CH_{bridge} (400 MHz, 303 K, THF-d_8).

7.7 Titration of resorcin[4]arene (R4C) with tetrahexylammonium bromide (Hex_4NBr)

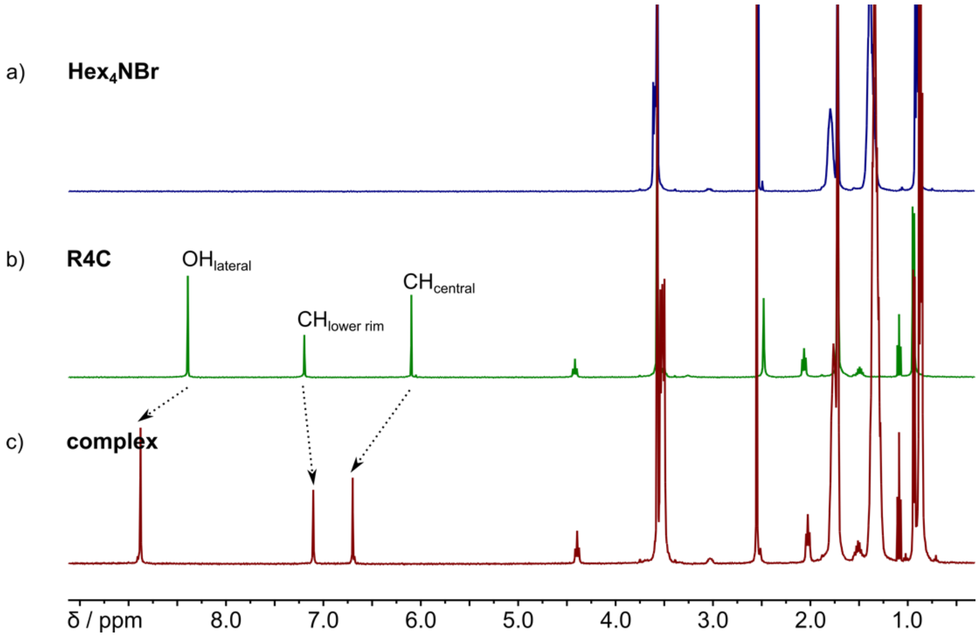

Figure S48. ^{1}H NMR spectra of (a) **Hex_4NBr**; (b) **R4C**; (c) complex of **R4C** and **Hex_4NBr** (400 MHz, 303 K, THF-d_8).

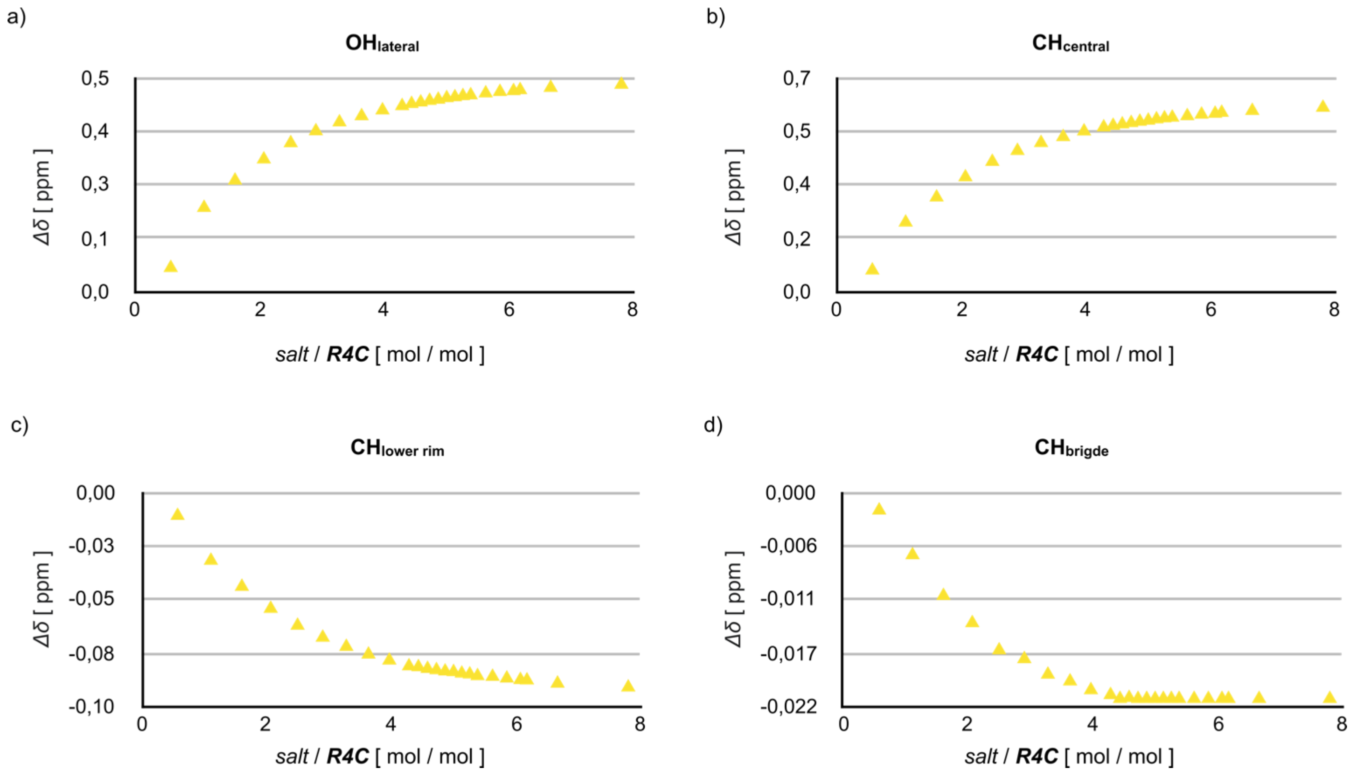

Figure S49. ^{1}H NMR titration curves for titration of **R4C** (C = 5.0 mM) with titrant **R4C** (C = 5.0 mM) + **Hex_4NBr** (C = 75 mM).^{1}H NMR chemical shifts change for: (a) $OH_{lateral}$; (b) $OH_{central}$; (c) $CH_{lowr\ rim}$; (d) CH_{bridge} (400 MHz, 303 K, THF-d_8).

7.8 Titration of resorcin[4]arene (R4C) with tetraoctylammonium bromide (Oct_4NBr)

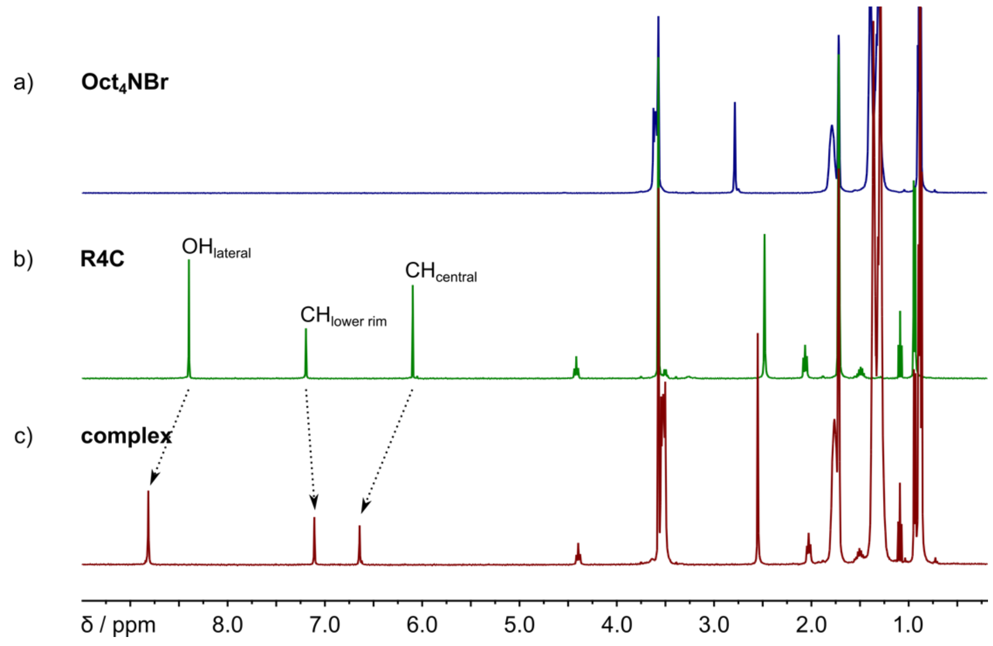

Figure S50. ^{1}H NMR spectra of (a) **Oct_4NBr**; (b) **R4C**; (c) complex of **R4C** and **Oct_4NBr** (400 MHz, 303 K, THF-d_8).

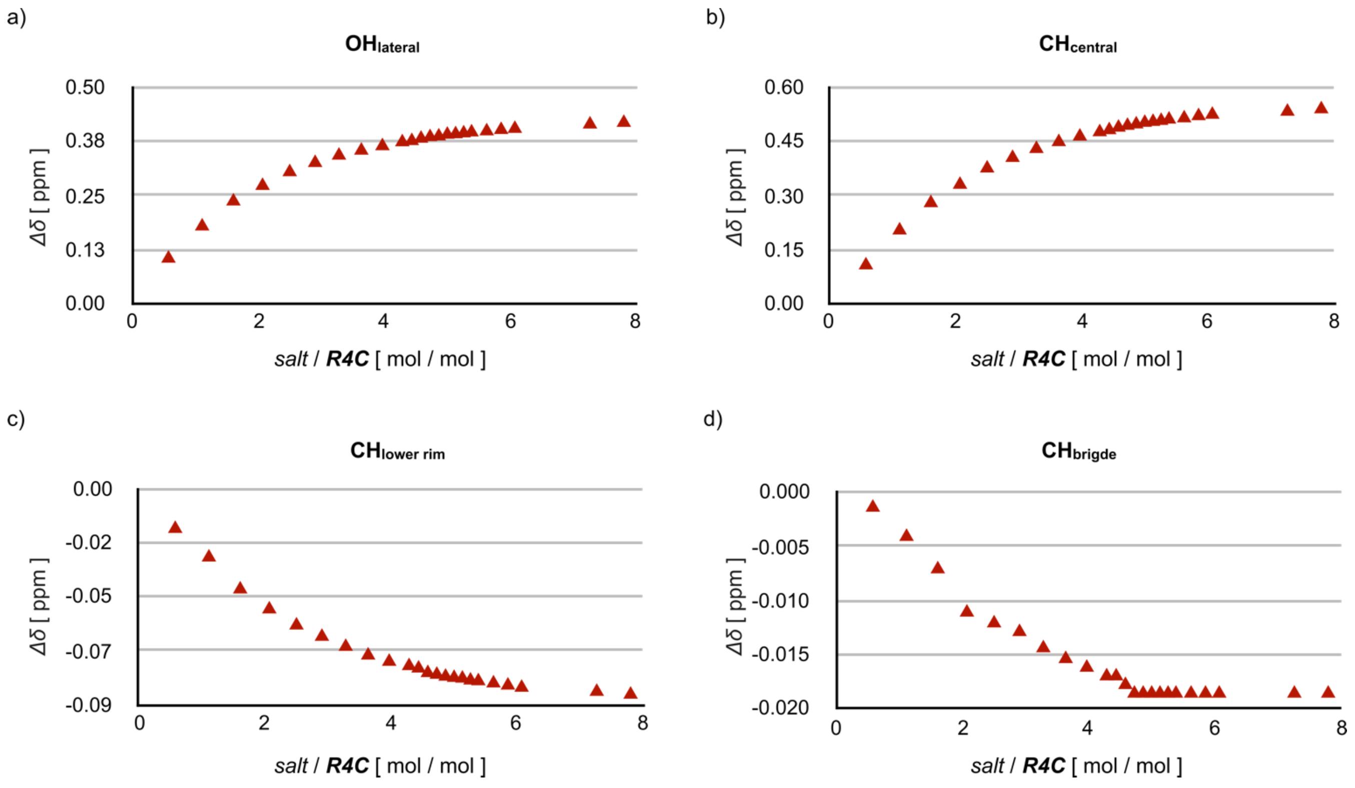

Figure S51. ^{1}H NMR titration curves for titration of **R4C** (C = 5.0 mM) with titrant **R4C** (C = 5.0 mM) + **Oct_4NBr** (C = 75 m).^{1}H NMR chemical shifts change for: (a) $OH_{lateral}$; (b) $OH_{central}$; (c) $CH_{lowr\ rim}$; (d) CH_{bridge} (400 MHz, 303 K, THF-d_8).

7.9 Titration of resorcin[4]arene (R4C) with tetradecylammonium bromide (Dec_4NBr)

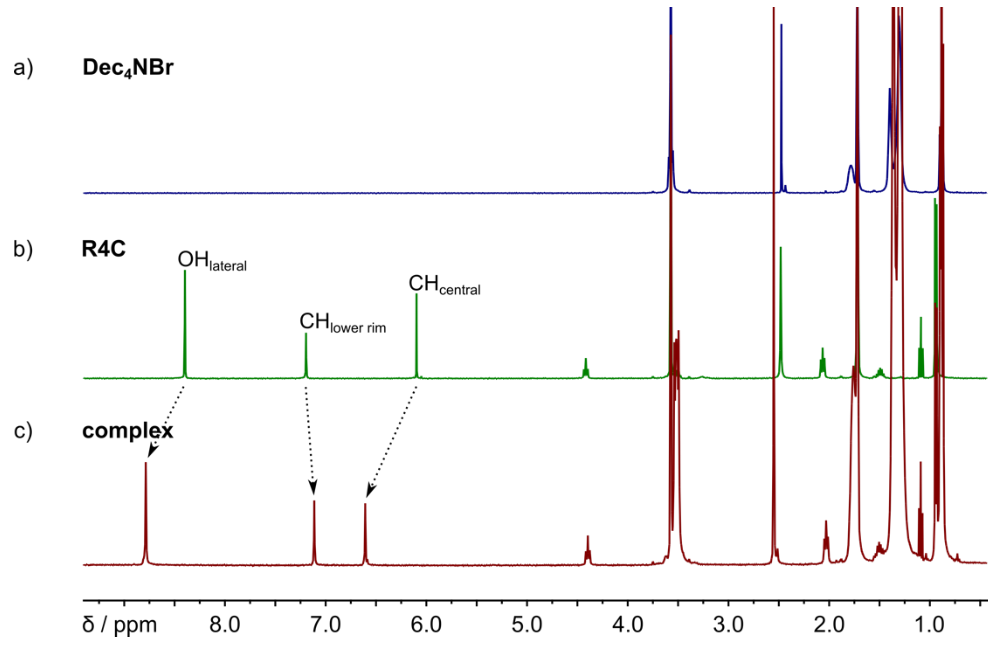

Figure S52. ^{1}H NMR spectra of (a) **Dec_4NBr**; (b) **R4C**; (c) complex of **R4C** and **Dec_4NBr** (400 MHz, 303 K, THF-d_8).

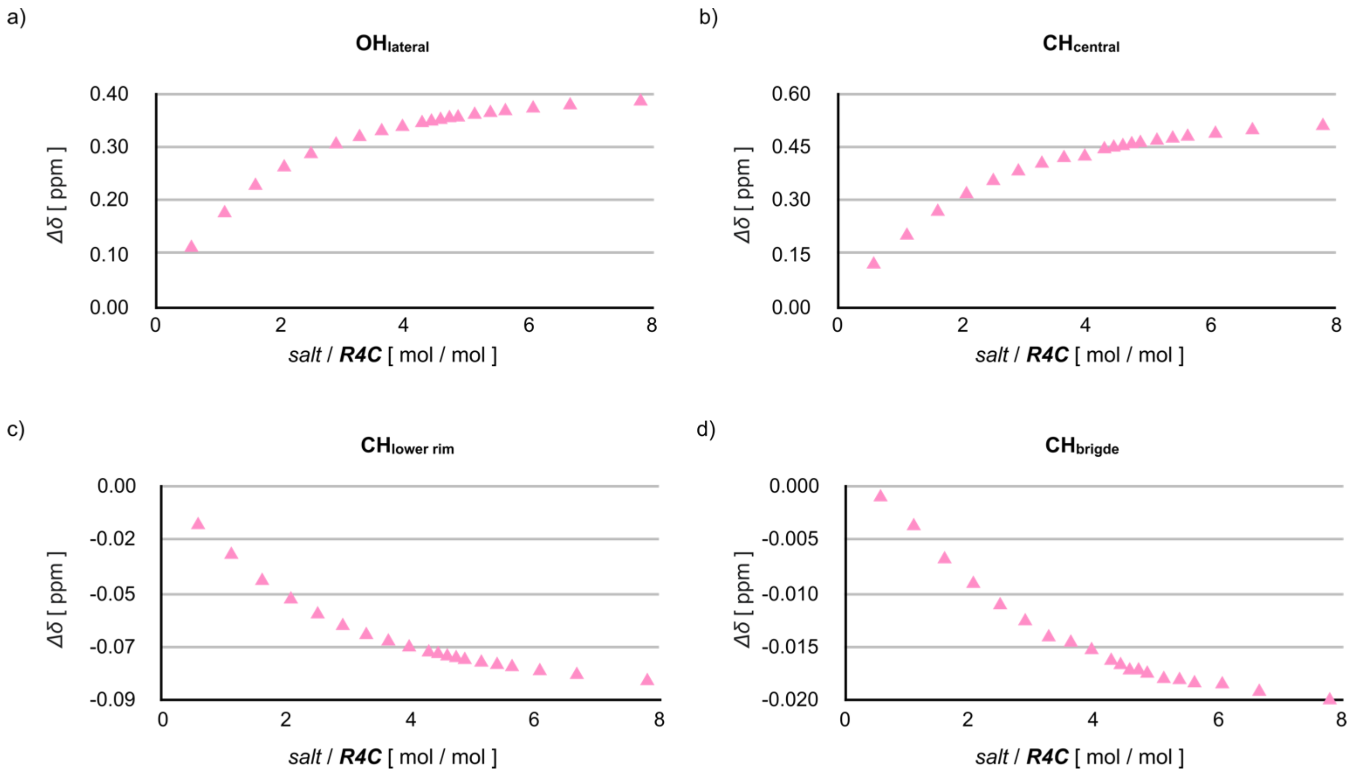

Figure S53. ^{1}H NMR titration curves for titration of **R4C** (C = 5.0 mM) with titrant **R4C** (C = 5.0 mM) + **Dec_4NBr** (C = 75 mM). ^{1}H NMR chemical shifts change for: (a) $OH_{lateral}$; (b) $OH_{central}$; (c) $CH_{lowr\ rim}$; (d) CH_{bridge} (400 MHz, 303 K, THF-d_8).

8. Titrations of Resorcin[4]arene (R4H) in THF

8.1 Titration of resorcin[4]arene (R4H) with tetrapenylammonium chloride (Pen_4NCl)

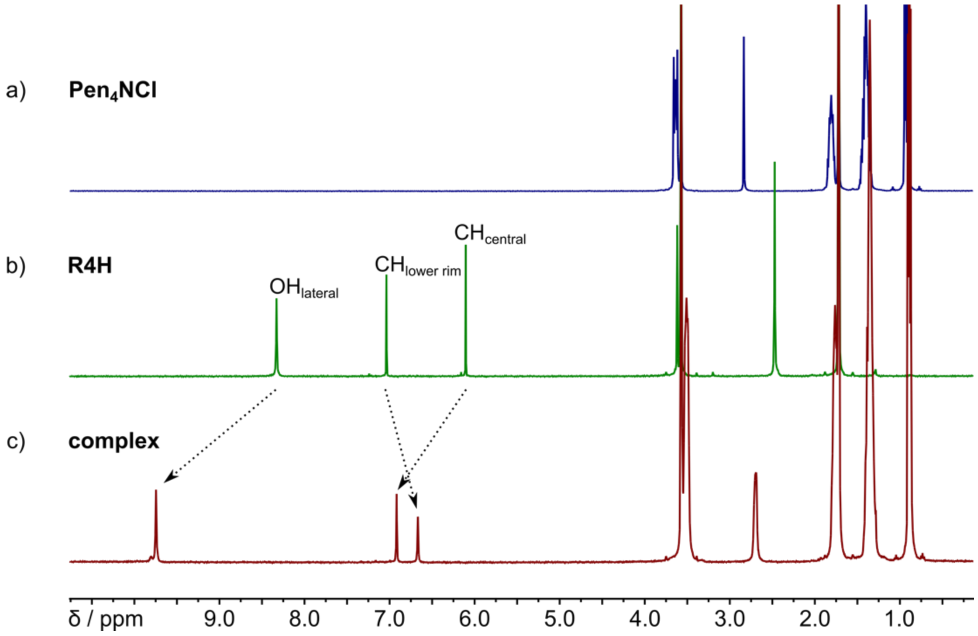

Figure S54. ^{1}H NMR spectra of (a) **Pen_4NCl**; (b) **R4H**; (c) complex of **R4H** and **Pen_4NCl** (400 MHz, 303 K, THF-d_8).

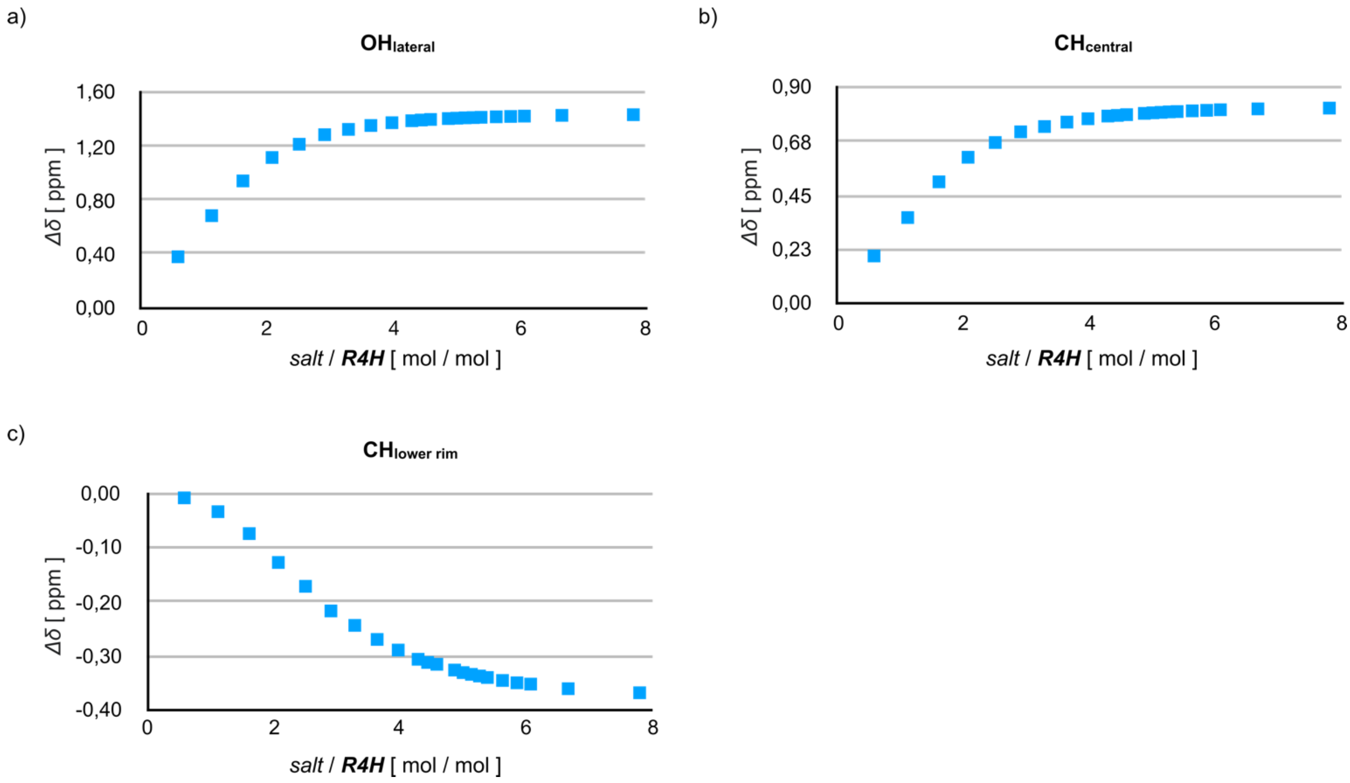

Figure S55. ^{1}H NMR titration curves for titration of **R4H** (C = 5.0 mM) with titrant **R4H** (C = 5.0 mM) + **Pen_4NCl** (C = 75 mM).^{1}H NMR chemical shifts change for: (a) $OH_{lateral}$; (b) $OH_{central}$; (c) $CH_{lowr\ rim}$; (d) $CH_{2\ bridge}$ (400 MHz, 303 K, THF-d_8).

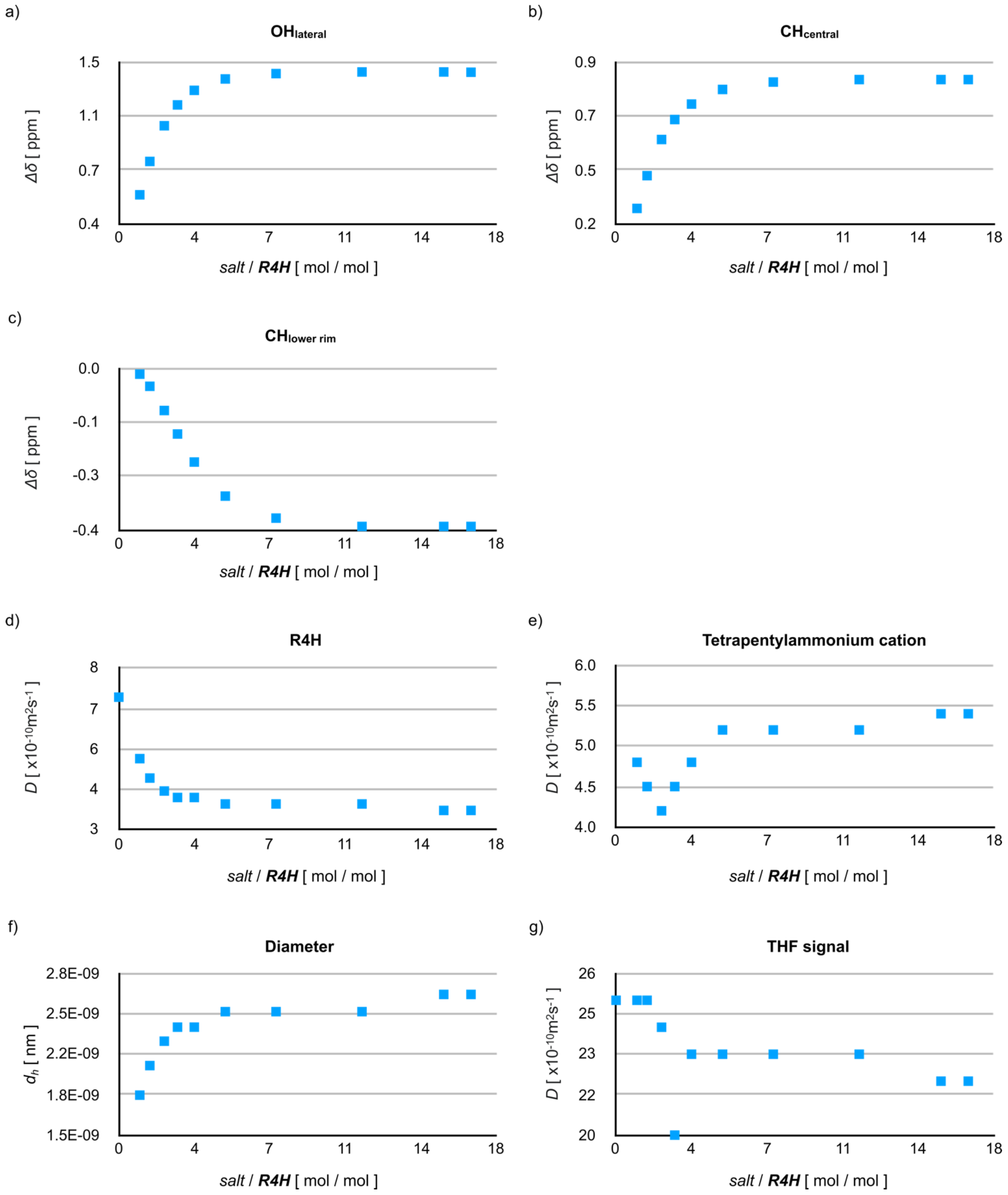

Figure S56. DOSY titration curves for titration of **R4H** (C = 2.5 mM) with titrant **R4H** (C = 2.5 mM) + **Pen$_4$NCl** (C = 65.6 mM). ^{1}H NMR chemical shift changes for: (a) OH$_{lateral}$; (b) OH$_{central}$; (c) CH$_{lower\ rim}$. Diffusion coefficient changes: for (d) **R4H;** (e) **Pen$_4$NCl**; (f) diameter of complex; (g) THF (600 MHz, 303 K, THF-d$_8$).

R4H	Pen_4N^+	Salt / M [mol / mol]
7.1		0
5.2	4.8	1
4.6	4.5	1.48
4.2	4.2	2.17
4	4.5	2.8
4	4.8	3.6
3.8	5.2	5.08
3.8	5.2	7.5
3.8	5.2	11.6
3.6	5.4	15.5
3.6	5.4	16.8

Table S8. Data for DOSY titration **R4H** (C = 2.5 mM) with titrant **R4H** (C = 2.5 mM) + **Pen_4NCl** (C = 65.6 mM).

8.2 Titration of resorcin[4]arene (R4H) with tetrahexylammonium chloride (Hex_4NCl)

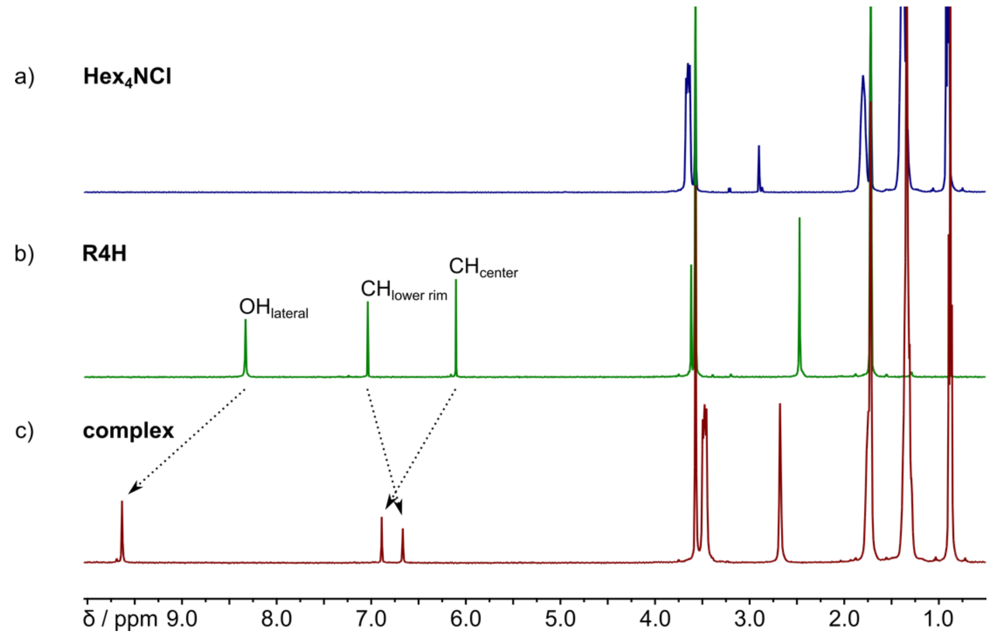

Figure S57. ^{1}H NMR spectra of (a) **Hex_4NCl**; (b) **R4H**; (c) complex of **R4H** and **Hex_4NCl** (400 MHz, 303 K, THF-d_8).

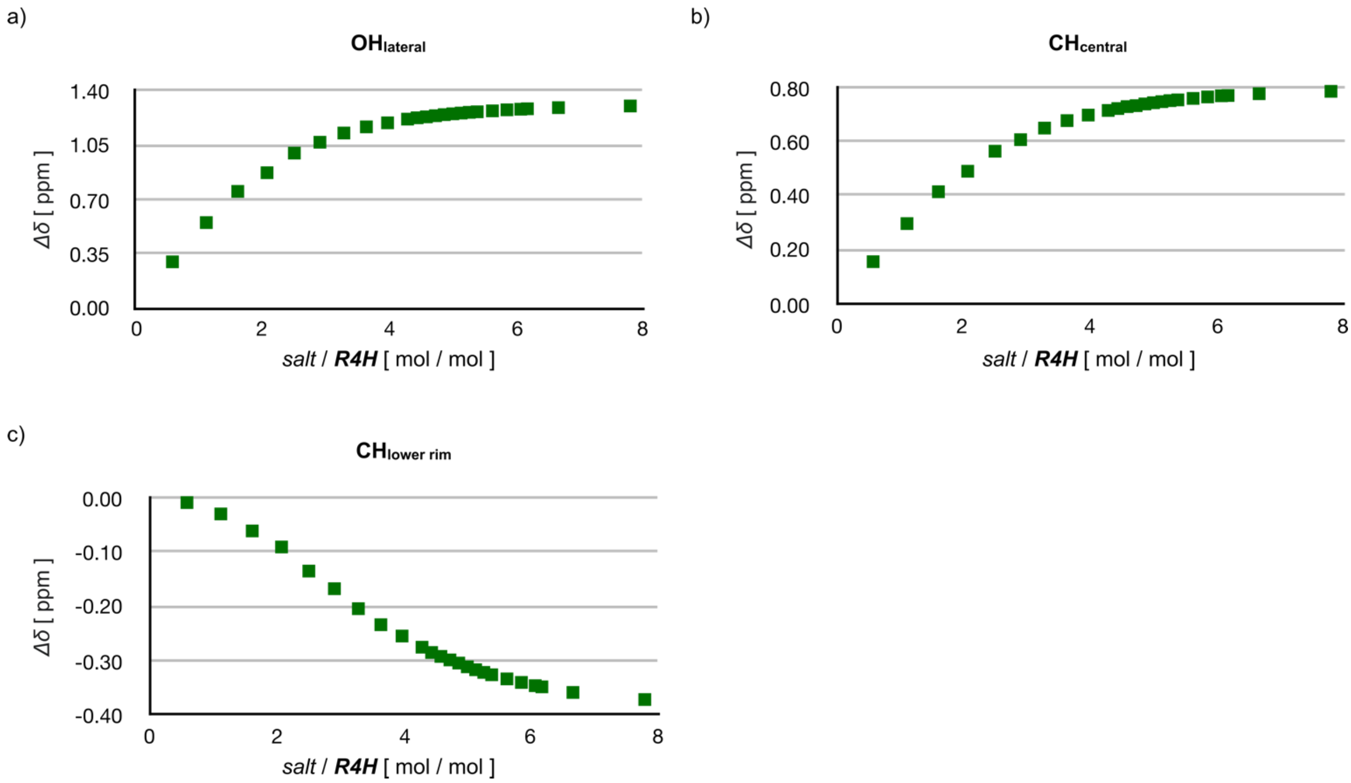

Figure S58. ^{1}H NMR titration curves for titration of **R4H** (C = 5.0 mM) with titrant **R4H** (C = 5.0 mM) + **Hex_4NCl** (C = 75 mM).^{1}H NMR chemical shifts change for: (a) $OH_{lateral}$; (b) $OH_{central}$; (c) $CH_{lowr\ rim}$; (d) $CH_{2\ bridge}$ (400 MHz, 303 K, THF-d_8).

8.3 Titration of resorcin[4]arene (R4H) with tetraoctylammonium chloride (Oct_4NCl)

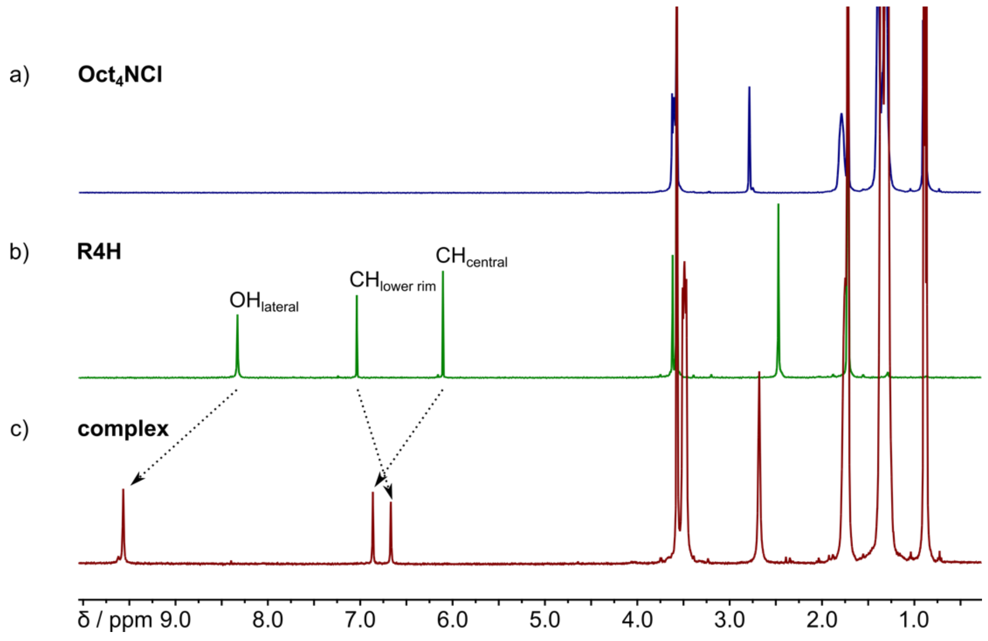

Figure S59. ^{1}H NMR spectra of (a) **Oct_4NCl**; (b) **R4H**; (c) complex of **R4H** and **Oct_4NCl** (400 MHz, 303 K, THF-d_8).

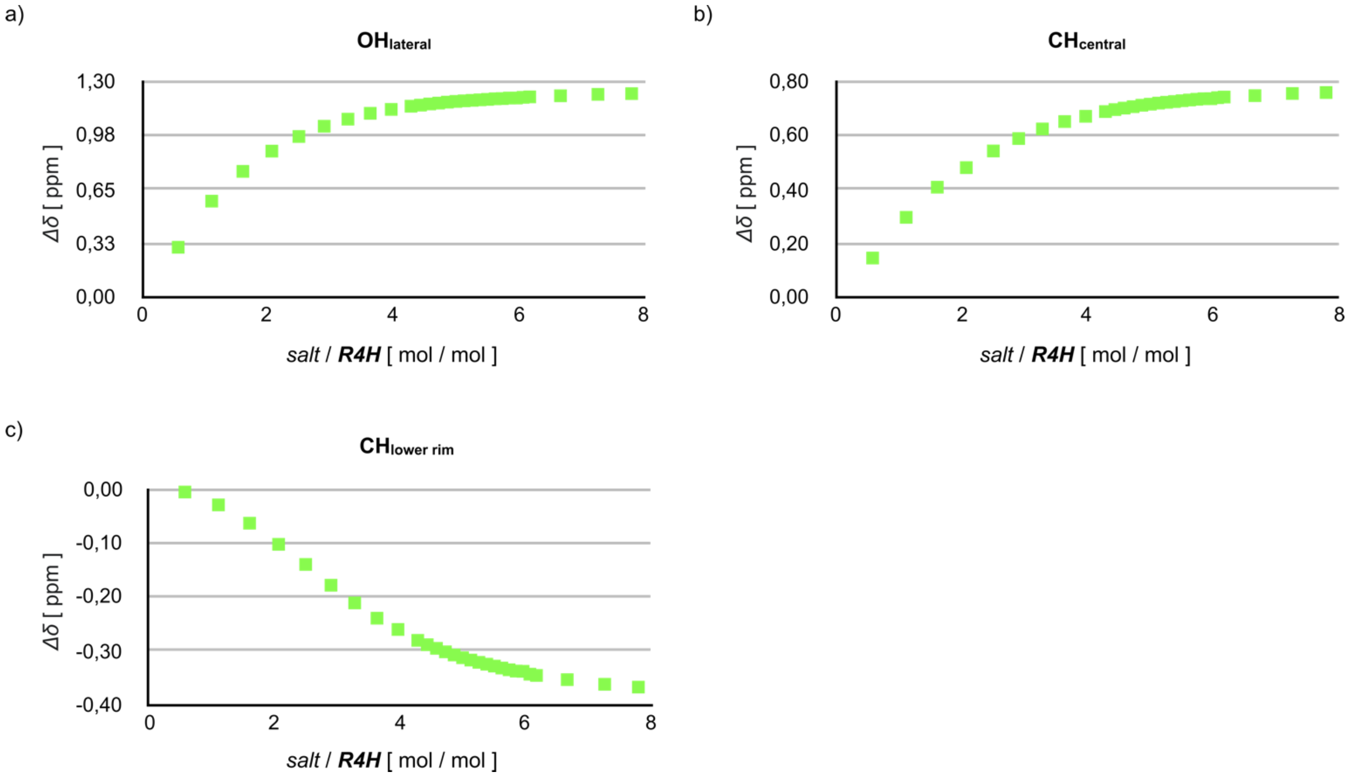

Figure S60. ^{1}H NMR titration curves for titration of **R4H** (C = 5.0 mM) with titrant **R4H** (C = 5.0 mM) + **Oct_4NCl** (C = 75 mM).^{1}H NMR chemical shifts change for: (a) $OH_{lateral}$; (b) $OH_{central}$; (c) $CH_{lowr\ rim}$; (d) $CH_{2\ bridge}$ (400 MHz, 303 K, THF-d_8).

8.4 Titration of resorcin[4]arene (R4H) with tetrabutylammonium bromide (But$_4$NBr)

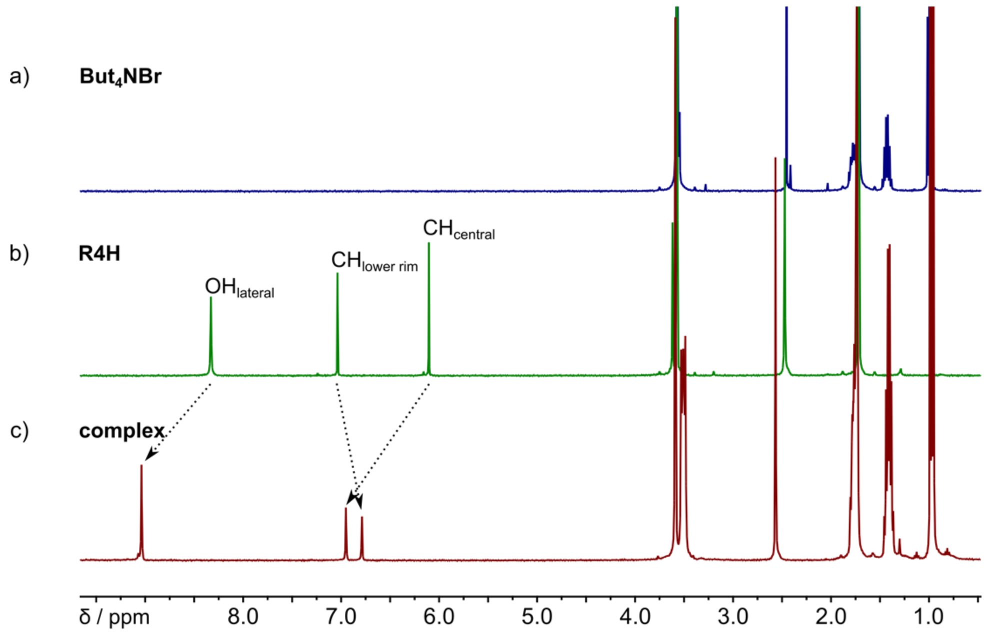

Figure S61. ^{1}H NMR spectra of (a) **But$_4$NBr**; (b) **R4H**; (c) complex of **R4H** and **But$_4$NBr** (400 MHz, 303 K, THF-d$_8$).

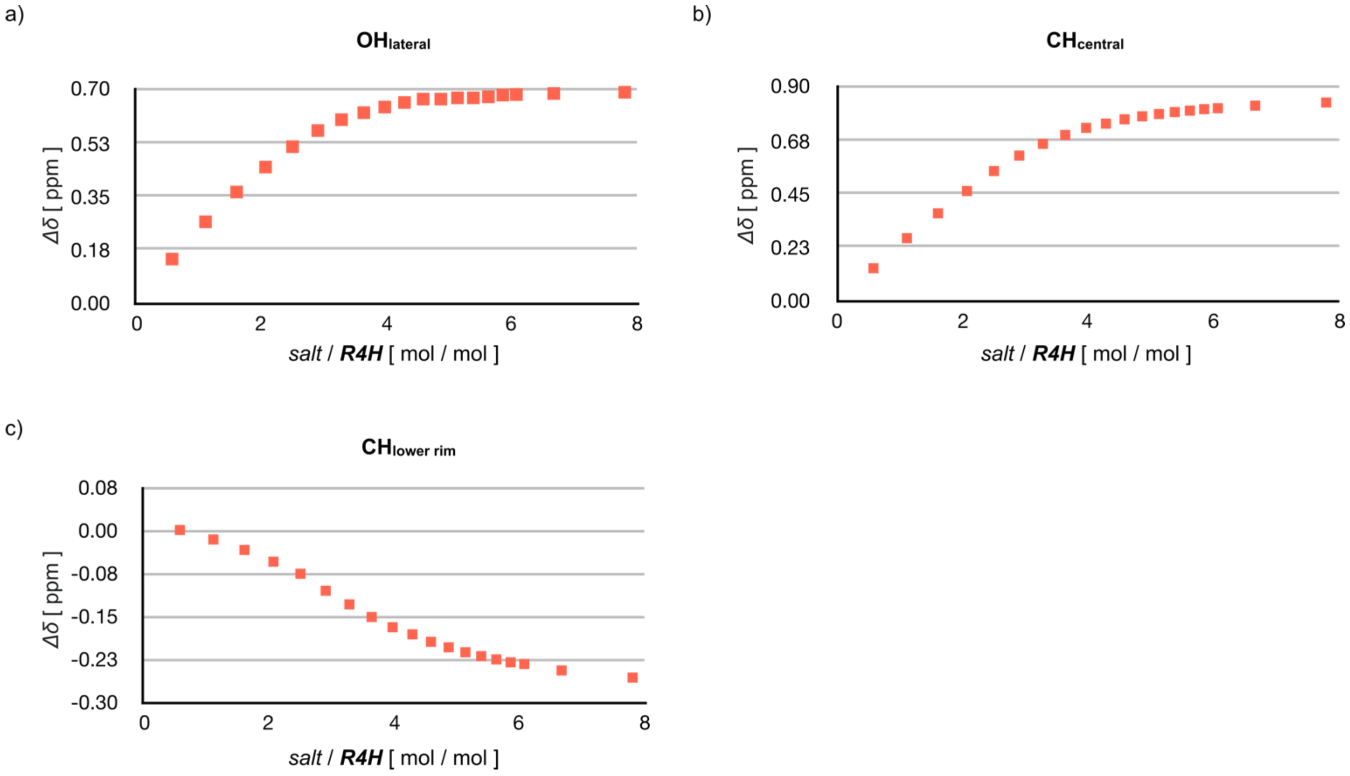

Figure S62. ^{1}H NMR titration curves for titration of **R4H** (C = 5.0 mM) with titrant **R4H** (C = 5.0 mM) + **But$_4$NBr** (C = 75 mM).^{1}H NMR chemical shifts change for: (a) OH$_{lateral}$; (b) OH$_{central}$; (c) CH$_{lowr\ rim}$; (d) CH$_{2\ bridge}$ (400 MHz, 303 K, THF-d$_8$).

8.5 Titration of resorcin[4]arene (R4H) with tetrapentylammonium bromide (Pen_4NBr)

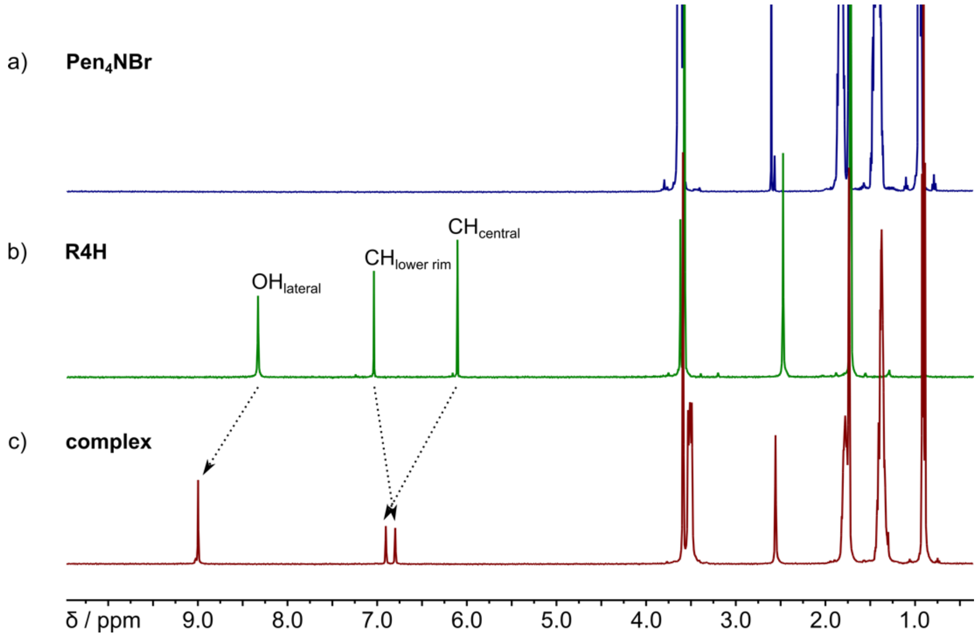

Figure S63. ^{1}H NMR spectra of (a) **Pen_4NBr**; (b) **R4H**; (c) complex of **R4H** and **Pen_4NBr** (400 MHz, 303 K, THF-d_8).

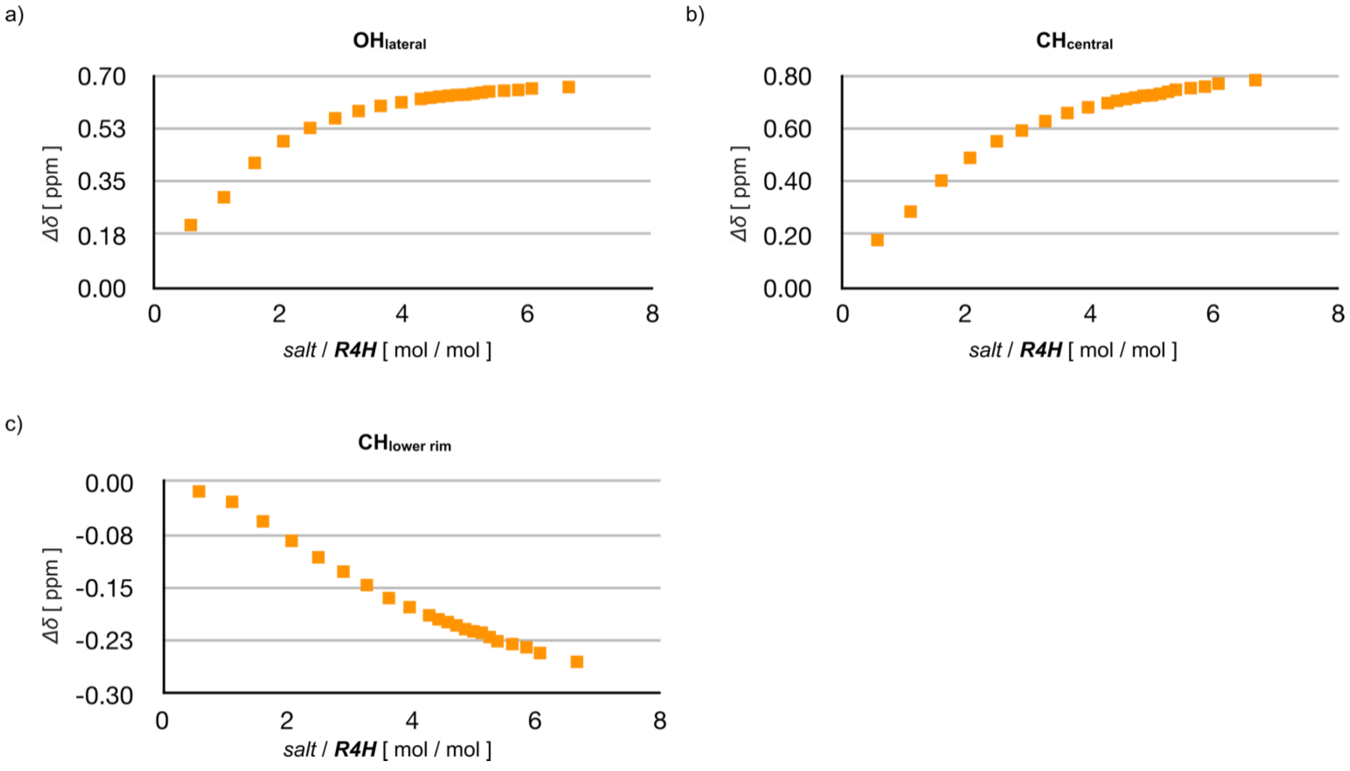

Figure S64. ^{1}H NMR titration curves for titration of **R4H** (C = 5.0 mM) with titrant **R4H** (C = 5.0 mM) + **Pen_4NBr** (C = 75 mM). ^{1}H NMR chemical shifts change for: (a) $OH_{lateral}$; (b) $OH_{central}$; (c) $CH_{lowr\ rim}$; (d) $CH_{2\ bridge}$ (400 MHz, 303 K, THF-d_8).

8.6 Titration of resorcin[4]arene (R4H) with tetrahexylammonium bromide (Hex_4NBr)

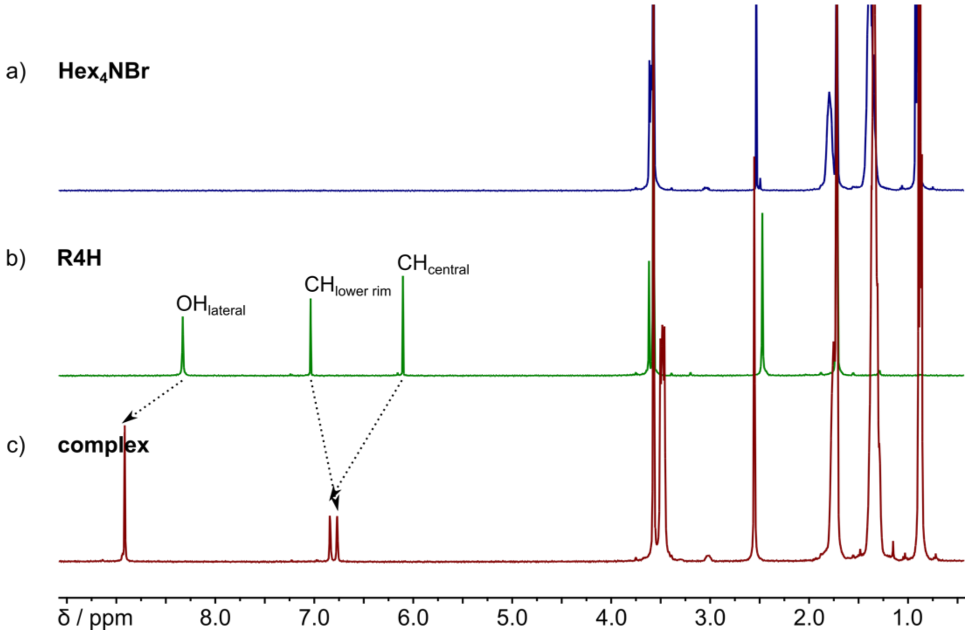

Figure S65. ^{1}H NMR spectra of (a) **Hex_4NBr**; (b) **R4H**; (c) complex of **R4H** and **Hex_4NBr** (400 MHz, 303 K, THF-d_8).

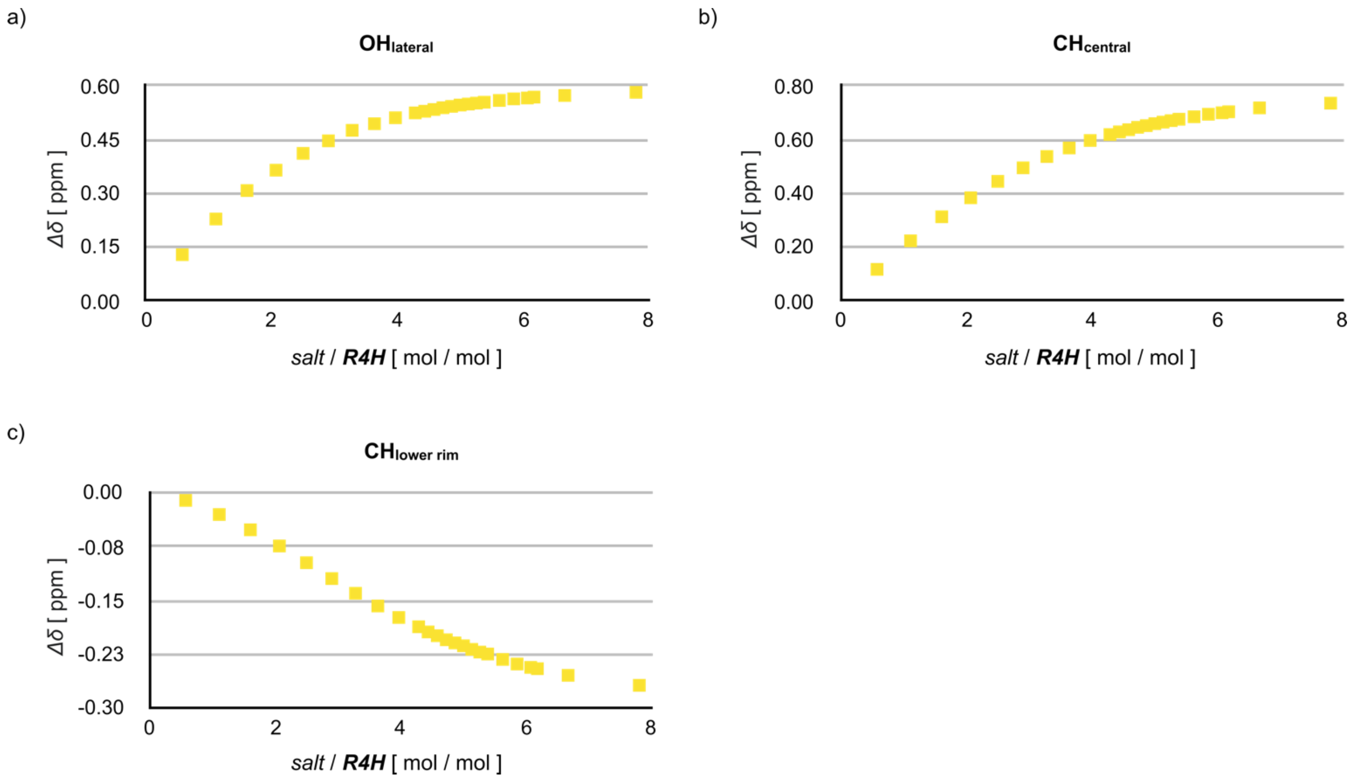

Figure S66. ^{1}H NMR titration curves for titration of **R4H** (C = 5.0 mM) with titrant **R4H** (C = 5.0 mM) + **Hex_4NBr** (C = 75 mM). ^{1}H NMR chemical shifts change for: (a) $OH_{lateral}$; (b) $OH_{central}$; (c) $CH_{lowr\ rim}$; (d) $CH_{2\ bridge}$ (400 MHz, 303 K, THF-d_8).

8.7 Titration of resorcin[4]arene (R4H) with tetraoctylammonium bromide (Oct_4NBr)

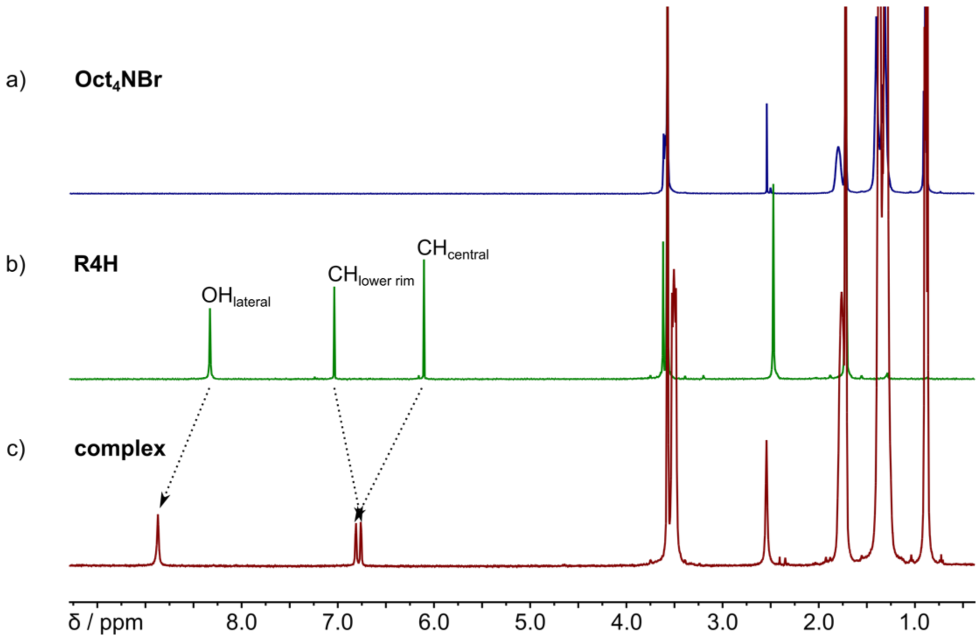

Figure S67. ^{1}H NMR spectra of (a) **Oct_4NBr**; (b) **R4H**; (c) complex of **R4H** and **Oct_4NBr** (400 MHz, 303 K, THF-d_8).

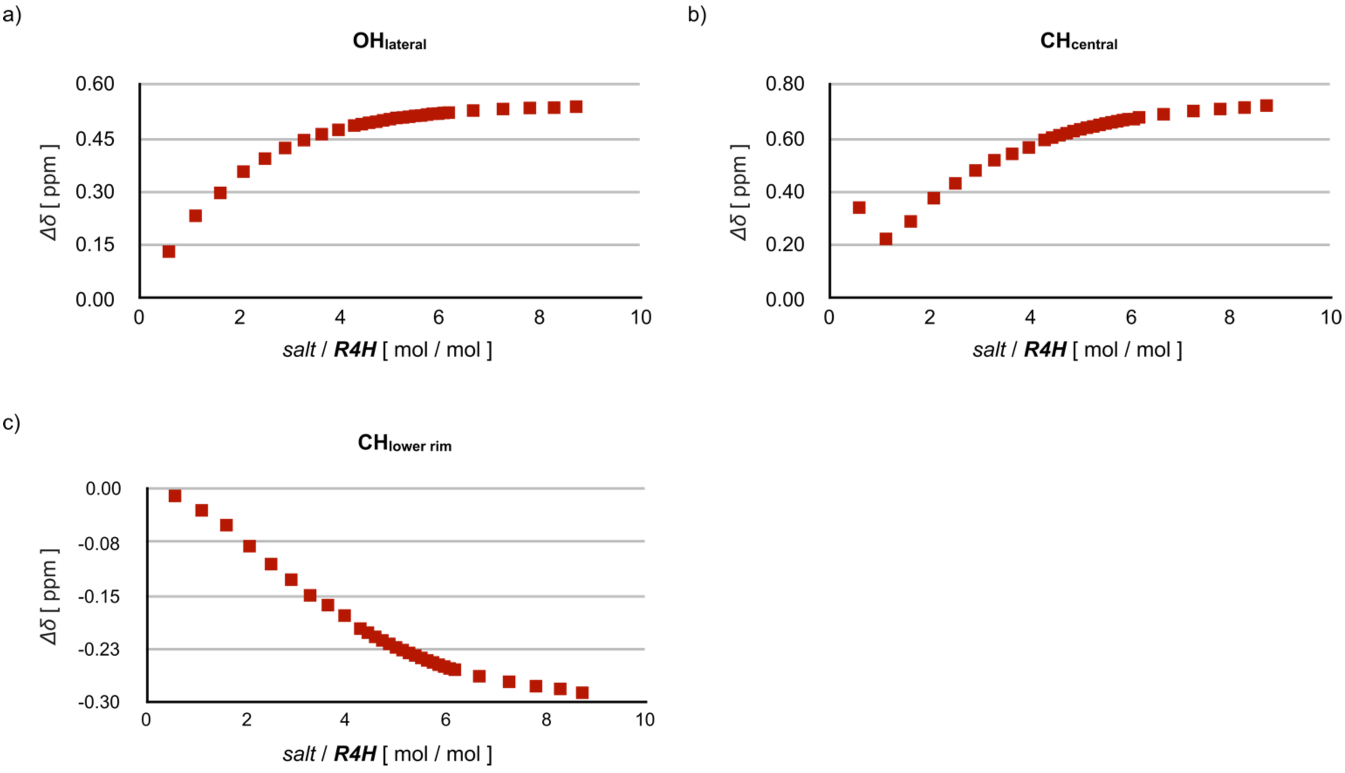

Figure S68. ^{1}H NMR titration curves for titration of **R4H** (C = 5.0 mM) with titrant **R4H** (C = 5.0 mM) + **Oct_4NBr** (C = 75 mM). ^{1}H NMR chemical shifts change for: (a) $OH_{lateral}$; (b) $OH_{central}$; (c) $CH_{lowr\ rim}$; (d) $CH_{2\ bridge}$ (400 MHz, 303 K, THF-d_8).

8.8 Titration of resorcin[4]arene (R4H) with tetradecylammonium bromide (Dec_4NBr)

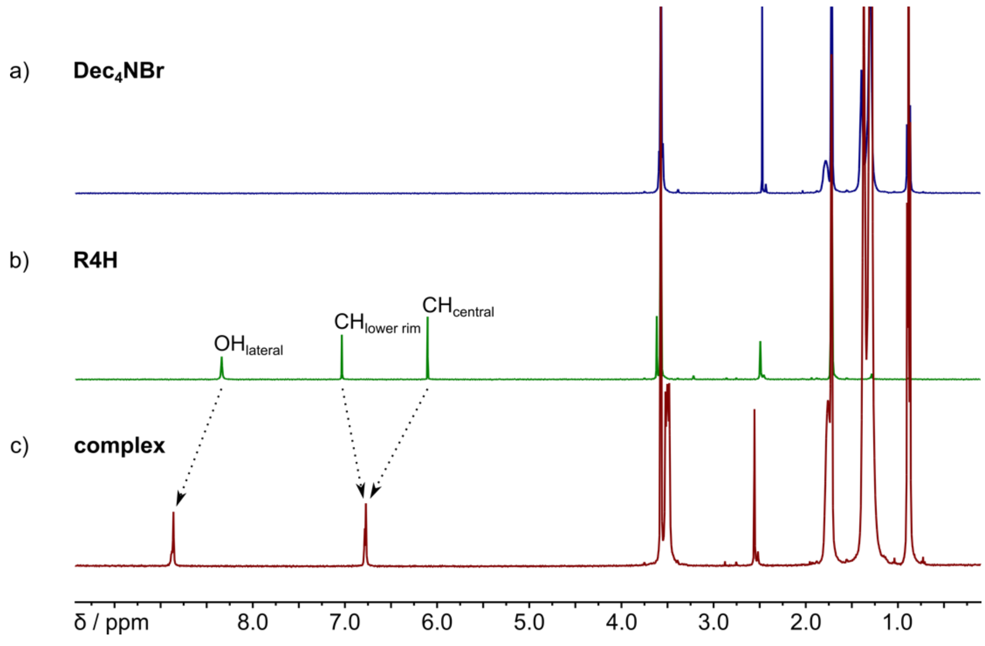

Figure S69. ^{1}H NMR spectra of (a) **Dec_4NBr**; (b) **R4H**; (c) complex of **R4H** and **Dec_4NBr** (400 MHz, 303 K, THF-d_8).

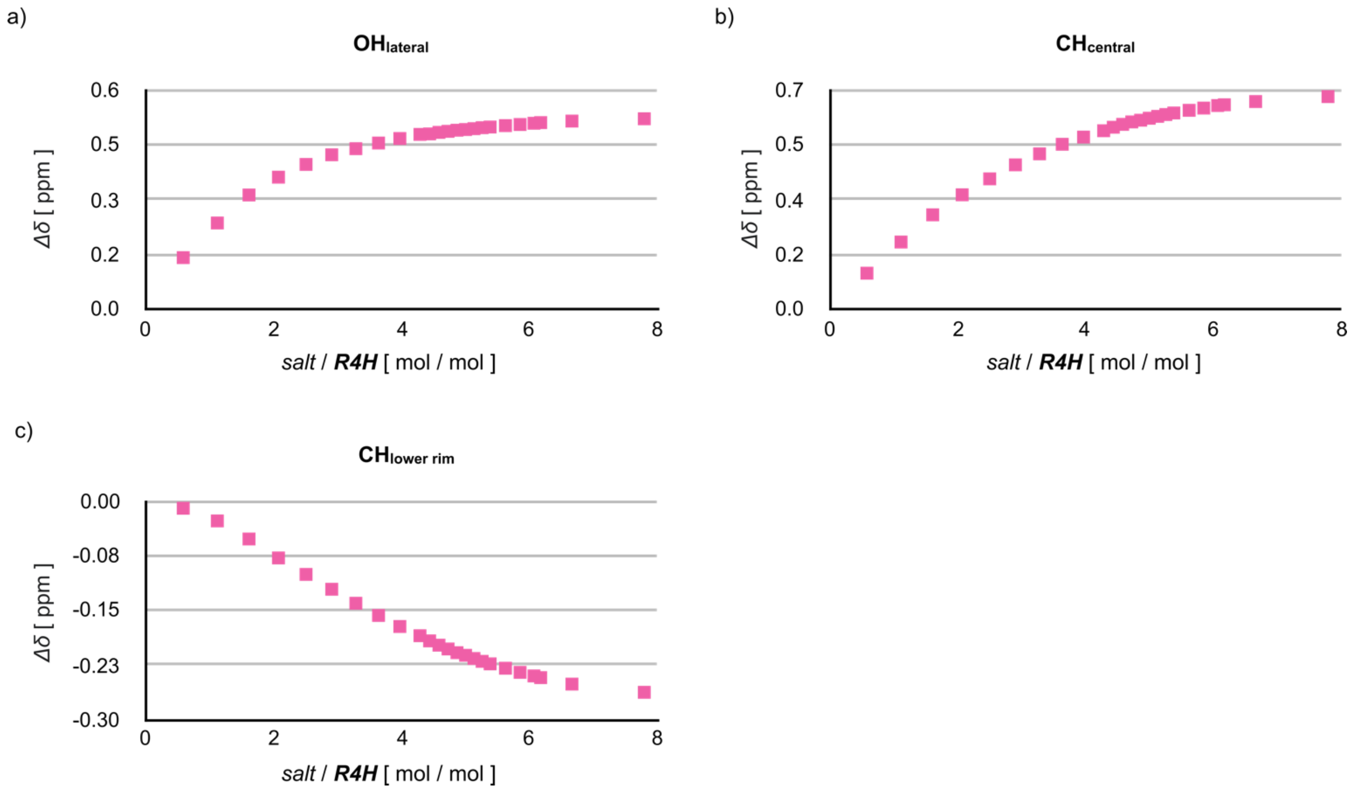

Figure S70. ^{1}H NMR titration curves for titration of **R4H** (C = 5.0 mM) with titrant **R4H** (C = 5.0 mM) + **Dec_4NBr** (C = 75 mM). ^{1}H NMR chemical shifts change for: (a) $OH_{lateral}$; (b) $OH_{central}$; (c) $CH_{lowr\ rim}$; (d) $CH_{2\ bridge}$ (400 MHz, 303 K, THF-d_8).

9. ^{1}H NMR spectra for mechanochemically treated samples

9.1 P4H with Hex$_4$NCl

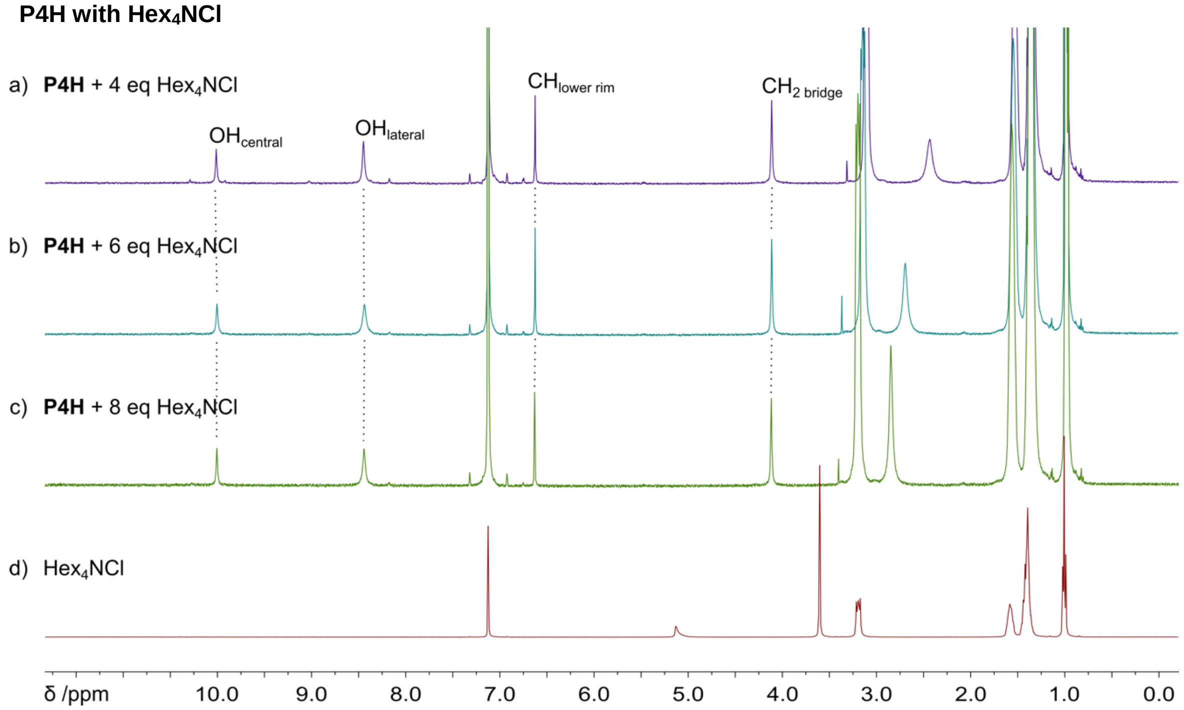

Figure S71. ^{1}H NMR of mechanochemically treated samples. Solid sample of **P4H** and **Hex$_4$NCl** were ball-milled (1h dry-milling in a planetary ball-mill) and the powders were dissolved in benzene-d$_6$ (0.7 ml) and then filtered. ^{1}H NMR spectrum of (a) **P4H** (10 mmol) **+ Hex$_4$NCl** (40 mmol); (b) **P4H** (6.7 mmol) **+ Hex$_4$NCl** (40 mmol); (c) **P4H** (5.0 mmol) **+ Hex$_4$NCl** (40 mmol); (d) reference sample **Hex$_4$NCl** (400 MHz, 303 K).

9.2 P4H with Oct$_4$NCl

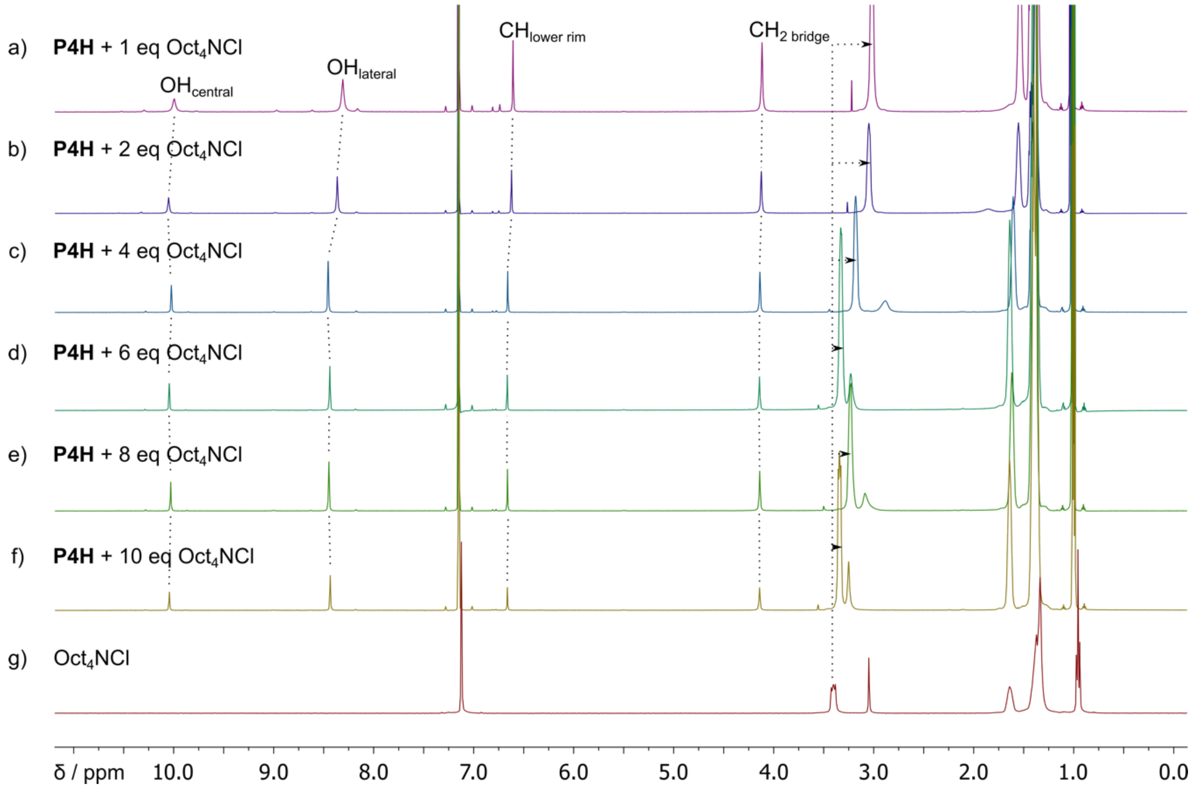

Figure S72. ^{1}H NMR of mechanochemically treated samples. Solid sample of **P4H** and **Oct$_4$NCl** were ball-milled (1h dry-milling in a planetary ball-mill) and the powders were dissolved in benzene-d$_6$ (0.7 ml) and then filtered. ^{1}H NMR spectrum of (a) **P4H** (40 mmol) **+ Oct$_4$NCl** (40 mmol); (b) **P4H** (20 mmol) **+ Oct$_4$NCl** (40 mmol); (c) **P4H** (10 mmol) **+ Oct$_4$NCl** (40 mmol); (d) **P4H** (6.7 mmol) **+ Oct$_4$NCl** (40 mmol); (e) **P4H** (5.0 mmol) **+ Oct$_4$NCl** (40 mmol); (f) **P4H** (4.0 mmol) **+ Oct$_4$NCl** (40 mmol); (g) reference sample **Oct$_4$NCl** (400 MHz, 303 K).

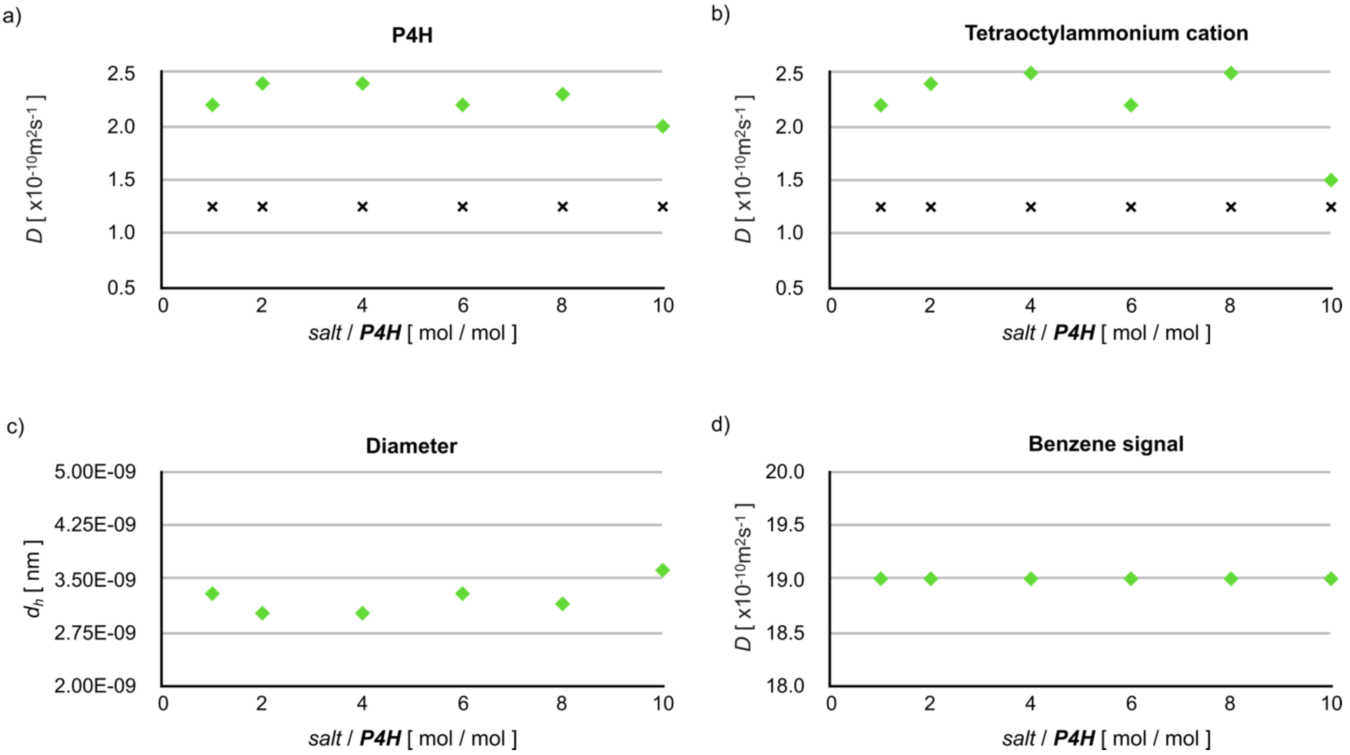

Figure S73. Solid sample of **P4H** (various rations from 0.04 mol to 0.005 mol) and **Oct$_4$NCl** (0.04 mol) were ball-milled (1h dry-milling in a planetary ball-mill) and the powders were dissolved in benzene-d$_6$ (0.7 ml). The sample was filtered and the solution was checked by ^{1}H NMR. Diffusion coefficient changes: for (a) **P4H;** (b) **Oct$_4$NCl**; (c) diameter of complex; (d) benzene (600 MHz, 303 K, benzene-d$_6$). **x**- free salt sygnal (0.04 mmol)

P4H	Oct$_4$N$^+$	salt / M [mol / mol]
2.2	2.2	1
2.4	2.4	2
2.4	2.5	4
2.2	2.2	6
2.3	2.5	8
2.2	2.2	10

Table S9. Data for DOSY titration (sample preparation as above).

9.3 P4H with Pen_4NBr

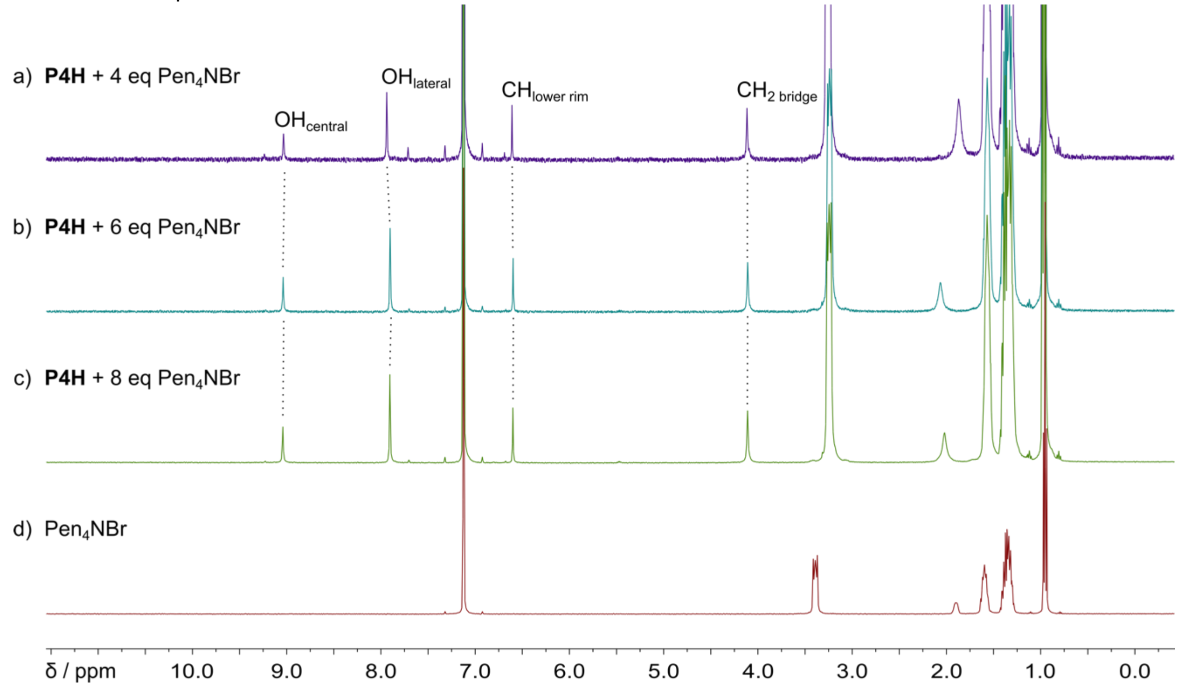

Figure S74. ^{1}H NMR of mechanochemically treated samples. Solid sample of **P4H** and **Pen_4NBr** were ball-milled (1h dry-milling in a planetary ball-mill) and the powders were dissolved in benzene-d_6 (0.7 ml) and then filtered. ^{1}H NMR spectrum of (a) **P4H** (10 mmol) **+ Pen_4NBr** (40 mmol); (b) **P4H** (6.7 mmol) **+ Pen_4NBr** (40 mmol); (c) **P4H** (5.0 mmol) **+ Pen_4NBr** (40 mmol); (d) reference sample **Pen_4NBr** (400 MHz, 303 K).

9.4 P4H with Hex_4NBr

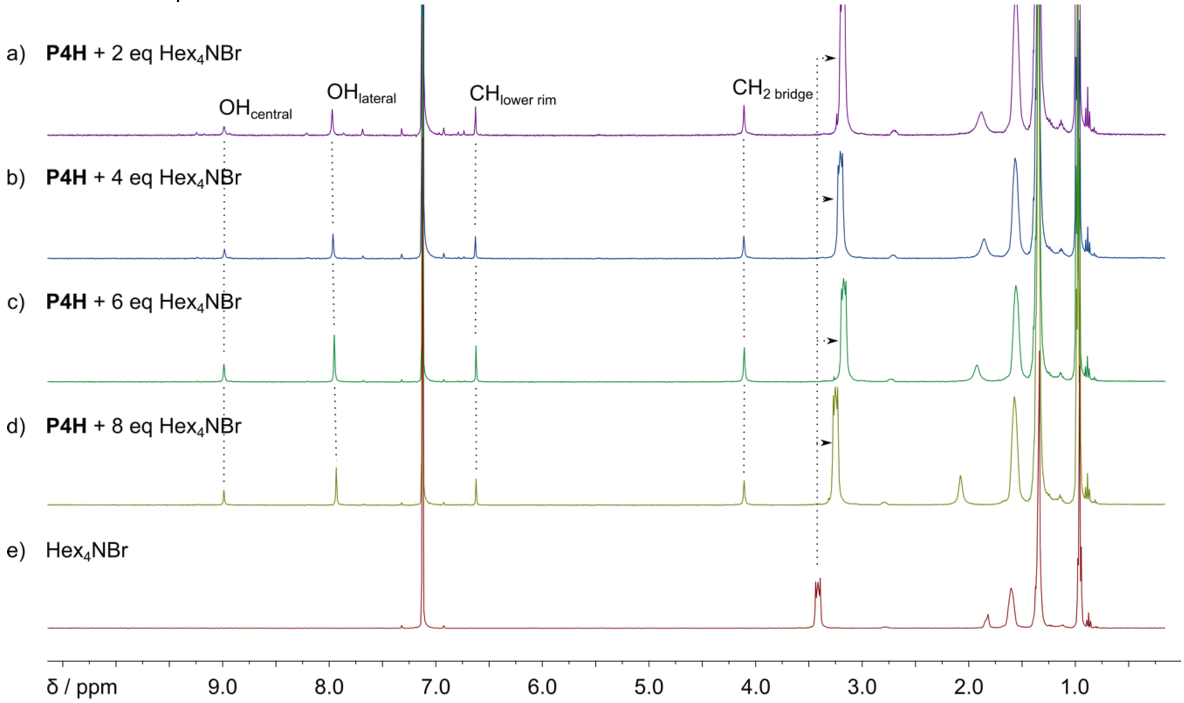

Figure S75. ^{1}H NMR of mechanochemically treated samples. Solid sample of **P4H** and **Hex_4NBr** were ball-milled (1h dry-milling in a planetary ball-mill) and the powders were dissolved in benzene-d_6 (0.7 ml) and then filtered. ^{1}H NMR spectrum of (a) **P4H** (20 mmol) **+ Hex_4NBr** (40 mmol); (b) **P4H** (10 mmol) **+ Hex_4NBr** (40 mmol); (c) **P4H** (6.7 mmol) **+ Hex_4NBr** (40 mmol); (d) **P4H** (5.0 mmol) **+ Hex_4NBr** (40 mmol); (e) reference sample **Hex_4NBr** (400 MHz, 303 K).

9.5 P4H with Oct$_4$NBr

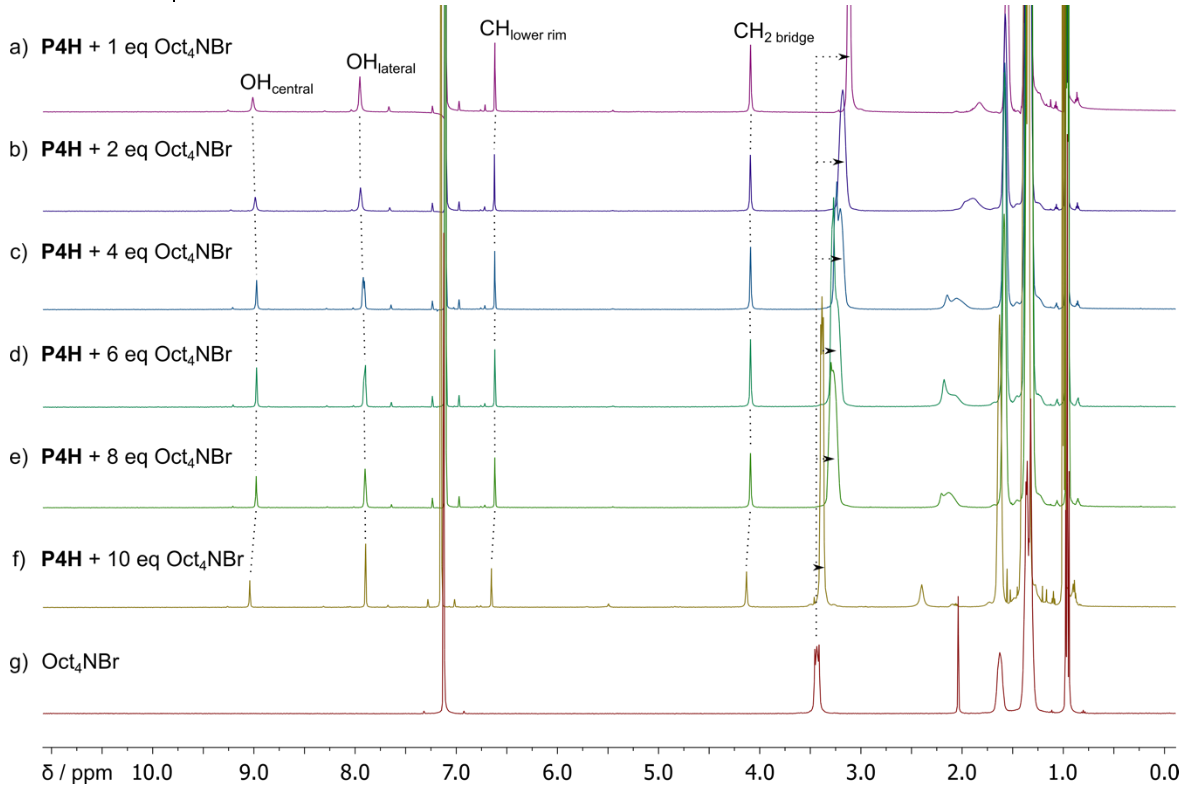

Figure S76. ^{1}H NMR of mechanochemically treated samples. Solid sample of **P4H** and **Oct$_4$NBr** were ball-milled (1h dry-milling in a planetary ball-mill) and the powders were dissolved in benzene-d$_6$ (0.7 ml) and then filtered. ^{1}H NMR spectrum of (a) **P4H** (40 mmol) **+ Oct$_4$NBr** (40 mmol); (b) **P4H** (20 mmol) **+ Oct $_4$NBr** (40 mmol); (c) **P4H** (10 mmol) **+ Oct $_4$NBr** (40 mmol); (d) **P4H** (6.7 mmol) **+ Oct $_4$NBr** (40 mmol); (e) **P4H** (5.0 mmol) **+ Oct $_4$NBr** (40 mmol); (f) **P4H** (4.0 mmol) **+ Oct $_4$NBr** (40 mmol); (g) reference sample **Oct $_4$NBr** (400 MHz, 303 K).

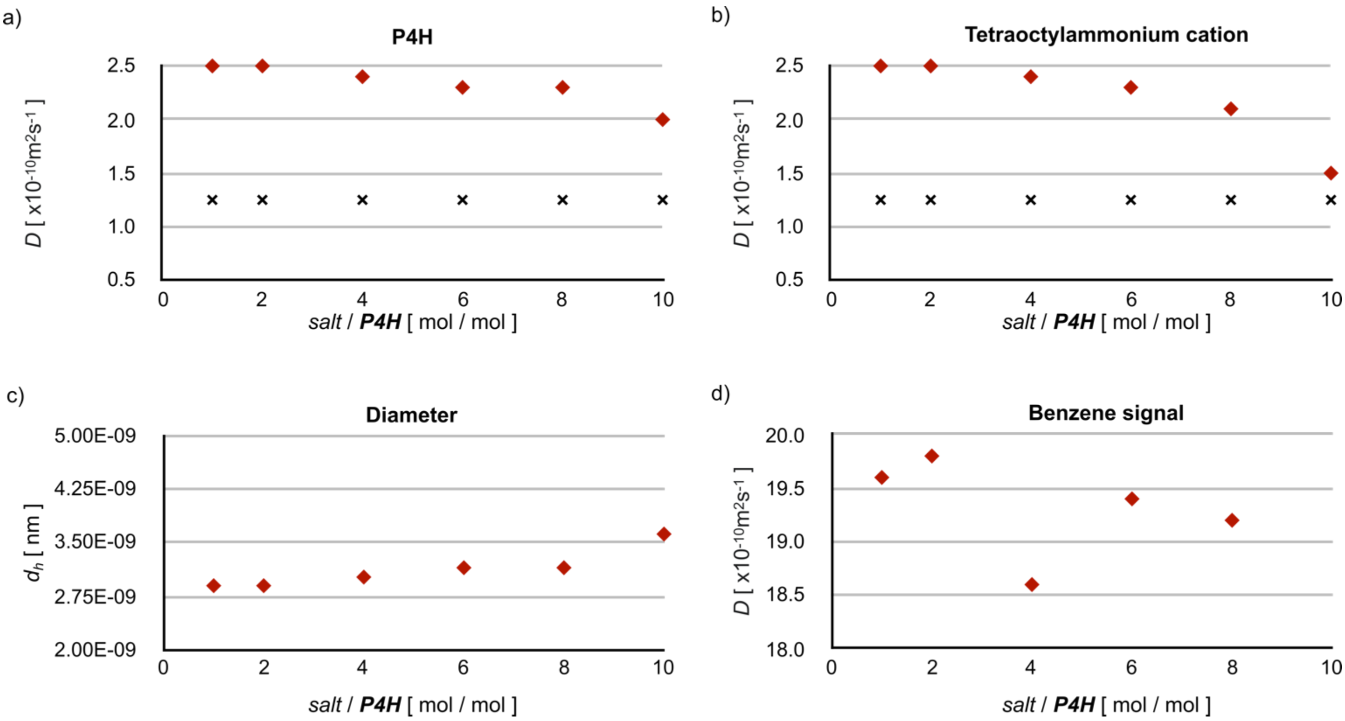

Figure S77. Solid sample of **P4H** (various rations from 0.04 mmol to 0.005 mmol) and **Oct$_4$NBr** (0.04 mol) were ball-milled (1h dry-milling in a planetary ball-mill) and the powders were dissolved in benzene-d$_6$ (0.7 ml). The sample was filtered and the solution was checked by ^{1}H NMR. Diffusion coefficient changes: for (a) **P4H;** (b) **Oct$_4$NBr**; (c) diameter of complex; (d) benzene (600 MHz, 303 K, benzene-d$_6$). **x**- free salt sygnal (0.04 mmol)

P4H	Oct_4N^+	salt / M [mol / mol]
2.5	2.5	1
2.5	2.5	2
2.4	2.4	4
2.3	2.3	6
2.3	2.1	8
2.0	1.5	10

Table S10. Data for DOSY titration (sample preparation as above).

9.6 P4C with Hex_4NCl

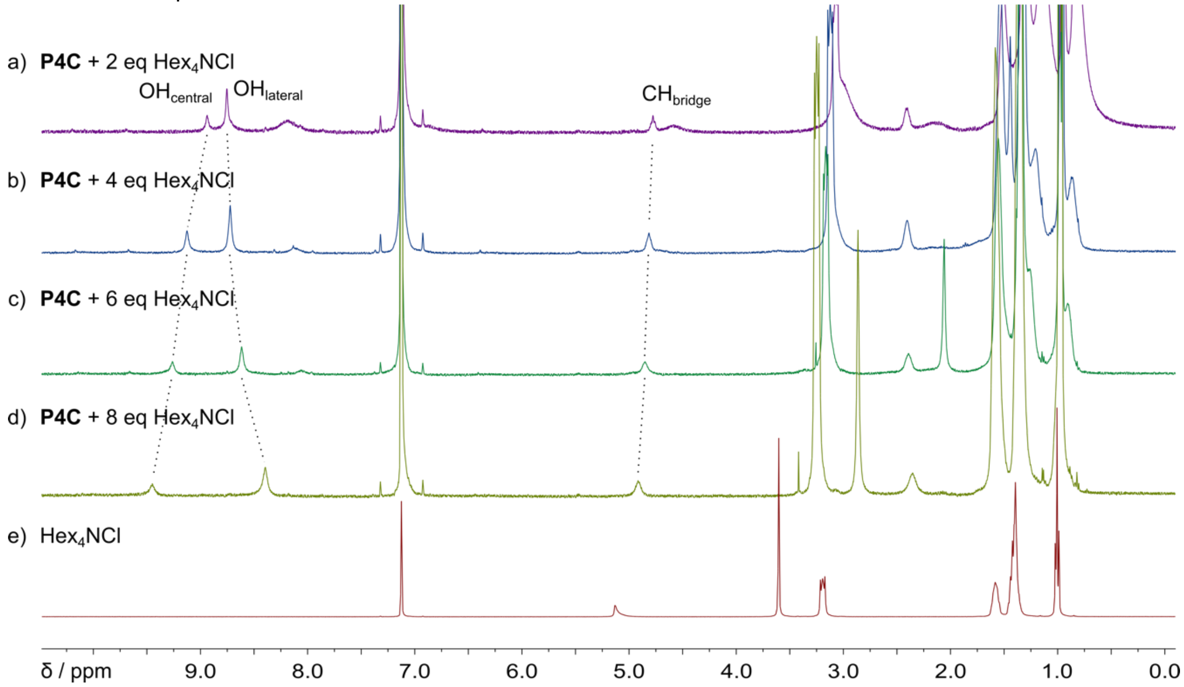

Figure S78. ^{1}H NMR of mechanochemically treated samples. Solid sample of **P4C** and **Hex_4NCl** were ball-milled (1h dry-milling in a planetary ball-mill) and the powders were dissolved in benzene-d_6 (0.7 ml) and then filtered. ^{1}H NMR spectrum of (a) **P4C** (20 mmol) **+ Hex_4NCl** (40 mmol); (b) **P4C** (10 mmol) **+ Hex_4NCl** (40 mmol); (c) **P4C** (6.7 mmol) **+ Hex_4NCl** (40 mmol); (d) **P4C** (5.0 mmol) **+ Hex_4NCl** (40 mmol); (e) reference sample **Hex_4NCl** (400 MHz, 303 K).

9.7 P4C with Oct_4NCl

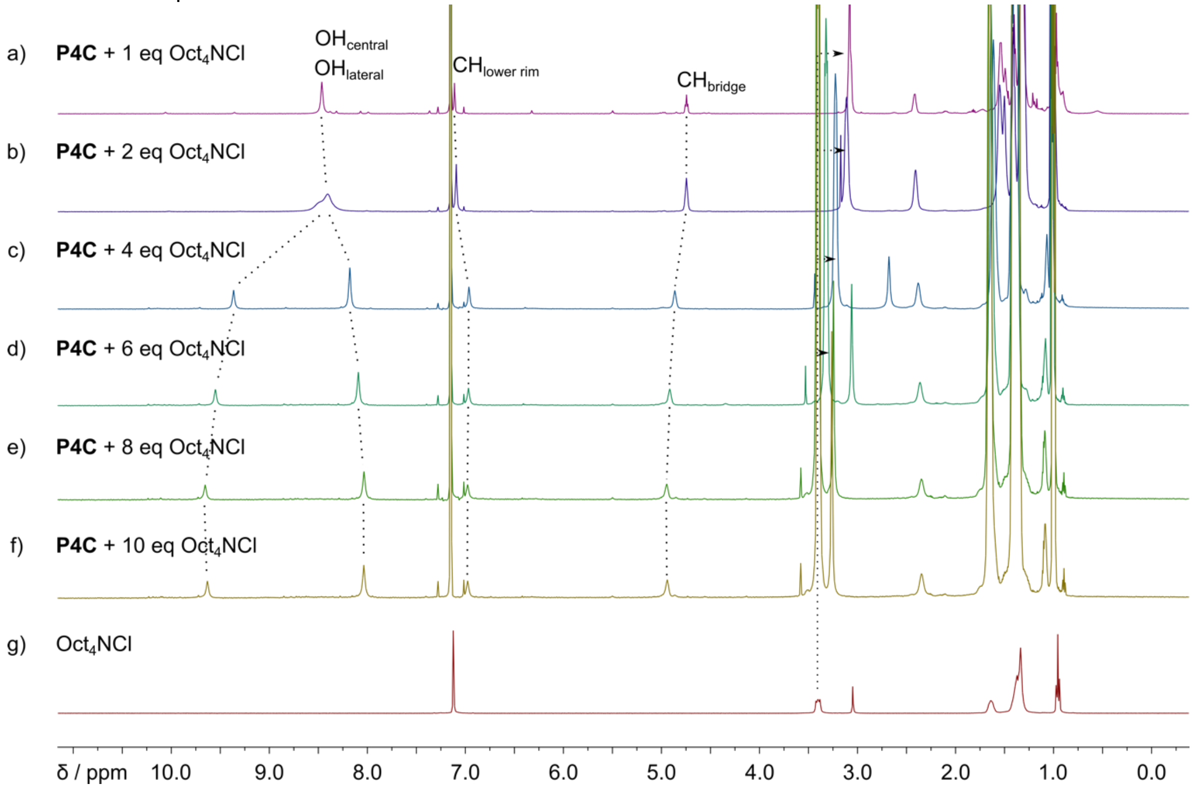

Figure S79. ^{1}H NMR of mechanochemically treated samples. Solid sample of **P4C** and **Oct_4NCl** were ball-milled (1h dry-milling in a planetary ball-mill) and the powders were dissolved in benzene-d_6 (0.7 ml) and then filtered. ^{1}H NMR spectrum of (a) **P4C** (40 mmol) **+ Oct_4NCl** (40 mmol); (b) **P4C** (20 mmol) **+ Oct_4NCl** (40 mmol); (c) **P4C** (10 mmol) **+ Oct_4NCl** (40 mmol); (d) **P4C** (6.7 mmol) **+ Oct_4NCl** (40 mmol); (e) **P4C** (5.0 mmol) **+ Oct_4NCl** (40 mmol); (f) **P4C** (4.0 mmol) **+ Oct_4NCl** (40 mmol); (g) reference sample **Oct_4NCl** (400 MHz, 303 K).

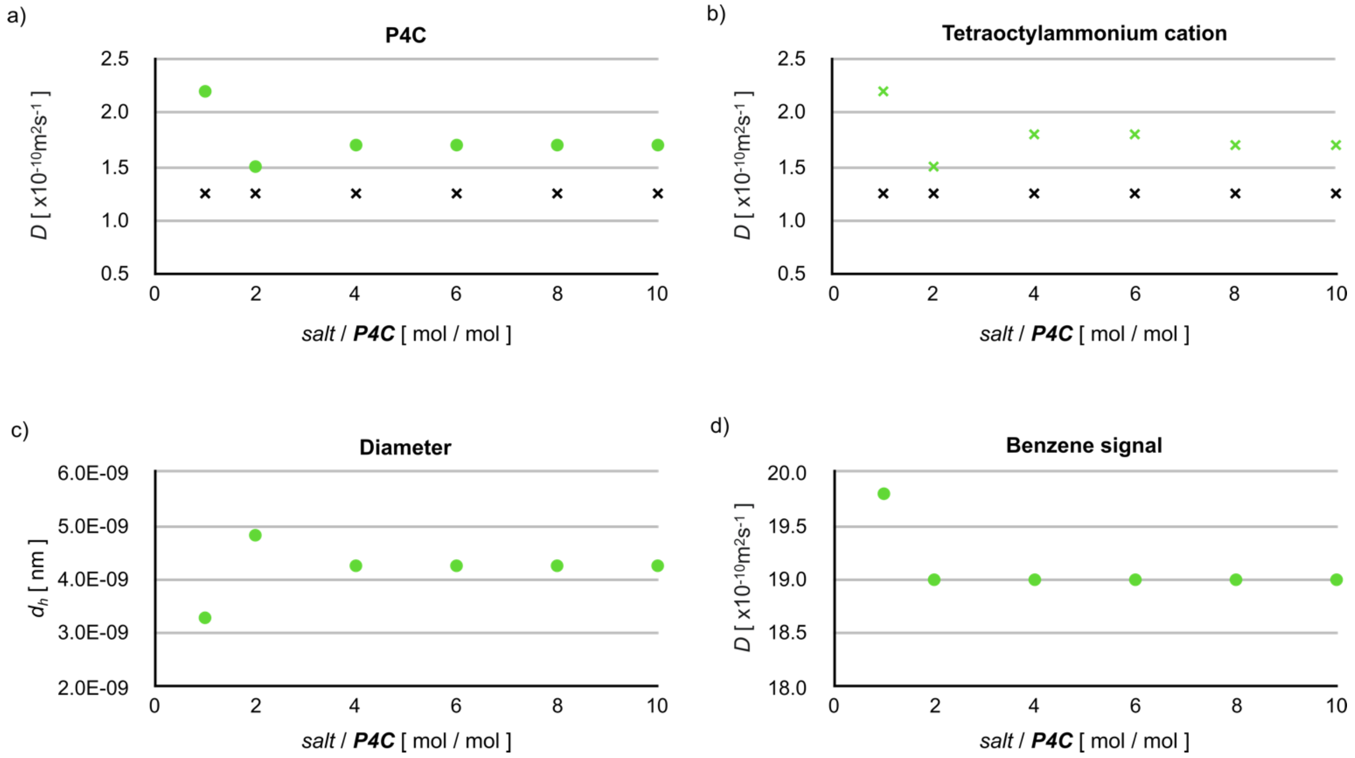

Figure S80. Solid sample of **P4C** (various rations from 0.04 mol to 0.005 mol) and **Oct$_4$NCl** (0.04 mol) were ball-milled (1h dry-milling in a planetary ball-mill) and the powders were dissolved in benzene-d$_6$ (0.7 ml). The sample was filtered and the solution was checked by ^{1}H NMR. Diffusion coefficient changes: for (a) **P4C;** (b) **Oct$_4$NCl**; (c) diameter of complex; (d) benzene (600 MHz, 303 K, benzene-d$_6$). **x**- free salt sygnal (0.04 mmol)

P4C	Oct$_4$N$^+$	salt / M [mol / mol]
2.2	2.2	1
1.5	1.5	2
1.7	1.8	4
1.7	1.8	6
1.7	1.7	8
1.7	1.7	10

Table S11. Data for DOSY titration (sample preparation as above).

9.8 P4C with Pen_4NBr

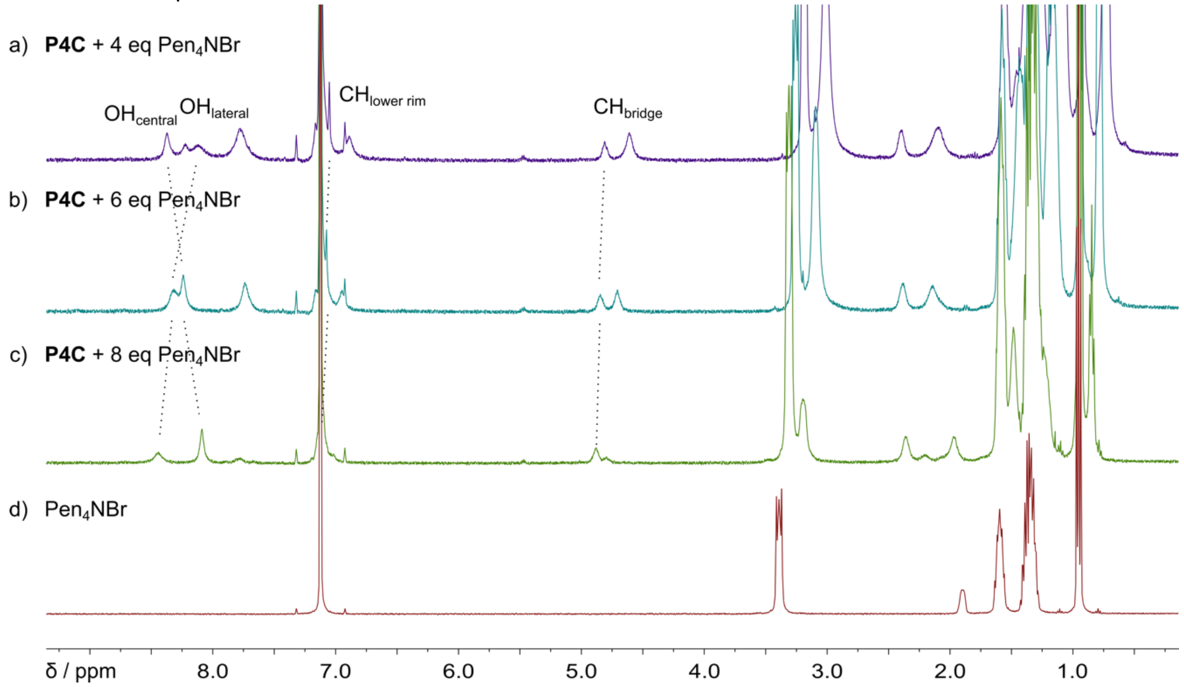

Figure S81. ^{1}H NMR of mechanochemically treated samples. Solid sample of **P4C** and **Pen_4NBr** were ball-milled (1h dry-milling in a planetary ball-mill) and the powders were dissolved in benzene-d_6 (0.7 ml) and then filtered. ^{1}H NMR spectrum of (a) **P4C** (10 mmol) **+ Pen_4NBr** (40 mmol); (b) **P4C** (6.7 mmol) **+ Pen_4NBr** (40 mmol); (c) **P4C** (5.0 mmol) **+ Pen_4NBr** (40 mmol); (d) reference sample **Pen_4NBr** (400 MHz, 303 K).

9.9 P4C with Hex_4NBr

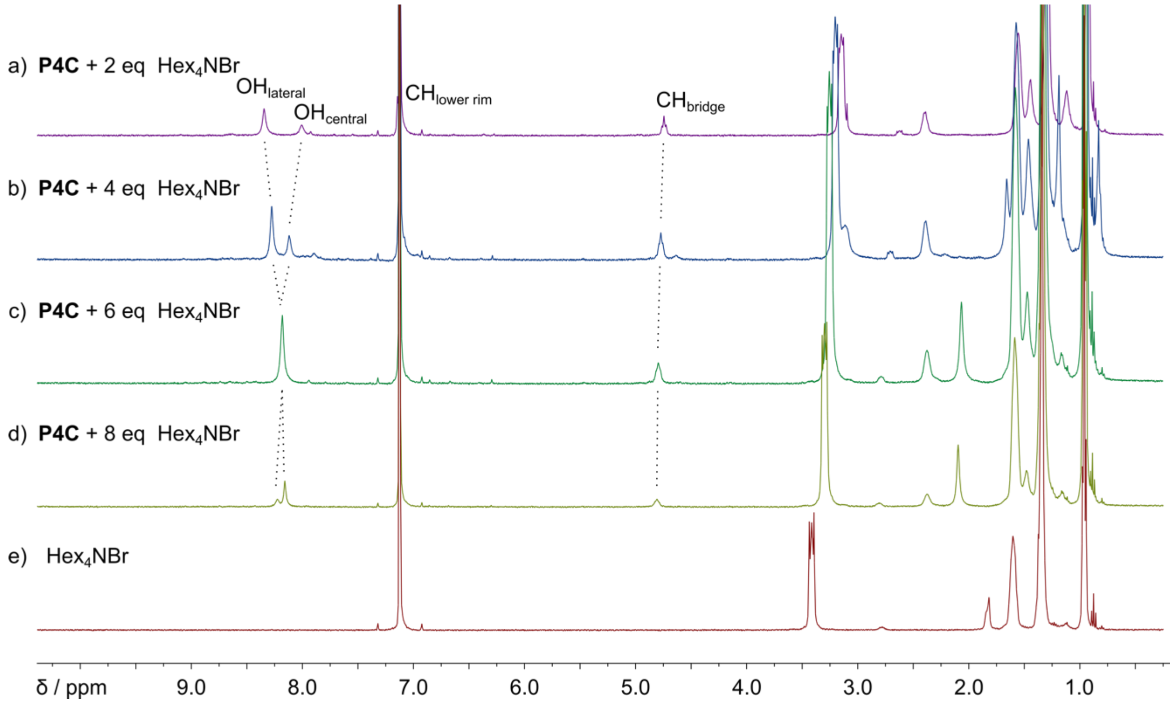

Figure S82. ^{1}H NMR of mechanochemically treated samples. Solid sample of **P4C** and **Hex_4NBr** were ball-milled (1h dry-milling in a planetary ball-mill) and the powders were dissolved in benzene-d_6 (0.7 ml) and then filtered. ^{1}H NMR spectrum of (a) **P4C** (20 mmol) **+ Hex_4NBr** (40 mmol); (b) **P4C** (10 mmol) **+ Hex_4NBr** (40 mmol); (c) **P4C** (6.7 mmol) **+ Hex_4NBr** (40 mmol); (d) **P4C** (5.0 mmol) **+ Hex_4NBr** (40 mmol); (e) reference sample **Hex_4NBr** (400 MHz, 303 K).

9.10 P4C with Oct_4NBr

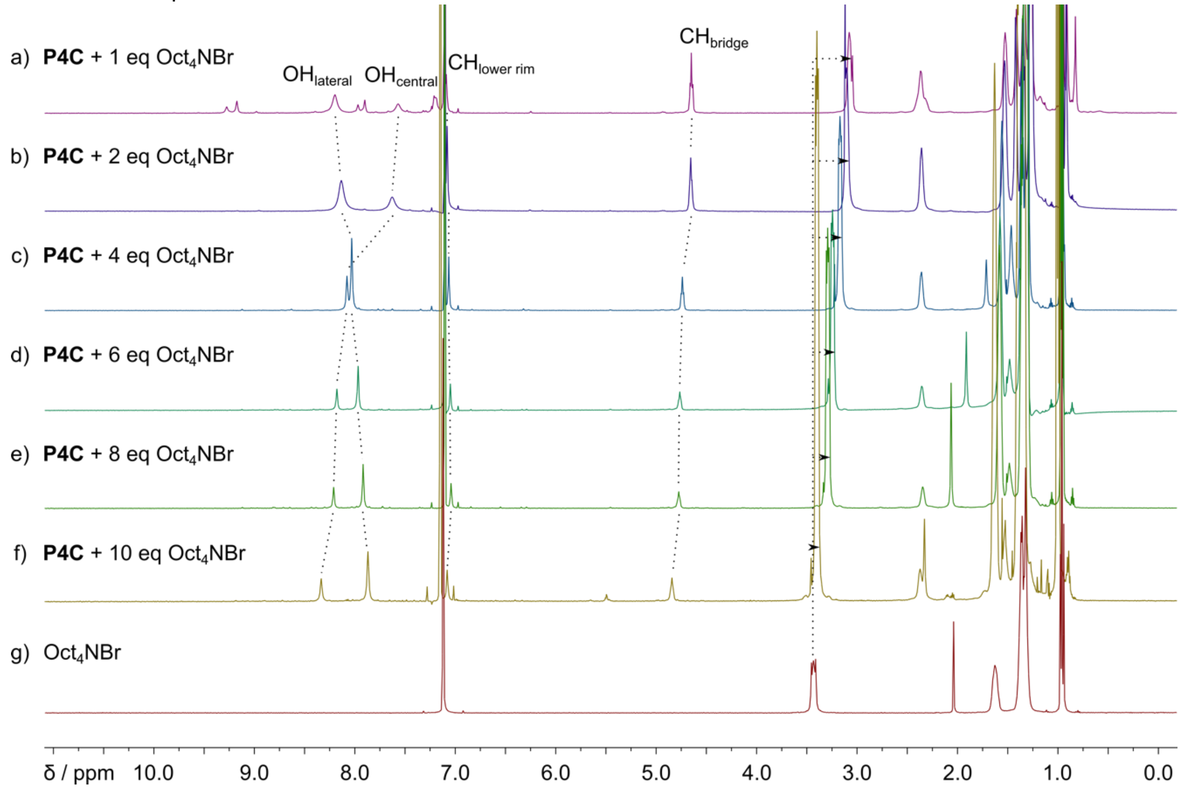

Figure S83. ^{1}H NMR of mechanochemically treated samples. Solid sample of **P4C** and **Oct_4NBr** were ball-milled (1h dry-milling in a planetary ball-mill) and the powders were dissolved in benzene-d_6 (0.7 ml) and then filtered. ^{1}H NMR spectrum of (a) **P4C** (40 mmol) **+ Oct_4NBr** (40 mmol); (b) **P4C** (20 mmol) **+ Oct_4NBr** (40 mmol); (c) **P4C** (10 mmol) **+ Oct_4NBr** (40 mmol); (d) **P4C** (6.7 mmol) **+ Oct_4NBr** (40 mmol); (e) **P4C** (5.0 mmol) **+ Oct_4NBr** (40 mmol); (f) **P4C** (4.0 mmol) **+ Oct_4NBr** (40 mmol); (g) reference sample **Oct_4NBr** (400 MHz, 303 K).

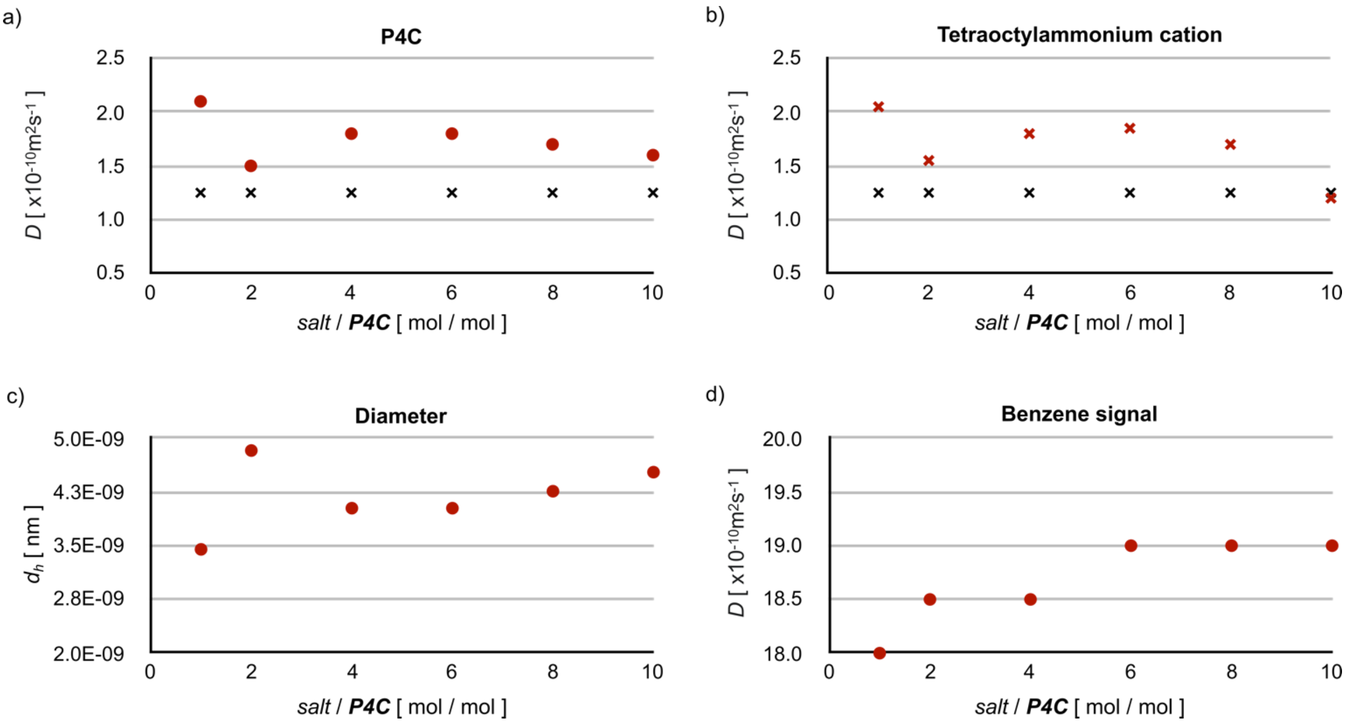

Figure S84. Solid sample of **P4C** (various rations from 0.04 mol to 0.005 mol) and **Oct_4NBr** (0.04 mmol) were ball-milled (1h dry-milling in a planetary ball-mill) and the powders were dissolved in benzene-d_6 (0.7 ml). The sample was filtered and the solution was checked by ^{1}H NMR. Diffusion coefficient changes: for (a) **P4C;** (b) **Oct_4NBr**; (c) diameter of complex; (d) benzene (600 MHz, 303 K, benzene-d_6). **x**- free salt sygnal (0.04 mmol)

P4C	Oct_4N^+	salt / M [mol / mol]
2.1	2.0	1
1.5	1.6	2
1.8	1.8	4
1.8	1.85	6
1.7	1.7	8
1.6	1.2	10

Table S12. Data for DOSY titration (sample preparation as above).

9.11 R4H with Hex_4NCl

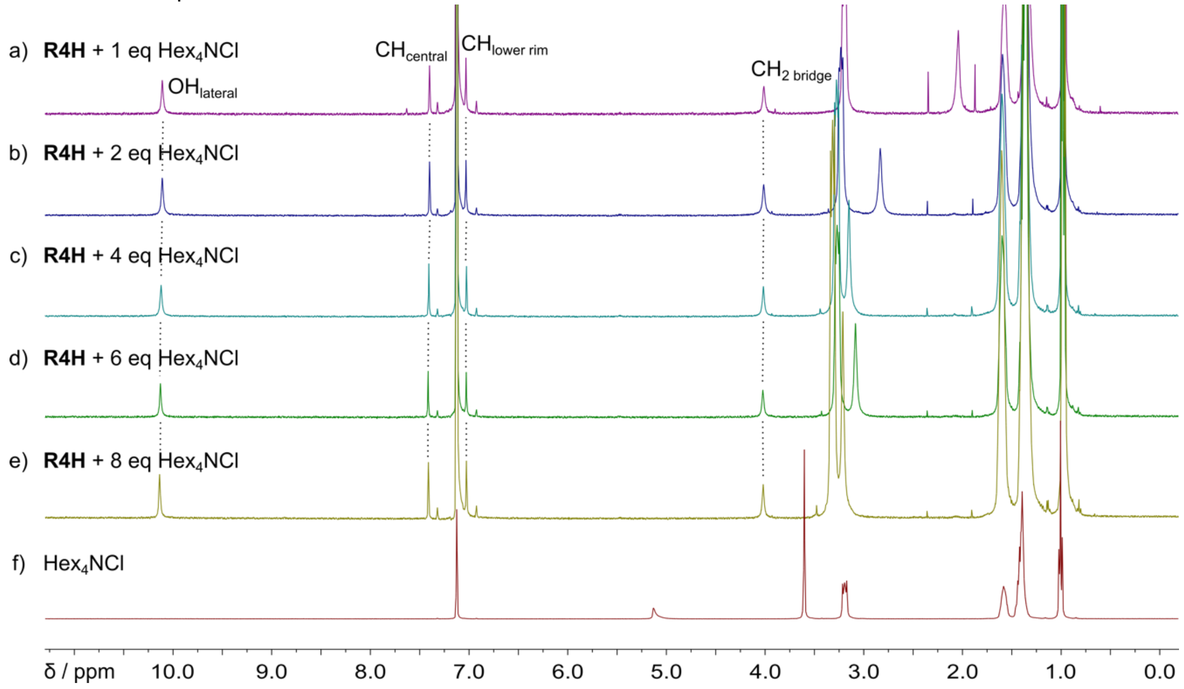

Figure S85. ^{1}H NMR of mechanochemically treated samples. Solid sample of **R4H** and **Hex_4NCl** were ball-milled (1h dry-milling in a planetary ball-mill) and the powders were dissolved in benzene-d_6 (0.7 ml) and then filtered. ^{1}H NMR spectrum of (a) **R4H** (40 mmol) **+ Hex_4NCl** (40 mmol); (b) **R4H** (20 mmol) **+ Hex_4NCl** (40 mmol); (c) **R4H** (10 mmol) **+ Hex_4NCl** (40 mmol); (d) **R4H** (6.7 mmol) **+ Hex_4NCl** (40 mmol); (e) **R4H** (5.0 mmol) **+ Hex_4NCl** (40 mmol); (f) reference sample **Hex_4NCl** (400 MHz, 303 K).

9.12 R4H with Oct_4NCl

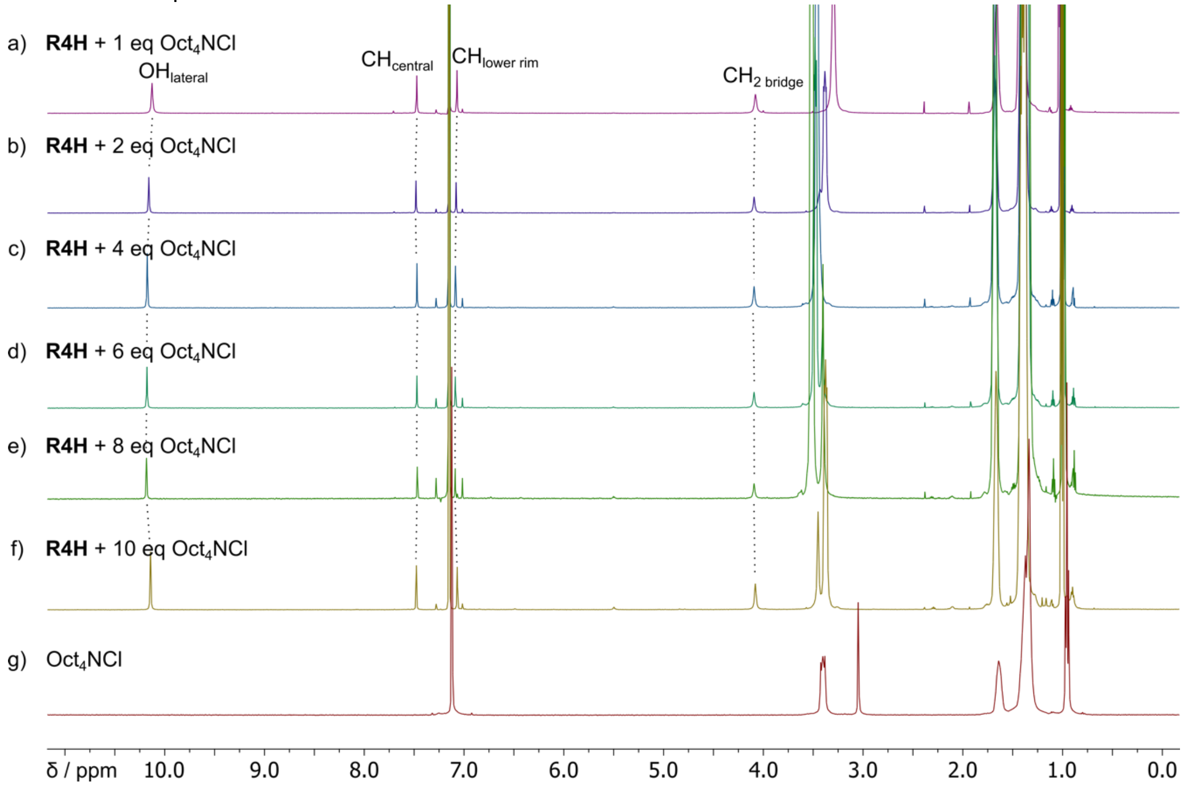

Figure S86. ^{1}H NMR of mechanochemically treated samples. Solid sample of **R4H** and **Oct_4NCl** were ball-milled (1h dry-milling in a planetary ball-mill) and the powders were dissolved in benzene-d_6 (0.7 ml) and then filtered. ^{1}H NMR spectrum of (a) **R4H** (40 mmol) **+ Oct_4NCl** (40 mmol); (b) **R4H** (20 mmol) **+ Oct_4NCl** (40 mmol); (c) **R4H** (10 mmol) **+ Oct_4NCl** (40 mmol); (d) **R4H** (6.7 mmol) **+ Oct_4NCl** (40 mmol); (e) **R4H** (5.0 mmol) **+ Oct_4NCl** (40 mmol); (f) **R4H** (4.0 mmol) **+ Oct_4NCl** (40 mmol); (g) reference sample **Oct_4NCl** (400 MHz, 303 K).

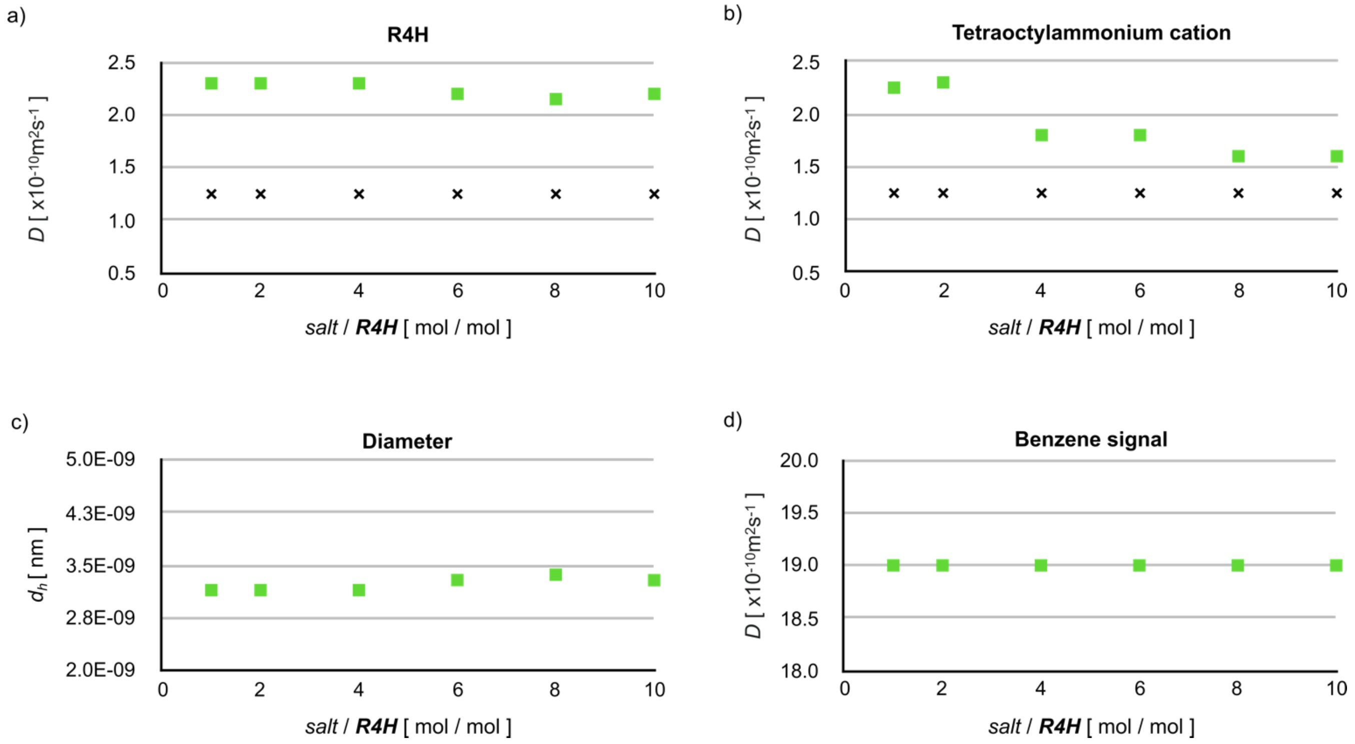

Figure S87. Solid sample of **R4H** (various rations from 0.04 mol to 0.005 mol) and **Oct_4NCl** (0.04 mol) were ball-milled (1h dry-milling in a planetary ball-mill) and the powders were dissolved in benzene-d_6 (0.7 ml). The sample was filtered and the solution was checked by ^{1}H NMR. Diffusion coefficient changes: for (a) **R4H;** (b) **Oct_4NCl**; (c) diameter of complex; (d) benzene (600 MHz, 303 K, benzene-d_6). **x**- free salt sygnal (0.04 mmol)

R4H	Oct_4N^+	salt / M [mol / mol]
2.3	2.3	1
2.3	2.3	2
2.3	1.8	4
2.2	1.8	6
2.2	1.6	8
2.2	2.2	10

Table S13. Data for DOSY titration (sample preparation as above).

9.13 R4H with Pen_4NBr

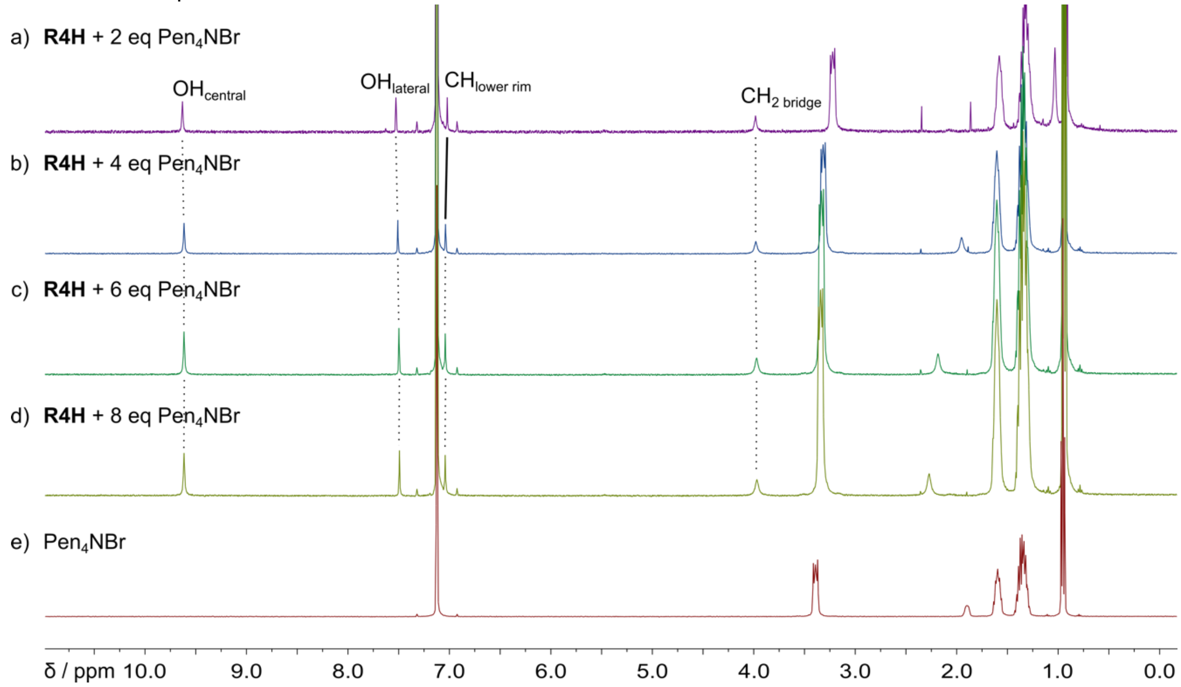

Figure S88. ^{1}H NMR of mechanochemically treated samples. Solid sample of **R4H** and **Pen_4NCl** were ball-milled (1h dry-milling in a planetary ball-mill) and the powders were dissolved in benzene-d_6 (0.7 ml) and then filtered. ^{1}H NMR spectrum of (a) **R4H** (20 mmol) + **Pen_4NBr** (40 mmol); (b) **R4H** (10 mmol) + **Pen_4NBr** (40 mmol); (c) **R4H** (6.7 mmol) + **Pen_4NBr** (40 mmol); (d) **R4H** (5.0 mmol) + **Pen_4NBr** (40 mmol); (e) reference sample **Pen_4NBr** (400 MHz, 303 K).

9.14 R4H with Hex_4NBr

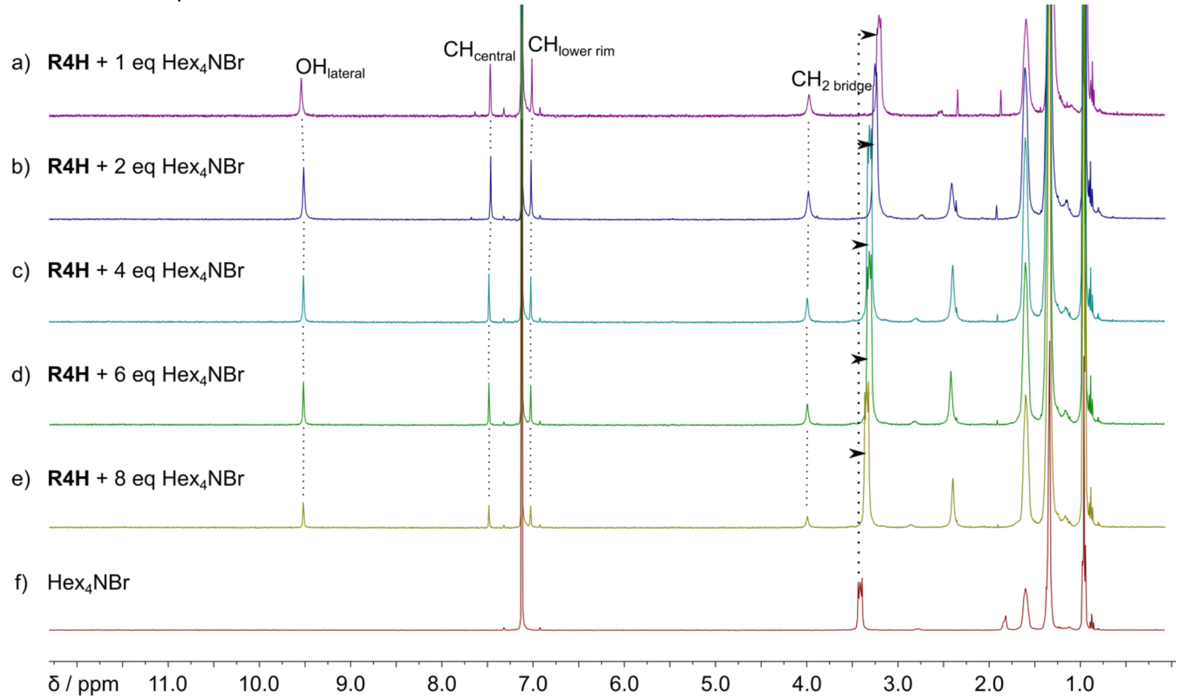

Figure S89. ^{1}H NMR of mechanochemically treated samples. Solid sample of **R4H** and **Hex_4NBr** were ball-milled (1h dry-milling in a planetary ball-mill) and the powders were dissolved in benzene-d_6 (0.7 ml) and then filtered. ^{1}H NMR spectrum of (a) **R4H** (40 mmol) **+ Hex_4NBr** (40 mmol); (b) **R4H** (20 mmol) **+ Hex_4NBr** (40 mmol); (c) **R4H** (10 mmol) **+ Hex_4NBr** (40 mmol); (d) **R4H** (6.7 mmol) **+ Hex_4NBr** (40 mmol); (e) **R4H** (5.0 mmol) **+ Hex_4NBr** (40 mmol); (f) reference sample **Hex_4NBr** (400 MHz, 303 K).

9.15 R4H with Oct_4NBr

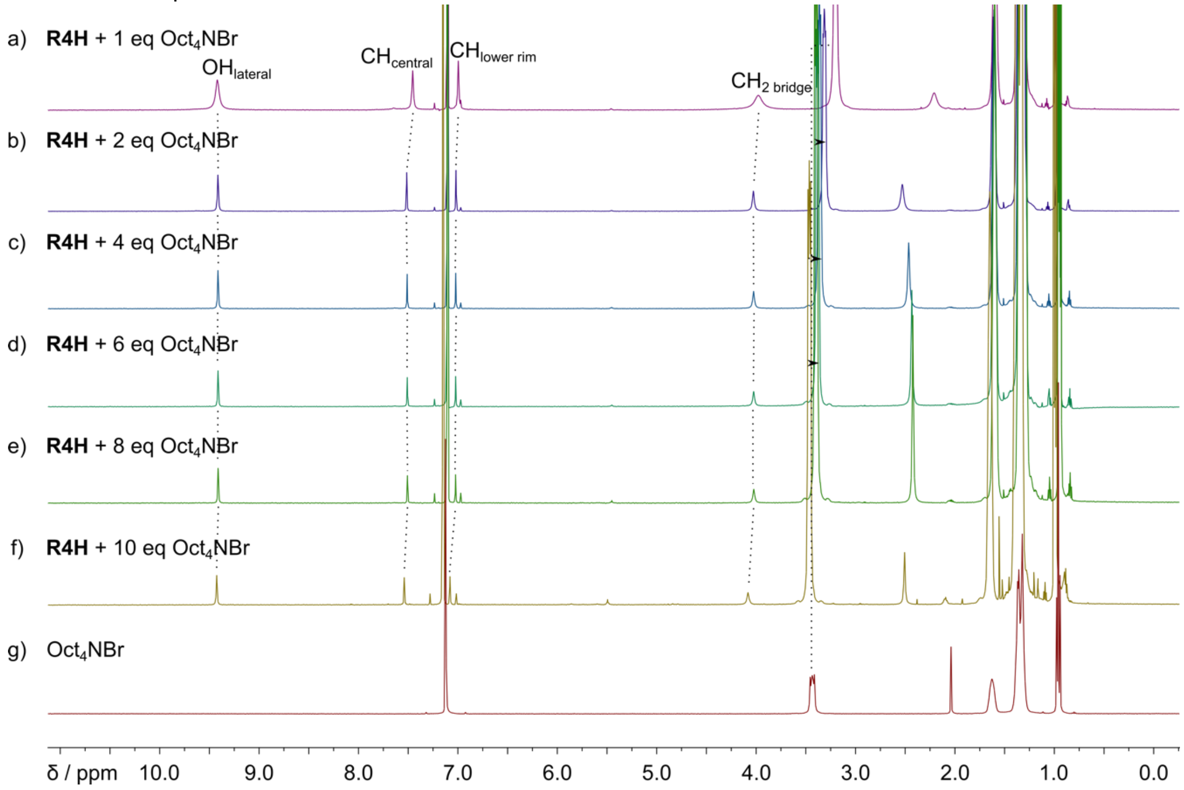

Figure S90. ^{1}H NMR of mechanochemically treated samples. Solid sample of **R4H** and **Oct_4NBr** were ball-milled (1h dry-milling in a planetary ball-mill) and the powders were dissolved in benzene-d_6 (0.7 ml) and then filtered. ^{1}H NMR spectrum of (a) **R4H** (40 mmol) **+ Oct_4NBr** (40 mmol); (b) **R4H** (20 mmol) **+ Oct_4NBr** (40 mmol); (c) **R4H** (10 mmol) **+ Oct_4NBr** (40 mmol); (d) **R4H** (6.7 mmol) **+ Oct_4NBr** (40 mmol); (e) **R4H** (5.0 mmol) **+ Oct_4NBr** (40 mmol); (f) **R4H** (4.0 mmol) **+ Oct_4NBr** (40 mmol); (g) reference sample **Oct_4NBr** (400 MHz, 303 K). All samples were dissolved in benzene-d_6 (0.7 ml).

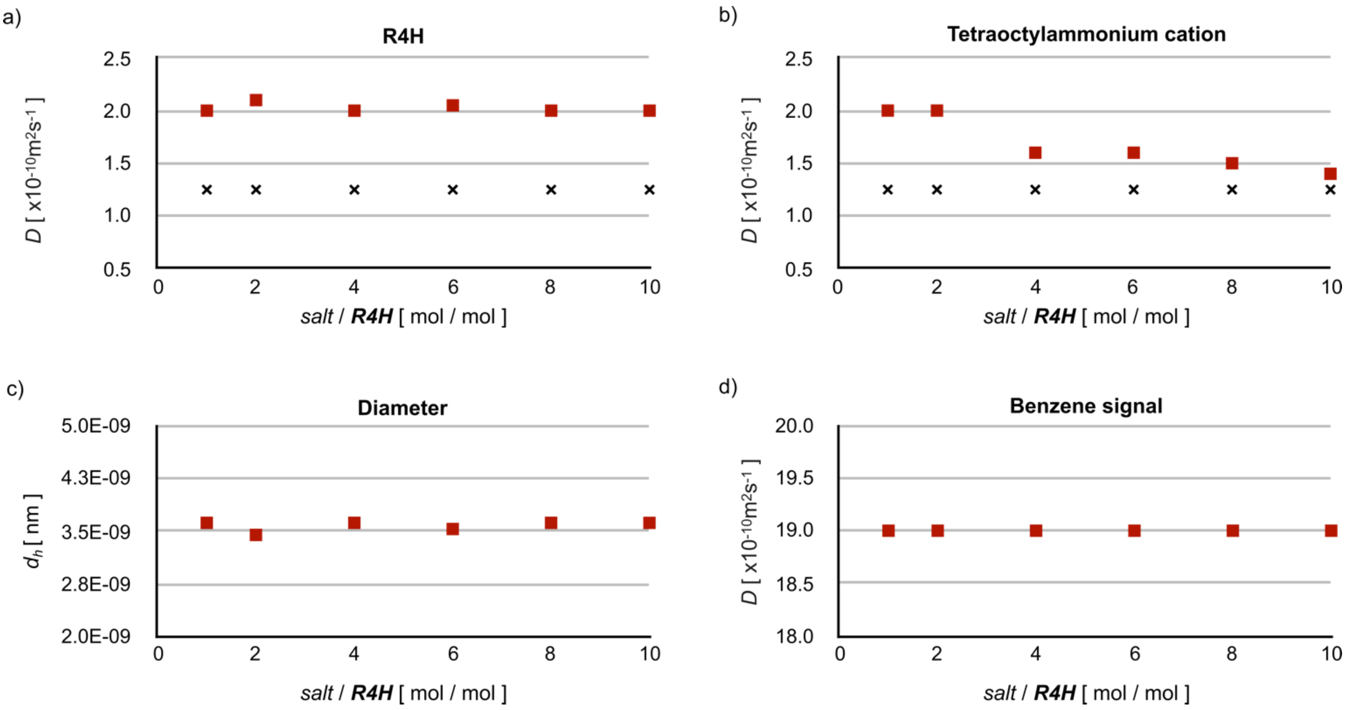

Figure S91. Solid sample of **R4H** (various rations from 0.04 mol to 0.005 mol) and **Oct_4NBr** (0.04 mol) were ball-milled (1h dry-milling in a planetary ball-mill) and the powders were dissolved in benzene-d_6 (0.7 ml). The sample was filtered and the solution was checked by ^{1}H NMR. Diffusion coefficient changes: for (a) **R4H;** (b) **Oct_4NBr**; (c) diameter of complex; (d) benzene (600 MHz, 303 K, benzene-d_6). **x**- free salt sygnal (0.04 mmol)

R4H	Oct_4N^+	salt / M [mol / mol]
2.0	2.0	1
2.1	2.0	2
2.0	1.6	4
2.1	1.6	6
2.0	1.5	8
2.0	1.4	10

Table S14. Data for DOSY titration (sample preparation as above).

9.16 R4C with Hex_4NCl

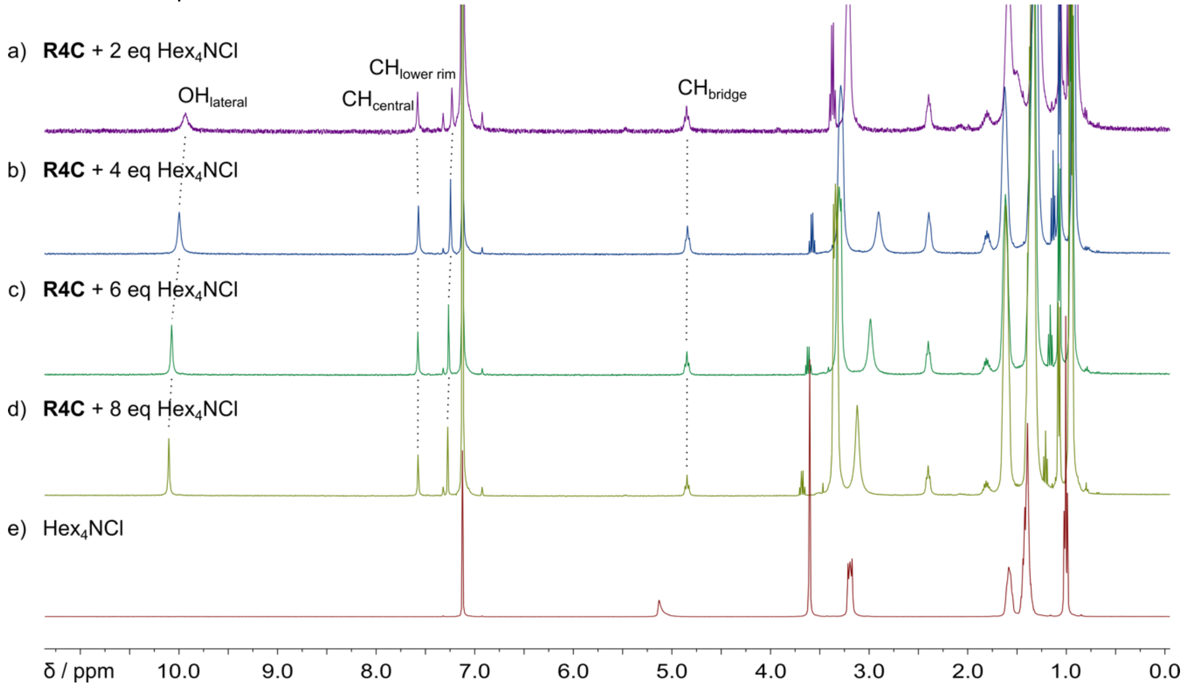

Figure S92. ^{1}H NMR of mechanochemically treated samples. Solid sample of **R4C** and **Hex_4NCl** were ball-milled (1h dry-milling in a planetary ball-mill) and the powders were dissolved in benzene-d_6 (0.7 ml) and then filtered. ^{1}H NMR spectrum of (a) **R4C** (20 mmol) **+ Hex_4NCl** (40 mmol); (b) **R4C** (10 mmol) **+ Hex_4NCl** (40 mmol); (c) **R4C** (6.7 mmol) **+ Hex_4NCl** (40 mmol); (d) **R4C** (5.0 mmol) **+ Hex_4NCl** (40 mmol); (e) reference sample **Hex_4NCl** (400 MHz, 303 K).

9.17 R4C with Oct_4NCl

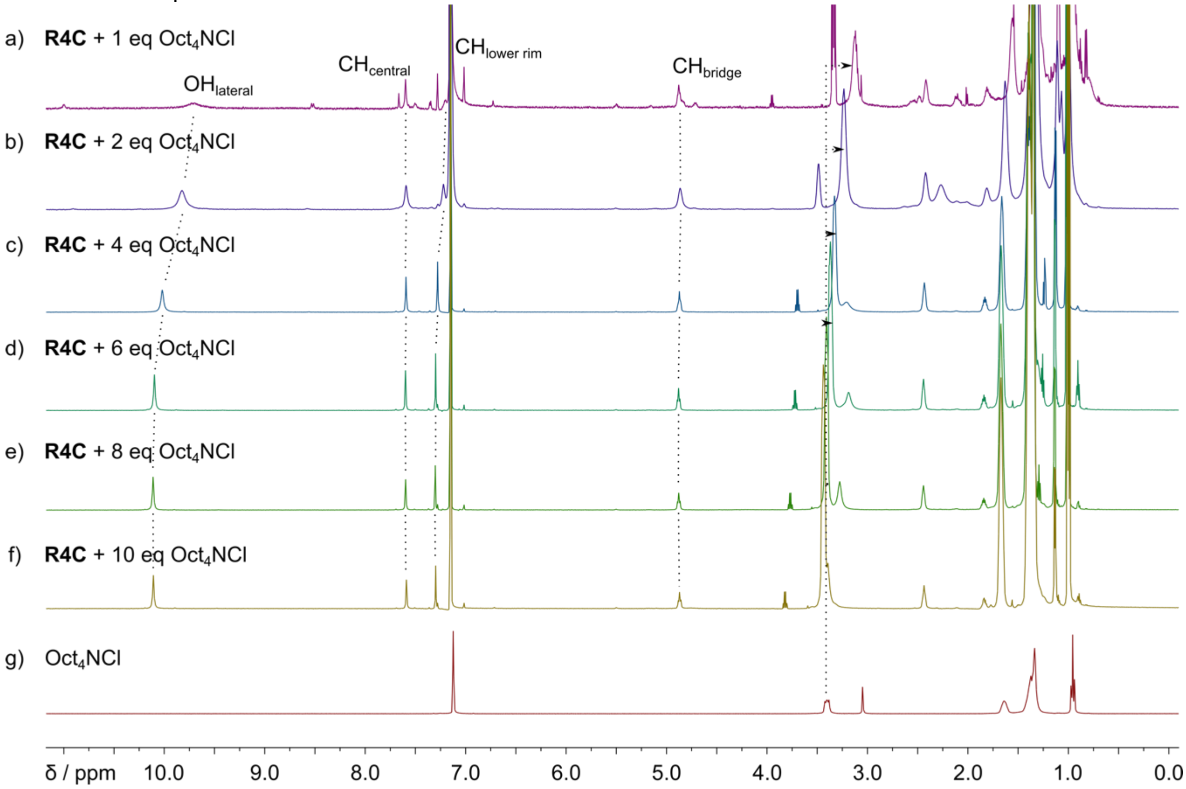

Figure S93. ^{1}H NMR of mechanochemically treated samples. Solid sample of **R4C** and **Oct_4NCl** were ball-milled (1h dry-milling in a planetary ball-mill) and the powders were dissolved in benzene-d_6 (0.7 ml) and then filtered. ^{1}H NMR spectrum of (a) **R4C** (40 mmol) **+ Oct_4NCl** (40 mmol); (b) **R4C** (20 mmol) **+ Oct_4NCl** (40 mmol); (c) **R4C** (10 mmol) **+ Oct_4NCl** (40 mmol); (d) **R4C** (6.7 mmol) **+ Oct_4NCl** (40 mmol); (e) **R4C** (5.0 mmol) **+ Oct_4NCl** (40 mmol); (f) **R4C** (4.0 mmol) **+ Oct_4NCl** (40 mmol); (g) reference sample **Oct_4NCl** (400 MHz, 303 K).

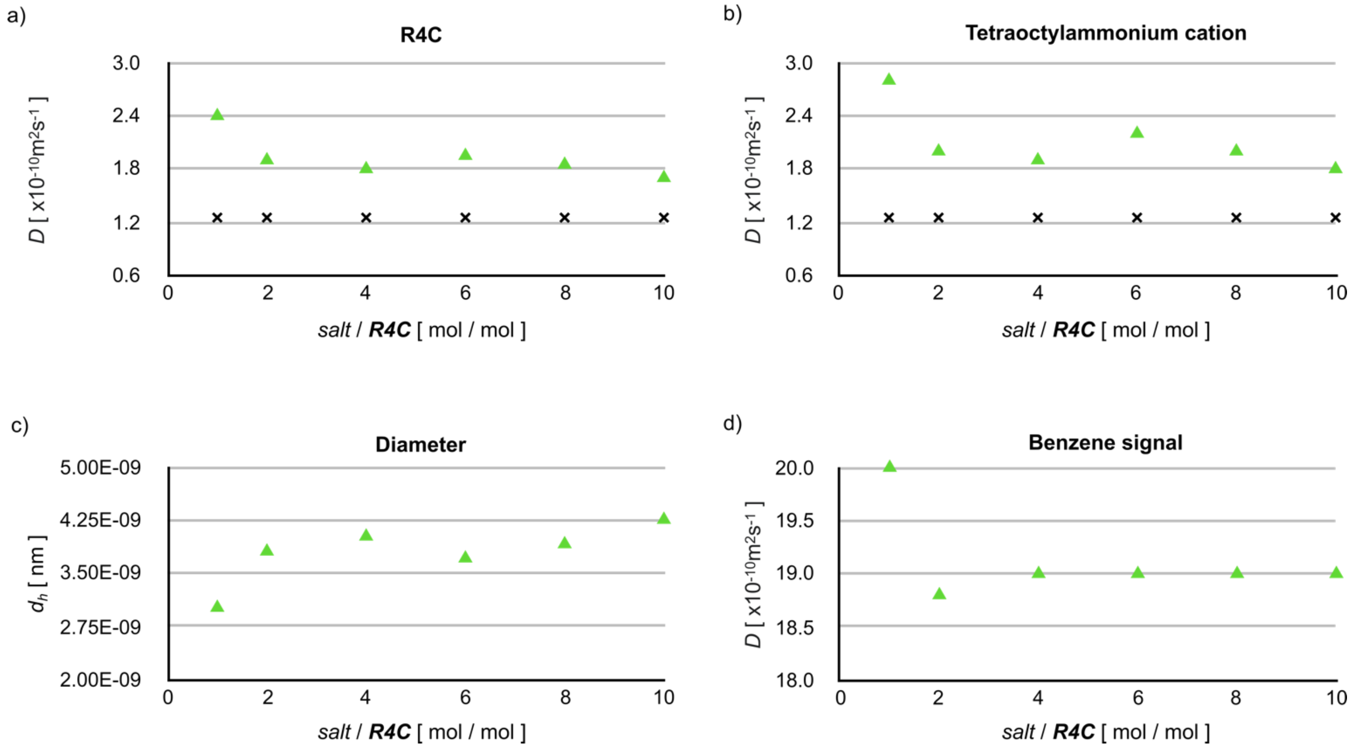

Figure S94. Solid sample of **R4C** (various rations from 0.04 mol to 0.005 mol) and **Oct_4NCl** (0.04 mol) were ball-milled (1h dry-milling in a planetary ball-mill) and the powders were dissolved in benzene-d_6 (0.7 ml). The sample was filtered and the solution was checked by ^1H NMR. Diffusion coefficient changes: for (a) **R4C;** (b) **Oct_4NCl**; (c) diameter of complex; (d) benzene (600 MHz, 303 K, benzene-d_6). **x**- free salt sygnal (0.04 mmol)

R4C	Oct_4N^+	salt / M [mol / mol]
2.4	2.8	1
1.9	2.0	2
1.8	1.9	4
2.0	2.2	6
1.9	2.0	8
1.7	1.8	10

Table S15. Data for DOSY titration (sample preparation as above).

9.18 R4C with Pen_4NBr

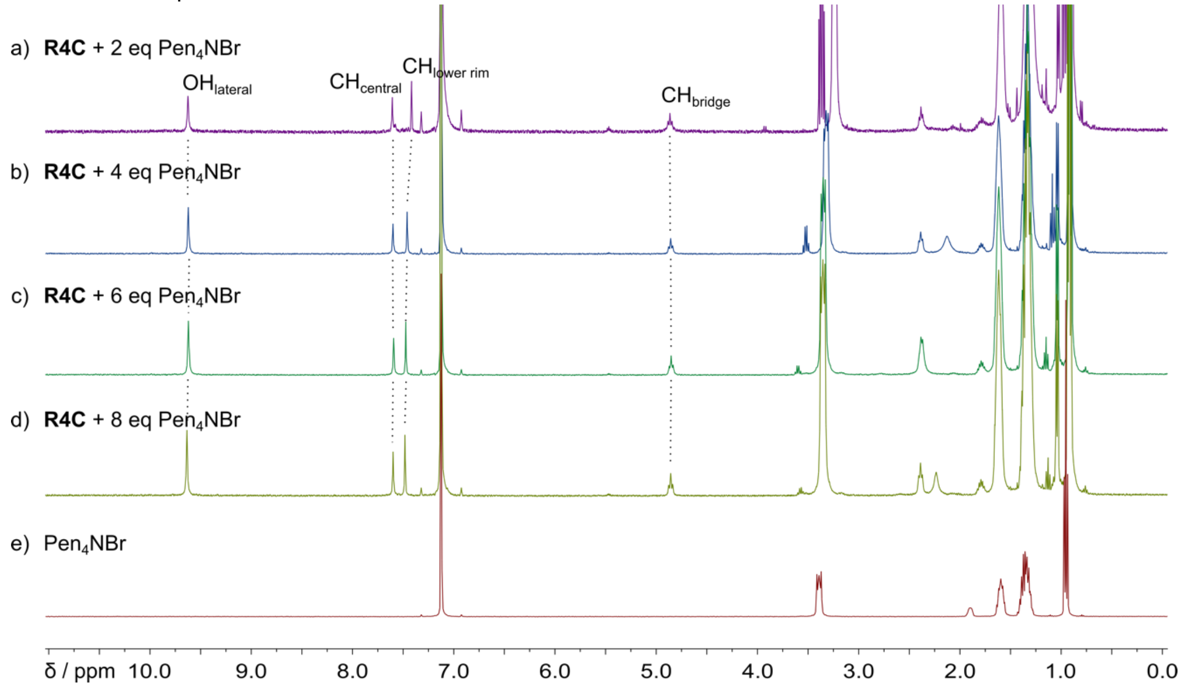

Figure S95. ^{1}H NMR of mechanochemically treated samples. Solid sample of **R4C** and **Pen_4NBr** were ball-milled (1h dry-milling in a planetary ball-mill) and the powders were dissolved in benzene-d_6 (0.7 ml) and then filtered. ^{1}H NMR spectrum of (a) **R4C** (20 mmol) **+ Pen_4NBr** (40 mmol); (b) **R4C** (10 mmol) **+ Pen_4NBr** (40 mmol); (c) **R4C** (6.7 mmol) **+ Pen_4NBr** (40 mmol); (d) **R4C** (5.0 mmol) **+ Pen_4NBr** (40 mmol); (e) reference sample **Pen_4NBr** (400 MHz, 303 K).

9.19 R4C with Hex_4NBr

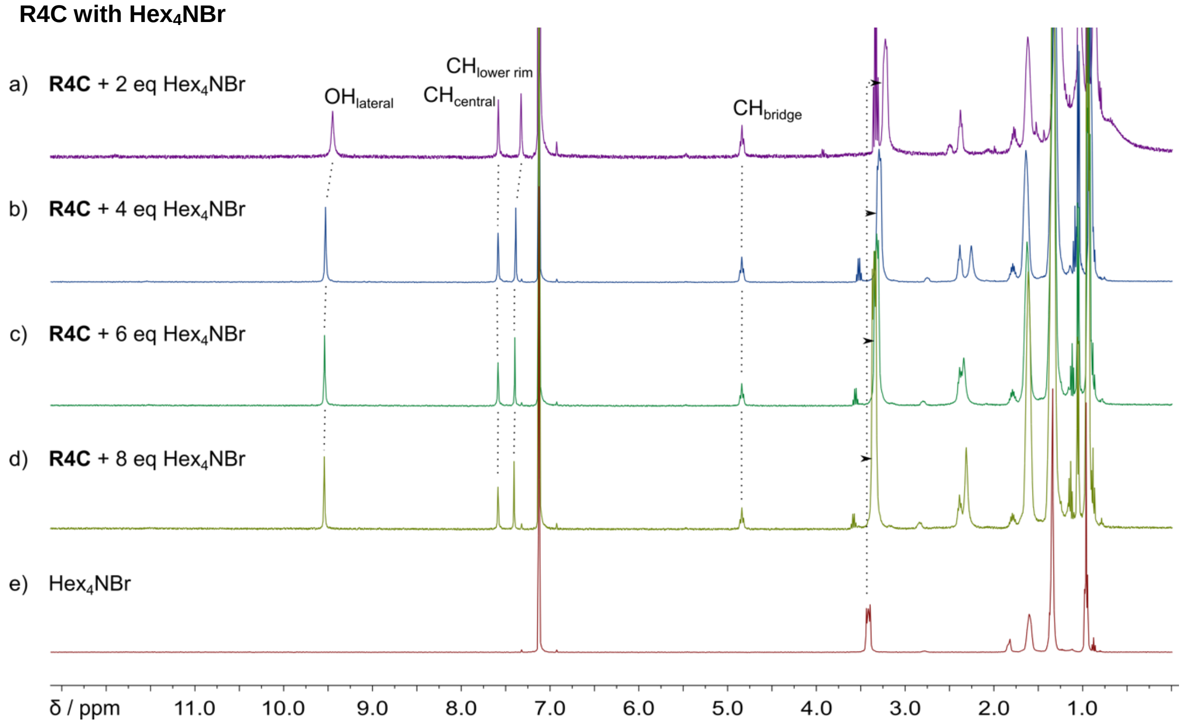

Figure S96. ^{1}H NMR of mechanochemically treated samples. Solid sample of **R4C** and **Hex_4NBr** were ball-milled (1h dry-milling in a planetary ball-mill) and the powders were dissolved in benzene-d_6 (0.7 ml) and then filtered. ^{1}H NMR spectrum of (a) **R4C** (20 mmol) **+ Hex_4NBr** (40 mmol); (b) **R4C** (10 mmol) **+ Hex_4NBr** (40 mmol); (c) **R4C** (6.7 mmol) **+ Hex_4NBr** (40 mmol); (d) **R4C** (5.0 mmol) **+ Hex_4NBr** (40 mmol); (e) reference sample **Hex_4NBr** (400 MHz, 303 K).

9.20 R4C with Oct_4NBr

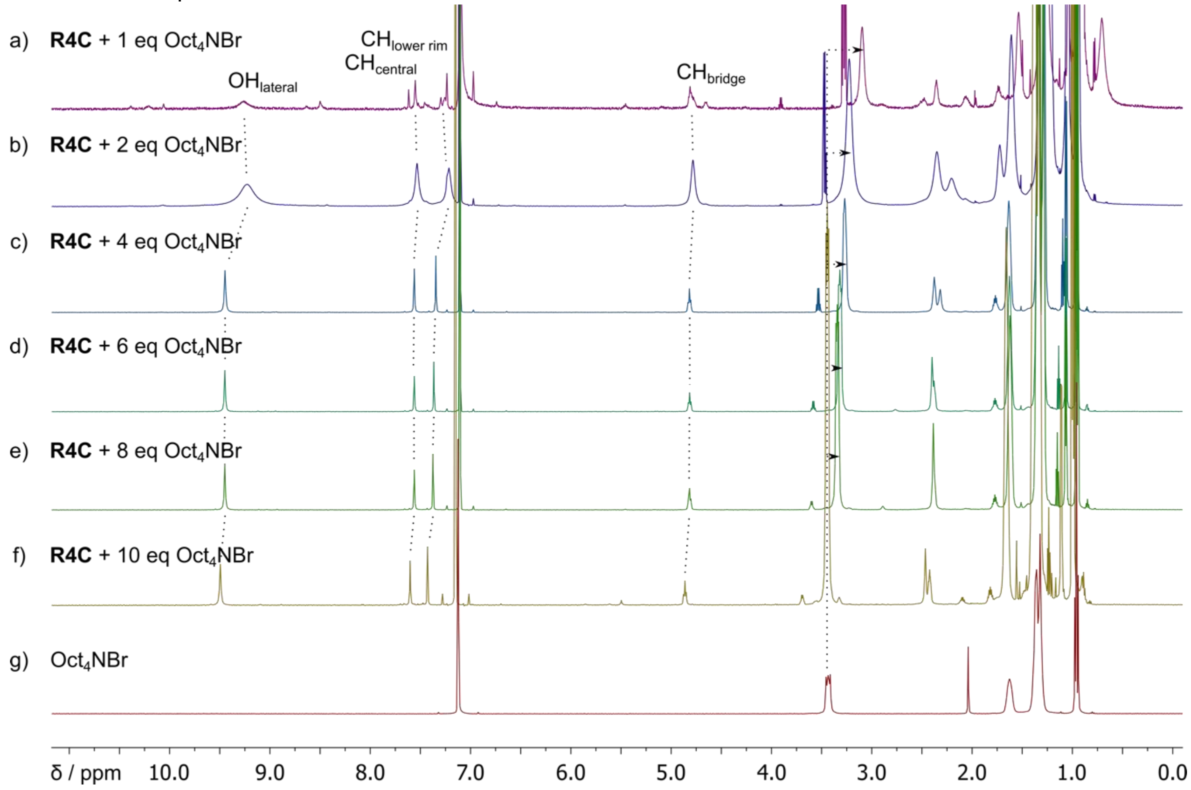

Figure S97. ^{1}H NMR of mechanochemically treated samples. Solid sample of **R4C** and **Oct_4NBr** were ball-milled (1h dry-milling in a planetary ball-mill) and the powders were dissolved in benzene-d_6 (0.7 ml) and then filtered. ^{1}H NMR spectrum of (a) **R4C** (40 mmol) **+ Oct_4NBr** (40 mmol); (b) **R4C** (20 mmol) **+ Oct_4NBr** (40 mmol); (c) **R4C** (10 mmol) **+ Oct_4NBr** (40 mmol); (d) **R4C** (6.7 mmol) **+ Oct_4NBr** (40 mmol); (e) **R4C** (5.0 mmol) **+ Oct_4NBr** (40 mmol); (f) **R4C** (4.0 mmol) **+ Oct_4NBr** (40 mmol); (g) reference sample **Oct_4NBr** (400 MHz, 303 K).

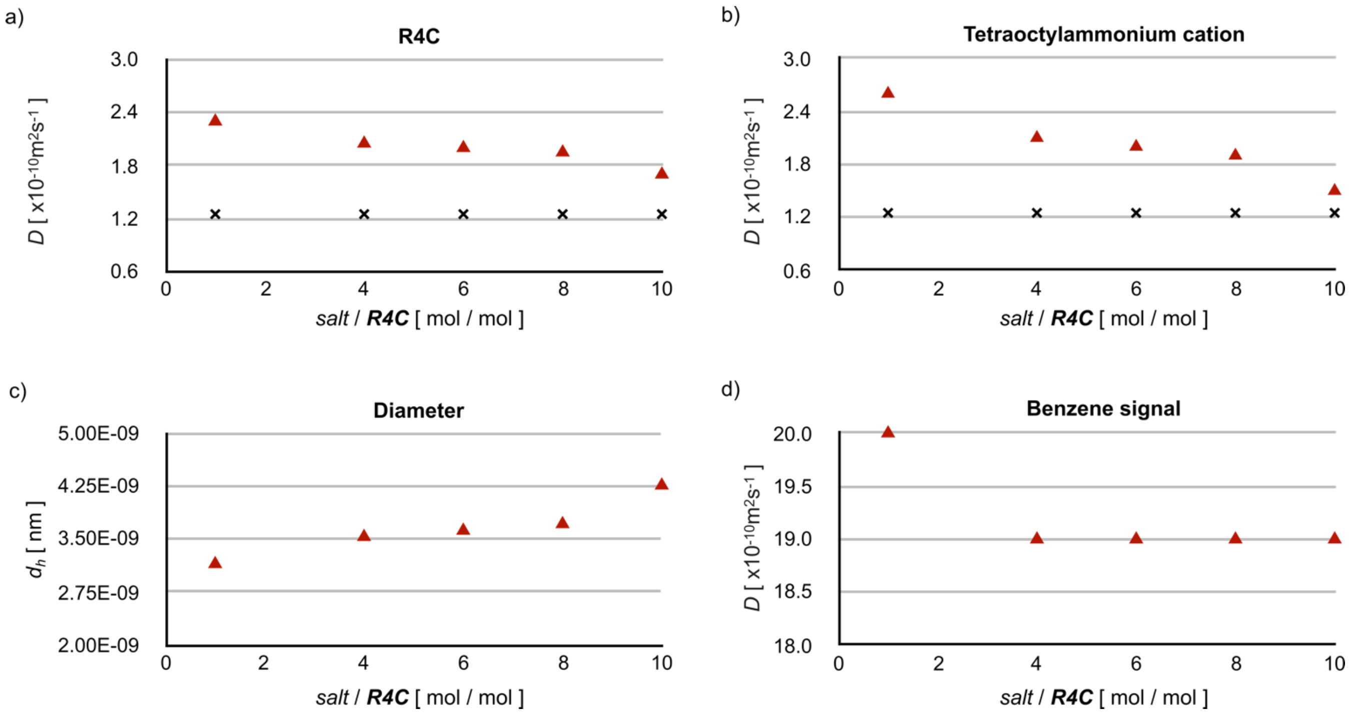

Figure S98. Solid sample of **R4C** (various rations from 0.04 mol to 0.005 mol) and **Oct_4NBr** (0.04 mol) were ball-milled (1h dry-milling in a planetary ball-mill) and the powders were dissolved in benzene-d_6 (0.7 ml). The sample was filtered and the solution was checked by ^{1}H NMR. Diffusion coefficient changes: for (a) **R4C;** (b) **Oct_4NBr**; (c) diameter of complex; (d) benzene (600 MHz, 303 K, benzene-d_6). **x**- free salt sygnal (0.04 mmol)

R4C	Oct_4N^+	salt / M [mol / mol]
2.0	2.0	1
2.1	2.0	2
2.0	1.6	4
2.0	1.6	6
2.0	1.5	8
2.0	1.4	10

Table S16. Data for DOSY titration (sample preparation as above).

10. Titrations in chloroform (P4C)

10.1 ^{1}H NMR spectra interaction of P4C with But$_4$NCl in chloroform

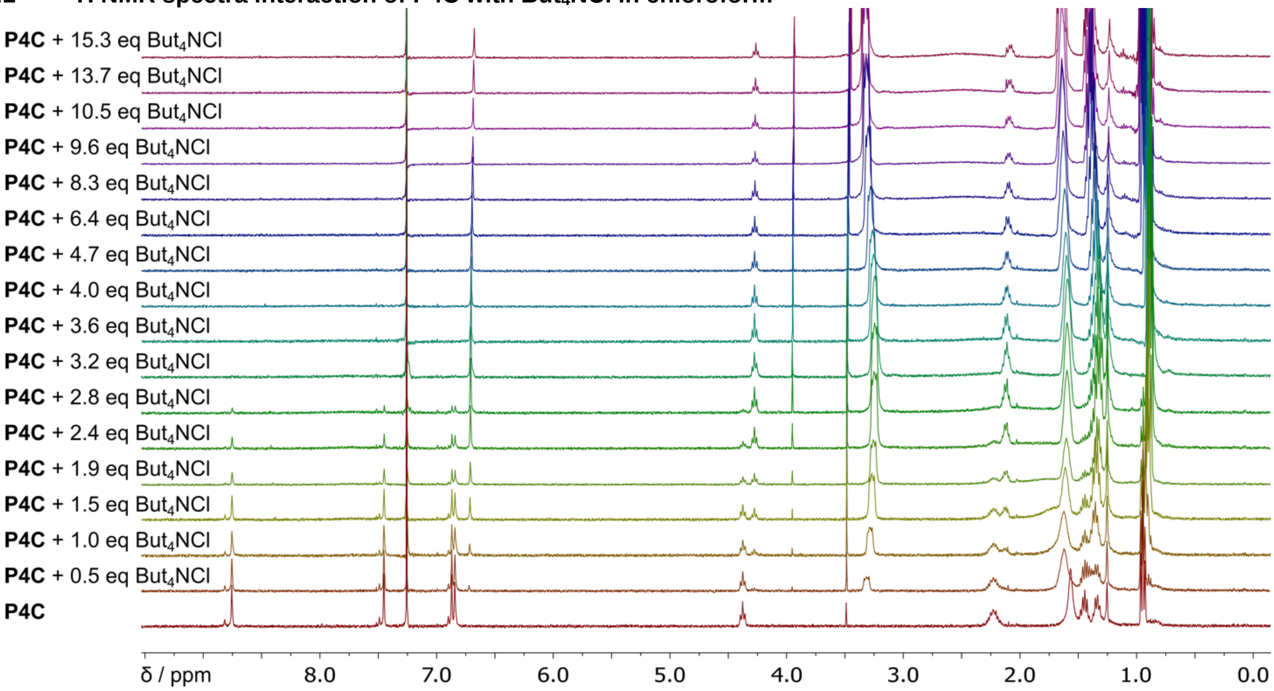

Figure S99. ^{1}H NMR spectra of titration of **P4C** (C = 2.5 mM) with titrant **P4C** (C = 2.5 mM) + **But$_4$NCl** (C = 65.6 mM) (400 MHz, 303 K, $CDCl_3$).

10.2 ^{1}H NMR spectra interaction of P4C with But$_4$NBr in chloroform

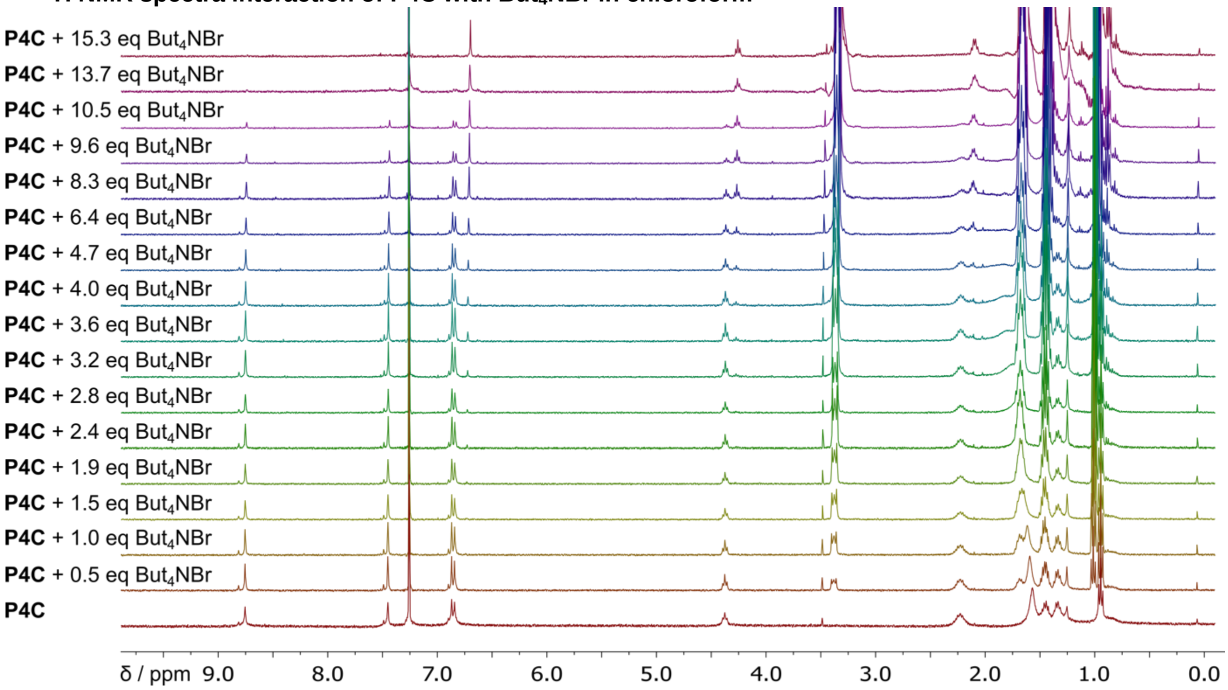

Figure S100. ^{1}H NMR spectra of titration of **P4C** (C = 2.5 mM) with titrant **P4C** (C = 2.5 mM) + **But$_4$NBr** (C = 65.6 mM) (400 MHz, 303 K, $CDCl_3$).

11. Data comparison

11.1 The influence of the type of anion on the size of anion-sealed species (in THF, DOSY)

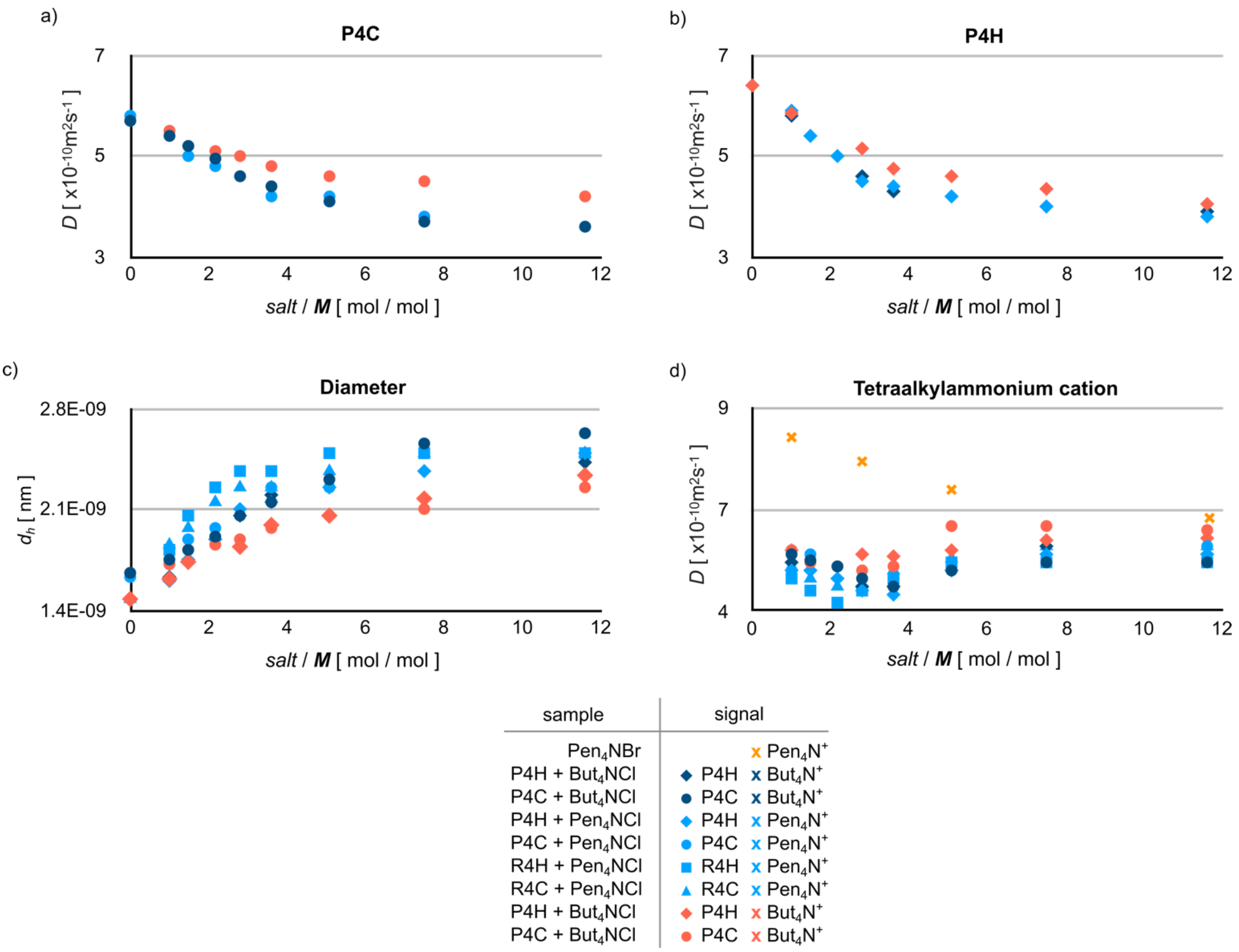

Figure S101. DOSY titrations of macrocycles (M) **P4H**, **P4C**, **R4H** and **R4C** with Alk_4NX salts in THF-d_8: (a) changes of diffusion coefficients (D) for signals of **P4C** with **But_4NCl**, **But_4NBr** and **Pen_4NCl**; (b) changes of diffusion coefficients (D) for signals **P4H** with **But_4NCl**, **But_4NBr** and **Pen_4NCl**; (e) diameter of complexes (d) changes of diffusion coefficients (D) for signals of Alk_4N^+. All titrations were performed using solutions of analyte: C (M) = 2.5 mM and titrant: C(M)=2.5 mM + C(Alk_4N_X) = 65 mM at 298 K, 600 MHz.

11.2 The influence of the macrocycles' lower rim substituents on formation of anion sealed species (in THF, ^{1}H NMR)

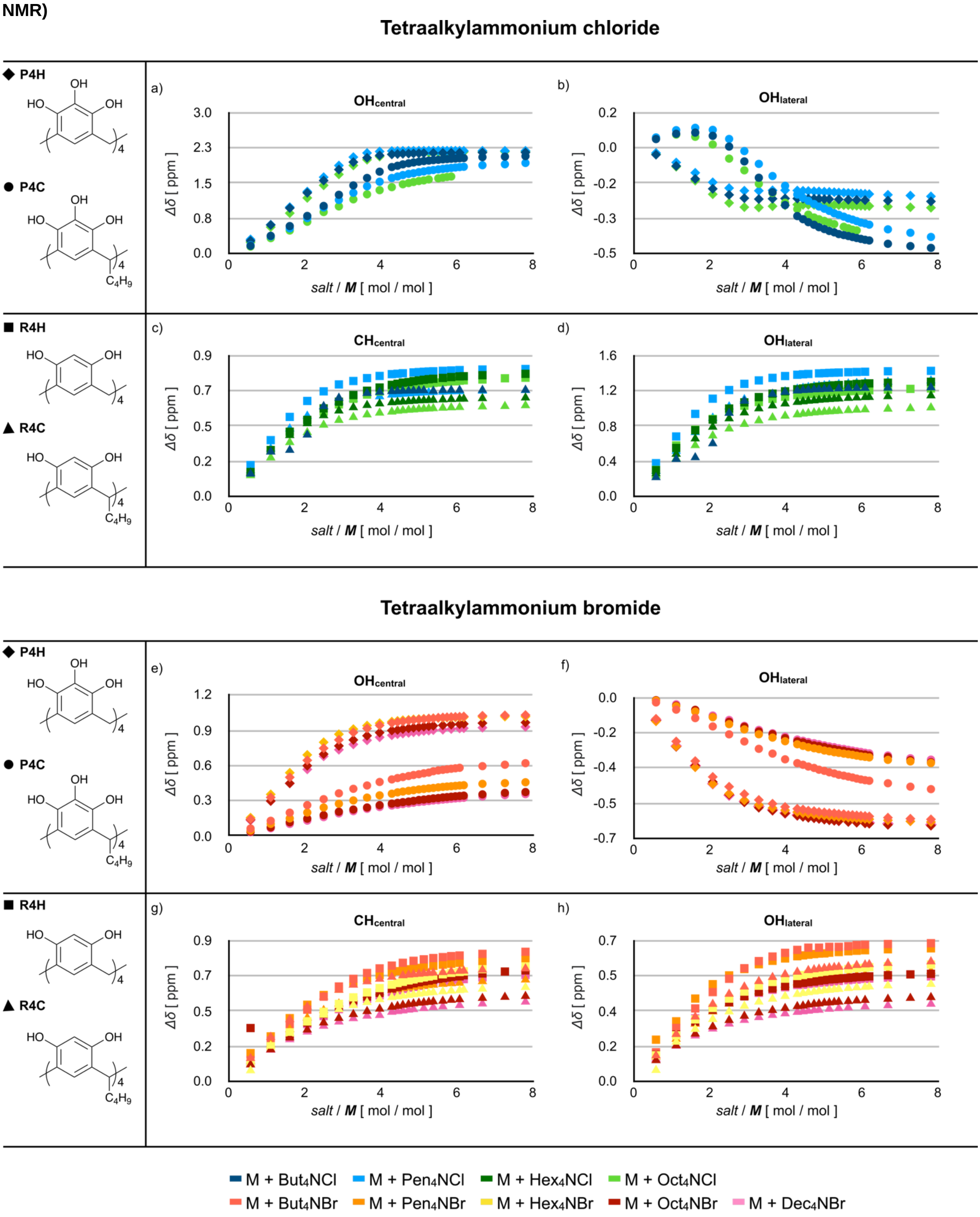

Figure S102. ^{1}H NMR titrations of macrocycles (M) **P4H**, **P4C**, **R4H** and **R4C** with Alk_4NX salts in THF-d_8. Changes of chemical shifts for (a) **P4H** and **P4C** $OH_{central}$ signals during titration with Alk_4NCl; (b) **P4H** and **P4C** $OH_{lateral}$ signals during titration with Alk_4NCl; (c) **R4H** and **R4C** $OH_{central}$ signals during titration with Alk_4NCl; (d) **R4H** and **R4C** $OH_{lateral}$ signals during titration with Alk_4NCl; (e) **P4H** and **P4C** $OH_{central}$ signals during titration with Alk_4NBr; (f) **P4H** and **P4C** $OH_{lateral}$ signals during titration with Alk_4NBr; (g) **R4H** and **R4C** $OH_{central}$ signals during titration with Alk_4NBr; (h) **P4H** and **P4C** OHlateral signals during titration with Alk_4NBr. All titrations were performed using solutions of analyte: C (M) = 5.0 mM and titrant: C(M)= 5.0 mM + C(Alk_4NX) = 75 mM at 303 K, 400 MHz.

11.3 The relative stability (normalized, ^{1}H NMR, THF)

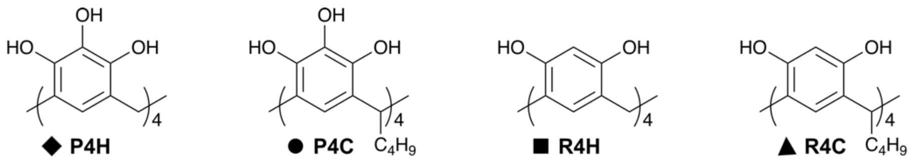

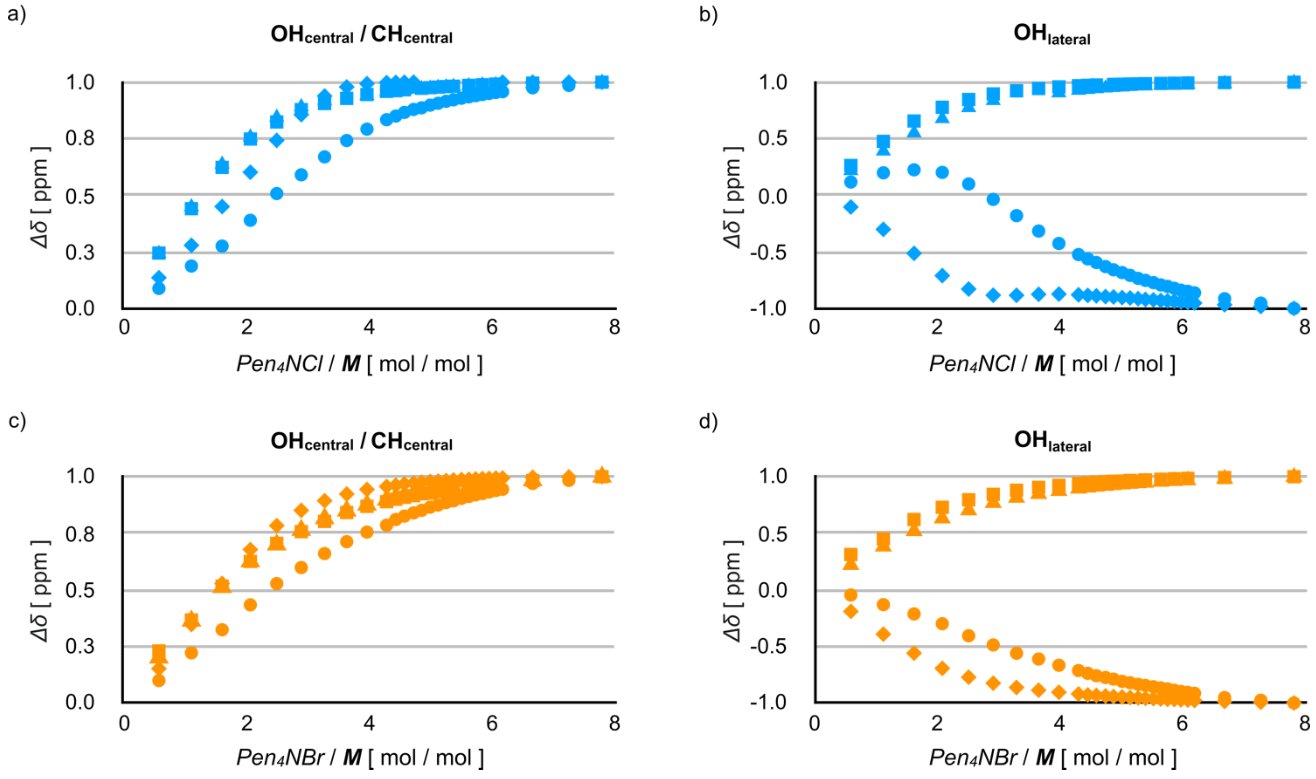

Figure S103. ^{1}H NMR titrations of macrocycles (M) **P4H**, **P4C**, **R4H** and **R4C** with **Pen_4NCl** and **Pen_4NBr** in THF-d_8. Changes of chemical shifts for (a) OH_{cental}/$CH_{central}$ signals during titration with **Pen_4NCl**; (b) $OH_{lateral}$ signals during titration with Pen_4NCl; (c) OH_{cental}/$CH_{central}$ signals during titration with **Pen_4NBr**; (d) $OH_{lateral}$ signals during titration with **Pen_4NBr**. All titrations were performed using solutions of analyte: C (M) = 5.0 mM and titrant: C(M)= 5.0 mM + C(Alk4NX) = 75 mM at 303 K, 400 MHz.

11.4 The influence of the type of macrocycle on interactions of anion-sealed species with cations (in THF, ^{1}H NMR)

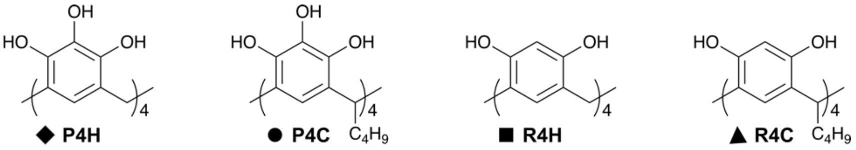

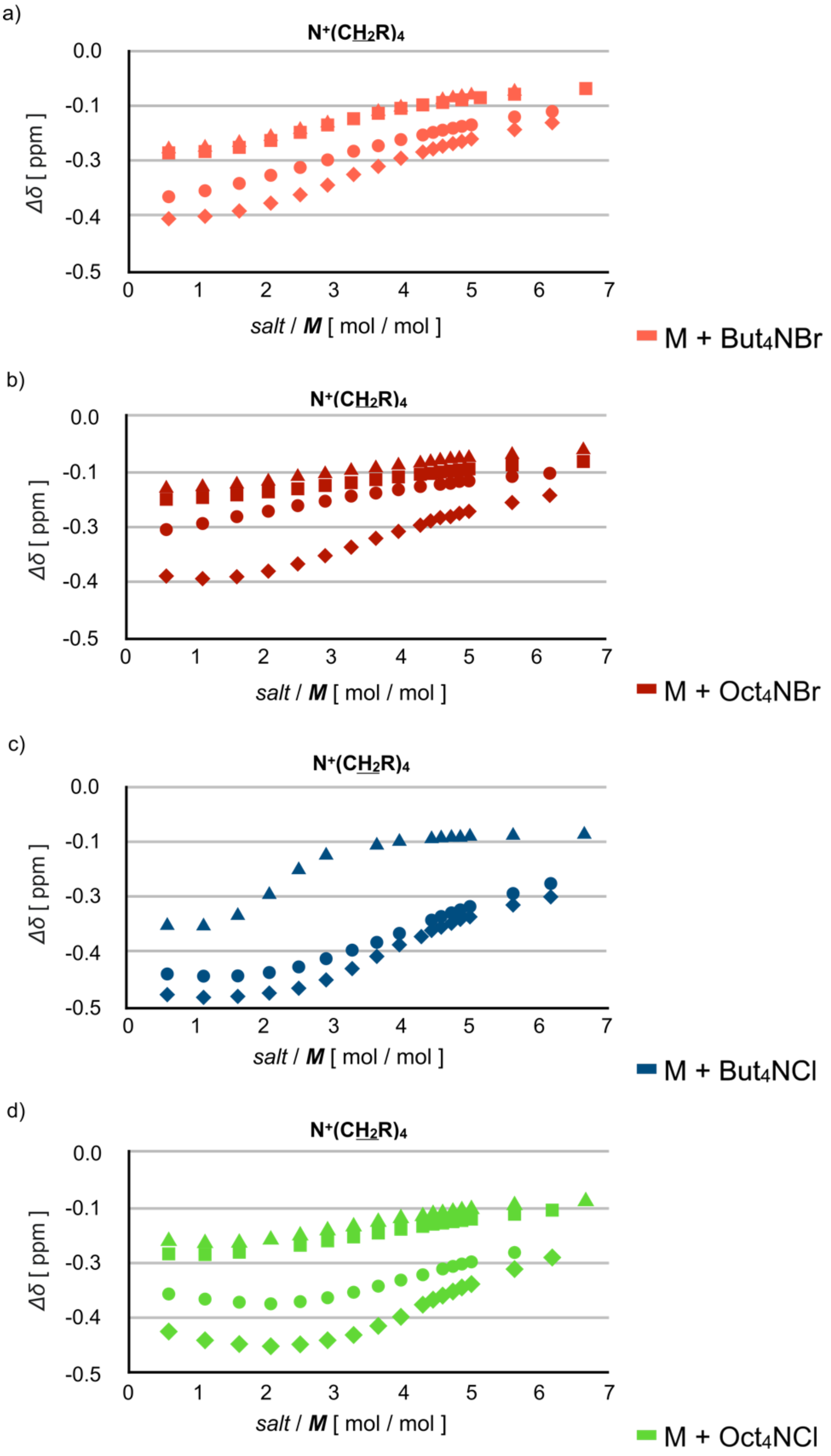

Figure S104. Changes of ^{1}H chemical shifts of -C$\underline{H}_2$N$^+$ signal during titration of **P4H, P4C, R4C and R4C** with (a) **But$_4$NBr** (b) **Oct$_4$NBr** (c) **But$_4$NCl** (d) **Oct$_4$NCl**. All titrations were performed using solutions of analyte: C (M) = 5.0 mM and titrant: C(M)= 5.0 mM + C(Alk$_4$NX) = 75 mM at 303 K, 400 MHz.

11.5 The influence of the type of cations on interaction of anion-sealed species with cations (in THF, ^{1}H NMR)

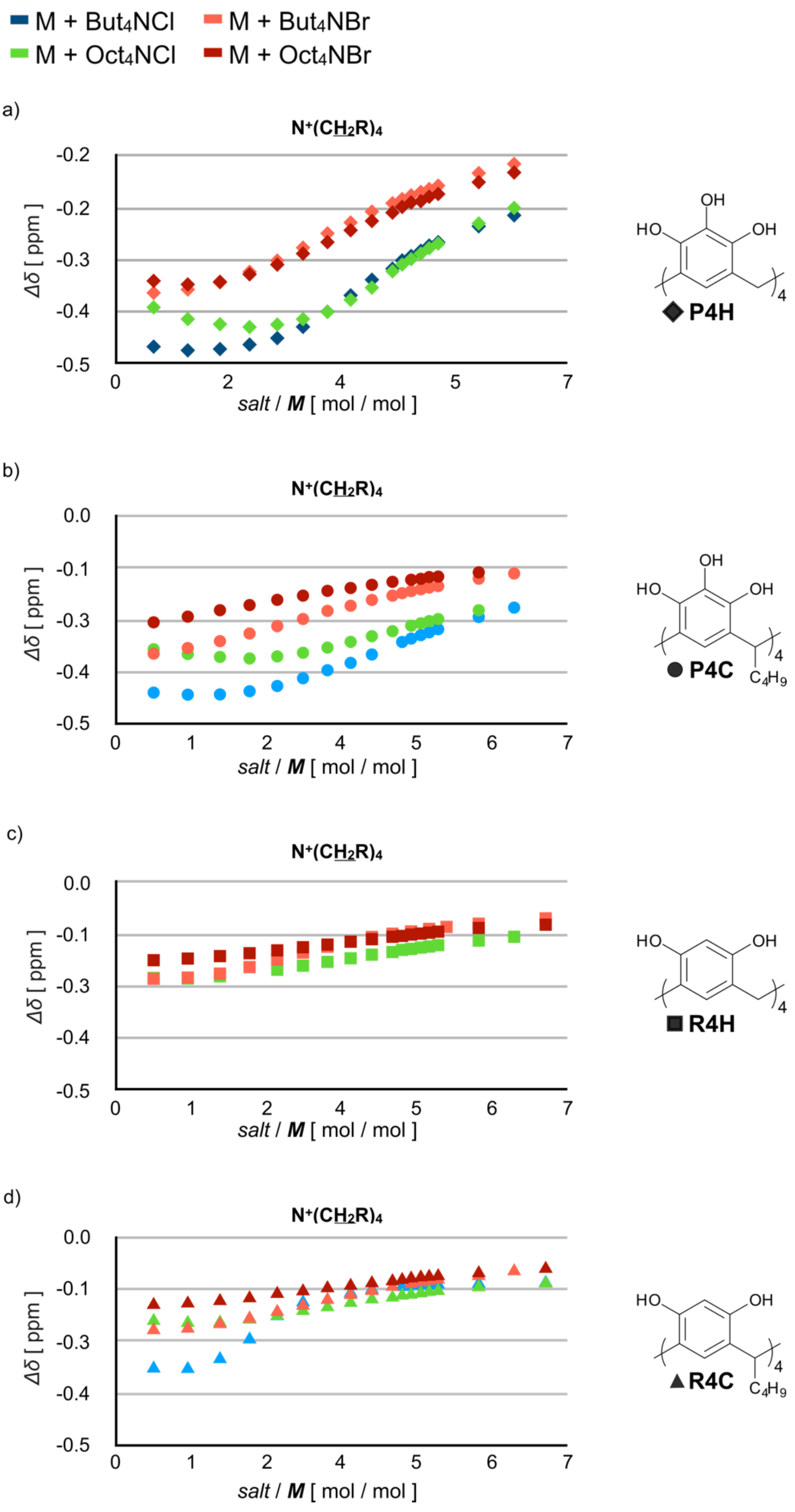

Figure S105. Changes of ^{1}H chemical shifts of -C$\underline{H}_2$N^{+} signal during titration of (a) **P4H**; (b) **P4C,** (c) **R4C** (d) **R4C** with **But$_4$NBr**, **Oct$_4$NBr**, **But$_4$NCl** and **Oct$_4$NCl** in THF-d$_8$. All titrations were performed using solutions of analyte: C (M) = 5.0 mM and titrant: C(M)= 5.0 mM + C(Alk$_4$NX) = 75 mM at 303 K, 400 MHz.

12. Titrations of pyrogallol

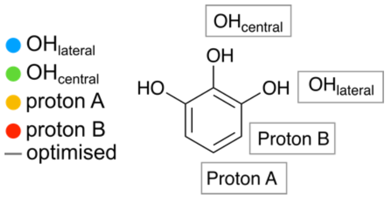

- **THF**

a)

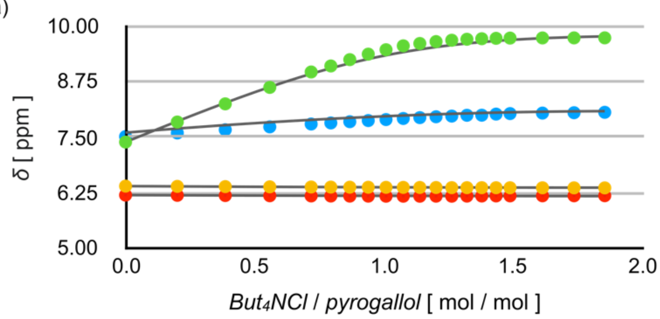

complex	constant	optimised	error
RCl	K_{11}	**2160 M^{-1}**	**± 8.6%**

b)

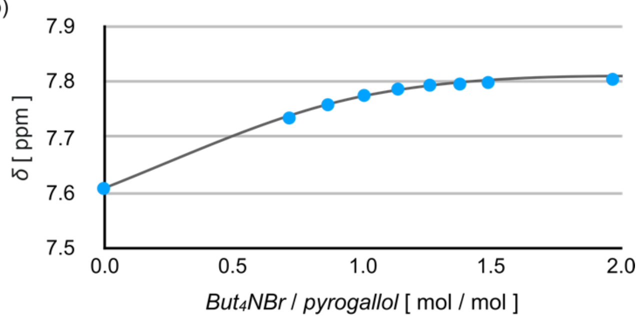

complex	constant	optimised	error
RBr	K_{11}	**1219 M^{-1}**	**± 9.7%**

- **acetonitrile**

c)

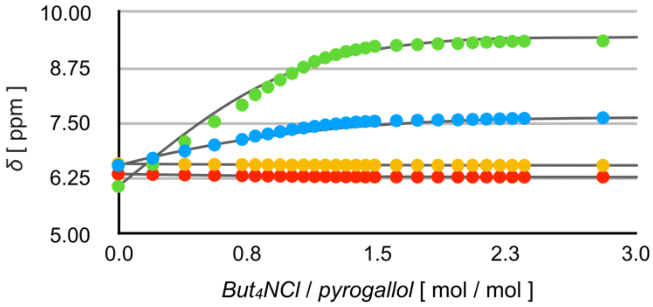

complex	constant	optimised	error
RCl	K_{11}	**655 M^{-1}**	**± 6.0%**

d)

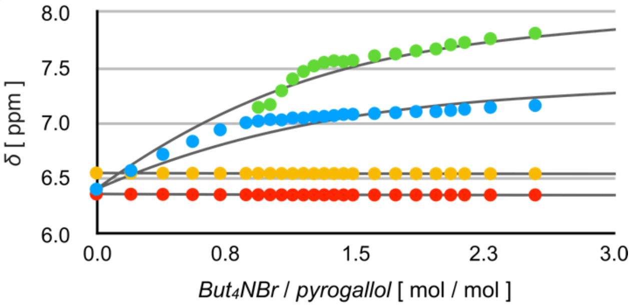

complex	constant	optimised	error
RBr	K_{11}	**139 M^{-1}**	**± 6.5%**

- **chloroform**

e)

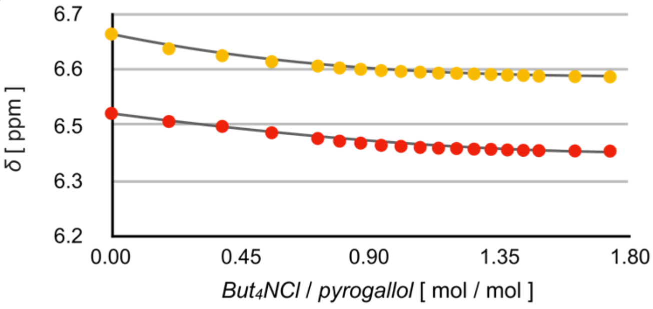

complex	constant	optimised	error
RCl	K_{11}	**1219 M^{-1}**	**± 9.1%**
R_2Cl	K_{21}	**35 M-1**	**± 15%**

f)

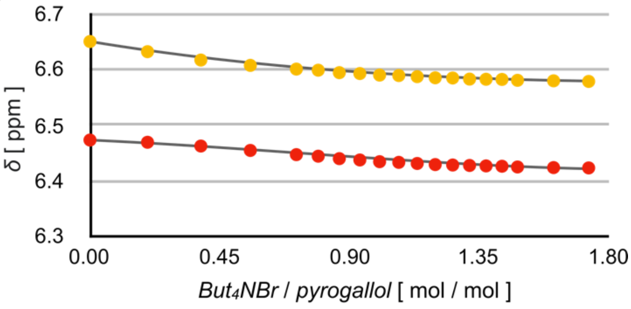

complex	constant	optimised	error
RBr	K_{11}	**877 M^{-1}**	**± 7.3%**
R_2Br	K_{21}	**80 M^{-1}**	**± 10%**

Figure S106. Titrations of pyrogallol with (a) **But_4NCl** in THF-d_8, (b) **But_4NBr** in THF-d_8, (c) **But_4NCl** in MeCN-d_3, (d) **But_4NBr** in MeCN-d_3, (e) **But_4NCl** in $CDCl_3$, (f) **But_4NBr** in $CDCl_3$. Solid lines represent fitted curves, dahed line represents theoretical curve for 1:1. All titrations were performed using solutions of analyte: C (pyrogallol) = 15 mM and titrant: C(pyrogallol)= 15 mM + C(Alk_4NX) = 78 mM at 303 K, 400 MHz.

	pyrogallol								
	THF			acetonitrile			chloroform		
	RCl	RCl_2	R_2Cl	RCl	RCl_2	R_2Cl	RCl	RCl_2	R_2Cl
	K_{11} [M^{-1}]	K_{12} [M^{-1}]	K_{21} [M^{-1}]	K_{11} [M^{-1}]	K_{12} [M^{-1}]	K_{21} [M^{-1}]	K_{11} [M^{-1}]	K_{12} [M^{-1}]	K_{21} [M^{-1}]
**But_4NCl**	**2160**	-	-	**655**	-	-	**1219**	-	**35**
error	± 8.6%	-	-	±6.0%	-	-	±9.1%	-	± 15.0%
**But_4NBr**	**1219**	-	-	**139**	-	-	**877**	-	**79**
error	± 9.7%	-	-	±6.5%	-	-	±7.3%	-	± 10.0%

Table S1. Comparison of the stability constant of the pyrogallol complexes in various solvents (THF, acetonitrile, chloroform).

13. Titrations of resorcinol

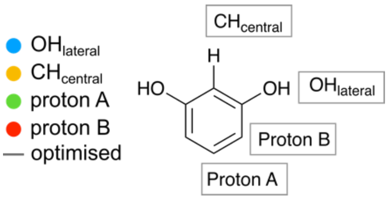

• **THF**

a)

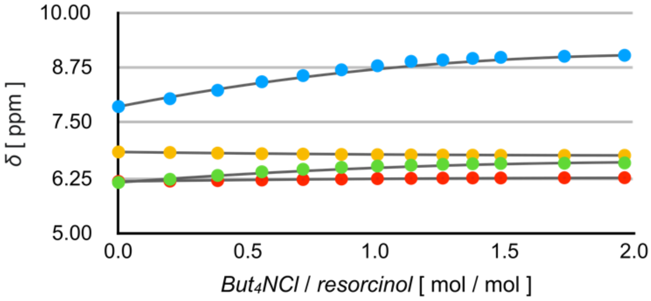

complex	constant	optimised	error
RCl	K_{11}	**670 M^{-1}**	**±7.5%**

b)

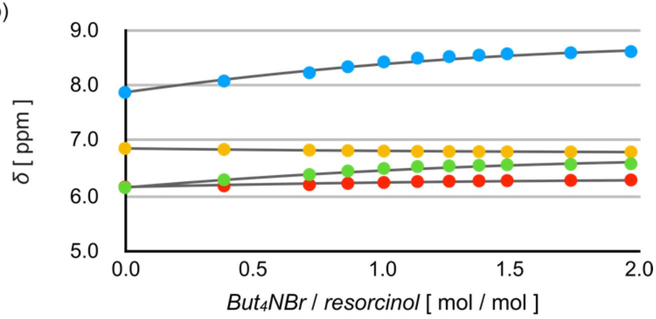

complex	constant	optimised	error
RBr	K_{11}	**163 M^{-1}**	**±7.0%**

• **acetonitrile**

c)

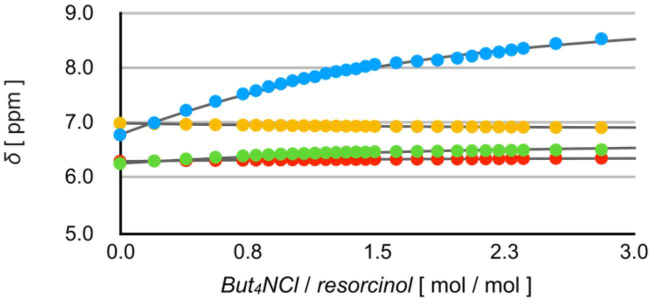

complex	constant	optimised	error
RCl	K_{11}	**71 M^{-1}**	**±1.4%**

d)

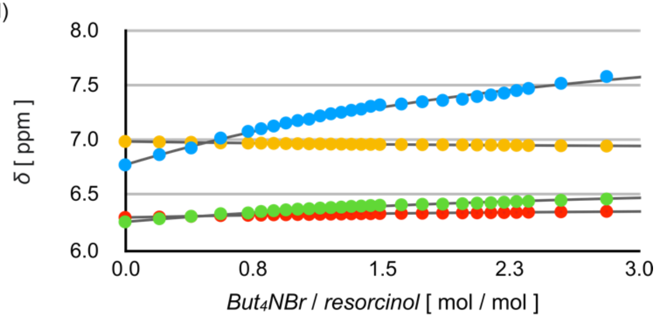

complex	constant	optimised	error
RBr	K_{11}	**36 M^{-1}**	**±1.3%**

• **chloroform**

e)

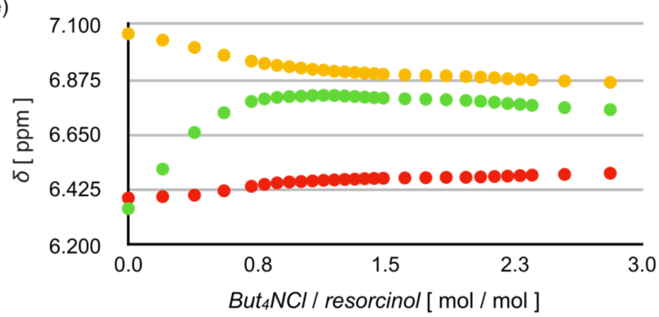

f)

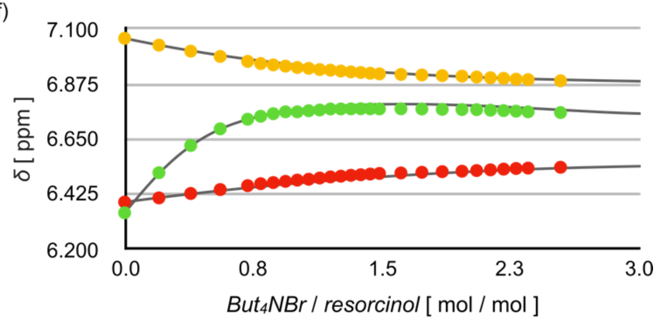

complex	constant	optimised	error
RBr	K_{11}	**272 M^{-1}**	**±1.7%**
R_2Br	K_{21}	**18 M^{-1}**	**±0.1%**

Figure S107. Titrations of resorcinol with (a) **But_4NCl** in THF-d_8, (b) **But_4NBr** in THF-d_8, (c) **But_4NCl** in MeCN-d_3, (d) **But_4NBr** in MeCN-d_3, (e) **But_4NCl** in $CDCl_3$, (f) **But_4NBr** in $CDCl_3$. Solid lines represent fitted curves, dahed line represents theoretical curve for 1:1. All titrations were performed using solutions of analyte: C (resorcinol) = 15 mM and titrant: C(resorcinol)= 15 mM + C(Alk_4NX) = 78 mM at 303 K, 400 MHz.

	resorcinol								
	THF			acetonitrile			Chloroform		
	RCl	RCl_2	R_2Cl	RCl	RCl_2	R_2Cl	RCl	RCl_2	R_2Cl
	K_{11} [M^{-1}]	K_{12} [M^{-1}]	K_{21} [M^{-1}]	K_{11} [M^{-1}]	K_{12} [M^{-1}]	K_{21} [M^{-1}]	K_{11} [M^{-1}]	K_{12} [M^{-1}]	K_{21} [M^{-1}]
**But_4NCl**	**670**	-	-	**71**	-	-	**286**	-	-
error	±7.5%	-	-	±1.4%	-	-	±4.0%	-	-
**But_4NBr**	**164**	-	-	**36**	-	-	**272**	-	**18**
error	±7.0%	-	-	±1.3%	-	-	±1.7%	-	±0.1%

Table S2. Comparison of the stability constant of the resorcinol complexes in various solvents (THF, acetonitrile, chloroform).

14. Titrations of pyrocatechol

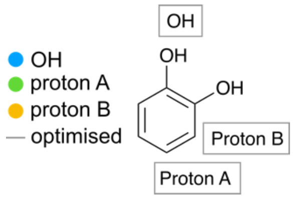

- **THF**

a)

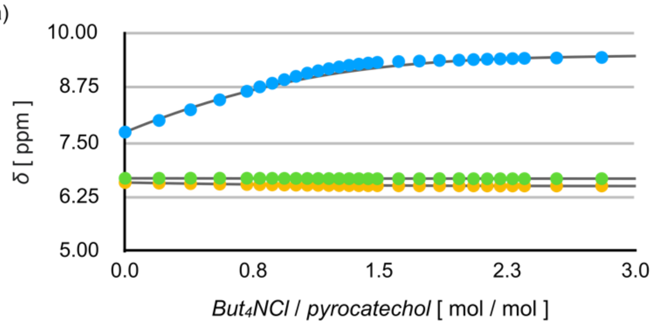

complex	constant	optimised	error
RCl	K_{11}	**496 M^{-1}**	**± 5.3%**

b)

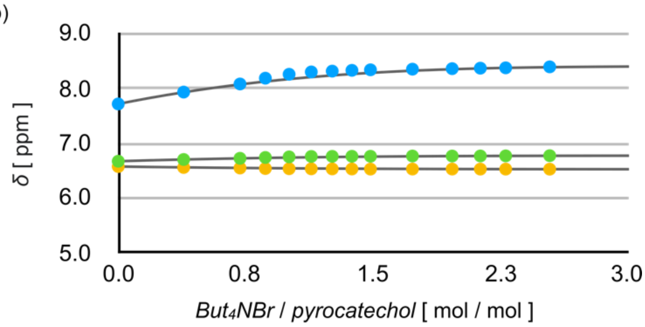

complex	constant	optimised	error
RBr	K_{11}	**676 M^{-1}**	**± 9.7%**

- **acetonitrile**

c)

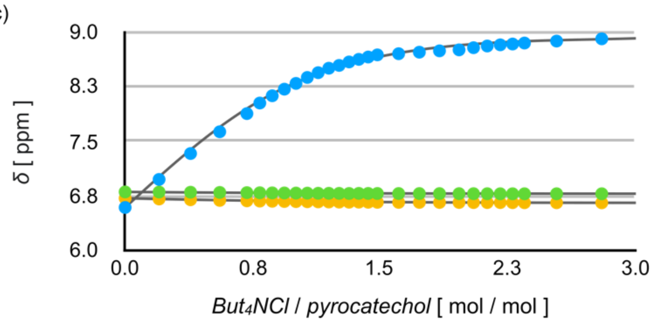

complex	constant	optimised	error
RCl	K_{11}	**476 M^{-1}**	**± 2.7%**

d)

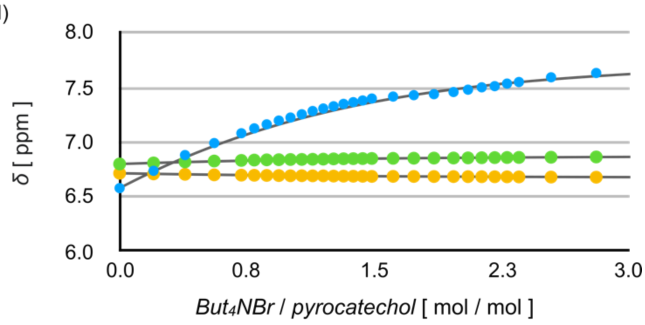

complex	constant	optimised	error
RBr	K_{11}	**131 M^{-1}**	**± 1.8%**

- **chloroform**

e)

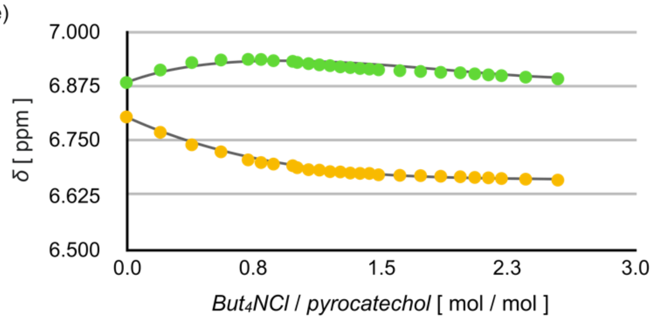

complex	constant	optimised	error
RCl	K_{11}	**347 M^{-1}**	**± 2.8%**
R_2Cl	K_{21}	**1 M^{-1}**	**± 1.3%**

f)

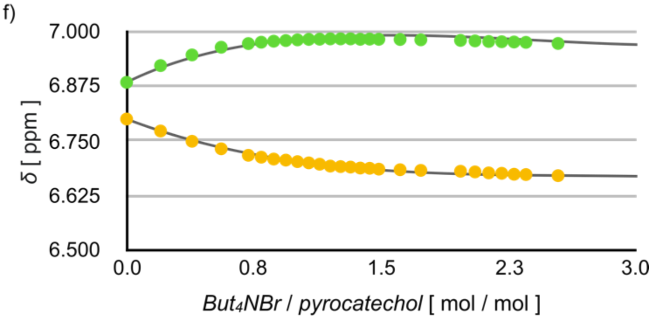

complex	constant	optimised	error
RBr	K_{11}	**154 M^{-1}**	**± 2.2%**
R_2Br	K_{21}	**0.2 M^{-1}**	**± 0.8%**

Figure S108. Titrations of pyrocatechol with (a) **But_4NCl** in THF-d_8, (b) **But_4NBr** in THF-d_8, (c) **But_4NCl** in MeCN-d_3, (d) **But_4NBr** in MeCN-d_3, (e) **But_4NCl** in $CDCl_3$, (f) **But_4NBr** in $CDCl_3$. Solid lines represent fitted curves, dahed line represents theoretical curve for 1:1. All titrations were performed using solutions of analyte: C (pyrocatechol) = 15 mM and titrant: C(pyrocatechol)= 15 mM + C(Alk_4NX) = 78 mM at 303 K, 400 MHz.

	pyrocatechol								
	THF			acetonitrile			Chloroform		
	RCl	RCl_2	R_2Cl	RCl	RCl_2	R_2Cl	RCl	RCl_2	R_2Cl
	K_{11} [M^{-1}]	K_{12} [M^{-1}]	K_{21} [M^{-1}]	K_{11} [M^{-1}]	K_{12} [M^{-1}]	K_{21} [M^{-1}]	K_{11} [M^{-1}]	K_{12} [M^{-1}]	K_{21} [M^{-1}]
**But_4NCl**	**497**	-	-	**476**	-	-	**347**	-	**1.2**
error	± 5.3%	-	-	±2.7%	-	-	± 2.8%	-	±1.3%
**But_4NBr**	**676**	-	-	**131**	-	-	**154**	-	**0.2**
error	± 9.7%	-	-	±1.8%	-	-	± 2.2%	-	±0.8%

Table S3. Comparison of the stability constant of the pyrocatechol complexes in various solvents (THF, acetonitrile, chloroform).

15. Interactions with smaller salts (P4C)

15.1 P4C + Pro_4NCl in THF

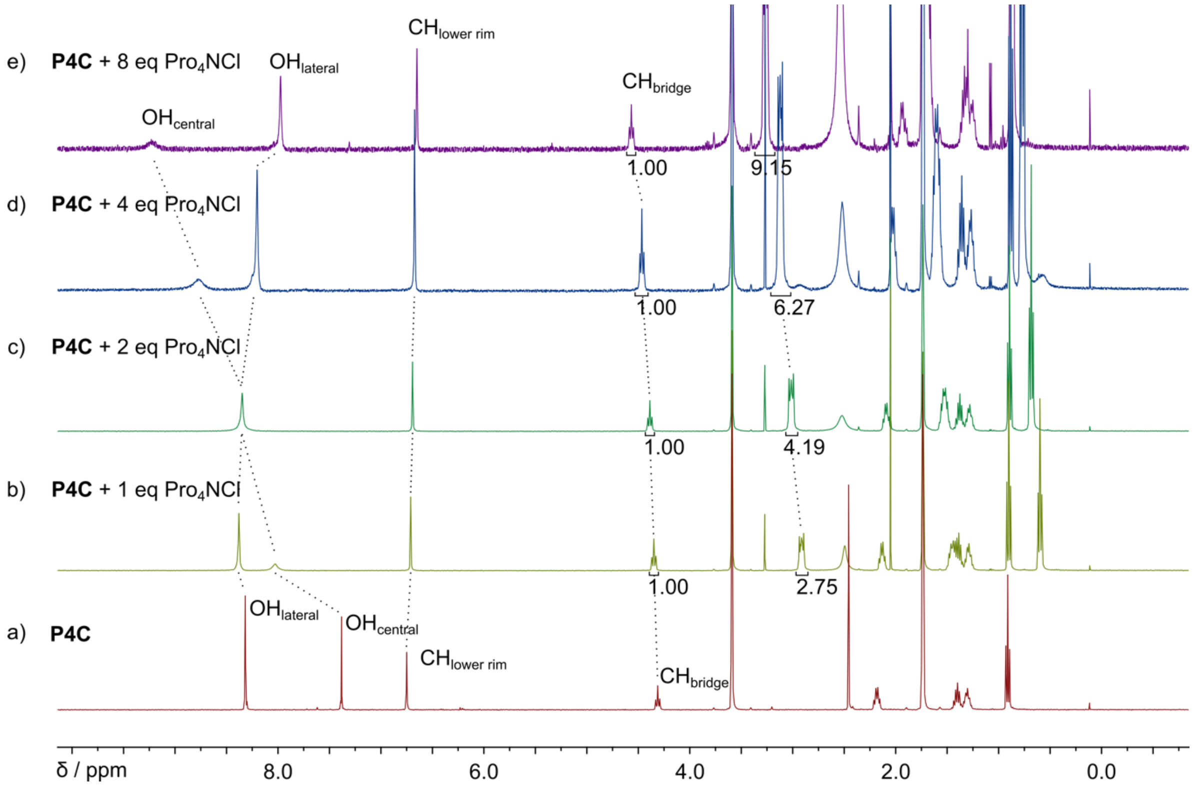

Figure S109. ^{1}H NMR of **P4C** complexes with Pro_4NCl in THF-d_8: (a) **P4C**; (b) **P4C** (10 mmol) + **Pro_4NCl** (10 mmol); (c) **P4C** (10 mmol) + **Pro_4NCl** (20 mmol); (d) **P4C** (10 mmol) + **Pro_4NCl** (40 mmol); (e) **P4C** (10 mmol) + **Pro_4NCl** (80 mmol); (400 MHz, 303 K). All samples were dissolved in THF-d_8 (0.7 ml).

15.2 P4C + Pro_4NBr in THF

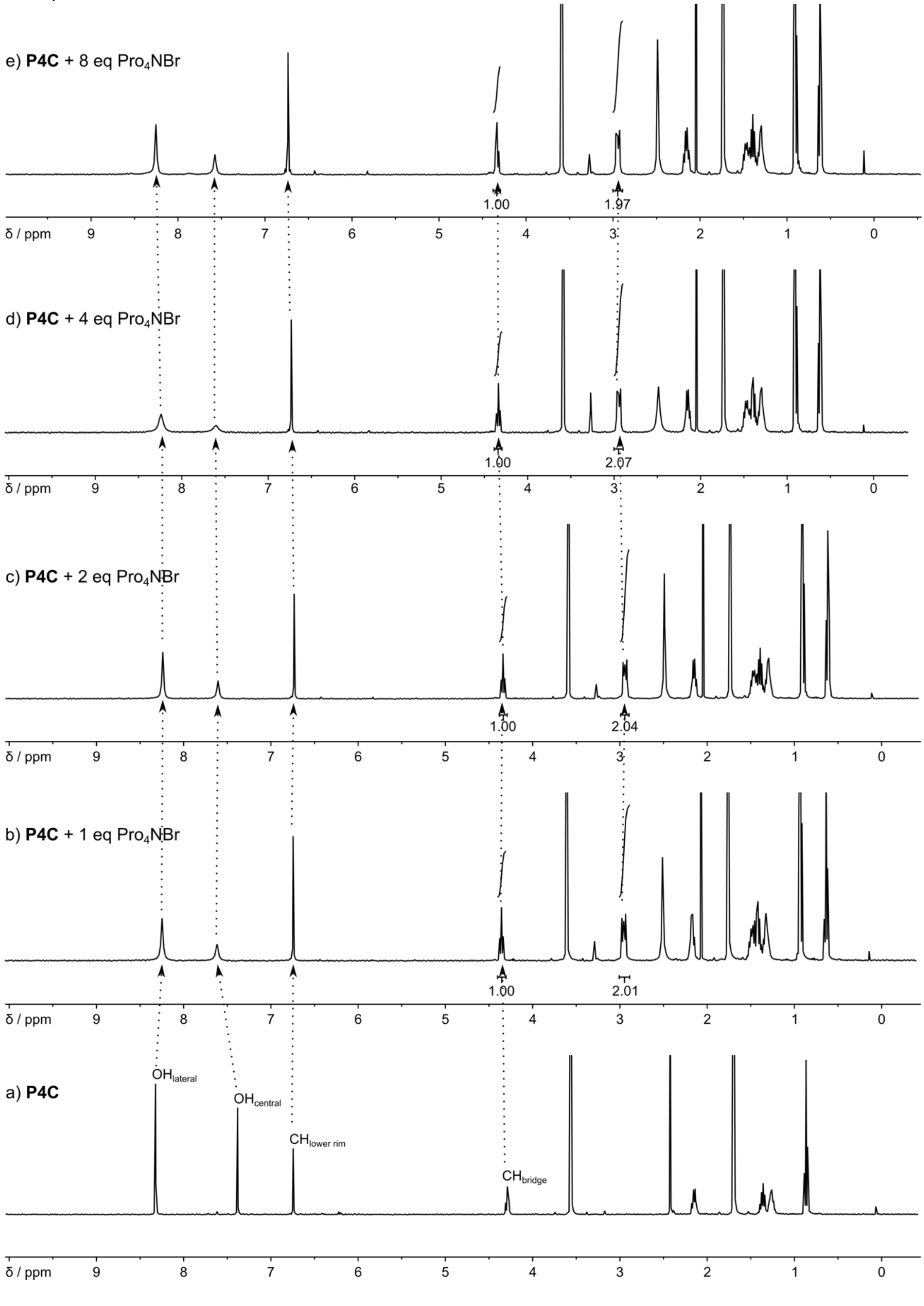

Figure S110. ^{1}H NMR of **P4C** complexes with Pro_4NBr in THF-d_8: (a) **P4C**; (b) **P4C** (10 mmol) + **Pro_4NBr** (10 mmol); (c) **P4C** (10 mmol) + **Pro_4NBr** (20 mmol); (d) **P4C** (10 mmol) + **Pro_4NBr** (40 mmol); (e) **P4C** (10 mmol) + **Pro_4NBr** (80 mmol); (400 MHz, 303 K). All samples were dissolved in benzene-d_6 (0.7 ml).

16. Control experiments

16.1 P4H

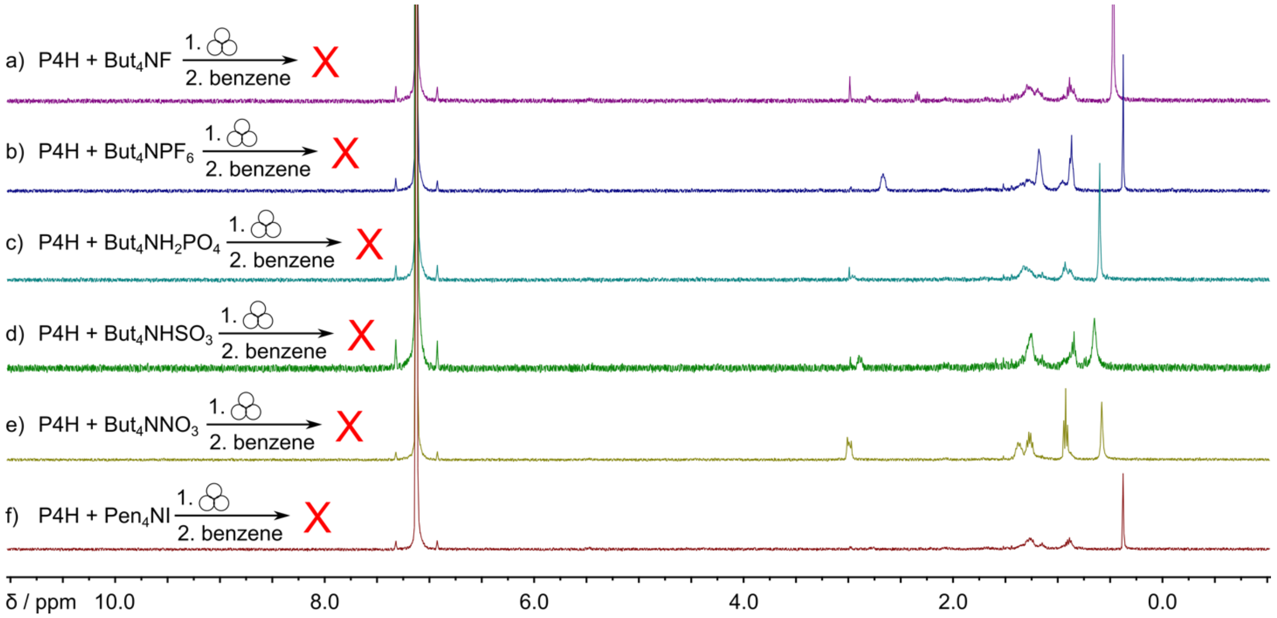

Figure S111. ^{1}H NMR in benzene-d_6 (400 MHz, 303 K) of mechanochemically treated samples: (a) **P4H** (10 mmol) + **But_4NF** (40 mmol); (b) **P4H** (10 mmol) + **But_4NPF_6** (40 mmol); (c) **P4H** (10 mmol) + **$But_4NH_2PO_4$** (40 mmol); (d) **P4H** (10 mmol) + **But_4NHSO_3** (40 mmol); (e) **P4H** (10 mmol) + **But_4NNO_3** (40 mmol); (f) **P4H** (10 mmol) + **But_4NI** (40 mmol). All samples were dissolved in benzene-d_6 (0.7 ml).

16.2 P4C

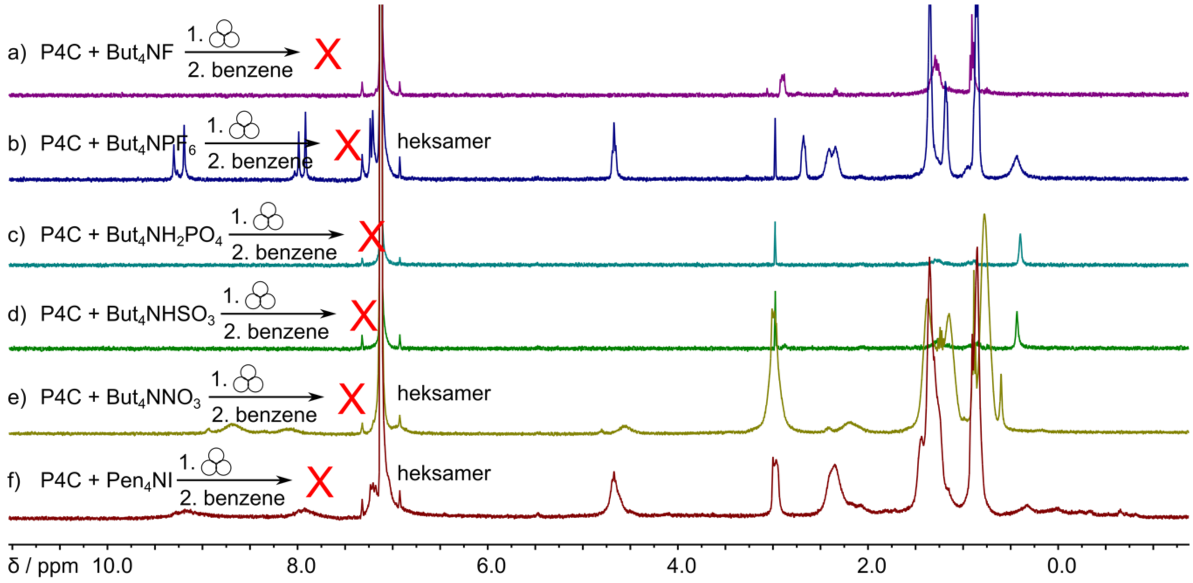

Figure S112. ^{1}H NMR in benzene-d_6 (400 MHz, 303 K) of mechanochemically treated samples: (a) **P4C** (10 mmol) + **But_4NF** (40 mmol); (b) **P4C** (10 mmol) + **But_4NPF_6** (40 mmol); (c) **P4C** (10 mmol) + **$But_4NH_2PO_4$** (40 mmol); (d) **P4C** (10 mmol) + **But_4NHSO_3** (40 mmol); (e) **P4C** (10 mmol) + **But_4NNO_3** (40 mmol); (f) **P4C** (10 mmol) + **But_4NI** (40 mmol). All samples were dissolved in benzene-d_6 (0.7 ml).

16.3 R4H

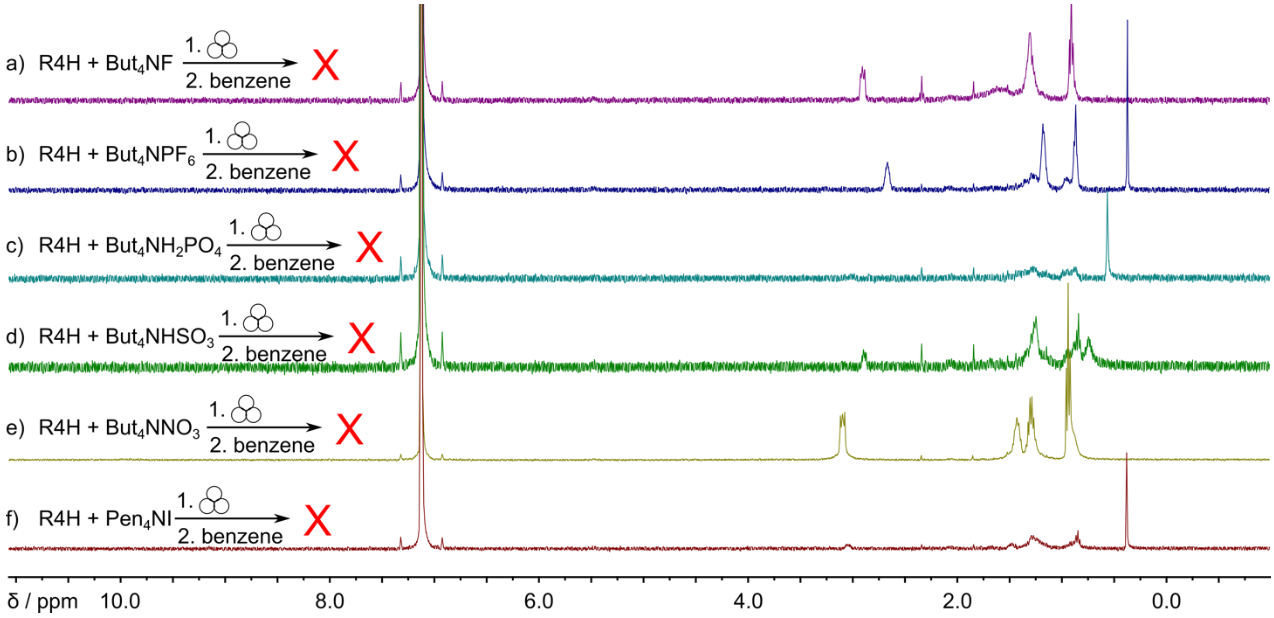

Figure S113. ^{1}H NMR in benzene-d_6 (400 MHz, 303 K) of mechanochemically treated samples: (a) **R4H** (10 mmol) + **But_4NF** (40 mmol); (b) **R4H** (10 mmol) + **But_4NPF_6** (40 mmol); (c) **R4H** (10 mmol) + **$But_4NH_2PO_4$** (40 mmol); (d) **R4H** (10 mmol) + **But_4NHSO_3** (40 mmol); (e) **R4H** (10 mmol) + **But_4NNO_3** (40 mmol); (f) **R4H** (10 mmol) + **But_4NI** (40 mmol). All samples were dissolved in benzene-d_6 (0.7 ml).

16.4 R4C

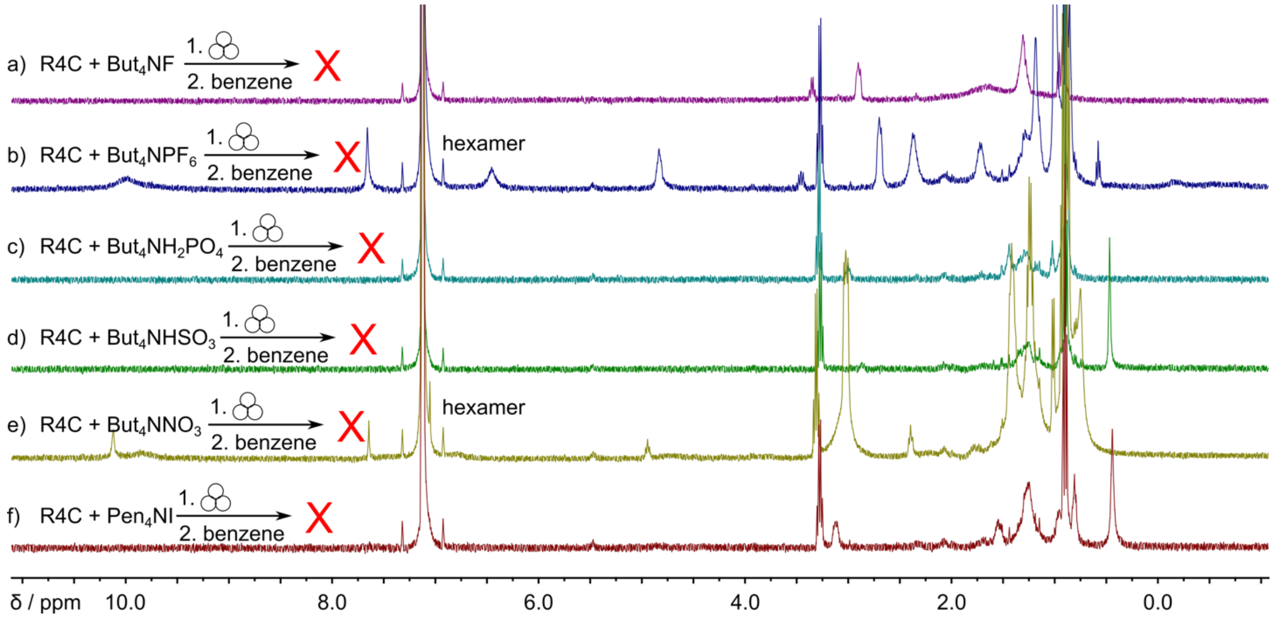

Figure S114. ^{1}H NMR in benzene-d_6 (400 MHz, 303 K) of mechanochemically treated samples: (a) **R4C** (10 mmol) + **But_4NF** (40 mmol); (b) **R4C** (10 mmol) + **But_4NPF_6** (40 mmol); (c) **R4C** (10 mmol) + **$But_4NH_2PO_4$** (40 mmol); (d) **R4C** (10 mmol) + **But_4NHSO_3** (40 mmol); (e) **R4C** (10 mmol) + **But_4NNO_3** (40 mmol); (f) **R4C** (10 mmol) + **But_4NI** (40 mmol). All samples were dissolved in benzene-d_6 (0.7 ml).

17. Model of tetramer $(M)_4(X^-)_{16}$

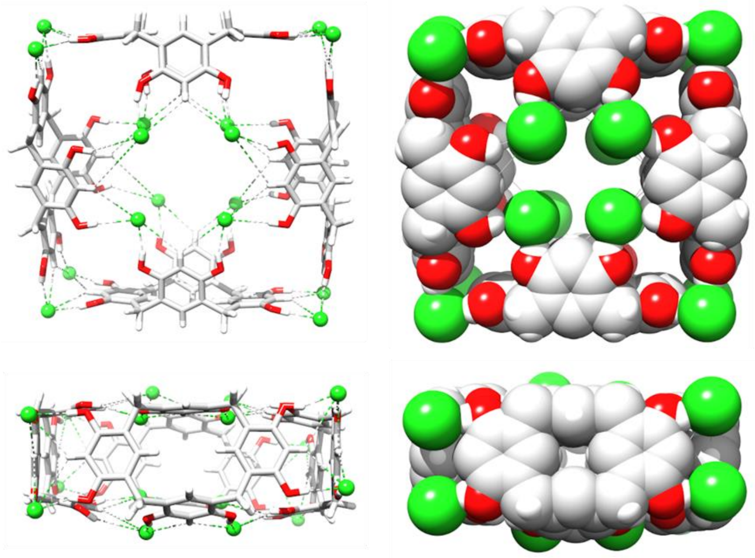

Figure S115. Tetramer a hypothetical anion-based structure $(M)_4(X^-)_{16.}$ Model was constructed using X-ray structure of anion-surrounded resorcinarene molecule (CCDC 195432).

18. Non-specific Alk_4NX aggregation in benzene and THF

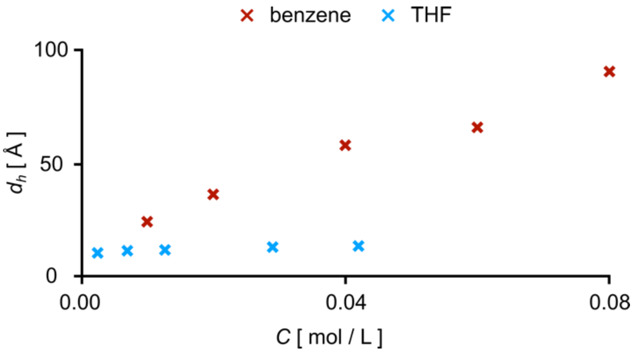

Figure S116. Aggregation of $\mathbf{Oct_4NBr}$ in benzene-d_6 and THF.

19. Influence of methanol and water on the stability of the capsule.

To the solution of **P4H** (1.55 mmol) and Pen4NCl (7.8mmol) in THF-d_8 (0.62 ml) methanol-d_3 or D_2O was added. After evry addition of methonol-d_3 or D_2O DOSY spectrum was recoreded.

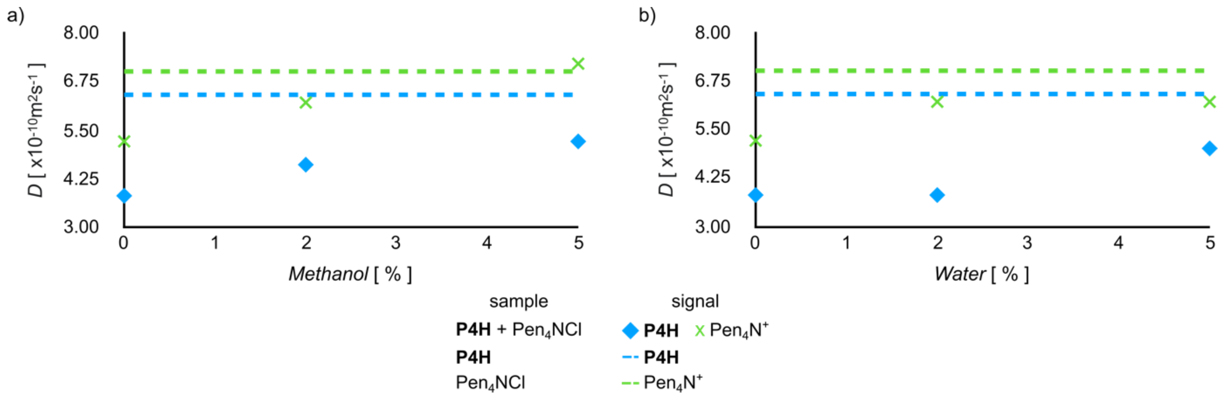

Figure S117. DOSY meserments after addition of (a) methanol; (b) water (600 MHz, 303 K, THF-d_8).

20. Comparison of changes of chemical shift sof Alk_4N+ signals

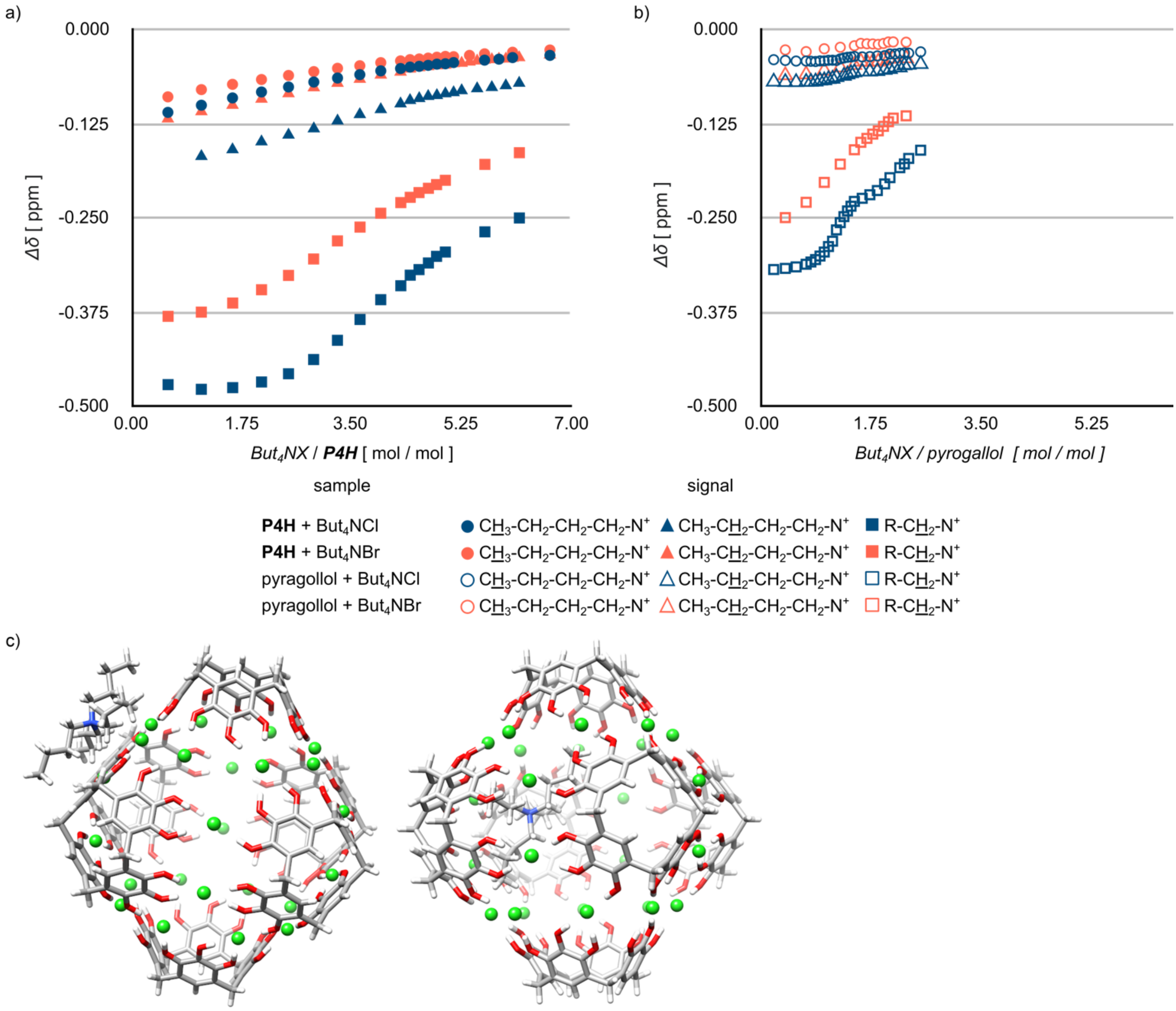

Figure S118. ^{1}H NMR chemical shifts change for $C\underline{H}_3$-CH_2-N^+, CH_3-$C\underline{H}_2$-N^+, R-$C\underline{H}_2$-N^+ durign titration (a) **P4H** (b) pyrogallol (400 MHz, 303 K, THF-d_8); (c) the model of the interaction.

21. Variable temperature ¹H NMR spectra

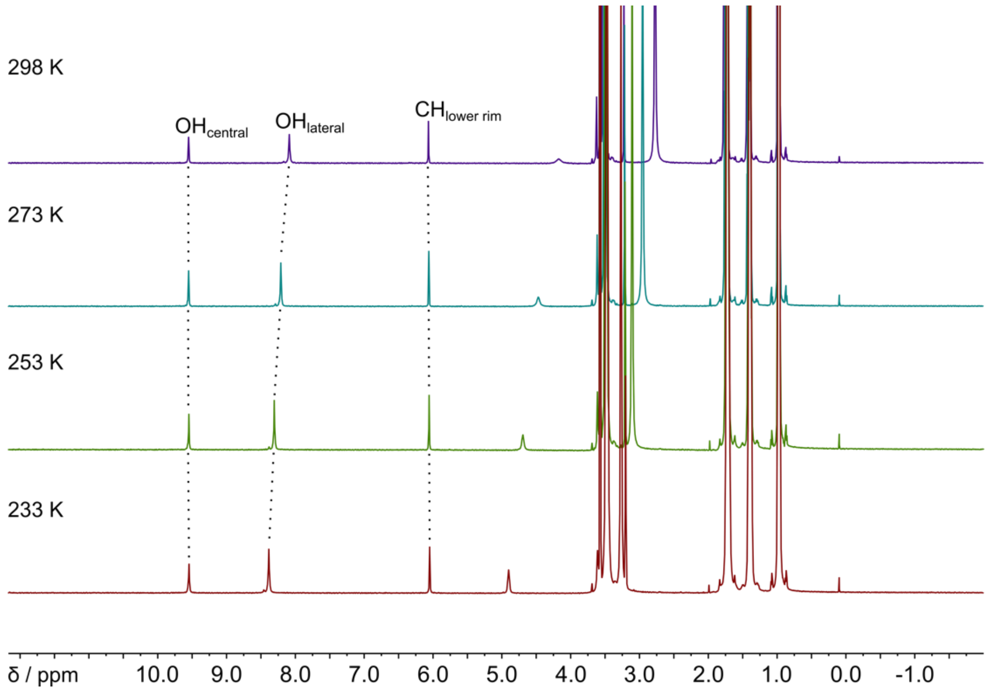

Figure S119. ¹H NMR variable temperature spectra of **P4H** + 5 eq **Pen_4NCl** (600 MHz, THF-d_8).

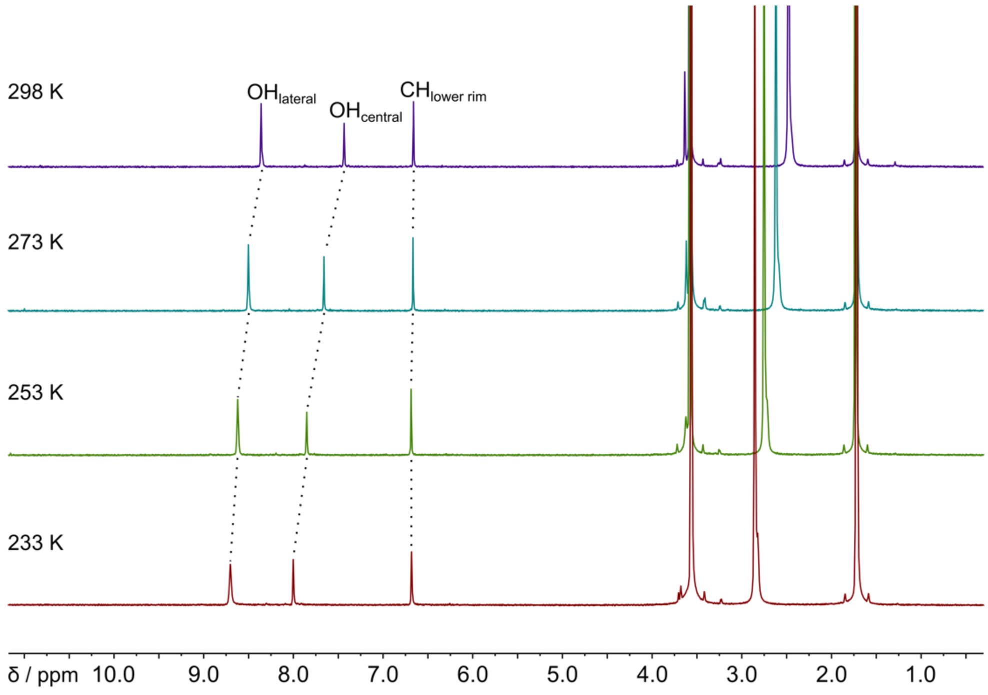

Figure S120. ¹H NMR variable temperature spectra of **P4H** (600 MHz, THF-d_8).

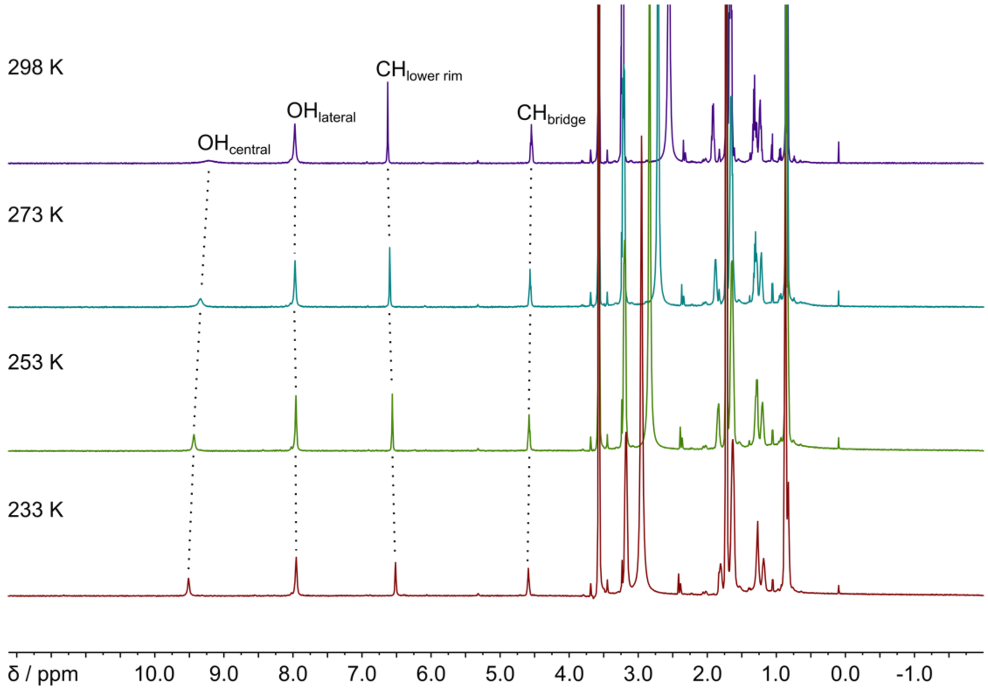

Figure S121. ^{1}H NMR variable temperature spectra of **P4C** + 6 eq **Pro_4NCl** (600 MHz, THF-d_8).

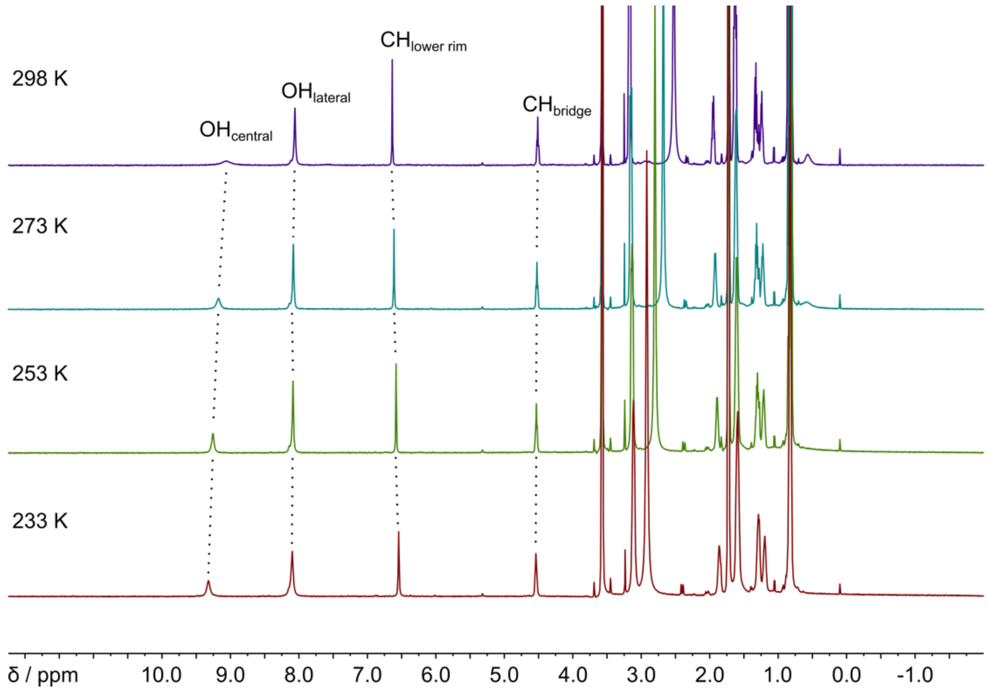

Figure S122. ^{1}H NMR variable temperature spectra of **P4C** + 4 eq **Pro_4NCl** (600 MHz, THF-d_8).

22. Repeatability

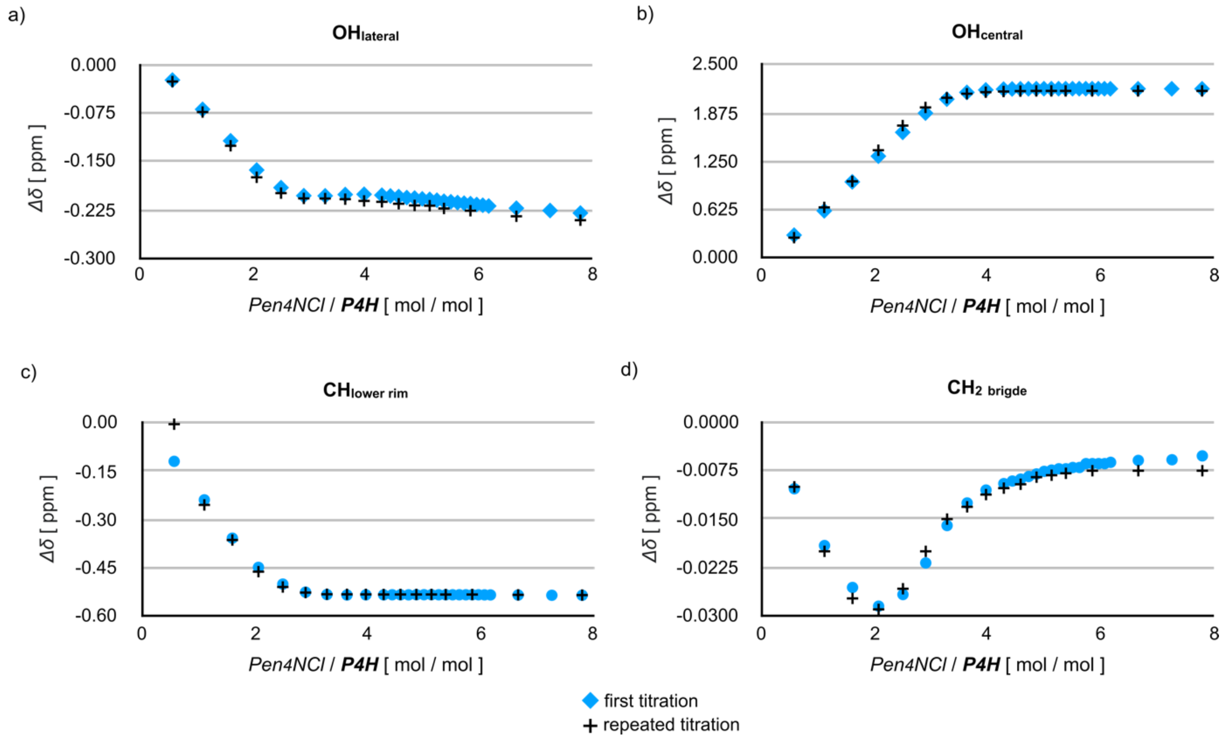

Figure S123. ^{1}H NMR titration curves obtained during two independent titrations of **P4H** (C = 5.0 mM) with titrant **P4H** (C = 5.0 mM) + **Pen$_4$NCl** (C = 75 mM). ^{1}H NMR chemical shifts change for: (a) $OH_{lateral}$; (b) $OH_{central}$; (c) $CH_{lowr\ rim}$; (d) $CH_{2\ bridge}$ (400 MHz, 303 K, THF-d_8).

23. DOSY titrations of resorcinol in THF

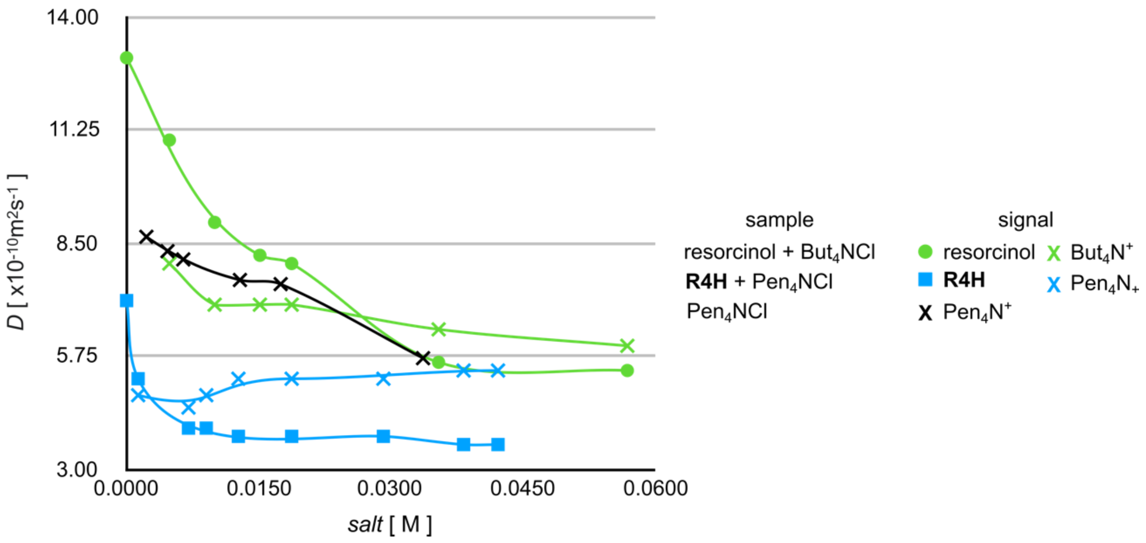

Figure S124.. Comparison of DOSY titration curves for titration of **resorcinol** (C = 10 mM) with titrant **resorcinol** (C = 10 mM) + **But_4NCl** (C = 65 mM) and **R4H** (C = 2.5 mM) with titrant **R4H** (C = 2.5 mM) + **Pen_4NCl** (C = 65.6 mM); THF (600 MHz, 303 K, THF-d_8).

resorcinol	But_4N^+	salt [M]
13.0		0.0000
11.0	8.0	0.0049
9.0	7.0	0.0100
8.2	7.0	0.0151
8.0	7.0	0.0188
5.6	6.4	0.0354
5.4	6.0	0.0569

Table S17. Data of DOSY titration of **resorcinol** (C = 10 mM) with titrant **resorcinol** (C = 10 mM) + **But_4NCl** (C = 65 mM).

24. DOSY titrations of pyrogallol in THF

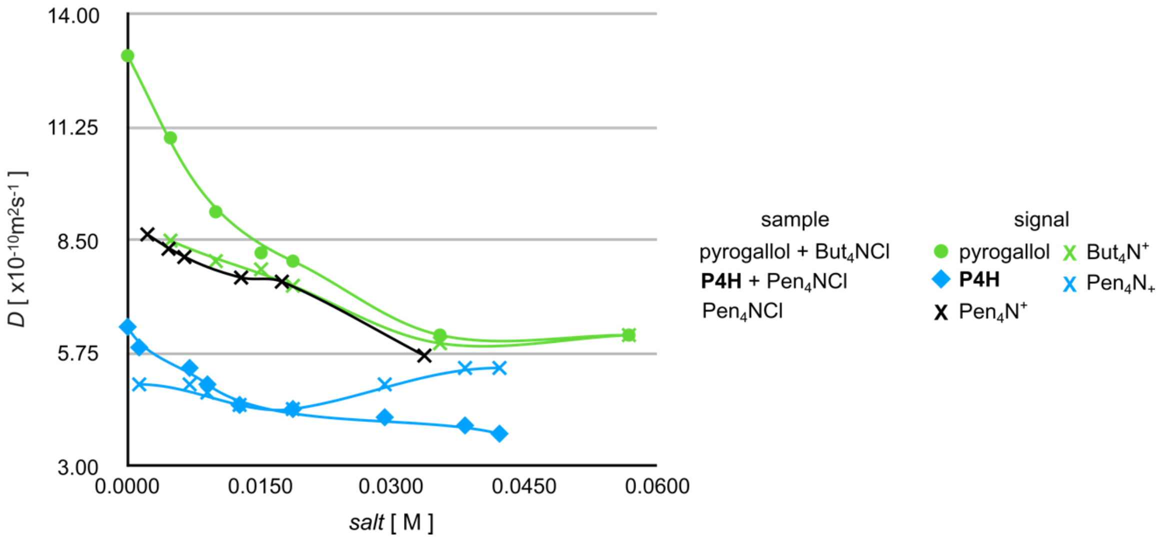

Figure S125. Comparison of DOSY titration curves for titration of **pyrogallol** (C = 10 mM) with titrant **pyrogallol** (C = 10 mM) + **But$_4$NCl** (C = 65 mM) and **P4H** (C = 2.5 mM) with titrant **P4H** (C = 2.5 mM) + **Pen$_4$NCl** (C = 65.6 mM); THF (600 MHz, 303 K, THF-d$_8$).

**pyrogallol**	**But$_4$N$^+$**	**salt [M]**
13.0		0.0000
11.0	8.5	0.0049
9.2	8.0	0.0100
8.2	7.8	0.0151
8.0	7.4	0.0188
6.2	6.0	0.0354
6.2	6.2	0.0569

Table S18. Data of DOSY for titration of **pyrogallol** (C = 10 mM) with titrant **pyrogallol** (C = 10 mM) + **But$_4$NCl** (C = 65 mM).

25. DOSY titrations of catechol in THF

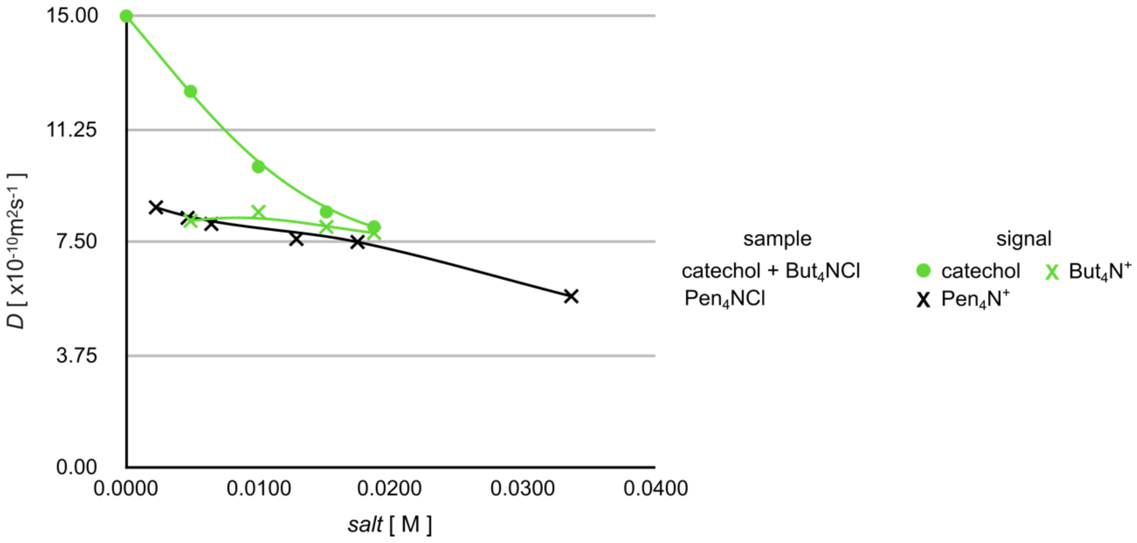

Figure S126. DOSY titration curves for titration of **catechol** (C = 10 mM) with titrant **catechol** (C = 10 mM) + **But_4NCl** (C = 65 mM) THF (600 MHz, 303 K, THF-d_8).

catechol	But_4N^+	salt [M]
15.0		0.0000
12.5	8.2	0.0049
10.0	8.5	0.0100
8.5	8.0	0.0151
8.0	7.8	0.0188

Table S19. Data of DOSY titration of **catechol** (C = 10 mM) with titrant **catechol** (C = 10 mM) + **But_4NCl** (C = 65 mM).

26. Comparison of the size of P4H and P5H capsules

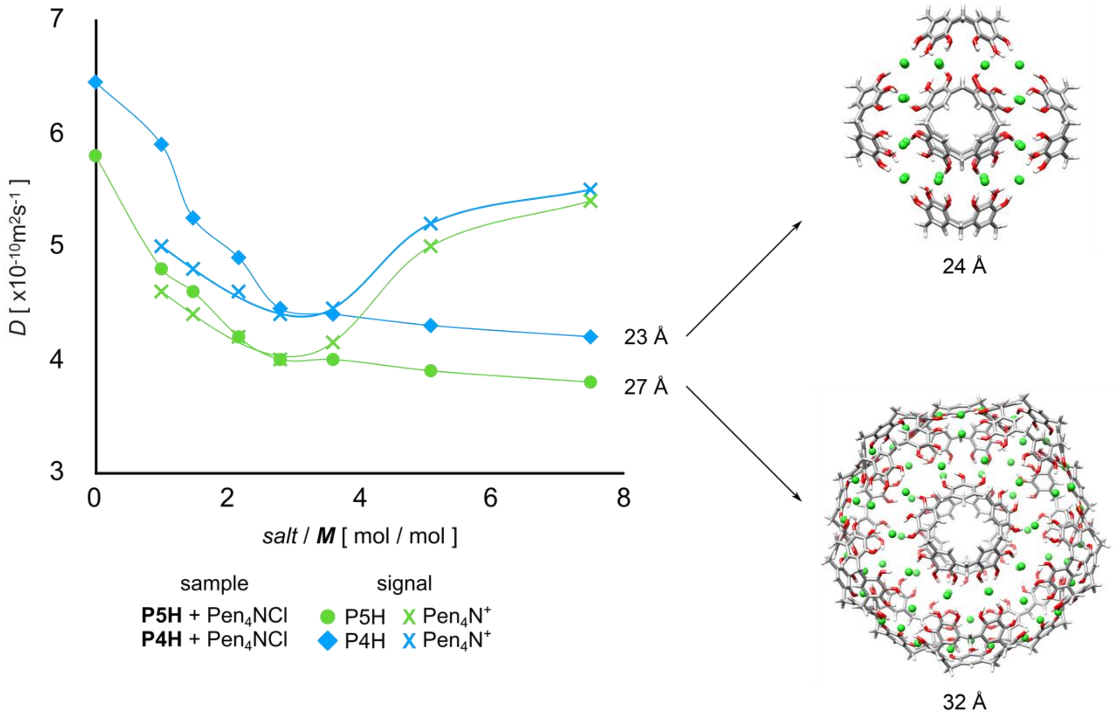

Figure S127. Comparison of DOSY titration curves for titration of **P5H** (C = 2.5 mM) with titrant **P5H** (C = 2.5 mM) + **Pen_4NCl** (C = 65.6 mM) and **P4H** (C = 2.5 mM) with titrant **P4H** (C = 2.5 mM) + **Pen_4NCl** (C = 65.6 mM); THF (600 MHz, 303 K, THF-d_8).

P4H + Pen_4NCl			P5H + Pen_4NCl		
salt / M [mol / mol]	P4H	Pen_4N^+	salt / M [mol / mol]	P5H	Pen_4N^+
0.00	6.5		0.00	5.8	
1.00	5.9	5.0	1.00	4.8	4.6
1.48	5.3	4.8	1.48	4.6	4.4
2.17	4.9	4.6	2.17	4.2	4.2
2.80	4.5	4.4	2.80	4.0	4.0
3.60	4.4	4.5	3.60	4.0	4.2
5.08	4.3	5.2	5.08	3.9	5.0
7.50	4.2	5.5	7.50	3.8	5.4

Table S20. Data of DOSY titration of **P4H** (C = 2.5 mM) with titrant **P4H** (C = 2.5 mM) + **Pen_4NCl** (C = 65.6 mM) and **P5H** (C = 2.5 mM) with titrant **P5H** (C = 2.5 mM) + **Pen_4NCl** (C = 65.6 mM).

27. DOSY measurement and calculation of the sizes

^{1}H DOSY experiments were performed on a Varian VNMRS-600 spectrometer at 298 K equipped with a 5 mm PFG AutoXID (^{1}H/X=^{15}N-^{31}P) probe. DOSY experiments were run with the DPFGDSTE (with convection compensation) pulse sequence for measurements in THF-d8, and benzene-d6 solutions. The gradient strengths were incremented as a square dependence in the range from 6 to 55 G/cm. 16 transients (with interleave option) were recorded for each increment with 3.2 s acquisition time and 1 s relaxation delay (overall experiment time *ca.* 18 - 20 min). The duration of magnetic field gradients (δ) was 1.5 – 2 ms, whereas a diffusion delay (Δ) was chosen as 50 – 150 ms. Other parameters include the following: a sweep width of 12 000 Hz, 32 K data points. The data were processed using Varian DOSY software.

The hydrodynamic diameters d_H ($d_H = 2r_H$) of the species were calculated using the Einstein-Stokes equation from D_{min}

$$r_H = \frac{k_b T}{6\pi\eta D}$$

$k_b - Boltzmann\ constant$
$T - temperature$
$\eta - viscosity\ coefficient$
$D - diffusion\ coeffcient$

28. Ab initio calculations

Calculation was performed within the density functional theory (DFT) approach using Gaussian 09 program suite.[v] Geometry was optimized with the B3LYP functional, employing the 6-31g basis set, in THF. Solvent effects were considered within the SCRF theory using the polarized continuum model (PCM) approach to model the interaction with the solvent.

Data for pyrogallol motif

Symbolic Z-matrix:

Charge = -3 Multiplicity = 1

O	-4.23582	2.97816	0.
O	-0.24133	5.74986	0.
O	-1.55282	3.06962	0.
C	-3.60916	4.19276	0.
C	-3.78672	6.59849	0.
H	-4.39495	7.49951	0.
C	-2.39624	6.70039	0.
C	-2.20182	4.27629	0.
H	-1.89896	7.66644	0.
C	-4.40117	5.34313	0.

C	-1.59489	5.54744	0.
H	-5.48197	5.23602	0.
H	0.29754	4.9197	0.
H	-0.56502	3.12643	0.
H	-3.59768	2.23347	0.
O	-0.46125	-5.1574	0.
O	-4.85886	-3.08393	0.
O	-1.88196	-2.87959	0.
C	-2.4267	-6.48309	0.
C	-1.82646	-5.222	0.
C	-2.60246	-4.04498	0.
C	-3.82111	-6.57864	0.
H	-4.29729	-7.55589	0.
C	-4.60458	-5.4254	0.
C	-4.00678	-4.15494	0.
H	-1.79355	-7.36553	0.
H	-5.68985	-5.47777	0.
H	-0.1354	-4.23242	0.
H	-2.42506	-2.05254	0.
H	-4.40935	-2.20217	0.
O	5.10019	-2.66593	0.
O	4.69707	2.17925	0.
O	3.43478	-0.19003	0.

C	5.60167	-1.39251	0.
C	6.82787	1.13996	0.
C	7.60782	-0.01986	0.
H	8.69224	0.05638	0.
H	7.58881	-2.18867	0.
H	7.27551	2.12951	0.
C	7.00083	-1.27499	0.
C	4.80428	-0.23131	0.
C	5.43562	1.02924	0.
H	2.99008	-1.07389	0.
H	4.11181	-2.71752	0.
H	3.73308	1.99895	0.
Cl	2.03669	-2.96975	0.
Cl	1.55353	3.2487	0.
Cl	-3.59022	-0.27895	0.

Data for resorsinol motif

Symbolic Z-matrix:

Charge = -3 Multiplicity = 1

Symbol	X	Y	Z
O	5.0360719	0.0060445	0.0397339
O	3.3131769	4.5058638	-0.3230918
C	5.1958271	1.3559554	0.0575045

C	6.6693077	3.2429564	0.2741709
H	7.6613213	3.6452116	0.4332899
C	5.5955815	4.1043844	0.0983370
C	4.1007784	2.2129953	-0.1334332
H	5.7261723	5.1781111	0.1139325
C	6.4755541	1.8668415	0.2560938
C	4.3091157	3.6008251	-0.1114712
H	7.2954454	1.1758151	0.3978226
H	2.4126227	4.1384579	-0.1695224
H	4.1067478	-0.2511357	-0.0953100
O	-1.8441034	-3.9887781	2.5066293
O	2.4977002	-5.0854502	0.6918983
C	-0.6702549	-5.8949251	3.2642275
C	-0.7339488	-4.7713079	2.4454296
C	0.3148716	-4.4600730	1.5664166
C	0.4517573	-6.7139671	3.2169144
H	0.5082618	-7.5872014	3.8537118
C	1.5038347	-6.4126759	2.3636363
C	1.4449833	-5.2914178	1.5313763
H	-1.4995370	-6.1041669	3.9263689
H	2.3874279	-7.0350672	2.3172089
H	-1.7799543	-3.2187183	1.9147339
H	2.5355219	-4.1730939	0.3231986

O	-5.3978986	0.5676480	1.9422808
O	-2.1579595	4.1506147	1.9995203
C	-4.9389219	1.7809146	2.3594002
C	-4.1663154	4.3261999	3.2324890
C	-5.3800539	3.7847119	3.6386931
H	-6.0278694	4.3490275	4.2969343
H	-6.7003408	2.0809202	3.5243106
H	-3.8442558	5.3064756	3.5567212
C	-5.7620643	2.5199183	3.2128303
C	-3.7125085	2.3188015	1.9390851
C	-3.3352082	3.5932254	2.3897920
H	-4.6807316	-0.0283861	1.6252781
H	-1.6313116	3.5420869	1.4516131
Cl	-3.1626216	-1.3491453	1.0786491
Cl	0.4993548	3.4337136	0.2536554
Cl	2.7217661	-2.2388880	-0.4325688
H	-3.1035728	1.8884285	1.3129316
H	3.2463182	1.7806129	-0.3096005
H	0.1734115	-3.6832685	0.9966310

[i] Bourgeois, J. -M.; Stoeckli-Evans, H. Synthesis of New Resorcinarenes Under Alkaline Conditions. *Helv. Chim.* **2005**, 88 (10), 2722-2730. DOI:10.1002/hlca.200590211

[ii] Miao, S.; Adams, R. D.; Guo, D. -S.; Zhangab, Q. -F. Structural conformers of symmetry substituted resorcin[4]arenes. *J. Mol. Struct*. **2003**, 659, 119-128. DOI: 10.1016/j.molstruc.2003.08.004

[iii] Wagle, D. V.; Kelley, S. P.; Baker, G. A.; Sikligar, K.; Atwood, J. L. An Indium-Seamed Hexameric Metal–Organic Cage as an Example of a Hexameric Pyrogallol[4]arene Capsule Conjoined Exclusively by Trivalent Metal Ions. *Angew. Chem. Int.Ed*. **2020**, 59, 8062-8065. DOI: 10.1002/anie.201914693

[iv] Chwastek, M.; Szumna, A. Higher Analogues of Resorcinarenes and Pyrogallolarenes: Bricks for Supramolecular *Org. Lett.*, **2020**, 22, 6838−6841. DOI:10.1021/acs.orglett.0c02357

[v] Gaussian 09, Revision E.01, Frisch, J. M.; Trucks, G. W.; Schlegel, H. B.; Scuseria, G. E.; Robb, M. A.; Cheeseman, J. R.; Scalmani, G.; Barone, V.; Mennucci, B.; Petersson, G. A.; Nakatsuji, H.; Caricato, M.; Li, X.; Hratchian, H. P.; Izmaylov, A. F.; Bloino, J.; Zheng, G.; Sonnenberg, J. L.; Hada, M.; Ehara, M.;

Toyota, K.; Fukuda, R.; Hasegawa, J.; Ishida, M.; Nakajima, T.; Honda, Y.; Kitao, O.; Nakai, H.; Vreven, T.; Montgomery, J. A.; Peralta, J. E.; Ogliaro, F.; Bearpark, M.; Heyd, J. J.; Brothers, E.; Kudin, K. N.; Staroverov, V. N.; Kobayashi, R.; Normand, J.; Raghavachari, K.; Rendell, A.; Burant, J. C.; Iyengar, S. S.; Tomasi, J.; Cossi, M.; Rega, N.; Millam, J. M.; Klene, M.; Knox, J. E.; Cross, J. B.; Bakken, V.; Adamo, C.; Jaramillo, J.; Gomperts, R.; Stratmann, R. E.; Yazyev, O.; Austin, A. J.; Cammi, R.; Pomelli, C.; Ochterski, J. W.; Martin, R. L.; Morokuma, K.; Zakrzewski, V. G.; Voth, G. A.; Salvador, P.; Dannenberg, J. J.; Dapprich, S.; Daniels, A. D.; Farkas, Ö.; Foresman, J. B.; Ortiz, J. V.; Cioslowski J.; Fox, D. J. Gaussian, Inc., Wallingford CT, 2009.
